# Visible light driven deuteration of formyl C–H and hydridic C(sp^3^)–H bonds in feedstock chemicals and pharmaceutical molecules†

**DOI:** 10.1039/d0sc02661a

**Published:** 2020-08-05

**Authors:** Yulong Kuang, Hui Cao, Haidi Tang, Junhong Chew, Wei Chen, Xiangcheng Shi, Jie Wu

**Affiliations:** Department of Chemistry, National University of Singapore 3 Science Drive 3 117543 Republic of Singapore chmjie@nus.edu.sg

## Abstract

Deuterium labelled compounds are of significant importance in chemical mechanism investigations, mass spectrometric studies, diagnoses of drug metabolisms, and pharmaceutical discovery. Herein, we report an efficient hydrogen deuterium exchange reaction using deuterium oxide (D_2_O) as the deuterium source, enabled by merging a tetra-*n*-butylammonium decatungstate (TBADT) hydrogen atom transfer photocatalyst and a thiol catalyst under light irradiation at 390 nm. This deuteration protocol is effective with formyl C–H bonds and a wide range of hydridic C(sp^3^)–H bonds (*e.g.* α-oxy, α-thioxy, α-amino, benzylic, and unactivated tertiary C(sp^3^)–H bonds). It has been successfully applied to the high incorporation of deuterium in 38 feedstock chemicals, 15 pharmaceutical compounds, and 6 drug precursors. Sequential deuteration between formyl C–H bonds of aldehydes and other activated hydridic C(sp^3^)–H bonds can be achieved in a selective manner.

## Abstract 2

A selective hydrogen deuterium exchange reaction with formyl C–H bonds and a wide range of hydridic C(sp^3^)–H bonds has been achieved by merging tetra-*n*-butylammonium decatungstate photocatalyst and a thiol catalyst under 390 nm light irradiation.

## Graphical abstract

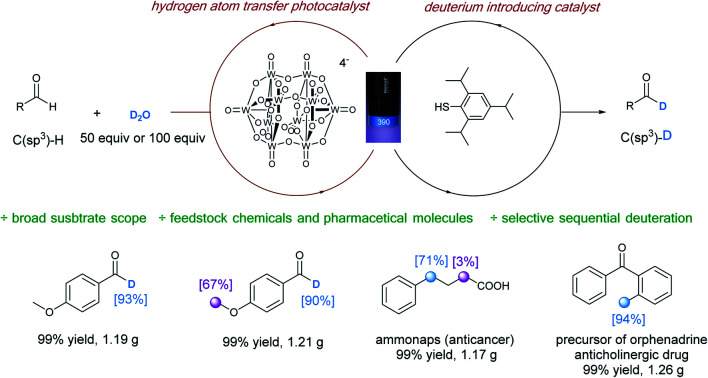

## Introduction

Deuterium labelling of organic molecules is of vital importance in many aspects.^1^ Deuterated compounds have been widely applied for the investigation of reaction mechanisms^2^ and diagnosis of drug metabolisms.^3^ The introduction of deuterium into pharmaceutical molecules can alter the absorption, distribution, metabolism, excretion, and toxicity (ADME-Tox) properties.^1^ In 2017, the U.S. Food and Drug Administration (FDA) approved SD-809 (Austedo) for the treatment of chorea associated with Huntington's disease.^4^ Austedo has OCD_3_ groups in place of the two OCH_3_ groups on the phenyl ring of its parent FDA approved drug, Xenazine, but shows similar efficacy at lower doses and has a longer duration of action. Currently, there are many other deuterium-containing drugs under clinical testing, such as d^6^-dextromethorphan (AVP-786).^5^ The importance of introducing deuterium into drug molecules originates from its significant kinetic isotope effects (KIEs) in enzyme-catalyzed metabolizing reactions (*e.g.*, cytochrome P450 enzyme,^6^ aldehyde oxidase,^7^ and monoamine oxidase^8^), resulting in a slower oxidative metabolism of the drug compounds. Therefore, it is important to incorporate deuterium atoms at readily metabolized sites in pharmaceutical compounds, especially the activated hydridic C(sp^3^)–H bonds.

**Scheme 1:**
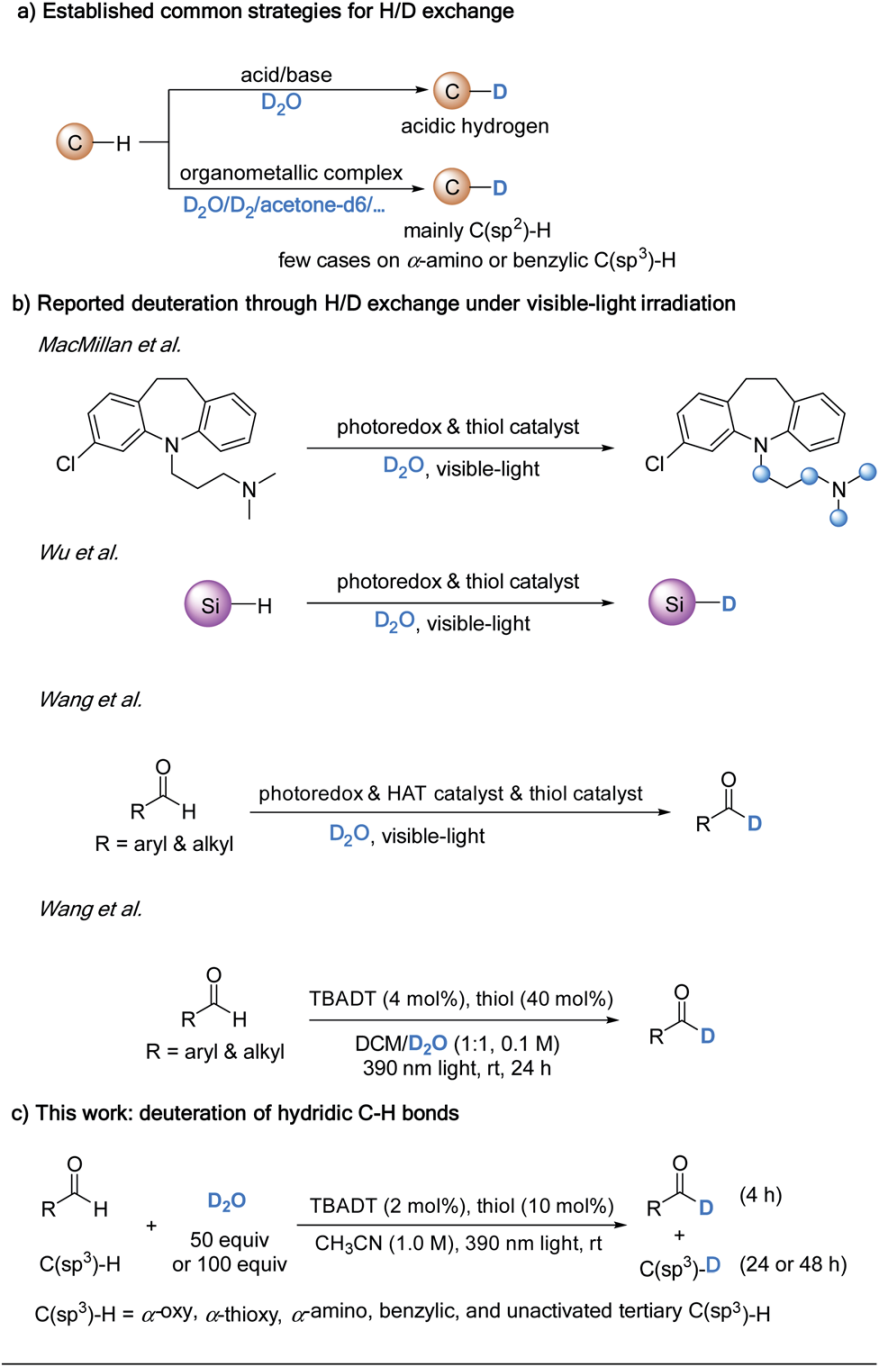
Hydrogen–deuterium exchange reactions.

Deuterium can be incorporated into a molecule by halogen/deuterium exchange^9^ or by reductive deuteration,^10^ which require the preparation of suitable precursors. A more atom- and step-economic pathway for the introduction of deuterium is hydrogen deuterium (H/D) exchange.^11^ Two well-known methods for H/D exchange are pH-dependent^12^ and transition-metal catalyzed H/D exchange.^13^ While the acid/base system is used to achieve the deuteration of acidic hydrogens, transition-metal catalysis is mainly applied to C(sp^2^)–H deuteration. Transition-metal catalysis has also been occasionally utilized in H/D exchange of C(sp^3^)–H bonds, including α-amino^14^ and benzylic C–H bonds^15^ (Scheme 1a).

In comparison, the emergence of photocatalysis has provided enormous opportunities for regioselective C(sp^3^)–H functionalization.^16^ MacMillan *et al.* have reported a hydrogen isotope exchange at α-amino C(sp^3^)–H bonds by the combination of a photoredox catalyst to generate the carbon radical, and a thiol catalyst to transfer the isotopic atom from isotopically labelled water (Scheme 1b).^17^ Our lab has reported a related visible-light-mediated deuteration of silanes using D_2_O as the deuterium source through the synergistic effect of a photoredox catalyst and a thiol catalyst.^18^ However, the substrate scope of photoredox catalyzed C(sp^3^)–H functionalization is limited, because it is successful only when the redox potential of the C–H substrate is matched with that of the photocatalyst.^19^ Wang *et al.* have recently reported a H/D exchange at formyl groups of both aromatic and aliphatic aldehydes, by merging a photoredox catalyst, a hydrogen atom transfer (HAT) catalyst, and a thiol catalyst.^20^

Direct HAT photocatalysis offers a broad substrate scope in C(sp^3^)–H functionalization without the need to match redox potentials.^21,22^ Among the direct HAT photocatalysts, tetra-*n*-butylammonium decatungstate (TBADT) represents a versatile one which can activate formyl C–H and a wide range of C(sp^3^)–H bonds.^23^ We envision that by taking advantage of a direct HAT photocatalyst,^21^ a variety of formyl C–H and hydridic C(sp^3^)–H bonds can be deuterated. In line with our continuing interest in direct HAT photocatalyzed C–H functionalization,^22^ we report herein that by implementing the cooperative effect of TBADT with a thiol catalyst, various types of formyl C–H and electron-rich C(sp^3^)–H bonds can be deuterated using deuterium oxide as the source of deuterium under irradiation by near-ultraviolet light (390 nm). Notably, during the process of our study, a similar catalytic system was reported by Wang *et al.* for the deuteration of only formyl C–H bonds.^24^ In stark contrast, with the addition of tetra-*n*-butylammonium bromide (TBAB) in our hand, the HAT efficiency was significantly improved, and the deuteration scope can go far beyond formyl C–H bonds, enabling the selective deuteration of a wide range of hydridic C(sp^3^)–H bonds, including α-oxy, α-thioxy, α-amino, benzylic and unactivated tertiary C(sp^3^)–H bonds (Scheme 1c). This provides a convenient and effective protocol for the regioselective incorporation of deuterium into drug molecules and their precursors and promises to find wide application in biological and pharmaceutical studies.

## Results and discussion

Our investigation began with the deuteration of 4-bromobenzaldehyde as a model reaction using D_2_O as the deuterium source. Direct HAT photocatalysts in combination of thiol catalysts (BDE of 2,4,6-triisopropylbenzenethiol = 80 kcal mol^−1^)^10*b*^ were evaluated because the formyl radical could not directly abstract deuterium atom from D_2_O (BDE of H_2_O = 119 kcal mol^−1^*vs.* BDE of formyl C(sp^2^)–H in benzaldehyde = 86.9 kcal mol^−1^).^25^ After extensive evaluation (Table 1), we established that the combination of TBADT (2 mol%), 2,4,6-triisopropylbenzenethiol (10 mol%), and D_2_O (50 equiv.) in acetonitrile (1.0 M) irradiated by 390 nm Kessil light (80 W) was the optimal condition, affording product **1** in 85% isolated yield with 94% deuterium incorporation within 4 hours (Table 1, entry 1). Light, TBADT, the thiol catalyst, and solvent were of significance in achieving a high deuteration efficiency (entries 2–5). Changing TBADT to other types of HAT photocatalysts (entries 6–8) or changing the thiol catalyst (entries 9–11) resulted in lower levels of deuterium incorporation. A less concentrated reaction or switching CH_3_CN to other solvents would cause decreasing of the deuteration ratio (entries 12–16).

**Table 1:** Survey of the deuteration conditions^a^

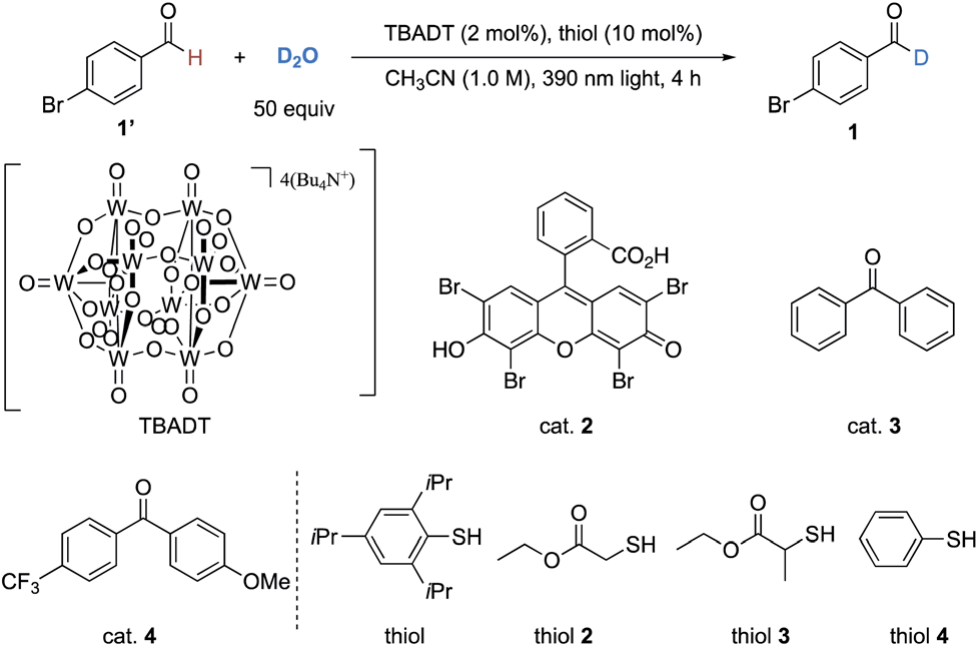			
Entry	Deviation	Yield^b^ (%)	D-inc.^c^ (%)
1	No change	99% (85%)^d^	94%
2	No light	99%	0%
3	No TBADT	99%	19%
4	No thiol	99%	30%
5	Neat	99%	14%
6^*e*^	Cat. **2** (5 mol%) instead of TBADT	99%	0%
7	Cat. **3** (10 mol%) instead of TBADT	94%	38%
8	Cat. **4** (10 mol%) instead of TBADT	99%	28%
9	Thiol **2** instead of thiol	99%	40%
10	Thiol **3** instead of thiol	99%	27%
11	Thiol **4** instead of thiol	99%	83%
12	CH_3_CN (0.5 M)	99%	89%
13	CH_3_CN (0.2 M)	99%	88%
14	Acetone (1.0 M)	99%	87%
15	CH_2_Cl_2_ (1.0 M)	99%	90%
16	*t*BuOH (1.0 M)	99%	19%

a Reactions were conducted following the optimal conditions with indicated deviation.

b Yield was determined by the analysis of ^1^H NMR spectra of the crude product mixture using CH_2_Br_2_ as an internal standard.

c Deuterium incorporation was determined by the analysis of ^1^H NMR spectra of the products.

d Yield in the parentheses was the isolated yield.

e Reaction was irradiated under a blue light emitting diode (LED, 24 W) for 24 h.

With the optimal conditions in hand, we evaluated the reaction scope with respect to aldehydes (Table 2). The results were similar to those of Wang's, but lower amounts of catalysts and D_2_O and a shorter reaction time were required. The transformations performed well with aromatic aldehydes to give products in generally good to excellent (70% to 96%) isolated yields and deuterium incorporation (88% to 97%). Functionalities ranging from electron-withdrawing substituents such as halides (**1–3**), trifluoromethyl (**7**), cyanides (**8**), and boronate esters (**9**), to electron-donating substituents, including amides (**5**), phenols (**6**), and ethers (**10**), were well tolerated. However, 4-acetoxybenzaldehyde gave very low deuterium incorporation (14%) under these conditions. Changing the light source to a 365 nm LED (24 W) with an extended reaction time (24 h) could deliver product **4** with 93% deuterium incorporation. Heteroaromatic aldehydes like 2-thenaldehyde proceeded well to afford product **12** with high deuterium incorporation. This protocol could also be applied to both linear and branched alkyl aldehydes with good regioselectivity and excellent deuterium incorporation (**13–16**). Even the volatile hexanal (bp = 129 °C) underwent 90% selective deuteration of its formyl group (**16**).

**Table 2:** Substrate scope of formyl deuteration of aldehydes^a^

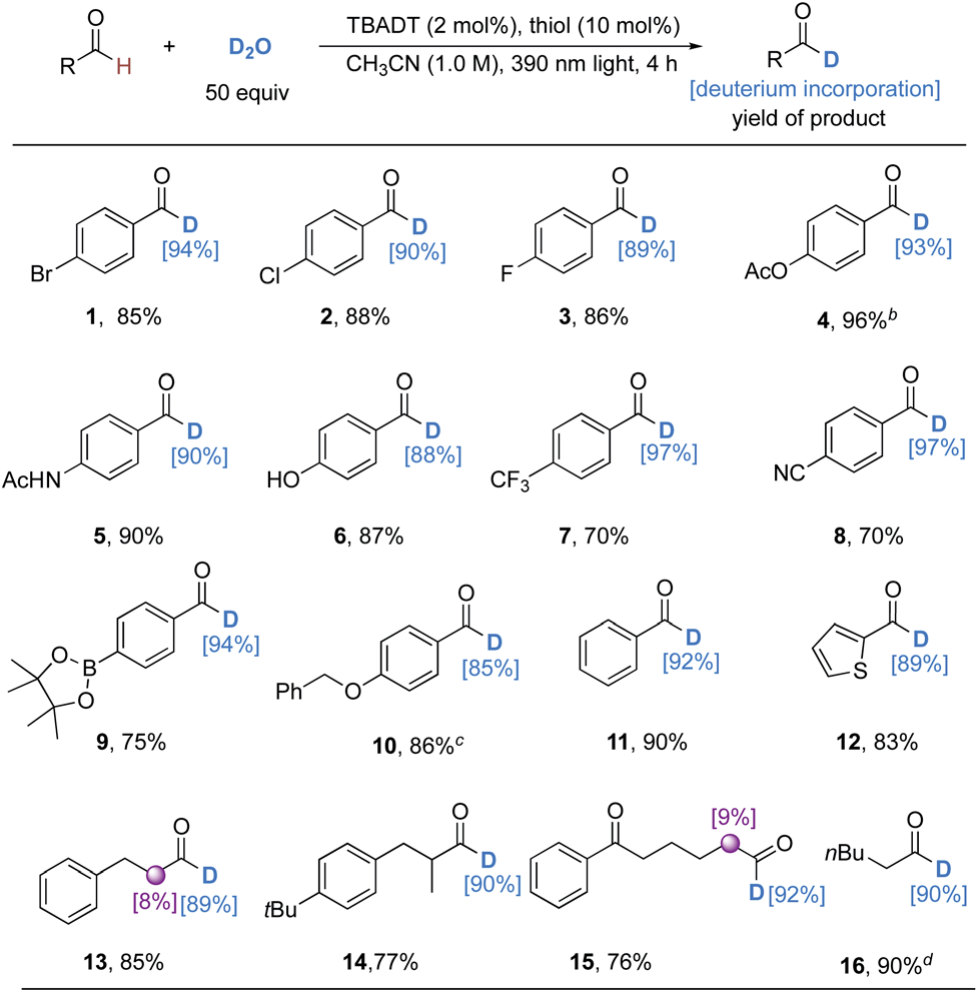

a Unless otherwise noted, the reactions were conducted under the optimized conditions for 4 h. Isolated yields are given. The deuterium incorporation was determined by the analysis of the ^1^H NMR spectra of the crude products.

b 365 nm LED light (24 W) was used instead of 390 nm Kessil light (80 W) for 24 h.

c Reaction time is 8 h.

d Due to the volatility of product **16**, the results were determined by the analysis of the crude ^1^H NMR spectra with the reaction conducted in CD_3_CN (1.0 M) using CH_2_Br_2_ as an internal standard.

TBADT is a well-known powerful photocatalyst which can activate not only formyl C–H bonds, but also activated and unactivated hydridic C(sp^3^)–H bonds.^23^ During our study of deuteration of the formyl C–H bond in 4-methoxybenzaldehyde, we observed that this formyl C–H bond underwent H/D exchange in a selective fashion within the first 4 h of light irradiation (**17**), and the methoxy group was subsequently deuterated with 70% deuterium incorporation, giving **17′** by prolonging the reaction time to 48 h, assisted by the addition of a phase transfer catalyst TBAB (20 mol%) to accelerate the reaction rate by transferring more decatungstate anions ([W_10_O_32_]^4−^) to the organic phase. This selective sequential deuteration can be extended to aldehydes bearing α-oxy, *α*-thioxy, or benzylic C(sp^3^)–H bonds (**17–20**, **17′–20′**, Table 3a). The examination of the site selectivity established a trend that more hydridic C(sp^3^)–H bonds undergo C–H isotopic exchange to a higher extent. High levels of deuterium incorporation are beneficial in the application of deuterated compounds as internal standards in pharmaceutical studies.^1^ To further demonstrate the selectivity of our H/D exchange protocol, a 1 : 1 mixture of 4-bromobenzaldehyde and 4-bromoanisole was treated under the optimal reaction conditions with 4 h light irradiation, achieving a deuteration selectivity >10 : 1, highlighting the excellent reactivity of formyl C–H bonds in the direct HAT photocatalysis (Table 3b).

**Table 3:** Selective sequential deuteration between formyl C–H and electron-rich C(sp^3^)–H bonds^a^

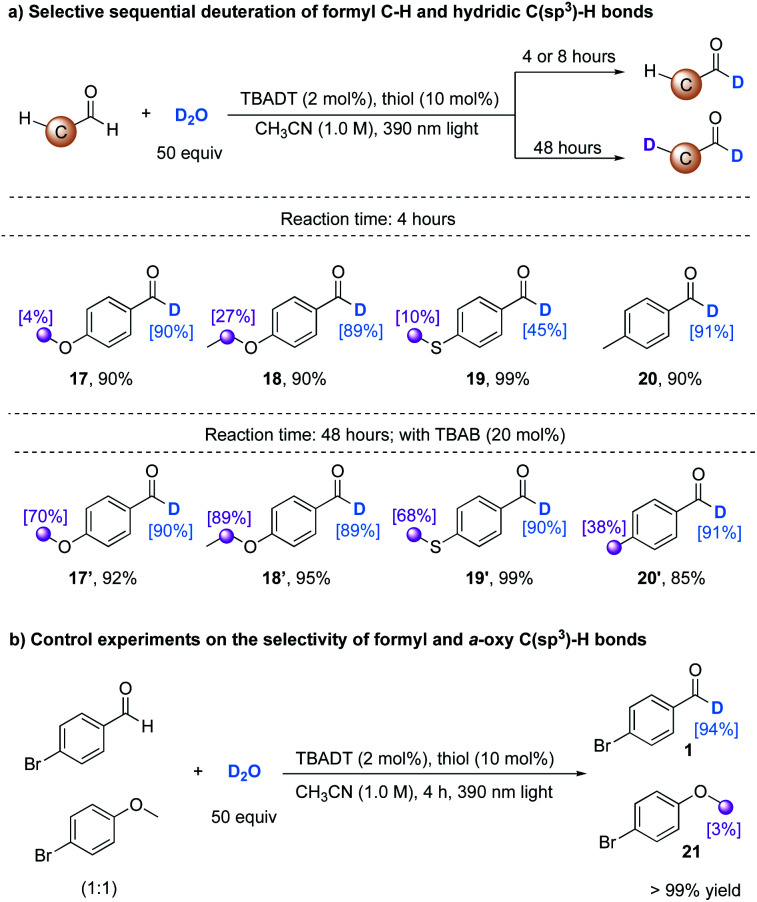

a Reactions were conducted under the optimized conditions. TBAB (20 mol%) was added when the reaction time was 48 h. Isolated yields are given. The deuterium incorporation was determined by the analysis of the ^1^H NMR spectra of products.

With an extended reaction time (24–48 h), the TBADT/thiol catalyzed H/D exchange also proceeds with various hydridic C(sp^3^)–H bonds. Both the polarity effect and steric effect play an important role in determining the regioselectivity in TBADT-catalyzed C–H functionalizations.^23*c*^ As illustrated in Table 4, the tertiary C(sp^3^)–H bonds in alkanes bearing remote electron-withdrawing functional groups such as esters, amides, and ketones can be selectively deuterated (**22–31**). Both cyclic and acyclic alkanes afforded the corresponding products with moderate to good deuterium incorporation. When a substrate bearing both activated C(sp^3^)–H bonds and a tertiary C–H bond was used (**25**, **28**, **30**, and **31**), both C–H bonds underwent H/D exchange to some extent. Addition of 20 mol% TBAB was crucial for an efficient H/D exchange, as significantly lower deuterium incorporation was observed in the absence of TBAB (*e.g.* 36% deuterium incorporation for **22** without TBAB). The benzylic (**32** and **33**), α-ether (**21** and **34–36**) and α-amino (**37** and **38**) C(sp^3^)–H bonds were all feasible substrates for this H/D exchange protocol. Trifluoromethylbenzene can be used as a cosolvent to improve the deuterium incorporation (*e.g.***36**, from 37% to 69%) when the substrate is poorly soluble in acetonitrile.

**Table 4:** TBADT/thiol catalyzed deuterium labelling of representative hydridic C(sp^3^)–H bonds^a^

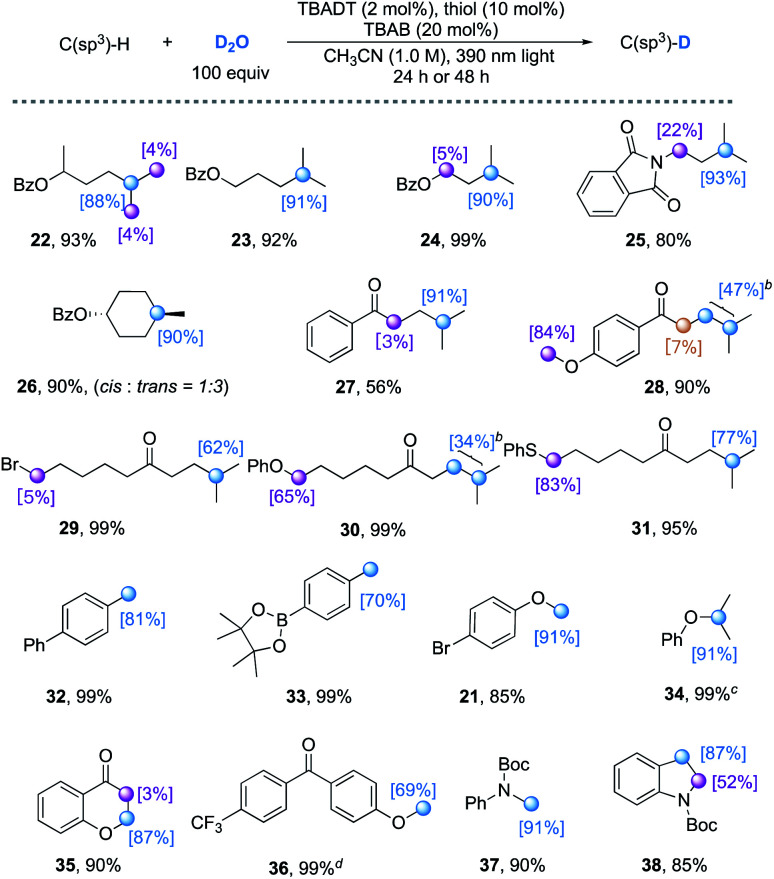

a Unless otherwise noted, reactions were conducted under optimized conditions with the addition of 20 mol% TBAB, irradiated by 390 nm light for 24 or 48 h. Isolated yields are given. The deuterium incorporation was determined by the analysis of the ^1^H NMR spectra of products.

b The overall deuterium incorporation ratio of three C(sp^3^)–H bonds is given.

c Due to the volatility of the substrate, the yield was determined by the analysis of the crude ^1^H NMR spectra using CH_2_Br_2_ as an internal standard.

d C_6_H_5_CF_3_ (1.0 M) was added to solubilize the starting substrate.

The promising functional group compatibility and the wide scope of hydridic C(sp^3^)–H bonds suitable for this dual catalytic method promoted an evaluation of the deuteration of a library of commercially available pharmaceuticals (Table 5), especially those with benzylic C–H bonds and methoxy moieties that are prone to metabolic oxidation.^26^ While isotopic exchange was preferred at the benzylic C–H bond of the acid form of ammonaps (**39**), decarboxylation was observed with ibuprofen and gemfibrozil, which possess a secondary and a tertiary acid moiety respectively. This is probably due to the TBADT promoted photoredox side reactions,^27^ and secondary and tertiary acids are more labile than the primary acid due to the generation of more stable carbon radicals. After methylation to avoid the undesired decarboxylation, the H/D exchange occurred at the tertiary and benzylic C–H bonds of ibuprofen methylester **40**, as well as at the benzylic and α-oxy C–H bonds of gemfibrozil methylester **41**. Heterocyclic drugs, such as benzoyl protected fomepizole (**42**), edaravone (**43**) and pirfenidone (**44**), were deuterated selectively at the benzylic C–H bonds. Notably, the dual catalytic system is not efficient with basic substrates probably due to the acidic nature of TBADT. To overcome this limitation, the free N–H was protected (**42**) or trifluoroacetic acid (TFA) was added (**44**) to achieve a smooth H/D exchange. Butylphthalide (**45**), TFA acidified lidocaine (**46**), benzoyl protected mexiletine (**47**), and acetyl protected chloroxylenol (**48**) could be converted to isotopically enriched products by deuterium incorporation which occurred mainly at the benzylic C–H bonds. Moreover, acetyl protected sesamol, a natural product and a precursor of paroxetine, could be deuterated selectively at the acetal moiety (**49**). (+)-Griseofulvin, an antifungal drug, was converted to the deuterated product **50** with moderate deuterium incorporation at the phenyl methoxy groups. Adamantane derivatives are an important family of drug molecules.^28^ For instance, amantadine is an antiviral drug effectively against various strains of influenza and is also used to treat Parkinson's disease, memantine is used to treat Alzheimer's disease, and 1-adamantanecarboxylic acid is a CerK inhibitor. The *N*-benzoyl protected amantadine (**51**) and memantine (**52**), and phenyl adamantane carboxylate (**53**) could be deuterated with more than 5 deuterium atoms per molecule and with less than 0.1% of unlabelled compound remaining, meeting the requirement for their usage as internal standards in ADME-Tox biostudies.

**Table 5:** Deuterium labelling of pharmaceutical compounds^a^

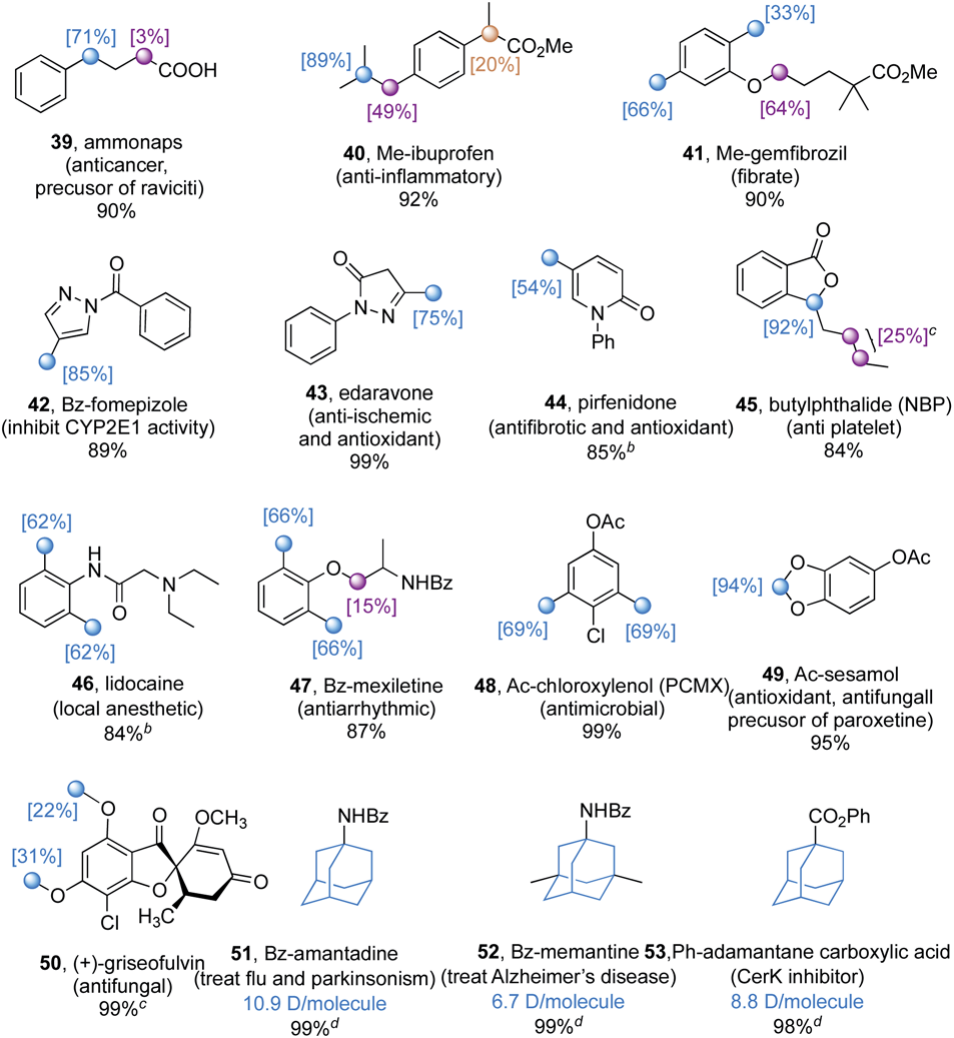

a Unless otherwise noted, reactions were conducted under optimized conditions with the addition of 20 mol% TBAB, irradiated by 390 nm light for 24 or 48 h. Isolated yields are given. The deuterium incorporation was determined by the analysis of the ^1^H NMR spectra of products.

b 3.0 equiv TFA was added.

c The average deuterium incorporation was given.

d C_6_H_5_CF_3_ (1.0 M) was added.

In addition to the late stage deuteration of pharmaceutical compounds, deuterium labelling of drug precursors for *de novo* synthesis also represents an important pathway to introduce deuterium atoms selectively into complex drug molecules. We first attempted to synthesize the precursor for the deuterated drug Austedo (deutetrabenazine, Table 6). However, the level of deuteration of the phenyl methoxy groups in compound **54** was low (33%), while there was full deuteration at the benzylic C–H bonds even though they are relatively electron deficient. Indanone (**55**), the precursor of rasagiline mesylate, which is used to treat Parkinson's disease, could be deuterated at the benzylic position (73%), with the α-carbonyl C–H bonds being deuterated to some extent (12%). Both the phenyl methoxy and benzylic C(sp^3^)–H bonds in the precursor of dextromethorphan underwent H/D exchange effectively (**56**). 2-Methylbenzophenone (**57**), 2-bromo-6-methoxynaphthalene (**58**), and *N*-(2,6-dimethylphenyl)acetamide (**59**) proceeded the H/D exchange smoothly with moderate to excellent deuterium incorporation (62–93%) at the activated hydridic C(sp^3^)–H bonds.

**Table 6:** Deuterium labelling of drug precursors^a^

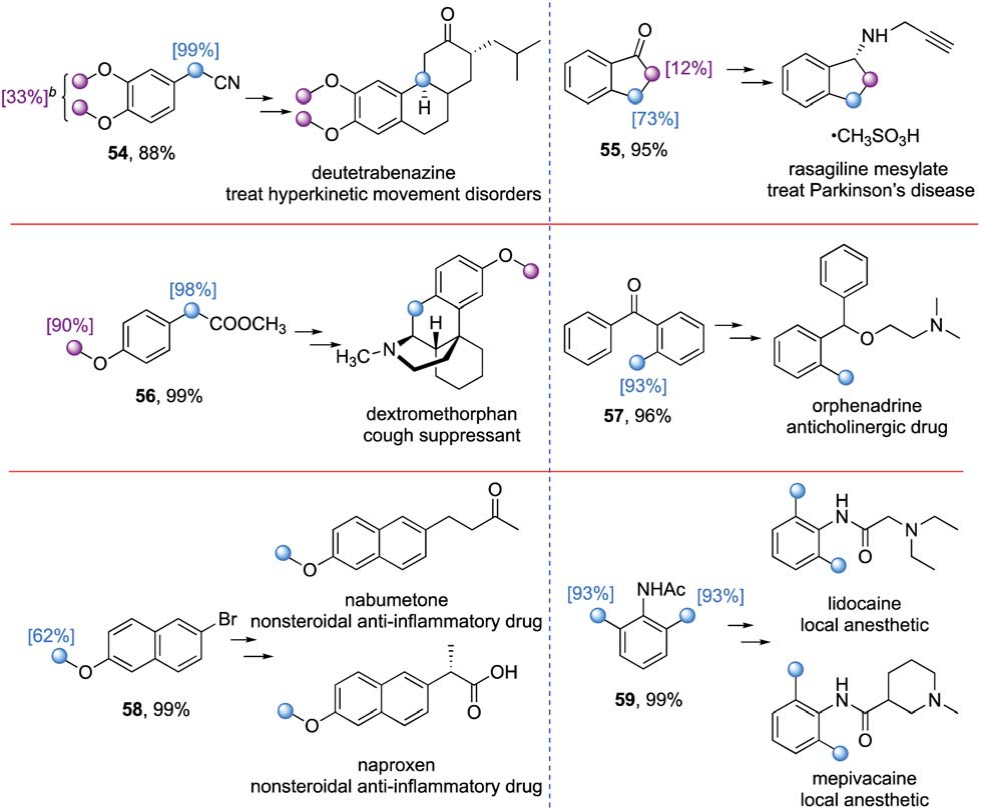

a Unless otherwise noted, reactions were conducted under optimized conditions with the addition of 20 mol% TBAB, irradiated by 390 nm light for 48 h. Isolated yields are given. The deuteration incorporation was determined by the analysis of ^1^H NMR spectra of products.

b The average deuterium incorporation was given.

The method is scalable (Table 7). After the treatment of 4-methoxybenzaldehyde under the optimized conditions for 4 h using a lower catalyst loading (1 mol% TBADT and 5 mol% thiol), a gram-scale synthesis of deuterium labeled **16** could be achieved with an even higher (93%) deuterium level. The selective sequential deuteration of 4-anisaldehyde was also successful, producing both the formyl C–H and α-oxy C(sp^3^)–H labeled product **16′**. Further gram-scale synthesis of the deuterated acid form of ammonaps **39** and an orphenadrine precursor **57** afforded results comparable to those obtained in the synthesis on a 0.2 mmol scale.

**Table 7:** Gram-scale deuteration^a^

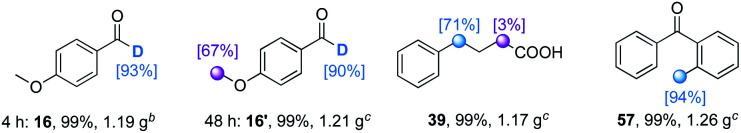

a Unless otherwise noted, the reaction was conducted on a gram-scale under the optimized conditions. Isolated yields are given. The deuterium incorporation was determined by the analysis of ^1^H NMR spectra of products.

b Reaction was conducted for 4 h by using TBADT (1 mol%) and thiol (5 mol%) in CH_3_CN (2.0 M).

c 20 mol% TBAB was added.

Compared to photoredox catalysis,^19^ the use of TBADT as a direct HAT photocatalyst exhibits a wider substrate scope without the limitation of required redox matching. Aldehydic and a variety of hydridic C(sp^3^)–H bonds (*e.g.* α-oxy, α-thioxy, α-amino, benzylic, and unactivated tertiary C(sp^3^)–H bonds) underwent efficient H/D exchange through the synergistic merging of TBADT and a thiol catalyst. The regioselectivity thus is orthogonal and complementary to existing pH-dependent H/D exchange (acidic protons) and transition-metal catalysis (mainly C(sp^2^)–H bonds). We highlighted several H/D exchange products obtained under our conditions that exhibited orthogonal regioselectivity to those obtained by reported transition-metal catalysis (Table 8).^15*c*,29^

**Table 8:** Orthogonal selectivity obtained for our protocol and reported organometallic methodologies in H/D exchange

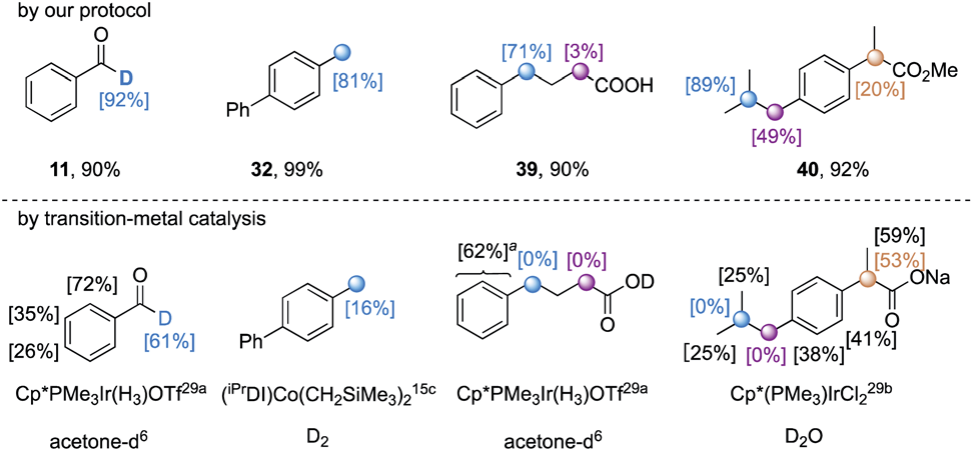

a Average deuterium incorporation was given.

## Mechanism studies

**Scheme 2:**
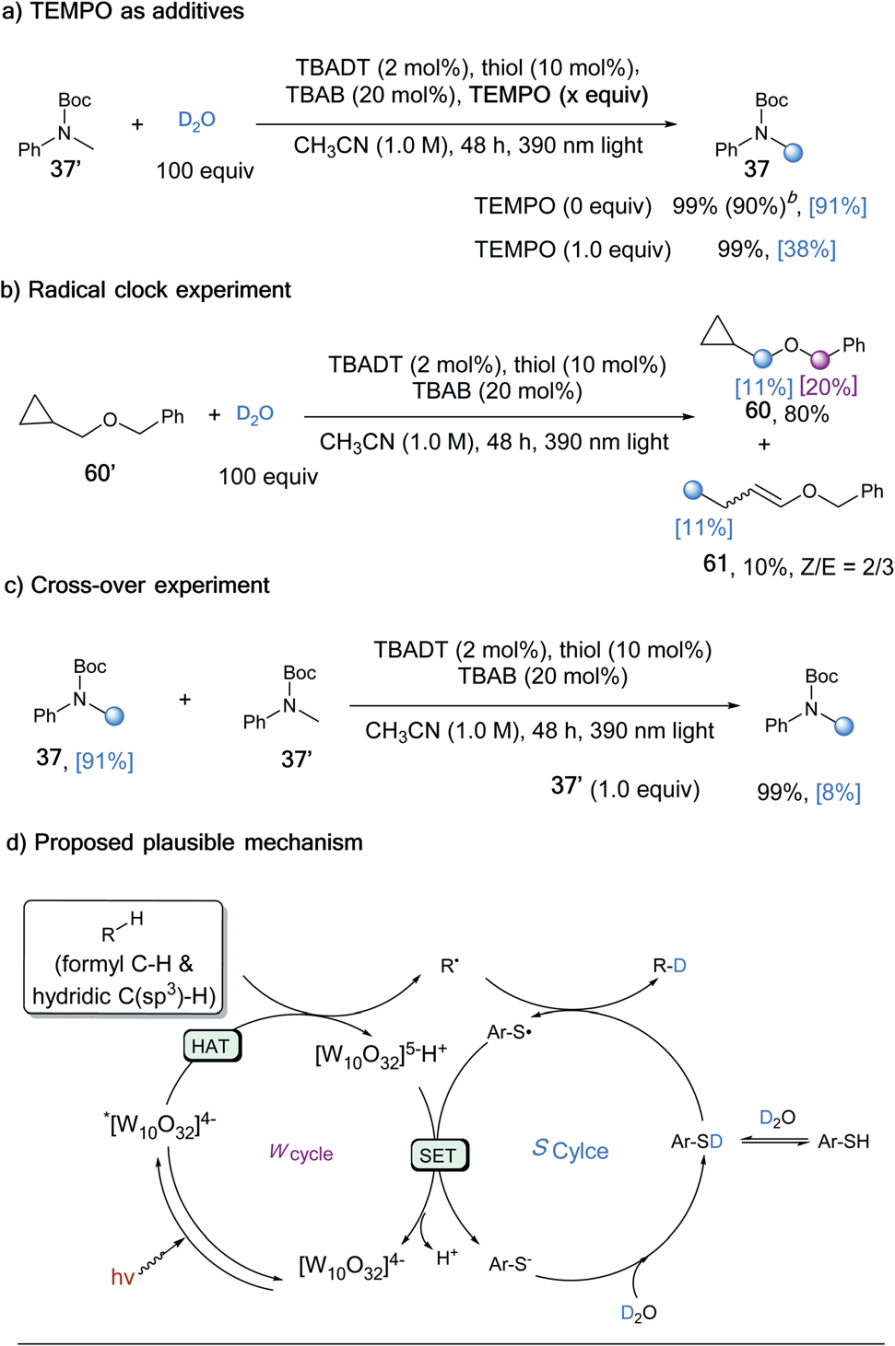
Control experiments and proposed mechanism^*a*^. ^*a*^ Unless otherwise noted, the reaction was conducted under the optimized conditions. Yields were determined by the analysis of ^1^H NMR spectra of the crude product mixture using CH_2_Br_2_ as an internal standard. The deuterium incorporation was determined by the analysis of ^1^H NMR spectra of products. ^*b*^ Yield in parentheses was the isolated yield.

A series of control experiments were conducted to further elucidate the reaction mechanism. The deuterium incorporation was significantly lower in the presence of TEMPO (38% *vs.* 91%, Scheme 2a), which supported the radical process. When radical clock substrate **60′** was applied to our standard reaction conditions, the ring opening product **61** was generated, even though with a low deuterium ratio (Scheme 2b). To further probe the reaction process, deuterated **37** and un-deuterated **37′** were subjected to the reaction conditions in the absence of D_2_O, which resulted in a product with only 8% deuterium incorporation (Scheme 2c). This control experiment indicated that the deuterated product would also undergo H/D exchange, with the generated carbon radical intermediate capturing hydrogen atoms more favourably than deuterium atoms.

A plausible mechanism was proposed in light of all the experimental data and precedent related literature (Scheme 2d).^23^ The transient carbon radical intermediate was generated through a HAT process between the aldehyde/alkane substrate and photo-excited *TBADT. In the presence of excess amounts of D_2_O, the H/D exchange of the thiol catalyst leads to dominant formation of deuterated thiol.^18^ The carbon radical abstracts a deuterium atom from the deuterated thiol to deliver the desired deuterated product and a thiyl radical. A single electron transfer between the thiyl radical and [W_10_O_32_]^5−^H^+^ would regenerate the TBADT catalyst and deuterated thiol after deuteration with D_2_O.^23*a*,30^

## Conclusions

A synergistic catalytic system comprised of the direct HAT photocatalyst TBADT and a thiol catalyst was developed for H/D exchange with formyl C–H and hydridic C(sp^3^)–H bonds, which is orthogonal and complementary to existing pH-dependent H/D exchange and transition-metal catalyzed H/D exchange. A wide range of C–H bonds underwent the H/D exchange effectively, including the formyl C–H, α-oxy, α-thioxy, α-amino, benzylic, and unactivated tertiary C(sp^3^)–H bonds, as a result of the absence of required redox matching between the direct HAT photocatalyst and the substrate. The developed protocol is easily scalable. The ability to introduce deuterium atoms into unique positions of pharmaceutical molecules and drug precursors provides a new diagnostic tool for ADME-Tox studies attempting to develop new molecular therapies. Current efforts are devoted to applying this method to more pharmaceutical candidates and their bioactivity evaluation.

## Conflicts of interest

There are no conflicts to declare.

## Supplementary Material

SC-011-D0SC02661A-s001
