# Supplementary material for: Visible light driven deuteration of formyl C–H and hydridic C(sp3)–H bonds in feedstock chemicals and pharmaceutical molecules
Source: Chem Sci. 2020 Aug 5;11(33):8912–8. doi: 10.1039/d0sc02661a (PMC8163369; doi:10.1039/d0sc02661a)
Supplement: SC-011-D0SC02661A-s001 [file SC-011-D0SC02661A-s001.pdf]

Electronic Supplementary Material (ESI) for Chemical Science.
This journal is © The Royal Society of Chemistry 2020

Visible Light Driven Deuteration of Formyl C-H and Hydridic $C(sp^3)$-H Bonds in Feedstock Chemicals and Pharmaceutical Molecules

Yulong Kuang, Hui Cao, Haidi Tang, Junhong Chew, Wei Chen, Xiangcheng Shi, Jie Wu*

Department of Chemistry, National University of Singapore, 3 Science Drive 3, Singapore, 117543, Singapore

E-Mail: chmjie@nus.edu.sg

Table of Contents

1. General considerations 1
2. Investigation for optimal conditions 2
1) Time course of selective sequential deuteration of 4-anisaldehyde 2
2) Investigation of phase transfer agents 2
3. General procedure 3
4. Results of deuteration of other hydridic C/X-H bonds 3
5. Radical clock expriment 4
6. Characterization of new compounds 4
7. Reference 27
8. Copies of NMR spectra 28

1. General considerations

All catalytic reactions were carried out with commercially available reagents in Schlenk tube (10 mL) under an argon atmosphere with magnetic stirring after freeze-pump-thaw. ^{1}H NMR yield was detected by adding CH_2Br_2 as an internal standard. The isolated yield was the purified product by flash chromatography over silica gel. ^{1}H NMR spectra were recorded on commercial instruments (500 MHz). Chemical shifts were reported in ppm from the solvent resonance as the internal standard ($CDCl_3$, δ = 7.26). Spectra were reported as follows: chemical shift (δ ppm), multiplicity (s = singlet, d = doublet, t = triplet, q = quartet, hept = heptet, m = multiplet), coupling constants (Hz), integration and assignment. ^{13}C NMR spectra were recorded on commercial instruments (126 MHz). Chemical shift was reported in ppm from the solvent resonance as the internal standard ($CDCl_3$, δ = 77). The densely deuterated compounds were also checked by D NMR spectra which were recorded on commercial instruments (77 MHz). Spectra ($CHCl_3$) were reported in ppm from the comparison with its resonance in the corresponding ^{1}H NMR spectra. Grey dots ("●") on the structures represent that the deuterium incorporation is less than 2% based on analysis of ^{1}H NMR spectra, but deuterium signals showed on D NMR spectrum. HRMS was recorded on a commercial apparatus (EI source).

TBADT (tetra-*n*-butylammonium decatungstate)[1] and thiol (2,4,6-triisopropylthiophenol)[2] were synthesized according to the reported literatures.

2. Investigation for optimal conditions

1) Time course of selective sequential deuteration of 4-anisaldehyde

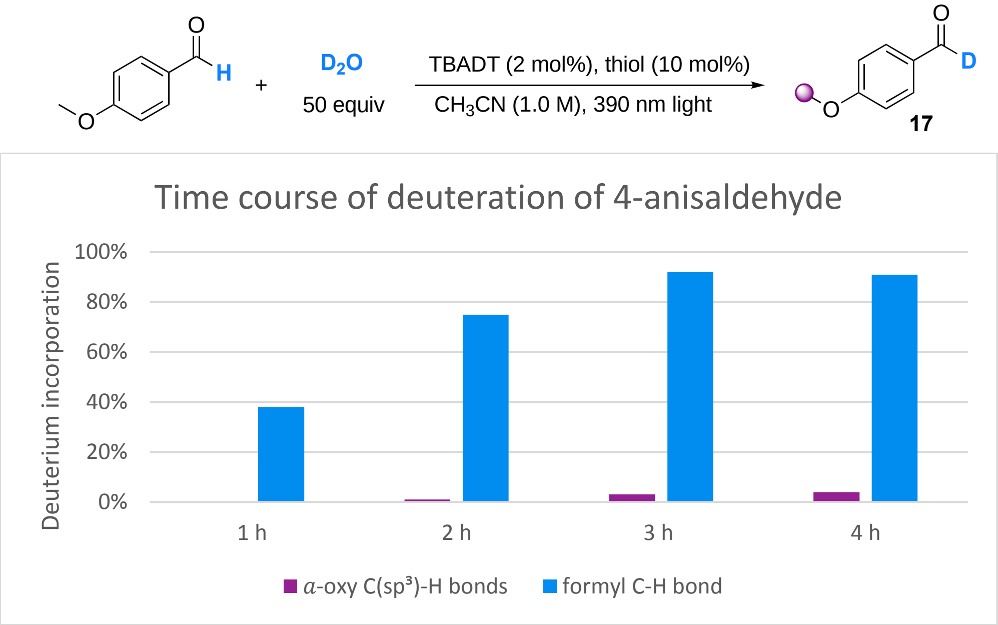

Figure S1. Time course study in selective sequential deuteration of 4-anisaldehyde

2) Investigation of phase transfer agents

Table S1. Investigation of various phase transfer agents[a]

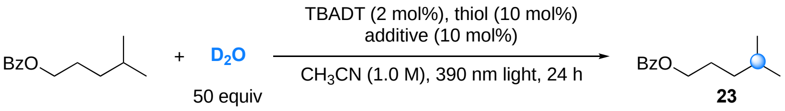

entry	additive	results	
		yield[b]	deuterium incorporation[c]
1	no	96%	84%
2	TBAF•3H_2O	94%	90%
3	TBAB	96%	90%
4[d]	TBAB	99%	91%
5	TBAI	90%	90%
6	$^{n}Bu_4N$•BF_4	92%	89%
7	SDS	93%	27%
8	PPh_4Cl	94%	4%

[a] Reaction was conducted with the indicated additive (10 mol%) for 24 h. [b] Yield was determined by analysis of ^{1}H NMR spectra of the crude product mixture using CH_2Br_2 as an internal standard. [c] Deuterium incorporation was determined by analysis of ^{1}H NMR spectra of products. [d] 20 mol% TBAB was added. TBAF = tetra-*n*-butylammonium fluoride; TBAB = tetra-*n*-butylammonium bromide; TBAI = tetra-*n*-butylammonium iodide; SDS = sodium 1-decanesulfonate.

3. General procedure

General procedure for deuteration of formyl C-H bonds: The aldehyde substrate (0.2 mmol), TBADT (13.2 mg, 2 mol%), thiol (5 uL, 10 mol%), D_2O (180 uL, 50 equiv) and CH_3CN (200 uL, 1.0 M) were added to a 10 mL Schlenk tube equipped with a stir bar. The mixture was operated by freeze-pump-thaw procedures three times before charging the tube with argon. The reactor was then sealed and placed under 390 nm Kessil light (80 W) and stirred for 4 h. Then, the crude reaction mixture was extracted with diethyl ether (3 × 2 mL). The combined organic layer was concentrated and purified by flash column chromatography over silica gel to afford the deuterated product.

General procedure for deuteration of hydridic C(sp^3)-H bonds and pharmaceuticals: A substrate (0.2 mmol), TBADT (13.2 mg, 2 mol%), thiol (5 uL, 10 mol%), D_2O (360 uL, 100 equiv), TBAB (12.8 mg, 20 mol%) and CH_3CN (200 uL, 1.0 M) were added to a Schlenk tube (10 mL) equipped with a stir bar. The mixture was operated by freeze-pump-thaw procedures three times before being charged with argon. The reactor was placed under 390 nm Kessil light (80 W) and kept stirring for 24 or 48 h. The crude reaction mixture was extracted with ethyl acetate (3 × 2 mL). The combined organic layer was concentrated and purified by flash column chromatography over silica gel to afford the deuterated product. $C_6H_5CF_3$ (200 uL, 1.0 M) was added at the beginning in cases where the substrate has low solubility in CH_3CN.

4. Results of deuteration of other hydridic C/X-H bonds

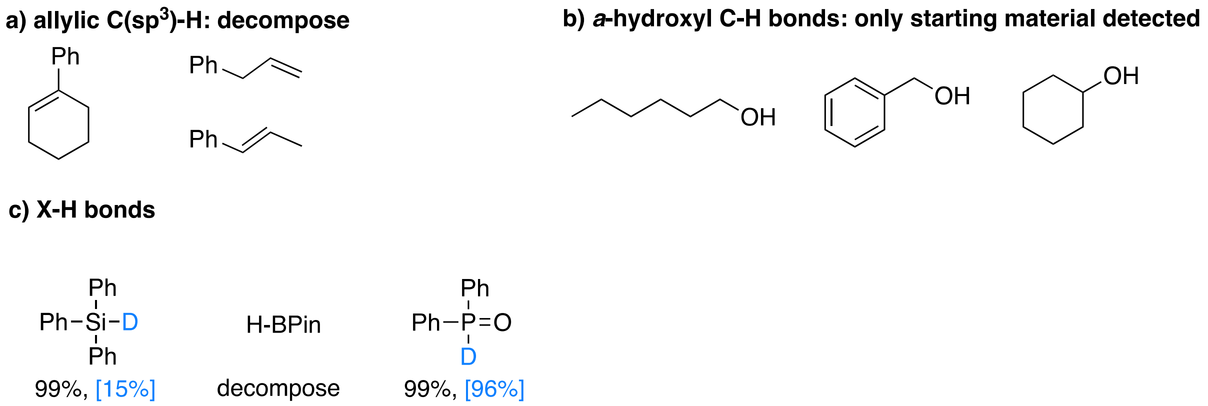

Figure S2. Results on deuteration of other hydridic C/X-H bonds

5. Radical clock experiment

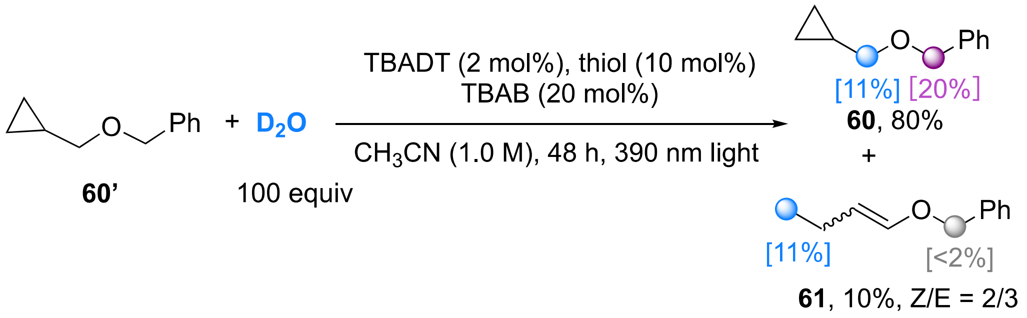

The reaction was conducted under the optimal conditions with 20 mol% TBAB. After 48 h under 390 nm light irritation, the reaction mixture was quenched. The crude NMR showed the above results by comparing with the known spectra.[3] The deuterium incorporation was obtained from ^{1}H NMR spectra of products after purification.

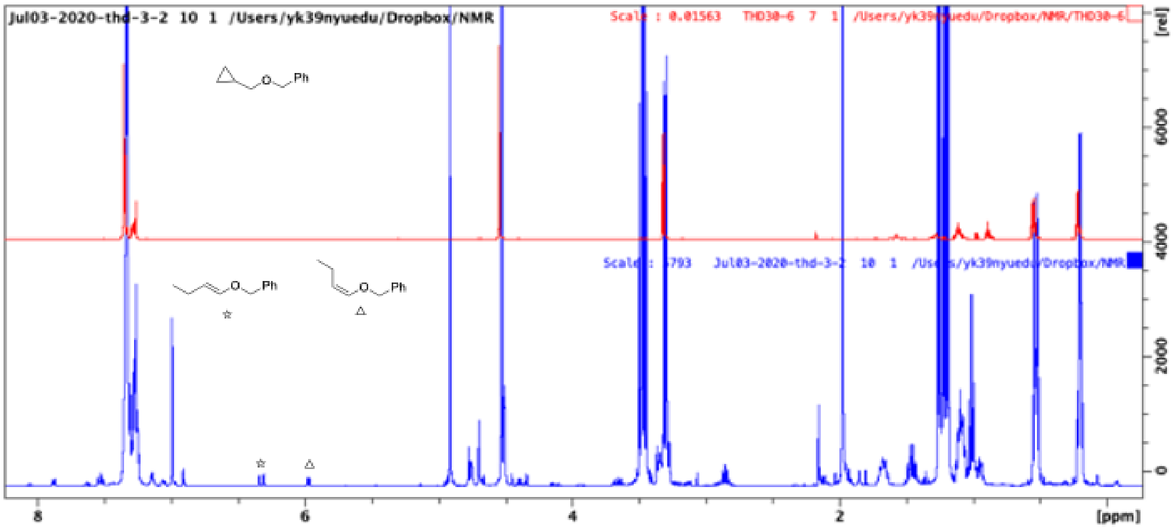

6. Characterization of new compounds

4-Bromobenzaldehyde-formyl-*d1* (**1**):

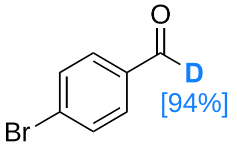

Prepared following the general procedure. ^{1}H NMR spectrum showed 94% deuterium incorporation and quantitative yield (> 99%) with CH_2Br_2 as an internal standard. Then the crude mixture was purified over silica gel chromatography (hexane : diethyl ether = 20 : 1) to afford product as a colorless solid in 31.6 mg (85% yield). ^{1}H NMR (500 MHz, $CDCl_3$) δ = 9.96 (s, 0.06H), 7.73 (d, *J* = 8.0 Hz, 2H), 7.66 (d, *J* = 8.0 Hz, 2H). ^{13}C NMR (126 MHz, $CDCl_3$) δ = 191.0 (COH), 190.7 (t, *J* = 27.2 Hz, COD), 134.9, 132.3, 130.9, 129.7. HRMS (EI-TOF) calcd for C_7H_4DBrO (M^+) = 189.9561, found 186.9562.

4-Chlorobenzaldehyde-formyl-*d1* (**2**):

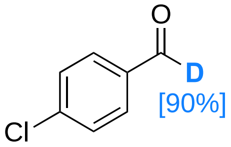

Prepared following the general procedure. ^{1}H NMR spectrum showed 90% deuterium incorporation and quantitative yield (> 99%) with CH_2Br_2 as an internal standard. Then the crude mixture was purified over silica gel chromatography (hexane : diethyl ether = 20 : 1) to afford product as a colorless oil in 24.8 mg (88% yield). ^{1}H NMR (500 MHz, $CDCl_3$) δ = 9.98 (s, 0.10H), 7.82 (d, *J* = 8.5 Hz, 2H), 7.51 (d, *J* = 8.5 Hz, 2H). ^{13}C NMR (126 MHz, $CDCl_3$) δ = 190.8 (COH), 190.5 (t, *J* = 26.8 Hz, COD), 140.9, 134.6 (t, *J* = 3.8 Hz), 130.9, 129.4. HRMS (EI-TOF) calcd for C_7H_4DClO (M^+) = 141.0086, found 141.0081.

4-Fluorobenzaldehyde-formyl-*d1* (**3**):

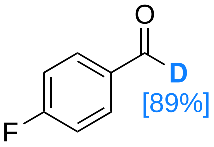

Prepared following the general procedure. ^{1}H NMR spectrum showed 89% deuterium incorporation and quantitative yield (> 99%) using CH_2Br_2 as an internal standard. Then the crude mixture was purified over silica gel chromatography (hexane : diethyl ether = 20 : 1) to afford product as a colorless oil in 22.2 mg (86% yield). ^{1}H NMR (500 MHz, $CDCl_3$) δ = 9.96 (s, 0.11H), 7.96 – 7.86 (m, 2H), 7.24 – 7.17 (m, 2H). ^{13}C NMR (126 MHz, $CDCl_3$) δ = 190.5 (COH), 190.2 (t, *J* = 26.6 Hz, COD), 166.5 (d, *J* = 257.4 Hz), 133.0 – 132.5 (m), 132.2 (d, *J* = 9.6 Hz), 116.3 (d, *J* = 22.6 Hz). HRMS (EI-TOF) calcd for C_7H_4DFO (M^+) = 125.0382, found 125.0384.

4-Acetoxybenzaldehyde-formyl-*d1* (**4**):

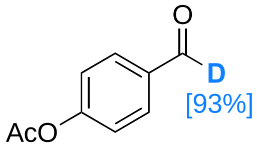

Prepared following the general procedure by using 365 nm (24 W) LED strip instead of 390 nm (80 W) Kessil light for 24 h. ^{1}H NMR spectrum showed 93% deuterium incorporation and 99% yield using CH_2Br_2 as an internal standard. Then the crude mixture was purified over silica gel chromatography (hexane : diethyl ether = 20 : 1) to afford product as a colorless oil in 31.7 mg (96% yield). ^{1}H NMR (500 MHz, $CDCl_3$) δ = 9.99 (s, 0.07H), 7.92 (d, *J* = 8.5 Hz, 2H), 7.27 (d, *J* = 8.5 Hz, 2H), 2.33 (s, 3H). ^{13}C NMR (126 MHz, $CDCl_3$) δ = 190.9 (COH), 190.6 (t, *J* = 26.6 Hz, COD), 168.6, 153.3, 133.9 (t, *J* = 3.5 Hz), 131.1, 122.3, 21.1. HRMS (EI-TOF) calcd for $C_9H_7DO_3$ (M^+) = 165.0531, found 165.0532.

4-Acetamidobenzaldehyde-formyl-*d1* (**5**):

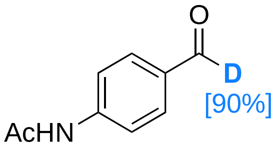

Prepared following the general procedure. ^{1}H NMR spectrum showed 90% deuterium incorporation and 95% yield using CH_2Br_2 as an internal standard. Then the crude mixture was purified over silica gel chromatography (hexane : diethyl ether = 20 : 1) to afford product as a colorless solid in 29.5 mg (90% yield). ^{1}H NMR (500 MHz, CD_3CN) δ = 9.87 (s, 0.10H), 8.71 (br, 1H), 7.88 – 7.79 (m, 2H), 7.78 – 7.68 (m, 2H), 2.10 (s, 3H). ^{13}C NMR (126 MHz, CD_3CN) δ = 192.3 (COH), 192.0 (t, J = 26.8 Hz, COD), 170.3, 145.7, 132.9, 131.8, 119.8, 24.7. HRMS (EI-TOF) calcd for $C_9H_8DNO_2$ (M^+) = 164.0691, found 164.0690.

4-Hydroxybenzaldehyde-formyl-*d1* (**6**):

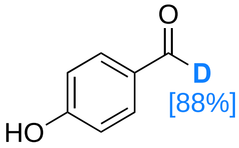

Prepared following the general procedure. ^{1}H NMR spectrum showed 88% deuterium incorporation and 95% yield using CH_2Br_2 as an internal standard. Then the crude mixture was purified over silica gel chromatography (hexane : diethyl ether = 5 : 1) to afford product as a colorless solid in 21.4 mg (87% yield). ^{1}H NMR (500 MHz, CD_3CN) δ = 9.82 (s, 0.12H), 7.77 (d, J = 8.5 Hz, 2H), 6.96 (d, J = 8.5 Hz, 2H). ^{13}C NMR (126 MHz, CD_3CN) δ = 191.9 (COH), 191.2 (t, J = 28.1 Hz, COD), 163.7, 133.1, 130.6 (t, J = 3.7 Hz), 116.9. HRMS (EI-TOF) calcd for $C_7H_5DO_2$ (M^+) = 123.0425, found 123.0423.

4-(Trifluoromethyl)benzaldehyde-formyl-*d1* (**7**):

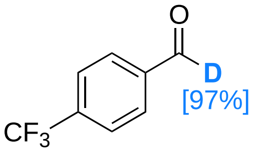

Prepared following the general procedure. ^{1}H NMR spectrum showed 97% deuterium incorporation and 80% yield using CH_2Br_2 as an internal standard. Then the compound was purified over silica gel chromatography (hexane : diethyl ether = 20 : 1) to afford product as a colorless oil in 26.2 mg (70% yield). ^{1}H NMR (500 MHz, $CDCl_3$) δ = 10.10 (s, 0.03H), 8.01 (d, J = 8.0 Hz, 2H), 7.80 (d, J = 8.0 Hz, 2H). ^{13}C NMR (126 MHz, $CDCl_3$) δ = 190.7 (t, J = 26.8 Hz, COD), 138.5, 135.6 (q, J = 32.8 Hz), 129.9, 126.8 (q, J = 3.8 Hz), 123.4 (q, J = 273.5 Hz). HRMS (EI-TOF) calcd for $C_8H_4DF_3O$ (M^+) = 175.0350, found 175.0346.

4-Cyanobenzaldehyde-formyl-*d1* (**8**):

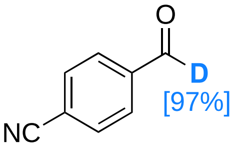

Prepared following the general procedure. ^{1}H NMR spectrum showed 97% deuterium incorporation and 75% yield using CH_2Br_2 as an internal standard. Then the crude mixture was purified over silica gel

chromatography (hexane : diethyl ether = 20 : 1) to afford product as a colorless oil in 18.5 mg (70% yield). ^{1}H NMR (500 MHz, $CDCl_3$) δ = 10.09 (s, 0.03H), 8.00 (d, *J* = 8.5 Hz, 2H), 7.85 (d, *J* = 8.5 Hz, 2H). ^{13}C NMR (126 MHz, $CDCl_3$) δ = 190.2 (t, *J* = 27.5 Hz, COD), 138.6 (t, *J* = 3.7 Hz), 132.9, 129.8, 117.7, 117.6. HRMS (EI-TOF) calcd for C_8H_4DNO (M^+) = 132.0428, found 132.0429.

4-(4,4,5,5-Tetramethyl-1,3,2-dioxaborolan-2-yl)benzaldehyde-formyl-*d1* (**9**):

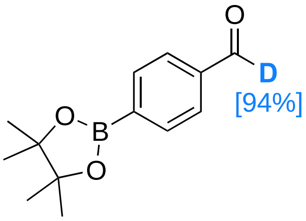

Prepared following the general procedure. ^{1}H NMR spectrum showed 94% deuterium incorporation and quantitative yield (> 99%) with CH_2Br_2 as an internal standard. Then the compound was purified over silica gel chromatography (hexane : diethyl ether = 2 : 1) to afford product as a colorless solid in 34.9 mg (75% yield). ^{1}H NMR (500 MHz, $CDCl_3$) δ = 10.04 (s, 0.06H), 7.96 (d, *J* = 8.0 Hz, 2H), 7.86 (d, *J* = 8.0 Hz, 2H), 1.36 (s, 9H). ^{13}C NMR (126 MHz, $CDCl_3$) δ = 192.6 (COH), 192.3 (t, *J* = 26.7 Hz, COD), 138.0 (t, *J* = 3.2 Hz), 135.2, 128.7, 84.3, 24.9. HRMS (EI-TOF) calcd for $C_{13}H_{16}DBO_3$ (M^+) = 233.1328, found 233.1323.

4-Benzyloxybenzaldehyde-formyl-*d1*(**10**):

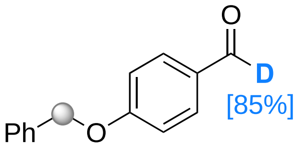

Prepared following the general procedure for 8 h. ^{1}H NMR spectrum showed 85% deuterium incorporation on the formyl C-H bond, less than 2% deuterium incorporation on the *a*-oxy C(sp^3)-H bonds and 99% yield using CH_2Br_2 as an internal standard. Then crude mixture was purified over silica gel chromatography (hexane : diethyl ether = 20 : 1) to afford product as a colorless solid in 36.7 mg (86% yield). ^{1}H NMR (500 MHz, $CDCl_3$) δ = 9.89 (s, 0.15H), 7.86 – 7.82 (m, 2H), 7.46 – 7.37(m, 4H), 7.37 – 7.31 (m, 1H), 7.08 (d, *J* = 8.5 Hz, 2H), 5.15 (s, 2H). ^{13}C NMR (126 MHz, $CDCl_3$) δ = 190.7 (COH), 190.4 (t, *J* = 26.3 Hz, COD), 163.7, 135.9, 131.9, 130.0, 128.7, 128.3, 127.4, 115.1, 70.2. HRMS (EI-TOF) calcd for $C_{14}H_{11}DO_2$ (M^+) = 213.0900, found 213.0895.

Benzaldehyde-formyl-*d1* (**11**):

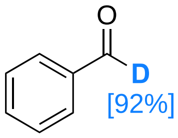

Prepared following the general procedure. ^{1}H NMR spectrum showed 91% deuterium incorporation and quantitative yield (> 99%) with CH_2Br_2 as an internal standard. Then the crude mixture was purified over silica gel

chromatography (hexane : diethyl ether = 20 : 1) to afford product as a colorless oil in 19.2 mg (90% yield). ^{1}H NMR (500 MHz, $CDCl_3$) δ = 10.0 (s, 0.08H), 7.92 – 7.81 (m, 2H), 7.63 (t, J = 7.5 Hz, 1H), 7.57 – 7.47 (m, 2H). ^{13}C NMR (126 MHz, $CDCl_3$) δ = 192.3 (CHO), 192.0 (t, J = 26.7 Hz, COD), 136.3, 134.4, 129.7, 129.0. HRMS (EI-TOF) calcd for C_7H_5DO (M^+) = 107.0481, found 107.0480.

Thiophen-2-carbaldehyde-formyl-*d1* (**12**):

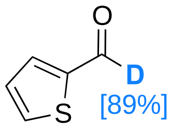

Prepared following the general procedure. ^{1}H NMR spectrum showed 89% deuterium incorporation and quantitative yield (> 99%) with CH_2Br_2 as an internal standard. Then the compound was purified over silica gel chromatography (hexane : diethyl ether = 20 : 1) to afford product as a colorless oil in 18.8 mg (83% yield). ^{1}H NMR (500 MHz, $CDCl_3$) δ = 9.94 (s, 0.11H), 7.82 – 7.70 (m, 2H), 7.21 (t, J = 4.0 Hz, 1H). ^{13}C NMR (126 MHz, $CDCl_3$) δ = 182.9 (COH), 182.7 (t, J = 27.3 Hz, COD), 144.0 (t, J = 4.7 Hz), 136.2, 135.1, 128.3. HRMS (EI-TOF) calcd for C_5H_3DOS (M^+) = 113.0040, found 113.0042.

3-Phenylpropionaldehyde-formyl-*d1* (**13**):

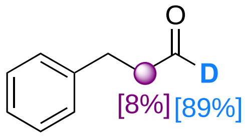

Prepared following the general procedure. ^{1}H NMR spectrum showed 89% deuterium incorporation on formyl group, 8% deuterium incorporation on the *a*-carbonyl C(sp^3)-H bonds and 99% yield using CH_2Br_2 as an internal standard. Then the crude mixture was purified over silica gel chromatography (hexane : diethyl ether = 20 : 1) to afford product as a colorless oil in 22.9 mg (85% yield). ^{1}H NMR (500 MHz, $CDCl_3$) δ = 9.82 (t, J = 1.5 Hz, 0.11H), 7.31 (td, J = 7.5, 1.5 Hz, 2H), 7.22 (t, J = 7.5 Hz, 3H), 2.97 (t, J = 7.5 Hz, 2H), 2.82 – 2.74 (m, 1.84H). ^{13}C NMR (126 MHz, $CDCl_3$) δ = 201. 5 (COH), 201.2 (t, J = 26.5 Hz, COD), 140.3, 128.5, 128.2, 126.2, 45.0 (t, J = 3.7 Hz), 28.0. D NMR (77 MHz, $CHCl_3$) δ = 9.82. HRMS (EI-TOF) calcd for C_9H_9DO (M^+) = 135.0789, found 135.0788.

p-*tert*-Butyl-α-methylhydrocinnamic aldehyde-formyl-*d1* (**14**):

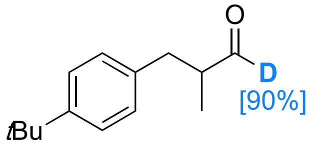

Prepared following the general procedure. ^{1}H NMR spectrum showed 90% deuterium incorporation and 99% NMR yield using CH_2Br_2 as an internal standard. Then the compound was purified over silica gel chromatography (hexane : diethyl ether = 20 : 1) to afford product as a colorless oil in 31.6 mg (77% yield). ^{1}H NMR (500 MHz, $CDCl_3$) δ = 9.72 (m, 0.10H), 7.32 (d, J = 8.5 Hz, 2H), 7.11 (d, J = 8.5 Hz, 2H), 3.06 (dd, J = 13.5, 6.0 Hz, 1H), 2.72 – 2.62 (m, 1H), 2.59

(dd, J = 13.5, 8.0 Hz, 1H), 1.32 (s, 9H), 1.10 (d, J = 7.0 Hz, 3H). ^{13}C NMR (126 MHz, $CDCl_3$) δ = 204.6 (COH), 204.3 (t, J = 26.3 Hz, COD), 149.2, 135.7, 128.6, 125.4, 48.0 - 47.8 (m), 36.1, 34.4, 31.3, 13.2. D NMR (77 MHz, $CHCl_3$) δ = 9.73. HRMS (EI-TOF) calcd for $C_{14}H_{19}DO$ (M^+) = 205.1571, found 205.1570.

6-Oxo-6-phenylhexanal-formyl-*d1* (**15**):

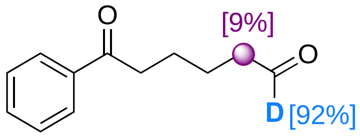

Prepared following the general procedure. ^{1}H NMR spectrum showed 92% deuterium incorporation on formyl group, 9% deuterium incorporation on the *a*-carbonyl C(sp^3)-H bonds and quantitative yield (> 99%) using CH_2Br_2 as an internal standard. Then the crude mixture was purified over silica gel chromatography (hexane : diethyl ether = 20 : 1) to afford product as a colorless solid in 29.0 mg (76% yield). ^{1}H NMR (500 MHz, $CDCl_3$) δ = 9.78 (s, 0.08H), 8.01 – 7.82 (m, 2H), 7.64 – 7.52 (m, 1H), 7.52 – 7.38 (m, 2H), 3.00 (t, J = 7.0 Hz, 2H), 2.49 (t, J = 7.0 Hz, 1.83H), 1.87 – 1.60 (m, 4H). ^{13}C NMR (126 MHz, $CDCl_3$) δ = 202.2, 201.9 (t, J = 26.5 Hz), 136.8, 133.0, 128.6, 128.0, 43.7 - 43.5 (m), 38.1, 23.6, 21.6. D NMR (77 MHz, $CHCl_3$) δ = 9.78, 2.44. HRMS (EI-TOF) calcd for $C_{12}H_{13}DO_2$ (M^+) = 191.1051, found 191.1058.

n-Hexanal-formyl-*d1* (**16**):

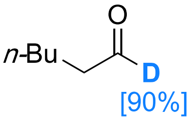

Prepared following the general procedure by using CD_3CN (1.0 M) instead of CH_3CN (1.0 M). ^{1}H NMR spectrum showed 90% deuterium incorporation and 90% yield using CH_2Br_2 as an internal standard. ^{1}H NMR (500 MHz, CD_3CN) δ = 9.64 (m, 0.10H), 2.42 – 2.31 (m, 1H), 2.29 – 2.19 (m, 1H), 1.59 – 1.50 (m, 2H), 1.28 – 1.24 (m, 4H), 0.86 (t, J = 7.0 Hz, 3H). HRMS (EI-TOF) calcd for $C_{12}H_{13}DO_2$ (M^+) = 101.0945, found 101.0950.

4-Methoxybenzaldehyde-formyl-*d1* (**17**):

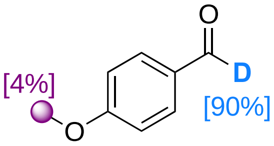

Prepared following the general procedure. ^{1}H NMR showed 90% deuterium incorporation on the formyl C-H bond, 4% deuterium incorporation on the *a*-oxy C(sp^3)-H bonds and 99% yield using CH_2Br_2 as an internal standard. Then the crude mixture was purified over silica gel chromatography (hexane : diethyl ether = 20 : 1) to afford product as a colorless oil in 24.9 mg (90% yield). ^{1}H NMR (500 MHz, $CDCl_3$) δ = 9.87 (s, 0.10H), 7.85 – 7.75 (m, 2H), 6.99 (d, J = 9.0 Hz, 2H), 3.90 – 3.85 (m, 2.87H). ^{13}C NMR (126 MHz, $CDCl_3$) δ = 190.8 (COH), 190.4

(t, J = 26.5 Hz, COD), 164.6, 131.9, 129.8 (t, J = 3.8 Hz), 114.3, 55.5 (OCH_3), 55.2 (t, J = 22.6 Hz, OCH_2D). HRMS (EI-TOF) calcd for $C_8H_7DO_2$ (M^+) = 137.0579, found 137.0574.

4-(1,1,1-*d3*-Methoxy)-benzaldehyde-formyl-*d1* (**17'**):

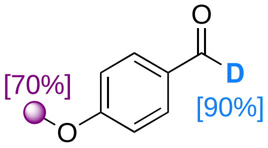

Prepared following the general procedure for 48 h with the addition of TBAB (20 mol%). ^1H NMR spectrum showed 90% deuterium incorporation on the formyl C-H bond, 70% deuterium incorporation on the *a*-oxy C(sp^3)-H bonds and 99% yield using CH_2Br_2 as an internal standard. Then the crude mixture was purified over silica gel chromatography (hexane : diethyl ether = 20 : 1) to afford product as a colorless oil in 26.0 mg (92% yield). ^1H NMR (500 MHz, $CDCl_3$) δ = 9.88 (s, 0.10H), 7.84 (d, J = 8.5 Hz, 2H), 7.01 (d, J = 8.5 Hz, 2H), 3.90 – 3.83 (m, 0.89H). ^{13}C NMR (126 MHz, $CDCl_3$) δ = 190.8 (COH), 190.5 (t, J = 26.5 Hz, COD), 164.6, 132.0, 129.9 (t, J = 3.7 Hz), 114.3, 55.7 – 54.6 (m). HRMS (EI-TOF) calcd for $C_8H_4D_4O_2$ (M^+) = 140.0770, found 140.0763.

4-(1,1-*d2*-Ethoxy)-benzaldehyde-formyl-*d1* (**18**):

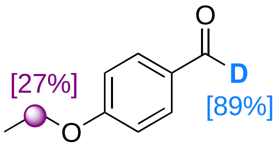

Prepared following the general procedure. ^1H NMR spectrum showed 88% deuterium incorporation on the formyl C-H bond, 27% deuterium incorporation on the *a*-oxy C(sp^3)-H bonds and 94% yield using CH_2Br_2 as an internal standard. Then the crude mixture was purified over silica gel chromatography (hexane : diethyl ether = 20 : 1) to afford product as a colorless oil in 27.3 mg (90% yield). ^1H NMR (500 MHz, $CDCl_3$) δ = 9.85 (s, 0.11H), 7.83 – 7.78 (m, 2H), 7.00 – 6.95 (m, 2H), 4.13 – 4.04 (m, 1.46H), 1.43 (t, J = 8.5 Hz, 3H). ^{13}C NMR (126 MHz, $CDCl_3$) δ = 190.7 (COH), 190.4 (t, J = 26.3 Hz, COD), 164.0, 131.9, 129.6 (t, J = 7.6 Hz), 114.6, 63.8 (OCH_2CH_3), 63.5 (t, J = 22.0 Hz, $OCHDCH_3$), 14.6 – 14.3 (m). HRMS (EI-TOF) calcd for $C_9H_8D_2O_2$ (M^+) = 152.0803, found 152.0799.

4-(1,1-*d2*-Ethoxy)-benzaldehyde-formyl-*d1* (**18'**):

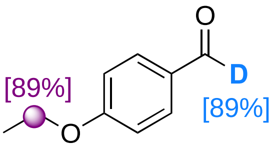

Prepared following the general procedure for 48 h with the addition of TBAB (20 mol%). ^1H NMR spectrum showed 89% deuterium incorporation on the formyl C-H bond, 89% deuterium incorporation on the *a*-oxy C(sp^3)-H bonds and 99% yield using CH_2Br_2 as an internal standard. Then the compound was purified over silica gel chromatography (hexane : diethyl ether = 20 : 1) to afford product as a colorless oil in 28.7 mg (95% yield). ^1H NMR (500 MHz, $CDCl_3$) δ = 9.87

(s, 0.11H), 7.81 (d, J = 8.5 Hz, 2H), 6.97 (d, J = 8.5 Hz, 2H), 4.14 – 4.06 (m, 0.23H), 1.45 – 1.38 (m, 3H). ^{13}C NMR (126 MHz, $CDCl_3$) δ = 190.8 (COH), 190.5 (t, J = 26.3 Hz, COD), 164.0, 131.9, 129.7 (t, J = 7.6 Hz), 114.7, 63.8 – 63.0 (m), 14.5 – 14.3 (m). HRMS (EI-TOF) calcd for $C_9H_7D_3O_2$ (M^+) = 153.0864, found 153.0860.

4-(Methylthio)benzaldehyde-formyl-*d1* (**19**):

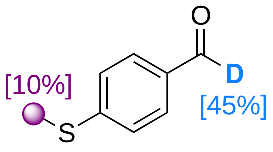

Prepared following the general procedure. ^{1}H NMR spectrum showed 45% deuterium incorporation on the formyl C-H bond, 10% deuterium incorporation on the *a*-thioxy C(sp^3)-H bonds and 99% yield using CH_2Br_2 as an internal standard. Then the crude mixture was purified over silica gel chromatography (hexane : diethyl ether = 20 : 1) to afford product as a colorless oil in 30.4 mg (99% yield). ^{1}H NMR (500 MHz, $CDCl_3$) δ = 9.89 (s, 0.55H), 7.77 – 7.69 (m, 2H), 7.32 – 7.27 (m, 2H), 2.55 – 2.41 (m, 2.71 H). ^{13}C NMR (126 MHz, $CDCl_3$) δ = 191.1 (COH), 190.8 (t, J = 26.3 Hz, COD), 147.8, 132.8, 129.8, 125.0, 14.5. HRMS (EI-TOF) calcd for $C_8H_6D_2SO$ (M^+) = 154.0410, found 154.0406.

4-(1,1,1-*d3*-Methylthio)benzaldehyde-formyl-*d1* (**19'**):

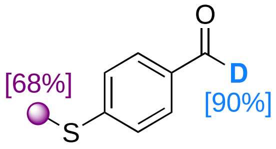

Prepared following the general procedure for 48 h with the addition of TBAB (20 mol%). ^{1}H NMR spectrum showed 90% deuterium incorporation on the formyl C-H bond, 68% deuterium incorporation on the *a*-thioxy C(sp^3)-H bonds and 99% yield using CH_2Br_2 as an internal standard. Then the crude mixture was purified over silica gel chromatography (hexane : diethyl ether = 20 : 1) to afford product as a colorless oil in 30.3 mg (99% yield). ^{1}H NMR (500 MHz, $CDCl_3$) δ = 9.90 (s, 0.10H), 7.75 (d, J = 8.5 Hz, 2H), 7.30 (d, J = 8.5 Hz, 2H), 2.52 – 2.47 (m, 0.95H). ^{13}C NMR (126 MHz, $CDCl_3$) δ = 191.1 (COH), 190.8 (t, J = 26.3 Hz, COD), 147.8, 132.8, 129.9, 125.1, 14.6 – 13.8 (m). HRMS (EI-TOF) calcd for $C_8H_4D_4SO$ (M^+) = 156.0541, found 156.0537.

4-Methylbenzaldehyde-formyl-*d1*(**20**):

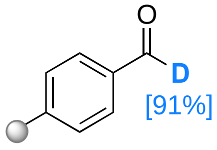

Prepared following the general procedure. ^{1}H NMR spectrum showed 91% deuterium incorporation on the formyl C-H bond, less than 2% deuterium incorporation on the benzyl C(sp^3)-H bond s and 97% yield using CH_2Br_2 as an internal standard. Then the crude mixture was purified over silica gel chromatography (hexane : diethyl ether = 20 : 1) to afford product as a colorless oil in 21.8 mg (90% yield). ^{1}H NMR (500 MHz, $CDCl_3$) δ = 9.96 (s, 0.09H), 7.77 (d, J = 9.5 Hz, 2H), 7.32 (d, J = 9.5 Hz, 2H),

2.43 (s, 3H). ^{13}C NMR (126 MHz, $CDCl_3$) δ = 191.9 (COH), 191.6 (t, *J* = 33.0 Hz, COD), 145.5, 134.1 (t, *J* = 4.3 Hz), 129.8, 129.7, 21.8. HRMS (EI-TOF) calcd for C_8H_7DO (M^+) = 121.0632, found 121.0631.

4-(1,1,1-*d3*-Methyl)benzaldehyde-formyl-*d1* (**20'**):

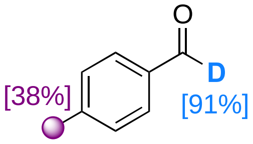

Prepared following the general procedure for 48 h with the addition of TBAB (20 mol%). ^{1}H NMR spectrum showed 91% deuterium incorporation on the formyl C-H bond, 38% deuterium incorporation on the benzyl C(sp^3)-H bonds and 95% yield using CH_2Br_2 as an internal standard. Then compound was purified over silica gel chromatography (hexane : diethyl ether = 20 : 1) to afford product as a colorless oil in 20.6 mg (85% yield). ^{1}H NMR (500 MHz, $CDCl_3$) δ = 9.96 (s, 0.09H), 7.78 (d, *J* = 8.0 Hz, 2H), 7.32 (d, *J* = 8.0 Hz, 2H), 2.43 (s, 1.87H). ^{13}C NMR (126 MHz, $CDCl_3$) δ = 192.0 (COH), 191.7 (t, *J* = 33.0 Hz, COD), 145.6 – 145.4 (m), 134.3 – 134.0 (m), 129.8, 129.7, 21.9 (CH_3), 21.6 (t, *J* = 18.0 Hz, CH_2D). HRMS (EI-TOF) calcd for $C_8H_6D_2O$ (M^+) = 122.0691, found 122.0681.

5-Methylhexan-2-yl-5-*d* benzoate (**22**):

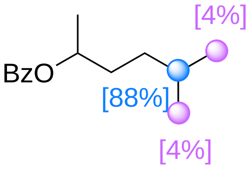

Prepared following the general procedure for 24 h with the addition of TBAB (20 mol%). The crude mixture was purified over silica gel chromatography (hexane : diethyl ether = 20 : 1) to afford product as a colorless oil in 41.0 mg (93% yield). ^{1}H NMR spectrum showed 88% deuterium incorporation on the tertiary C(sp^3)-H bond. ^{1}H NMR (500 MHz, $CDCl_3$) δ = 8.16 – 8.00 (m, 2H), 7.66 – 7.49 (m, 1H), 7.48 – 7.40 (m, 2H), 5.22 – 5.10 (m, 1H), 1.82 – 1.68 (m, 1H), 1.67 – 1.58 (m, 1.12H), 1.34 (d, *J* = 6.5 Hz, 3H), 1.32 – 1.20 (m, 2H), 0.96 – 0.84 (m, 5.74H). ^{13}C NMR (126 MHz, $CDCl_3$) δ = 166.2, 132.6, 131.0, 129.5, 128.2, 72.0, 34.4, 33.8, 27.9 ($CH(CH_3)_2$), 27.4 (t, *J* = 19.3 Hz, $CD(CH_3)_2$), 22.4, 22.4, 20.0. D NMR (77 MHz, $CHCl_3$) δ = 1.53, 0.89. HRMS (EI-TOF) calcd for $C_{14}H_{19}DO_2$ (M^+) = 221.1521, found 221.1528.

4-Methylpentyl-4-*d* benzoate (**23**):

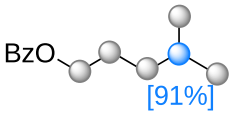

Prepared following the general procedure for 24 h with the addition of TBAB (20 mol%). The crude mixture was purified over silica gel chromatography (hexane : diethyl ether = 20 : 1) to afford product as a colorless oil in 38.0 mg (92% yield). ^{1}H NMR spectrum showed 91% deuterium incorporation on the tertiary C(sp^3)-H bond. ^{1}H NMR (500 MHz, $CDCl_3$) δ = 8.09 – 7.99 (m, 2H), 7.59 –

7.52 (m, 1H), 7.43 (t, J = 7.5 Hz, 2H), 4.31 (t, J = 7.0 Hz, 2H), 1.83 – 1.70 (m, 2H), 1.64 – 1.60 (m, 0.09H), 1.38 – 1.25 (m, 2H), 0.94 – 0.87 (m, 6H). ^{13}C NMR (126 MHz, $CDCl_3$) δ = 166.7, 132.8, 130.5, 129.5, 128.3, 65.4, 35.0, 27.7 ($CH(CH_3)_2$), 27.2 (t, J = 19.2 Hz, $CD(CH_3)_2$), 26.6, 22.4. D NMR (77 MHz, $CHCl_3$) δ = 4.32, 1.77, 1.61, 1.32, 0.93. HRMS (EI-TOF) calcd for $C_{13}H_{17}DO_2$ (M^+) = 207.1364, found 207.1364.

3-Methylbutyl-3-*d* benzoate (**24**):

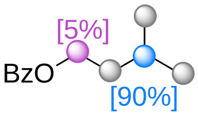

Prepared following the general procedure for 24 h with the addition of TBAB (20 mol%). The crude mixture was purified over silica gel chromatography (hexane : diethyl ether = 20 : 1) to afford product as a colorless oil in 38.1 mg (99% yield). ^{1}H NMR spectrum showed 90% deuterium incorporation on the tertiary C(sp^3)-H bond. ^{1}H NMR (500 MHz, $CDCl_3$) δ = 8.40 (d, J = 8.0 Hz, 2H), 7.55 (t, J = 8.0 Hz, 1H), 7.44 (t, J = 8.0 Hz, 2H), 4.36 (t, J = 6.5 Hz, 1.89H), 1.82 – 1.76 (m, 0.10H), 1.66 (t, J = 6.5 Hz, 2H), 0.97 (s, 6H). ^{13}C NMR (126 MHz, $CDCl_3$) δ = 166.7, 132.8, 130.5, 129.5, 128.3, 63.6, 37.3, 25.1 ($CH(CH_3)_2$), 24.7 (J = 19.5 Hz, $CD(CH_3)_2$), 22.4. D NMR (77 MHz, $CHCl_3$) δ = 4.33, 1.77, 1.64, 0.96. HRMS (EI-TOF) calcd for $C_{12}H_{15}DO_2$ (M^+) = 193.1208, found 193.1207.

2-(3-Methylbutyl-1,3-d_2)isoindoline-1,3-dione (**25**):

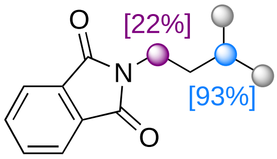

Prepared following the general procedure for 48 h with the addition of TBAB (20 mol%). The crude mixture was purified over silica gel chromatography (hexane : diethyl ether = 20 : 1) to afford product as a colorless oil in 34.5 mg (80% yield). ^{1}H NMR spectrum showed 93% deuterium incorporation on the tertiary C(sp^3)-H bond, and 22% deuterium incorporation on the *a*-amide C(sp^3)-H bonds. ^{1}H NMR (500 MHz, $CDCl_3$) δ = 7.84 – 7.79 (m, 2H), 7.72 – 7.58 (m, 2H), 3.81 – 3.42 (m, 1.57H), 1.66 – 1.60 (m, 0.07H), 1.58 – 1.47 (m, 2H), 0.96 – 0.92 (m, 6H). ^{13}C NMR (126 MHz, $CDCl_3$) δ = 168.4, 133.8, 132.2, 123.1, 37.4 – 37.0 (m), 36.4, 25.9 – 25.2 (m), 22.2. D NMR (77 MHz, $CHCl_3$) δ = 3.67, 1.58, 0.94. HRMS (EI-TOF) calcd for $C_{13}H_{13}D_2NO_2$ (M^+) = 219.1223, found 219.1219.

4-Methylcyclohexyl-4-*d* benzoate (**26**):

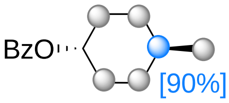

Prepared following the general procedure for 24 h with the addition of TBAB (20 mol%). The crude mixture was purified over silica gel chromatography (hexane : diethyl ether = 20 : 1) to afford product as a colorless oil in 39.2 mg

(90% yield). ^{1}H NMR spectrum showed 90% deuterium incorporation on the tertiary C(sp^{3})-H bond. *cis* : *trans* = 1 : 3, Major one, *trans*: ^{1}H NMR (500 MHz, $CDCl_3$) δ = 8.12 – 7.97 (m, 2H), 7.59 – 7.48 (m, 1H), 7.48 – 7.37 (m, 2H), 4.95 – 4.81 (m, 1H), 1.99 – 1.89 (m, 2H), 1.82 – 1.31 (m, 4.1H), 1.18 – 1.02 (m, 1.15H), 0.94 – 0.82 (m, 3H). ^{13}C NMR (126 MHz, $CDCl_3$) δ = 166.1, 132.6, 130.9, 129.5, 128.2, 73.9, 32.9 – 32.6 (m), 31.6 – 31.5 (m), 29.7 – 29.5 (m), 21.7. Minor one, *cis*, ^{1}H NMR (500 MHz, $CDCl_3$) δ = 8.12 – 7.97 (m, 2H), 7.59 – 7.48 (m, 1H), 7.48 – 7.37 (m, 2H), 5.35 – 5.18 (m, 1H), 1.99 – 1.89 (m, 2H), 1.82 – 1.31 (m, 4.1H), 1.18 – 1.02 (m, 1.15H), 1.09 – 0.95 (m, 3H). ^{13}C NMR (126 MHz, $CDCl_3$) δ = 166.0, 132.6, 131.1, 129.6, 128.2, 70.3, 32.6 – 32.2 (m), 31.2 – 30.7 (m), 29.4 – 29.0 (m), 22.0. D NMR (77 MHz, $CHCl_3$) δ = 2.05, 1.73, 1.53, 1.46, 1.37, 1.07, 0.90. HRMS (EI-TOF) calcd for $C_{14}H_{15}D_3O_2$ (M^+) = 221.1490, found 221.1485.

4-Methyl-1-phenylpentan-1-one-4-*d* (**27**):

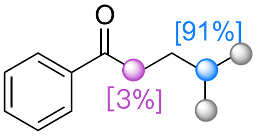

Prepared following the general procedure for 24 h with the addition of TBAB (20 mol%). The crude mixture was purified over silica gel chromatography (hexane : diethyl ether = 20 : 1) to afford product as a colorless oil in 19.7 mg (56% yield). ^{1}H NMR spectrum showed 91% deuterium incorporation on the tertiary C(sp^{3})-H bond. ^{1}H NMR (500 MHz, $CDCl_3$) δ = 7.96 (d, J = 7.5 Hz, 2H), 7.55 (t, J = 7.5 Hz, 1H), 7.46 (t, J = 7.5 Hz, 2H), 3.02 – 2.87 (m, 1.95H), 1.64 – 1.46 (m, 2.09H), 0.97 – 0.89 (m, 6H). ^{13}C NMR (126 MHz, $CDCl_3$) δ = 200.8, 137.1, 132.8, 128.5, 128.0, 36.7 – 36.5 (m), 33.1, 27.4 – 27.0 (m), 22.4 – 22.2 (m). D NMR (77 MHz, $CHCl_3$) δ = 2.93, 1.60, 0.92. HRMS (EI-TOF) calcd for $C_{12}H_{15}DO$ (M^+) = 177.1258, found 177.1261.

1-(4-(Methoxy-*d*3)phenyl)-4-methylpentan-1-one-4-*d* (**28**):

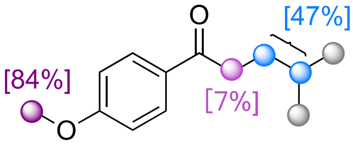

Prepared following the general procedure for 48 h with the addition of TBAB (20 mol%). The crude mixture was purified over silica gel chromatography (hexane : diethyl ether = 20 : 1) to afford product as a colorless oil in 37.2 mg (90% yield). ^{1}H NMR spectrum showed average 47% deuterium incorporation on the tertiary C(sp^{3})-H bond and adjacent secondary C(sp^{3})-H bonds which overlap with each other on the ^{1}H NMR spectrum, 7% deuterium incorporation on the *a*-oxy carbonyl bonds and 84% deuterium incorporation on the *a*-oxy C(sp^{3})-H bonds. ^{1}H NMR (500 MHz, $CDCl_3$) δ = 7.99 – 7.86 (m, 2H), 6.96 – 6.86 (m, 2H), 3.86 – 3.78 (m, 0.48H), 2.94 – 2.81 (m, 1.86H), 1.65 – 1.52 (m, 1.60H), 0.96 – 0.87 (m, 6H). ^{13}C NMR (126

MHz, $CDCl_3$) δ = 199.3, 163.2, 130.3, 130.1, 113.6, 55.3 – 54.5 (m), 36.3 – 36.0 (m), 33.4 – 32.6 (m), 28.1 – 26.3 (m), 23.5 – 22.0 (m). D NMR (77 MHz, $CHCl_3$) δ = 3.82, 2.89, 1.60, 0.92. HRMS (EI-TOF) calcd for $C_{13}H_{14}D_4O_2$ (M^+) = 210.1552, found 210.1548.

10-Bromo-2-methyldecan-5-one-2-*d* (**29**):

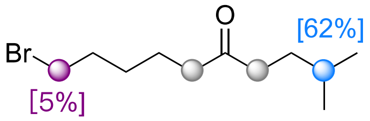

Prepared following the general procedure for 24 h with the addition of TBAB (20 mol%). The crude mixture was purified over silica gel chromatography (hexane : diethyl ether = 20 : 1) to afford product as a colorless oil in 49.2 mg (99% yield). ^{1}H NMR spectrum showed 5% deuterium incorporation on the *α*-bromide C(sp^3)-H bonds and 62% deuterium incorporation on the tertiary C(sp^3)-H bond. ^{1}H NMR (500 MHz, $CDCl_3$) δ = 3.40 (t, *J* = 7.0 Hz, 1.90H), 2.44 – 2.35 (m, 4H), 1.92 – 1.82 (m, 2H), 1.62 – 1.52 (m, 2H), 1.52 – 1.50 (m, 0.38H), 1.49 – 1.36 (m, 4H), 0.89 – 0.86 (m, 6H). ^{13}C NMR (126 MHz, $CDCl_3$) δ = 211.2, 42.4 – 42.2 (m), 40.9, 40.9, 33.6, 32.7 – 32.4 (m), 27.8, 27.7, 22.9 – 22.6 (m), 22.4, 22.2. D NMR (77 MHz, $CHCl_3$) δ = 3.39, 2.39, 1.50. HRMS (EI-TOF) calcd for $C_{11}H_{20}DBrO$ (M^+) = 249.0833, found 249.0823.

2-Methyl-10-phenoxydecan-5-one-2,10,10-d_3 (**30**):

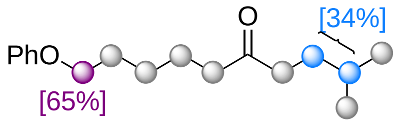

Prepared following the general procedure for 24 h with the addition of TBAB (20 mol%). The crude mixture was purified over silica gel chromatography (hexane : diethyl ether = 20 : 1) to afford product as a colorless oil in 51.9 mg (99% yield). ^{1}H NMR spectrum showed average 34% deuterium incorporation on the tertiary C(sp^3)-H adjacent secondary C(sp^3)-H bonds which overlap with each other in the ^{1}H NMR spectrum, and 65% deuterium incorporation on the *α*-oxy C(sp^3)-H bonds. ^{1}H NMR (500 MHz, $CDCl_3$) δ = 7.32 – 7.25 (m, 2H), 6.94 (t, *J* = 7.5 Hz, 1H), 6.89 (d, *J* = 8.5 Hz, 2H), 4.00 – 3.92 (m, 0.70H), 2.47 – 2.34 (m, 4H), 1.82 – 1.74 (m, 2H), 1.68 – 1.61 (m, 1.97H), 1.51 – 1.41 (m, 4H), 0.92 – 0.88 (m, 5.89H). ^{13}C NMR (126 MHz, $CDCl_3$) δ = 211.4, 159.0, 129.4, 120.5, 114.4, 67.5 - 67.1 (m), 42.6 – 42.3 (m), 40.9 – 40.6 (m), 32.7 – 32.4 (m), 29.1 ($CH_2CH(CH_3)_2$), 28.8 (t, *J* = 11.8 Hz, $CHDCH(CH_3)_2$), 27.7 ($CH(CH_3)_2$), 27.2 (t, *J* = 19.4 Hz, $CD(CH_3)_2$), 25.8 – 25.4 (m), 23.7 – 23.2 (m), 22.5 – 22.1 (m). D NMR (77 MHz, $CHCl_3$) δ = 3.95, 2.44, 1.78, 1.63, 1.53, 0.90. HRMS (EI-TOF) calcd for $C_{17}H_{21}D_5O_2$ (M^+) = 267.2241, found 267.2239.

2-Methyl-10-(phenylthio)decan-5-one-2,10,10-d_3 (**31**):

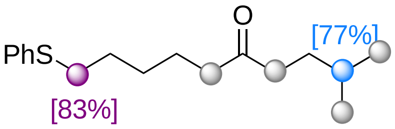

Prepared following the general procedure for 24 h with the addition of TBAB (20 mol%). The crude mixture was purified over silica gel chromatography (hexane : diethyl ether = 20 : 1) to afford product as a colorless oil in 52.8 mg (95% yield). ^{1}H NMR spectrum showed 77% deuterium incorporation on the tertiary C(sp^3)-H bond, and 83% deuterium incorporation on the *a*-thioxy C(sp^3)-H bonds. ^{1}H NMR (500 MHz, $CDCl_3$) δ = 7.33 – 7.29 (m, 2H), 7.29 – 7.25 (m, 2H), 7.18 – 7.13 (m, 1H), 2.92 – 2.86 (m, 0.34H), 2.42 – 2.31 (m, 3.91H), 1.67 – 1.60 (m, 2H), 1.60 – 1.53 (m, 2H), 1.52 – 1.48 (m, 0.23H), 1.46 – 1.35 (m, 4H), 0.90 – 0.84 (m, 6H). ^{13}C NMR (126 MHz, $CDCl_3$) δ = 211.3, 136.7, 128.9, 128.8, 125.7, 42.4, 40.9, 40.8, 32.6 - 32.5 (m), 28.8 - 28.6 (m), 28.2, 27.7, 23.3, 22.3, 22.2. D NMR (77 MHz, $CHCl_3$) δ = 2.88, 2.36, 1.49, 0.89, HRMS (EI-TOF) calcd for $C_{17}H_{23}D_3OS$ (M^+) = 281.1887, found 281.1881.

4-(Methyl-d_3)-1,1'-biphenyl (**32**):

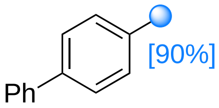

Prepared following the general procedure for 48 h with the addition of TBAB (20 mol%). The crude mixture was purified over silica gel chromatography (hexane : diethyl ether = 20 : 1) to afford product as a colorless solid in 33.2 mg (99% yield), and ^{1}H NMR spectrum showed 81% deuterium incorporation on the benzylic C(sp^3)-H bonds. After second running with the 81% deuteride product, the deuterium incorporation was improved to 90%. ^{1}H NMR (500 MHz, $CDCl_3$) δ = 7.78 – 7.66 (m, 2H), 7.65 – 7.59 (m, 2H), 7.58 – 7.51 (m, 2H), 7.51 – 7.42 (m, 1H), 7.41 – 7.32 (m, 2H), 2.54 – 2.43 (m, 0.30H). ^{13}C NMR (126 MHz, $CDCl_3$) δ = 141.1, 138.3, 136.9, 129.4, 128.7, 126.9, 126.9, 21.1 – 20.1 (m). HRMS (EI-TOF) calcd for $C_{13}H_9D_3$ (M^+) = 171.1122, found 171.1114.

4,4,5,5-Tetramethyl-2-(4-(methyl-d_3)phenyl)-1,3,2-dioxaborolane (**33**):

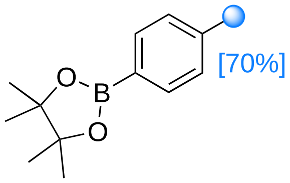

Prepared following the general procedure for 48 h with the addition of TBAB (20 mol%). The crude mixture was purified over silica gel chromatography (hexane : diethyl ether = 5 : 1) to afford product as colorless solid in 43.1 mg (99% yield), and the ^{1}H NMR spectrum showed 70% deuterium incorporation on the benzylic C(sp^3)-H bonds. ^{1}H NMR (500 MHz, $CDCl_3$) δ = 7.71(d, *J* = 6.5 Hz, 2H), 7.19 (d, *J* = 6.5 Hz, 2H), 2.38 – 2.32 (m, 0.90H), 1.35 (s, 12H). ^{13}C NMR (126 MHz, $CDCl_3$) δ = 141.3, 134.8, 128.5, 83.6, 24.8, 21.7 – 21.5 (m). HRMS (EI-TOF) calcd for $C_{13}H_{16}D_3BO_2$ (M^+) = 221.1661, found 221.1657.

1-Bromo-4-(methoxy-d_3)benzene (**21**):

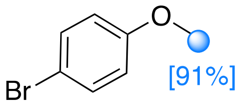

Prepared following the general procedure for 48 h with the addition of TBAB (20 mol%). The crude mixture was purified over silica gel chromatography (hexane : diethyl ether = 10 : 1) to afford product as a colorless solid in 31.8 mg (85% yield), and the ^{1}H NMR spectrum showed 91% deuterium incorporation on the *α*-oxy C(sp^3)-H bonds. ^{1}H NMR (500 MHz, $CDCl_3$) δ = 7.52 – 7.34 (m, 2H), 6.91 – 6.73 (m, 2H), 3.79 – 3.72 (m, 0.27H). ^{13}C NMR (126 MHz, $CDCl_3$) δ = 158.7, 132.2, 115.7, 112.7, 55.2 – 54.6 (m). HRMS (EI-TOF) calcd for $C_7H_4D_3BrO$ (M^+) = 188.9861, found 188.9859.

((Propan-2-yl-2-*d*)oxy)benzene (**34**):

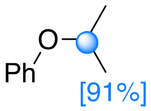

Prepared following the general procedure for 48 h with the addition of TBAB (20 mol%). The yield was detected to be 99% using CH_2Br_2 as internal standard. Finally, the crude mixture was purified over silica gel chromatography (pure hexane) to afford product as a colorless oil which still contained some diethyl ether, but ^{1}H NMR spectrum was clean enough to show 91% deuterium incorporation on the *α*-oxy C(sp^3)-H bonds. ^{1}H NMR (500 MHz, $CDCl_3$) δ = 7.33 – 7.27 (m, 2H), 7.00 – 6.90 (m, 3H), 4.61 – 4.53 (m, 0.09H), 1.39 – 1.35 (m, 6H). ^{13}C NMR (126 MHz, $CDCl_3$) δ = 157.8, 129.4, 120.4, 115.8, 70.0 – 69.0 (m), 21.9. HRMS (EI-TOF) calcd for $C_9H_{11}DO$ (M^+) = 137.0945, found 137.0947.

Chroman-4-one-2,2-d_2 (**35**):

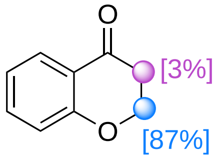

Prepared following the general procedure for 48 h with the addition of TBAB (20 mol%). The crude mixture was purified over silica gel chromatography (hexane : diethyl ether = 10 : 1) to afford product as a colorless oil in 26.7 mg (90% yield), and ^{1}H NMR spectrum showed 3% deuterium incorporation on the *α*-carbonyl C(sp^3)-H bonds and 87% deuterium incorporation on the *α*-oxy C(sp^3)-H bonds. ^{1}H NMR (500 MHz, $CDCl_3$) δ = 7.89 (dd, *J* = 7.5, 2.0 Hz, 1H), 7.49 – 7.43 (m, 1H), 7.04 – 6.97 (m, 1H), 6.96 (d, *J* = 8.0 Hz, 1H), 4.55 – 4.47 (m, 0.27 H), 2.79 (s, 1.94H). ^{13}C NMR (126 MHz, $CDCl_3$) δ = 191.8, 161.8, 135.9, 127.1, 121.3, 117.8, 66.9 – 66.0 (m), 37.5. D NMR (500 MHz, $CHCl_3$) δ = 4.50, 2.78. HRMS (EI-TOF) calcd for $C_9H_6D_2O_2$ (M^+) = 150.0644, found 150.0643.

(4-(Methoxy-d_3)phenyl)(4-(trifluoromethyl)phenyl)methanone (**36**):

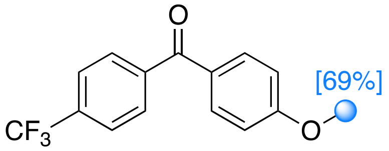

Prepared following the general procedure for 48 h with the addition of TBAB (20 mol%) and $C_6H_5CF_3$ (0.2 mL). The crude mixture was purified over silica gel chromatography (hexane : diethyl ether = 10 : 1) to afford a colorless solid in 55.4 mg (99% yield), and ^{1}H NMR spectrum showed 69% deuterium incorporation on the *a*-oxy C(sp^3)-H bonds. ^{1}H NMR (500 MHz, $CDCl_3$) δ = 7.88 – 7.77 (m, 4H), 7.73 (d, *J* = 8.5 Hz, 2H), 6.97(d, *J* = 8.5 Hz, 2H), 3.91 – 3.83 (m, 0.92H). ^{13}C NMR (126 MHz, $CDCl_3$) δ = 194.2, 163.7, 141.5, 133.2 (q, *J* = 32.8 Hz), 132.6, 129.7, 129.3, 125.2 (q, *J* = 3.8 Hz), 123.7 (q, *J* = 272.8 Hz), 113.8, 55.5 – 54.7 (m). HRMS (EI-TOF) calcd for $C_{15}H_8D_3F_3O_2$ (M^+) = 283.0894, found 283.0896.

tert-Butyl (methyl-d_3)(phenyl)carbamate (**37**):

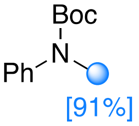

Prepared following the general procedure for 48 h with the addition of TBAB (20 mol%). The crude mixture was purified over silica gel chromatography (hexane : diethyl ether = 5 : 1) to afford product as a colorless oil in 37.7 mg (90% yield), and ^{1}H NMR spectrum showed 91% deuterium incorporation on the *a*-amide C(sp^3)-H bonds. ^{1}H NMR (500 MHz, $CDCl_3$) δ = 7.34 – 7.29 (m, 2H), 7.23 (d, *J* = 8.0 Hz, 2H), 7.17 – 7.13 (m, 1H), 3.26 – 3.21 (m, 0.26 H), 1.45 (s, 9H). ^{13}C NMR (126 MHz, $CDCl_3$) δ = 154.8, 143.8, 128.5, 125.5, 125.3, 80.2, 37.2 – 36.3 (m), 28.3. HRMS (EI-TOF) calcd for $C_{12}H_{14}D_3NO_2$ (M^+) = 210.1442, found 210.1446.

tert-Butyl indoline-1-carboxylate-2,2,3-d_3 (**38**):

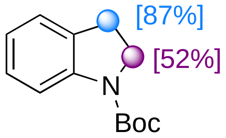

Prepared following the general procedure for 48 h with the addition of TBAB (20 mol%). The crude mixture was purified over silica gel chromatography (hexane : diethyl ether = 5 : 1) to afford product as a colorless solid in 37.2 mg (85% yield), and ^{1}H NMR spectrum showed 87% deuterium incorporation on the benzylic C(sp^3)-H bonds and 52% deuterium incorporation on the *a*-amide C(sp^3)-H bonds. ^{1}H NMR (500 MHz, $CDCl_3$) δ = 8.01 – 7.43 (m, 1H), 7.21 – 7.06 (m, 2H), 6.92 (t, *J* = 7.5 Hz, 1H), 3.96 (bs, 0.96H), 3.11 – 3.02 (m, 0.27H), 1.57 (s, 9H). ^{13}C NMR (126 MHz, $CDCl_3$) δ = 152.6, 143.3 – 143.0 (m), 132.1 – 130.0 (m), 127.3, 124.9 – 124.3 (m), 122.0, 114.6, 80.2 – 77.5 (m), 47.5 – 47.2 (m), 28.4, 27.1 – 26.2 (m). HRMS (EI-TOF) calcd for $C_{13}H_{14}D_3NO_2$ (M^+) = 222.1442, found 222.1437.

4-Phenylbutanoic-4,4-d_2 acid (**39**):

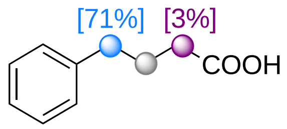

Prepared following the general procedure for 48 h with the addition of TBAB (20 mol%). The crude mixture was purified over silica gel chromatography (dichloromethane : methanol = 20 : 1) to afford product as a colorless solid in 29.5 mg (90% yield), and ^{1}H NMR spectrum showed 71% deuterium incorporation on the benzylic C(sp^3)-H bonds and 3% deuterium incorporation on the *a*-carboxylic acid C(sp^3)-H bonds. ^{1}H NMR (500 MHz, $CDCl_3$) δ = 7.30 (t, *J* = 7.5 Hz, 2H), 7.23 – 7.15 (m, 3H), 2.73 – 2.63 (m, 0.58H), 2.39 (t, *J* = 7.0 Hz, 2H), 2.02 – 1.83 (m, 1.94H). ^{13}C NMR (126 MHz, $CDCl_3$) δ = 179.9, 141.2 – 141.0 (m), 128.4, 128.4, 126.0, 35.0 – 34.3 (m), 33.3 – 33.0 (m), 26.1 – 26.0 (m). D NMR (77 MHz, $CHCl_3$) δ = 2.68, 2.40, 1.98. HRMS (EI-TOF) calcd for $C_{10}H_{10}D_2O_2$ (M$^+$) = 166.0957, found 166.0955.

Methyl (2*S*)-2-(4-(2-methylpropyl-1,2-d_2)phenyl)propanoate (**40**):

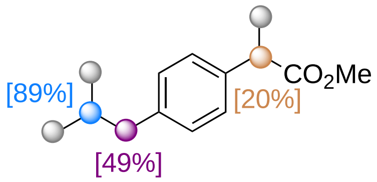

Prepared following the general procedure for 24 h with the addition of TBAB (20 mol%). The crude mixture was purified over silica gel chromatography (hexane : diethyl ether = 50 : 1) to afford product as a colorless solid in 40.5 mg (92% yield), and ^{1}H NMR spectrum showed 89% deuterium incorporation on the isopropyl C(sp^3)-H bond, 49% deuterium incorporation on the benzylic C(sp^3)-H bonds and 20% deuterium incorporation on the *a*-ester C(sp^3)-H bonds. ^{1}H NMR (500 MHz, $CDCl_3$) δ = 7.21 (d, *J* = 8.0 Hz, 2H), 7.10 (d, *J* = 8.0 Hz, 2H), 3.71 (m, 0.80H), 3.66 (s, 3H), 2.47 – 2.40 (m, 1.03H), 1.91 – 1.80 (m, 0.11H), 1.53 – 1.43 (m, 3H), 0.94 – 0.86 (m, 6H). ^{13}C NMR (126 MHz, $CDCl_3$) δ = 175.2, 140.5, 137.7, 129.3, 127.1, 51.9, 45.0, 30.1 – 29.8 (m), 29.7 – 29.4 (m), 22.2, 18.6, 18.5. D NMR (77 MHz, $CHCl_3$) δ = 3.70, 2.44, 1.84, 1.50, 0.91. HRMS (EI-TOF) calcd for $C_{14}H_{17}D_3O_2$ (M$^+$) = 223.1646, found 223.1647.

Methyl 2,2-dimethyl-5-(2-(methyl-*d*)-5-(methyl-d_2)phenoxy)pentanoate-5-*d* (**41**):

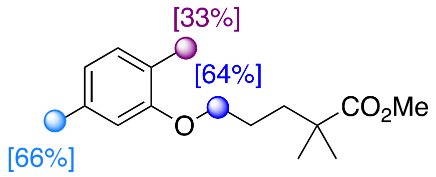

Prepared following the general procedure for 48 h with the addition of TBAB (20 mol%). The crude mixture was purified over silica gel chromatography (hexane : diethyl ether = 10 : 1) to afford product as a colorless solid in 47.7 mg (90% yield), and ^{1}H NMR spectrum showed 66%, 33% deuterium incorporation on the benzylic C(sp^3)-H bonds individually, and 64% deuterium incorporation on the *a*-oxy C(sp^3)-

H bonds. ^{1}H NMR (500 MHz, $CDCl_3$) δ = 7.01 (d, J = 7.5 Hz, 1H), 6.67 (d, J = 7.5 Hz, 1H), 6.62 (s, 1H), 3.96 – 3.88 (m, 0.72H), 3.68 (s, 3H), 2.33 – 2.27 (m, 1.02H), 2.21 – 2.15 (m, 1.69H), 1.77 – 1.69 (m, 4H), 1.24 (s, 6H). ^{13}C NMR (126 MHz, $CDCl_3$) δ = 178.3, 156.9, 136.3, 130.2, 123.6 – 123.3 (m), 120.6, 111.9, 67.5 (dd, J = 44.7, 23.2 Hz), 51.7, 42.1, 37.1 – 37.0 (m), 25.1, 25.0, 21.4 – 20.5 (m), 15.8 – 15.1 (m). D NMR (77 MHz, $CHCl_3$) δ = 3.92, 2.32 – 2.93, 2.20 – 2.17. HRMS (EI-TOF) calcd for $C_{16}H_{19}D_5O_3$ (M^+) = 269.2034, found 269.2038.

(4-(Methyl-d_3)-1*H*-pyrazol-1-yl)(phenyl)methanone (**42**):

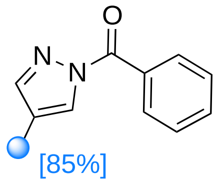

Prepared following the general procedure for 48 h with the addition of TBAB (20 mol%). The crude mixture was purified by silica gel chromatography (hexane : diethyl ether = 5 : 1) to afford product as a colorless solid in 33.1 mg (89% yield), and ^{1}H NMR spectrum showed 85% deuterium incorporation on the benzylic C(sp^3)-H bonds. ^{1}H NMR (500 MHz, $CDCl_3$) δ = 8.18 (s, 1H), 8.09 – 8.05 (m, 2H), 7.63 (s, 1H), 7.61 – 7.55 (m, 1H), 7.52 – 7.45 (m, 2H), 2.15 – 2.11 (m, 0.44H). ^{13}C NMR (126 MHz, $CDCl_3$) δ = 166.3, 146.2, 132.7, 131.7, 131.3, 128.1, 128.0, 120.2, 8.5 – 8.3 (m). HRMS (EI-TOF) calcd for $C_{11}H_7D_3N_2O$ (M^+) = 189.0976, found 189.0973.

5-(Methyl-d_3)-2-phenyl-2,4-dihydro-3*H*-pyrazol-3-one (**43**):

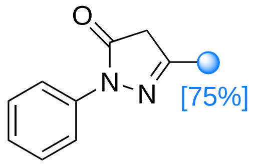

Prepared following the general procedure for 48 h with the addition of TBAB (20 mol%). The crude mixture was purified over silica gel chromatography (hexane : diethyl ether = 5 : 1) to afford the product as a colorless solid in 34.6 mg (99% yield), and ^{1}H NMR spectrum showed 75% deuterium incorporation on the methyl C(sp^3)-H bonds. ^{1}H NMR (500 MHz, $CDCl_3$) δ = 7.85 (d, J = 8.0 Hz, 2H), 7.38 (t, J = 8.0 Hz, 2H), 7.17 (t, J = 8.0 Hz, 1H), 3.41 (s, 2H), 2.19 – 2.12 (m, 0.74H). ^{13}C NMR (126 MHz, $CDCl_3$) δ = 170.5, 156.2, 138.0, 128.8, 125.0, 118.8, 43.0, 16.7 – 16.2 (m). HRMS (EI-TOF) calcd for $C_{10}H_7D_3N_2O$ (M^+) = 177.0976, found 177.0972.

5-(Methyl-d_3)-1-phenylpyridin-2(1*H*)-one (**44**):

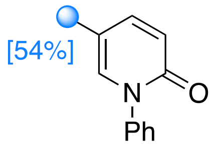

Prepared following the general procedure for 48 h with the addition of TBAB (20 mol%) after mixing with trifluoroacetic acid (3.0 equiv) in D_2O. The crude mixture was purified over silica gel chromatography (hexane :

diethyl ether = 2 : 1) to afford the product as a colorless solid in 31.7 mg (85% yield), and ^{1}H NMR spectrum showed 54% deuterium incorporation on the benzylic C(sp^3)-H bonds. ^{1}H NMR (500 MHz, $CDCl_3$) δ = 7.52 – 7.46 (m, 2H), 7.44 – 7.39 (m, 1H), 7.38- 7.35 (m, 2H), 7.27 (dd, J = 7.0, 2.5 Hz, 1H), 7.12 (s, 1H), 6.62 (d, J = 9.5 Hz, 1H), 2.12 – 2.06 (m, 1.39H). ^{13}C NMR (126 MHz, $CDCl_3$) δ = 161.7, 142.5, 141.1, 135.3, 129.3, 128.3, 126.5, 121.4, 114.8 – 114.7 (m), 17.0 – 16.4 (m). HRMS (EI-TOF) calcd for $C_{12}H_8D_3NO$ (M^+) = 188.1023, found 188.1018.

3-Butylisobenzofuran-1(3*H*)-one-3-*d* (**45**):

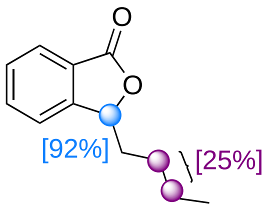

Prepared following the general procedure for 24 h with the addition of TBAB (20 mol%). The crude mixture was purified over silica gel chromatography (hexane : diethyl ether = 2 : 1) to afford product as a colorless oil in 32.0 mg (84% yield), and ^{1}H NMR spectrum showed 92% deuterium incorporation on the *α*-oxy C(sp^3)-H bond, and 25% deuterium incorporation on the indicated secondary C(sp^3)-H bonds. ^{1}H NMR (500 MHz, $CDCl_3$) δ = 7.87 (d, J = 8.0 Hz, 1H), 7.65 (t, J = 8.0 Hz, 1H), 7.50 (t, J = 7.5 Hz, 1H), 7.43 (d, J = 7.5 Hz, 1H), 5.46 (dd, J = 7.5, 4.0 Hz, 0.08H), 2.08 – 1.95 (m, 1H), 1.80 – 1.68 (m, 1H), 1.53 – 1.25 (m, 3.00H), 0.98 – 0.82 (m, 3H). ^{13}C NMR (126 MHz, $CDCl_3$) δ = 170.6, 150.0, 133.9, 129.0, 126.1, 125.6, 121.7, 81.4 (OCH*n*Bu), 81.0 (t, J = 22.4 Hz, OCD*n*Bu), 34.4 – 34.1 (m), 26.8 – 26.2 (m), 22.5 – 20.8 (m), 13.9 – 13.1 (m). D NMR (77 MHz, $CHCl_3$) δ = 5.45, 1.35. HRMS (EI-TOF) calcd for $C_{12}H_{12}D_2O_2$ (M^+) = 192.1114, found 192.1115.

N-(2,6-bis(methyl-*d*3)phenyl)-2-(diethylamino)acetamide (**46**):

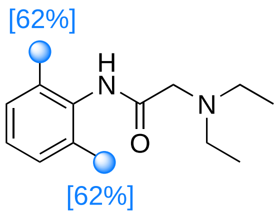

Prepared following the general procedure for 48 h with the addition of TBAB (20 mol%) after mixing with trifluoroacetic acid (3.0 equiv) in D_2O. The crude mixture was purified by silica gel chromatography (hexane : diethyl ether = 5 : 1) to afford product as a colorless solid in 39.4 mg (84% yield), and ^{1}H NMR spectrum showed 62% deuterium incorporation on the benzylic C(sp^3)-H bonds. ^{1}H NMR (500 MHz, $CDCl_3$) δ = 8.92 (bs, 1H), 7.15 – 7.00 (m, 3H), 3.22 (s, 2H), 2.69 (q, J = 7.0 Hz, 4H), 2.24 – 2.18 (m, 2.31H), 1.14 (t, J = 7.0 Hz, 6H). ^{13}C NMR (126 MHz, $CDCl_3$) δ = 170.3, 135.0, 134.0, 128.2, 127.0, 57.5, 48.9, 18.6 – 18.0 (m), 12.6. D NMR (77 MHz, $CHCl_3$) δ = 3.21 – 3.18. HRMS (EI-TOF) calcd for $C_{14}H_{18}D_4NO$ (M^+) = 238.1978, found 238.1970.

N-(1-(2,6-bis(methyl-d_3)phenoxy)propan-2-yl)benzamide (**47**):

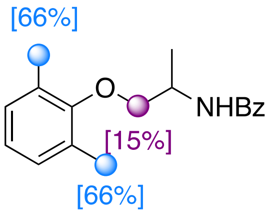

Prepared following the general procedure for 48 h with the addition of TBAB (20 mol%). The crude mixture was purified over silica gel chromatography (hexane : diethyl ether = 10 : 1) to afford product as a colorless solid in 49.4 mg (87% yield), and ^{1}H NMR spectrum showed 66% deuterium incorporation on the benzylic C(sp^3)-H bonds, and 15% deuterium incorporation on the α-oxy C(sp^3)-H bonds. ^{1}H NMR (500 MHz, CDCl$_3$) δ = 7.87 – 7.78 (m, 2H), 7.56 – 7.48 (m, 1H), 7.48 – 7.32 (m, 2H), 7.01 (d, J = 7.5 Hz, 2H), 6.97 – 6.89 (m, 1H), 6.73 (bs, 1H), 4.62 – 4.49 (m, 1H), 3.98 – 3.88 (m, 0.88H), 3.87 – 3.76 (m, 0.83H), 2.29 – 2.21 (m, 2.03H), 1.53 (d, J = 7.0 Hz, 3H). ^{13}C NMR (126 MHz, CDCl$_3$) δ = 166.8, 154.8, 134.6, 131.4, 130.7 – 130.5 (m), 129.0, 128.5, 126.8, 124.1, 73.8, 45.8 – 45.6 (m), 17.8 – 17.6 (m), 16.2 – 15.2 (m). D NMR (77 MHz, CHCl$_3$) δ = 3.89 – 3.78, 2.24 – 2.20. HRMS (EI-TOF) calcd for $C_{18}H_{17}D_4NO_2$ (M$^+$) = 287.1818, found 287.1823.

4-Chloro-3,5-bis(methyl-d_3)phenyl acetate (**48**):

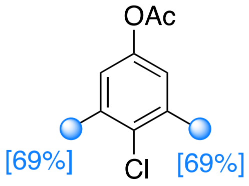

Prepared following the general procedure for 48 h with the addition of TBAB (20 mol%). The crude mixture was purified over silica gel chromatography (hexane : diethyl ether = 10 : 1) to afford product as a colorless oil in 39.2 mg (99% yield), and ^{1}H NMR spectrum showed 69% deuterium incorporation on the benzylic C(sp^3)-H bonds. ^{1}H NMR (500 MHz, CDCl$_3$) δ = 6.82 (s, 2H), 2.38 – 2.32 (m, 1.87H), 2.28 (s, 3H). ^{13}C NMR (126 MHz, CDCl$_3$) δ = 169.5, 148.2, 137.4, 131.8, 121.3, 21.0, 20.7 – 20.1 (m). HRMS (EI-TOF) calcd for $C_{10}H_7D_4ClO_2$ (M$^+$) = 202.0693, found 202.0695.

Benzo[d][1,3]dioxol-5-yl-2,2-d_2 acetate (**49**):

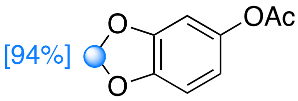

Prepared following the general procedure for 48 h with the addition of TBAB (20 mol%). The crude mixture was purified over silica gel chromatography (hexane : diethyl ether = 20 : 1) to afford product as a colorless solid in 34.2 mg (95% yield), and ^{1}H NMR spectrum showed 94% deuterium incorporation on the α-oxy C(sp^3)-H bonds. ^{1}H NMR (500 MHz, CDCl$_3$) δ = 6.77 (d, J = 9.0 Hz, 1H), 6.60 (d, J = 2.5 Hz, 1H), 6.52 (dd, J = 9.0, 2.5 Hz, 1H), 5.95 (s, 0.12H), 2.26 (s, 3H). ^{13}C NMR (126 MHz, CDCl$_3$) δ = 169.8, 148.0, 145.3, 144.9, 113.9, 107.9, 103.7, 101.6 – 100.8 (m), 20.9. HRMS (EI-TOF)

calcd for $C_9H_6D_2O_4$ (M^+) = 182.0543, found 182.0546.

(2*S*)-7-Chloro-2'-methoxy-4,6-bis(methoxy-*d*)-6'-methyl-3*H*-spiro[benzofuran-2,1'-cyclohexan]-2'-ene-3,4'-dione (**50**):

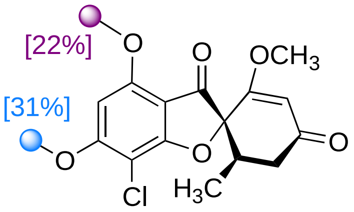

Prepared following the general procedure for 48 h with the addition of TBAB (20 mol%) and $C_6H_5CF_3$ (0.2 mL). The crude mixture was purified over silica gel chromatography (dichloromethane : diethyl ether = 1 : 1) to afford product as a colorless solid in 70.1 mg (99% yield), and ^{1}H NMR spectrum showed 31% and 22% deuterium incorporation on the *a*-oxy C(sp^3)-H bonds. ^{1}H NMR (500 MHz, $CDCl_3$) δ = 6.12 (s, 1H), 5.52 (s, 1H), 4.02 – 3.97 (m, 2.33H), 3.96 – 3.92 (m, 2.06H), 3.60 (s, 3H), 3.00 (dd, *J* = 16.5, 13.5 Hz, 1H), 2.86 – 2.77 (m, 1H), 2.41 (dd, *J* = 16.5, 4.5 Hz, 1H), 0.94 (d, *J* = 7.0 Hz, 3H). ^{13}C NMR (126 MHz, $CDCl_3$) δ = 197.0, 192.4, 170.7, 169.4, 164.6, 157.7, 104.8, 89.4, 57.2 – 56.0 (m), 40.0, 36.3, 14.2. D NMR (77 MHz, $CHCl_3$) δ = 3.99. HRMS (EI-TOF) calcd for $C_{17}H_{15}D_2ClO_6$ (M^+) = 354.0834, found 354.0831.

N-((3*s*,5*s*,7*s*)-adamantan-1-yl)benzamide-*d11* (**51**):

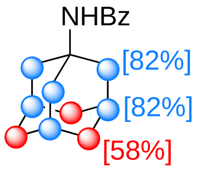

Prepared following the general procedure for 48 h with the addition of TBAB (20 mol%) and $C_6H_5CF_3$ (0.2 mL). The crude mixture was purified over silica gel chromatography (hexane : diethyl ether = 10 : 1) to afford product as a colorless solid in 50.4 mg (99% yield), and ^{1}H NMR spectrum showed 10.9 deuterium atoms per adamantyl molecule. ^{1}H NMR (500 MHz, $CDCl_3$) δ = 7.70 (d, *J* = 7.0 Hz, 2H), 7.48 – 7.41 (m, 1H), 7.41 – 7.33 (m, 2H), 5.84 (bs, 1H), 2.17 – 2.02 (m, 1.60H), 1.76 – 1.61 (m, 2.55H). ^{13}C NMR (126 MHz, $CDCl_3$) δ = 166.6 (166.5), 135.9, 130.9, 128.3, 126.6, 52.1 – 51.6 (m), 41.5 – 40.4 (m), 36.2 – 35.1 (m), 29.2 – 28.1 (m). D NMR (77 MHz, $CHCl_3$) δ = 2.04, 1.64. HRMS (EI-TOF) calcd for $C_{17}H_{10}D_{11}O$ (M^+) = 266.2304, found 266.2294.

N-((1*r*,3*R*,5*S*,7*r*)-3,5-dimethyladamantan-1-yl)benzamide-*d7* (**52**):

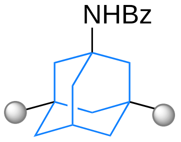

Prepared following the general procedure for 48 h with the addition of TBAB (20 mol%) and $C_6H_5CF_3$ (0.2 mL). The crude mixture was purified over silica gel chromatography (hexane : diethyl ether = 10 : 1) to afford the product as a colorless solid in 56.1 mg (99% yield), and ^{1}H NMR spectrum showed 6.7 deuterium atoms per adamantyl molecule. ^{1}H NMR (500 MHz, $CDCl_3$) δ = 7.75 – 7.65 (m, 2H), 7.47 – 7.41 (m, 1H), 7.41 – 7.33 (m, 2H), 5.90 (bs, 1H), 2.19 – 2.17 (m, 0.13H), 1.98 – 1.92 (m, 0.31H), 1.81

– 1.71 (m, 2.55H), 1.46 – 1.36 (m, 1.40 H), 1.36 – 1.25 (m, 1.56 H), 1.24 – 1.10 (m, 2.33H), 0.86 (s, 6H). ^{13}C NMR (126 MHz, $CDCl_3$) δ = 166.6, 135.8, 140.0, 128.3, 126.6, 53.6 (t, J = 8.3 Hz), 50.5, 47.5 – 46.9 (m), 42.5 – 41.8 (m), 40.7 – 40.5 (m), 32.3 (t, J = 10.5 Hz), 30.1, 39.8 – 29.0 (m). D NMR (77 MHz, $CHCl_3$) δ = 2.12, 1.89, 1.73, 1.37, 1.28, 0.86. HRMS (EI-TOF) calcd for $C_{19}H_{18}D_7NO$ (M^+) = 290.2370, found 290.2366.

Phenyl (3*r*,5*r*,7*r*)-adamantane-1-carboxylate-*d8* (**53**):

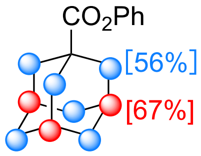

Prepared following the general procedure for 48 h with the addition of TBAB (20 mol%) and $C_6H_5CF_3$ (0.2 mL). The crude mixture was purified over silica gel chromatography (hexane : diethyl ether = 8 : 1) to afford product as a colorless solid in 50.3 mg (98% yield), and ^{1}H NMR spectrum showed 8.8 deuterium atoms per adamantyl molecule. ^{1}H NMR (500 MHz, $CDCl_3$) δ = 7.42 – 7.34 (m, 2H), 7.24 – 7.16 (m, 1H), 7.08 – 7.01 (m, 2H), 2.11 – 2.01 (m, 5.23H), 1.79 – 1.70 (m, 0.98H). ^{13}C NMR (126 MHz, $CDCl_3$) δ = 176.2, 151.1, 129.3, 125.4, 121.5, 40.9 – 40.6 (m), 38.7 – 38.3 (m), 38.3 – 37.8 (m), 36.0 – 35.1 (m), 27.9 – 26.6 (m). D NMR (77 MHz, $CHCl_3$) δ = 2.10, 1.77. HRMS (EI-TOF) calcd for $C_{17}H_{12}D_8O_2$ (M^+) = 264.1960, found 264.1955.

2-(3,4-Bis(methoxy-*d*)phenyl)acetonitrile-*d2* (**54**):

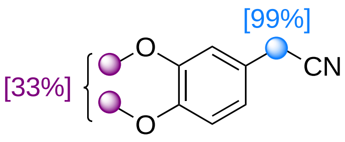

Prepared following the general procedure for 48 h with the addition of TBAB (20 mol%). The crude mixture was purified over silica gel chromatography (hexane : diethyl ether = 20 : 1) to afford product as a colorless solid in 31.2 mg (88% yield), and ^{1}H NMR spectrum showed 33% deuterium incorporation on the *a*-oxy C(sp^3)-H bonds, and 99% deuterium incorporation on the benzylic C(sp^3)-H bonds. ^{1}H NMR (500 MHz, $CDCl_3$) δ = 6.87 – 6.81 (m, 2H), 6.80 (s, 1H), 3.90 – 3.80 (m, 4.02H), 3.70 (m, 0.01H). ^{13}C NMR (126 MHz, $CDCl_3$) δ = 149.3, 148.8, 122.0, 120.1, 118.1, 111.4, 110.9, 56.0 – 55.2 (m), 22.7 (t, J = 20.5 Hz, OCH_2D). HRMS (EI-TOF) calcd for $C_{10}H_7D_4NO_2$ (M^+) = 181.1035, found 181.1027.

2,3-Dihydro-1*H*-inden-1-one-3,3-*d2* (**55**):

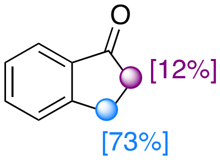

Prepared following the general procedure for 48 h with the addition of TBAB (20 mol%). The crude mixture was purified over silica gel chromatography (hexane : diethyl ether = 20 : 1) to afford product as a colorless solid in 25.2 mg (95% yield), and ^{1}H NMR spectrum showed 73% deuterium

incorporation on the benzylic C(sp^3)-H bonds, and 12% deuterium incorporation on the *a*-carbonyl C(sp^3)-H bonds. ^{1}H NMR (500 MHz, $CDCl_3$) δ = 7.75 (d, *J* = 8.0 Hz, 1H), 7.58 (dt, *J* = 7.5, 1.0 Hz, 1H), 7.48 (d, *J* = 7.5 Hz, 1H), 7.36 (t, *J* = 7.5 Hz, 1H), 3.18 – 3.08 (m, 0.55H), 2.71 – 2.62 (m, 1.76 H). ^{13}C NMR (126 MHz, $CDCl_3$) δ = 207.1, 155.0, 137.1, 134.5, 127.3, 126.7, 123.7, 36.1, 25.8 - 25.4 (m). HRMS (EI-TOF) calcd for $C_9H_6D_2O$ (M^+) = 134.0695, found 134.0692.

Methyl 2-(4-(methoxy-*d*3)phenyl)acetate-*d*2 (**56**):

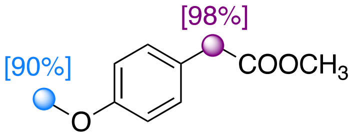

Prepared following the general procedure for 48 h with the addition of TBAB (20 mol%). The crude mixture was purified over silica gel chromatography (hexane : diethyl ether = 10 : 1) to afford product as a colorless solid in 35.7 mg (99% yield), and ^{1}H NMR spectrum showed 90% deuterium incorporation on the *a*-oxy C(sp^3)-H bonds, and 98% deuterium incorporation on the *a*-benzylic C(sp^3)-H bonds. ^{1}H NMR (500 MHz, $CDCl_3$) δ = 7.20 (d, *J* = 8.0 Hz, 2H), 6.86 (d, *J* = 8.0 Hz, 2H), 3.79 – 3.74 (m, 0.30H), 3.68 (s, 3H), 3.57 – 3.53 (m, 0.04H). ^{13}C NMR (126 MHz, $CDCl_3$) δ = 172.3, 158.7, 130.2, 125.9, 113.9, 55.0 – 54.3 (m), 51.9, 40.0 – 39.3 (m). HRMS (EI-TOF) calcd for $C_{10}H_7D_5O_3$ (M^+) = 185.1095, found 185.1093.

(2-(Methyl-*d*3)phenyl)(phenyl)methanone (**57**):

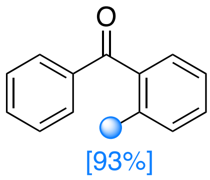

Prepared following the general procedure for 48 h with the addition of TBAB (20 mol%). The crude mixture was purified over silica gel chromatography (hexane : diethyl ether = 8 : 1) to afford product as a colorless oil in 37.7 mg (96% yield), and ^{1}H NMR spectrum showed 93% deuterium incorporation on the benzylic C(sp^3)-H bonds. ^{1}H NMR (500 MHz, $CDCl_3$) δ = 7.81 (d, *J* = 7.5 Hz, 2H), 7.62 – 7.54 (m, 1H), 7.49 – 7.43 (m, 2H), 7.42 – 7.37 (m, 1H), 7.34 – 7.28 (m, 2H), 7.27 – 7.22 (m, 1H), 2.34 – 2.29 (m, 0.21H). ^{13}C NMR (126 MHz, $CDCl_3$) δ = 198.6, 138.6, 137.7, 136.6, 133.1, 130.9, 130.2, 130.1, 128.5, 128.4, 125.1, 19.5 – 18.9 (m). HRMS (EI-TOF) calcd for $C_{14}H_{19}D_3O$ (M^+) = 199.1071, found 199.1067.

2-Bromo-6-(methoxy-d_2)naphthalene (**58**):

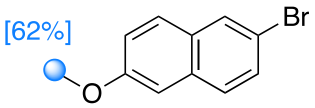

Prepared following the general procedure for 48 h with the addition of TBAB (20 mol%). The crude mixture was purified over silica gel chromatography (hexane : diethyl ether = 8 : 1) to afford product as a colorless solid in 46.7 mg (99% yield), and ^{1}H NMR spectrum showed 62% deuterium incorporation on the *a*-oxy C(sp^3)-H bonds. ^{1}H NMR (500 MHz, $CDCl_3$) δ = 7.91 (d, *J* = 2.0 Hz, 1H), 7.63 (d, *J* = 9.0 Hz, 1H), 7.60 (d, *J* = 9.0 Hz, 1H), 7.50 (dd, *J* = 9.0, 2.0Hz, 1H), 7.16 (dd, *J* = 9.0, 2.0 Hz, 1H), 7.09 (s, 1H), 3.92 – 3.86 (m, 1.15H). ^{13}C NMR (126 MHz, $CDCl_3$) δ = 157.8, 133.0, 130.0, 129.6, 129.6, 128.4, 128.3, 119.7, 117.0, 105.7, 52.3 – 54.7 (m). HRMS (EI-TOF) calcd for $C_{11}H_7D_2BrO$ (M^+) = 239.9936, found 239.9939.

N-(2,6-bis(methyl-d_3)phenyl)acetamide (**59**):

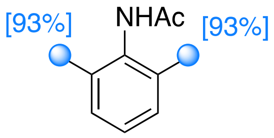

Prepared following the general procedure for 48 h with the addition of TBAB (20 mol%). The crude mixture was purified over silica gel chromatography (hexane : diethyl ether = 8 : 1) to afford product as a colorless solid in 32.2 mg (99% yield), and ^{1}H NMR spectrum showed 93% deuterium incorporation on the benzylic C(sp^3)-H bonds. ^{1}H NMR (500 MHz, $CDCl_3$) δ = 7.17 – 7.01 (m, 3H), 6.93 (br, 1H), 2.27 – 2.21 (m, 0.40H), 2.16 (s, 3H). ^{13}C NMR (126 MHz, $CDCl_3$) δ = 168.7, 136.5, 135.4, 128.6, 128.2, 128.1, 127.3, 23.0, 18.4 – 17.5 (m). HRMS (EI-TOF) calcd for $C_{10}H_7D_6NO$ (M^+) = 169.1368, found 169.1366.

7. Reference

1 I. B. Perry, T. F. Brewer, P. J. Sarver, D. M. Schultz, D. A. DiRocco and D. W. C. MacMillan, *Nature*, 2018, **560**, 70.

2 D. C. Miller, J. M. Ganley, A. J. Musacchio, T. C. Sherwood, W. R. Ewing and R. R. Knowles, *J. Am. Chem. Soc.*, 2019, **141**, 16590.

3 Y. Motoyama, M. Abe, K. Kamo, Y. Kosako and H. Nagashima, *Chem. Commun.*, 2008, 5321.

8. Copies of NMR spectra

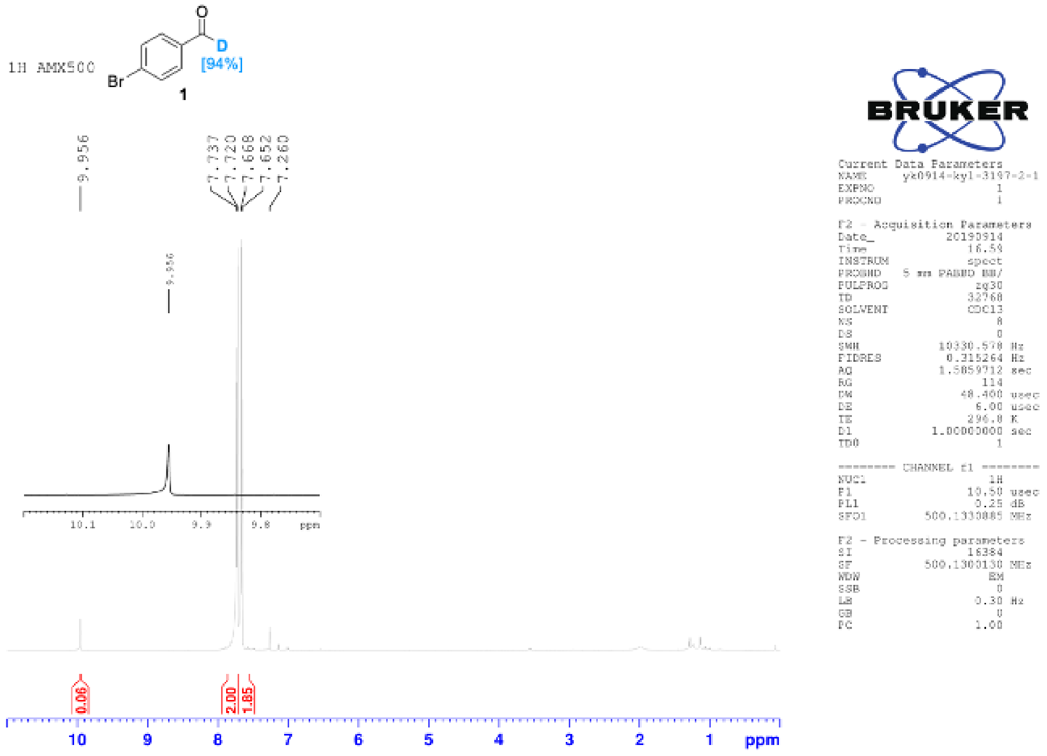

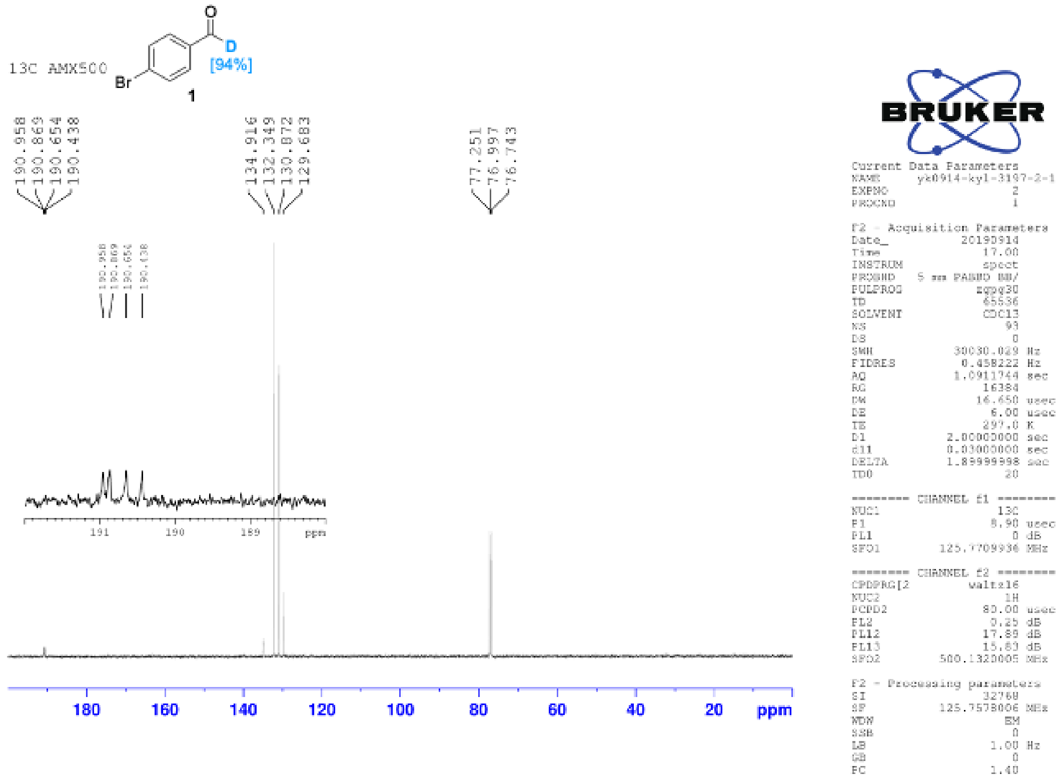

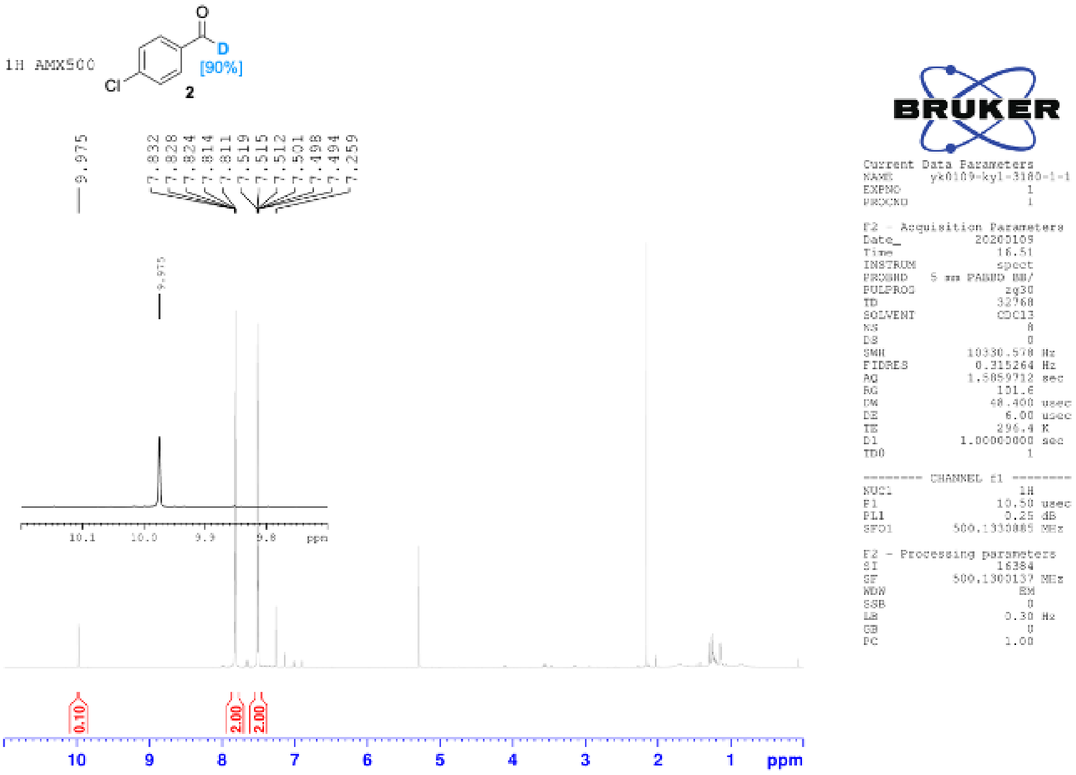
1H AMX500
O
D
[90%]
Cl
2
9.975
7.832
7.828
7.824
7.814
7.811
7.519
7.515
7.512
7.501
7.498
7.494
7.259
BRUKER
0.10
2.00
2.00
10
9
8
7
6
5
4
3
2
1
ppm

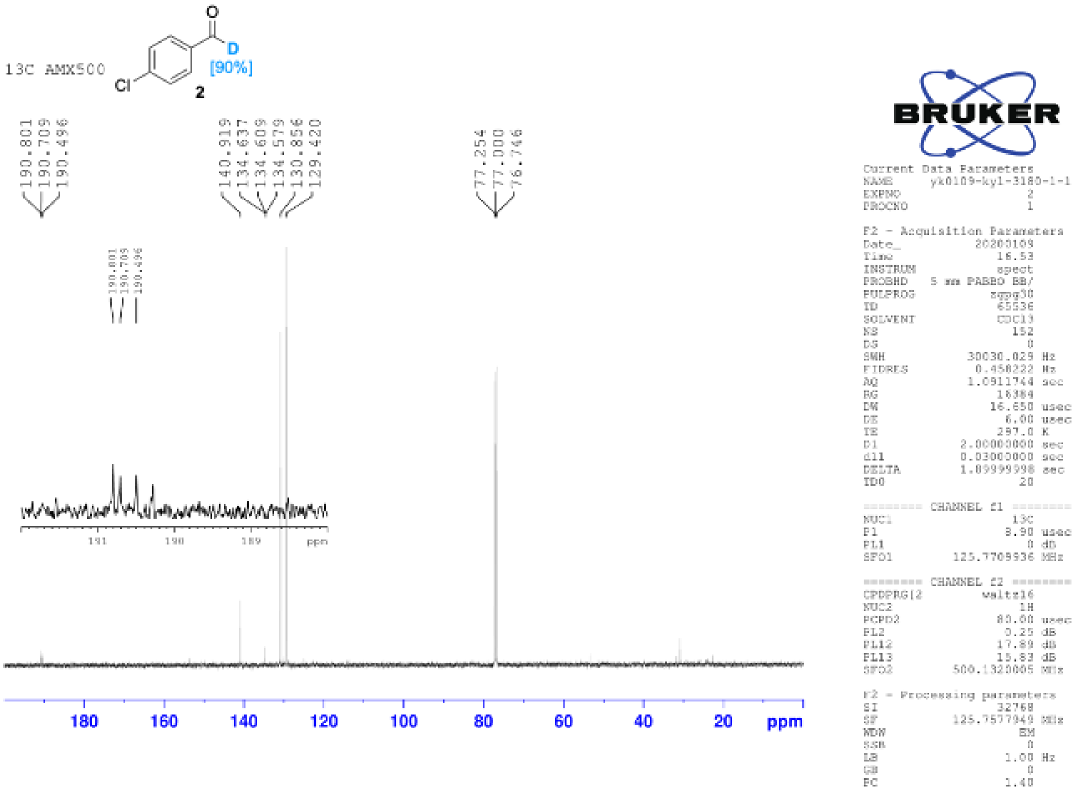
13C AMX500
O
D
[90%]
Cl
2
190.801
190.709
190.496
140.919
134.637
134.609
134.579
130.856
129.420
77.254
77.000
76.746
BRUKER
180
160
140
120
100
80
60
40
20
ppm

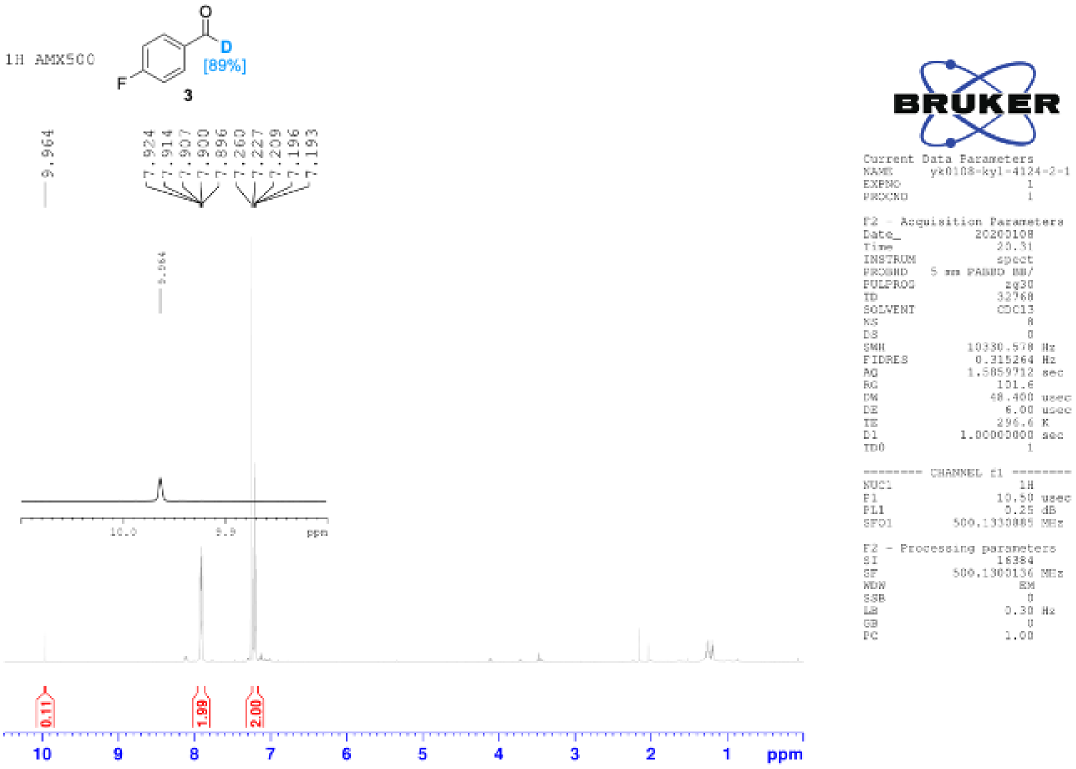
1H AMX500
O
D
[89%]
F
3
9.964
7.924
7.914
7.907
7.900
7.896
7.260
7.227
7.209
7.196
7.193
0.11
1.99
2.00
10 9 8 7 6 5 4 3 2 1 ppm
BRUKER
Current Data Parameters
NAME yk0108-kyl-4124-2-1
EXPNO 1
PROCNO 1
F2 - Acquisition Parameters
Date_ 20200108
INSTRUM spect
PROBHD 5 mm PABBO BB/
PULPROG zg30
TD 32768
SOLVENT CDCl3
NS 8
DS 0
SWH 10330.578 Hz
FIDRES 0.315264 Hz
AQ 1.5859712 sec
RG 101.6
DW 48.400 usec
DE 6.00 usec
TE 296.6 K
D1 1.00000000 sec
TD0 1
======== CHANNEL f1 ========
NUC1 1H
P1 10.50 usec
PL1 0.25 dB
SFO1 500.1330885 MHz
F2 - Processing parameters
SI 16384
SF 500.1300136 MHz
WDW EM
SSB 0
LB 0.30 Hz
GB 0
PC 1.00

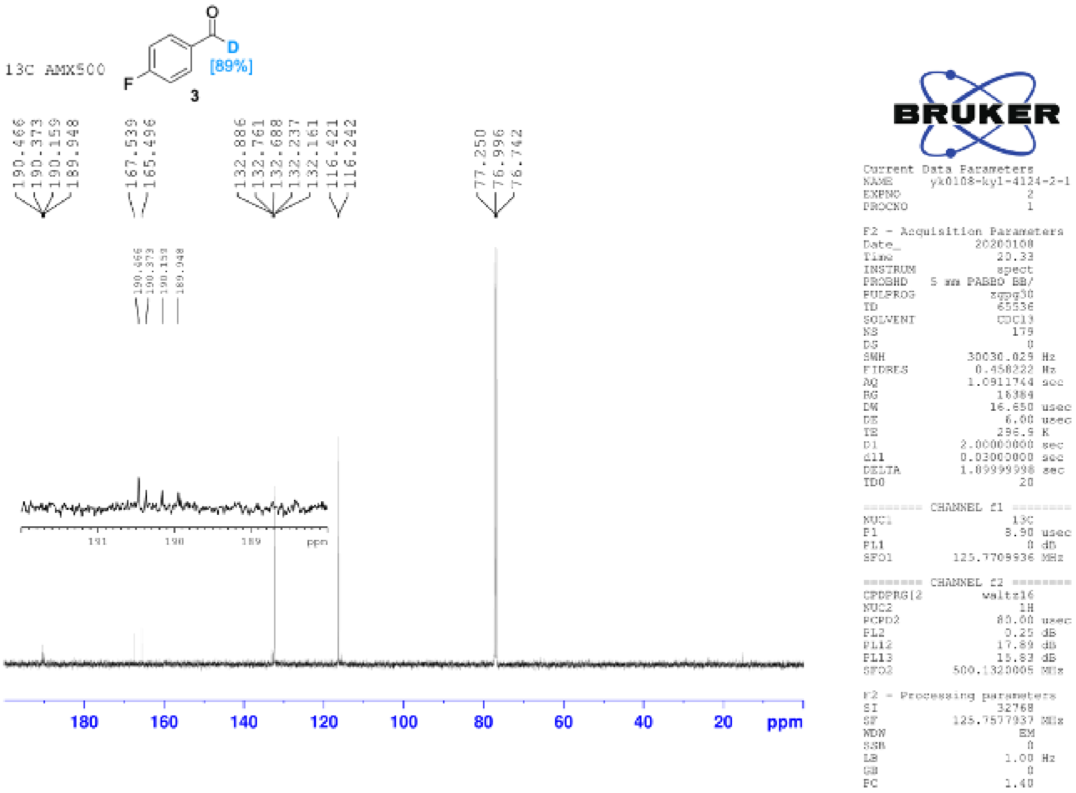
13C AMX500
O
D
[89%]
F
3
190.466
190.373
190.159
189.948
167.539
165.496
132.886
132.761
132.688
132.237
132.161
116.421
116.242
77.250
76.996
76.742
180 160 140 120 100 80 60 40 20 ppm
BRUKER
Current Data Parameters
NAME yk0108-kyl-4124-2-1
EXPNO 2
PROCNO 1
F2 - Acquisition Parameters
Date_ 20200108
INSTRUM spect
PROBHD 5 mm PABBO BB/
PULPROG zgpg30
TD 65536
SOLVENT CDCl3
NS 179
DS 0
SWH 30030.029 Hz
FIDRES 0.458222 Hz
AQ 1.0911744 sec
RG 16384
DW 16.650 usec
DE 6.00 usec
TE 296.9 K
D1 2.00000000 sec
d11 0.03000000 sec
DELTA 1.89999998 sec
TD0 20
======== CHANNEL f1 ========
NUC1 13C
P1 8.90 usec
PL1 0 dB
SFO1 125.7709936 MHz
======== CHANNEL f2 ========
CPDPRG2 waltz16
NUC2 1H
PCPD2 80.00 usec
PL2 0.25 dB
PL12 17.89 dB
PL13 15.83 dB
SFO2 500.1320005 MHz
F2 - Processing parameters
SI 32768
SF 125.7577937 MHz
WDW EM
SSB 0
LB 1.00 Hz
GB 0
PC 1.40

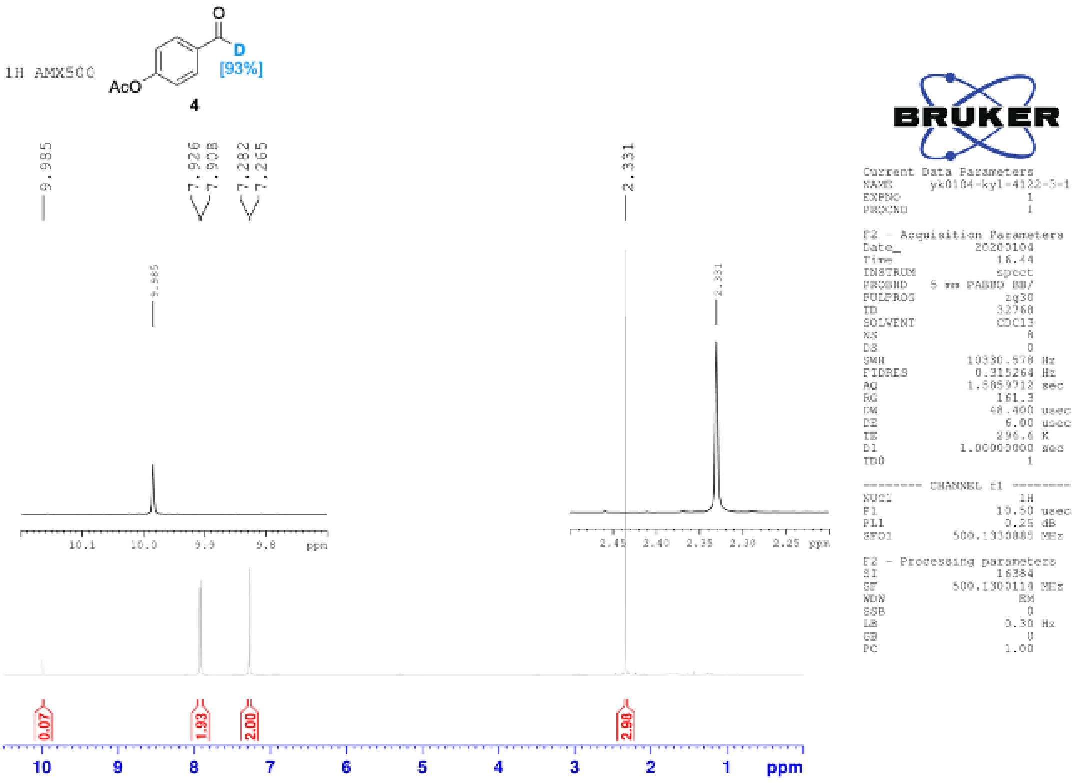

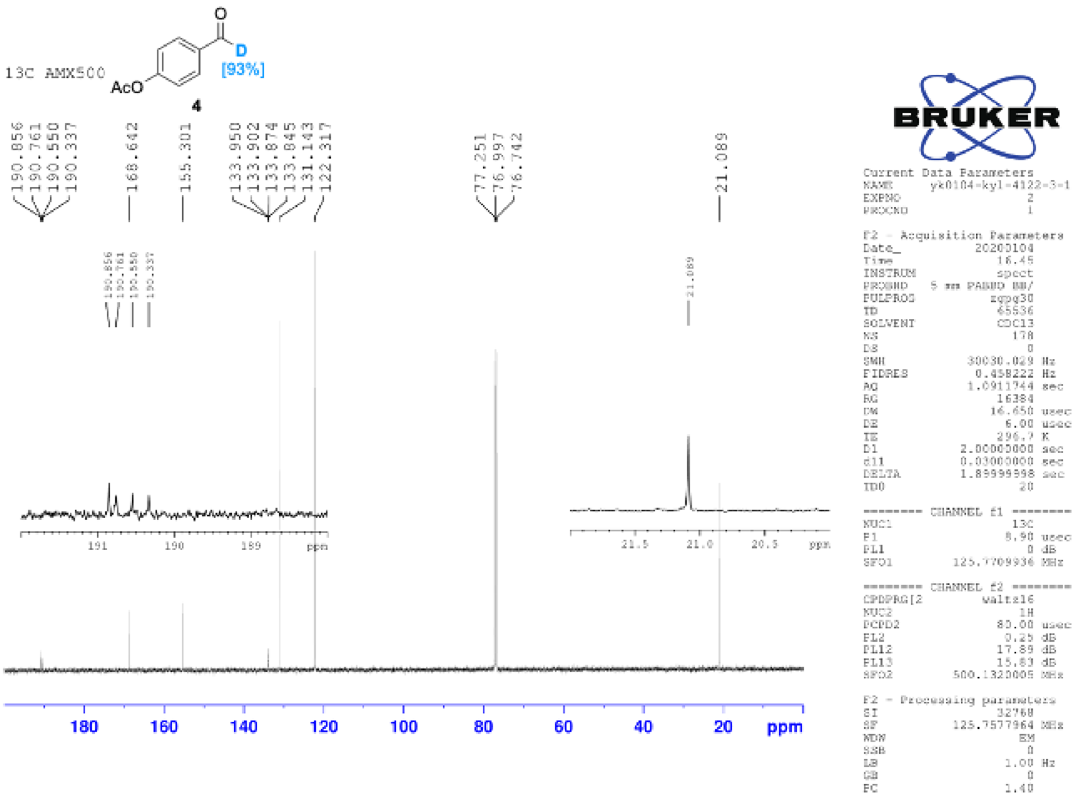

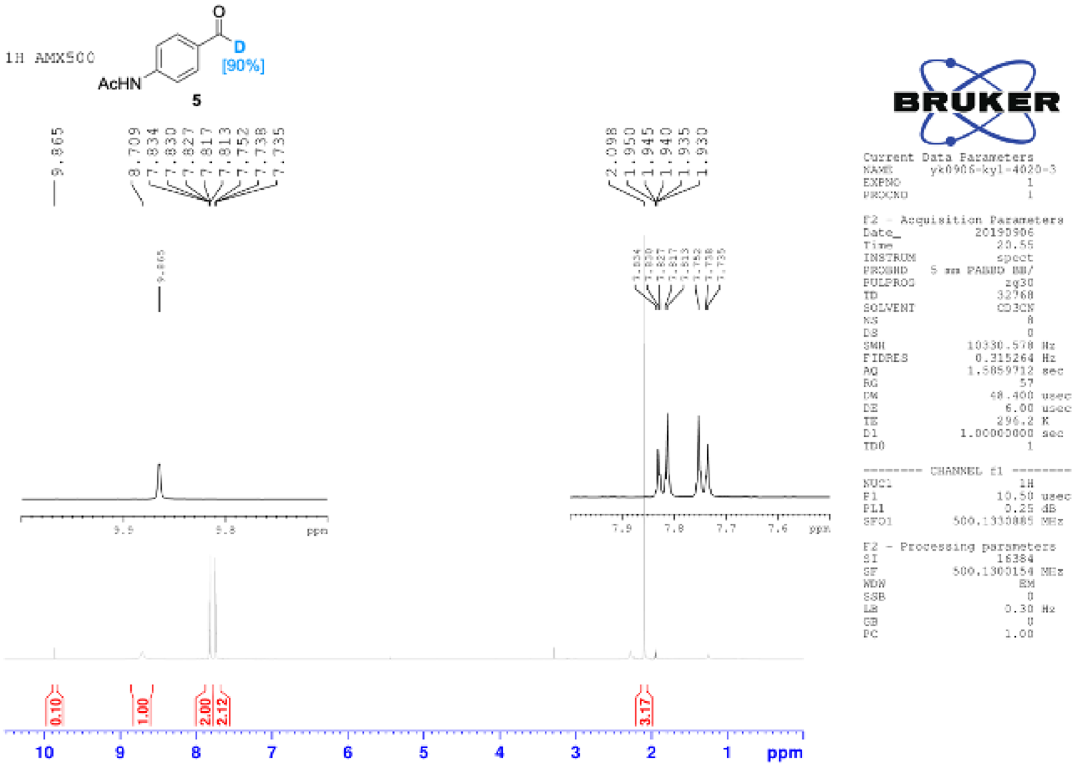

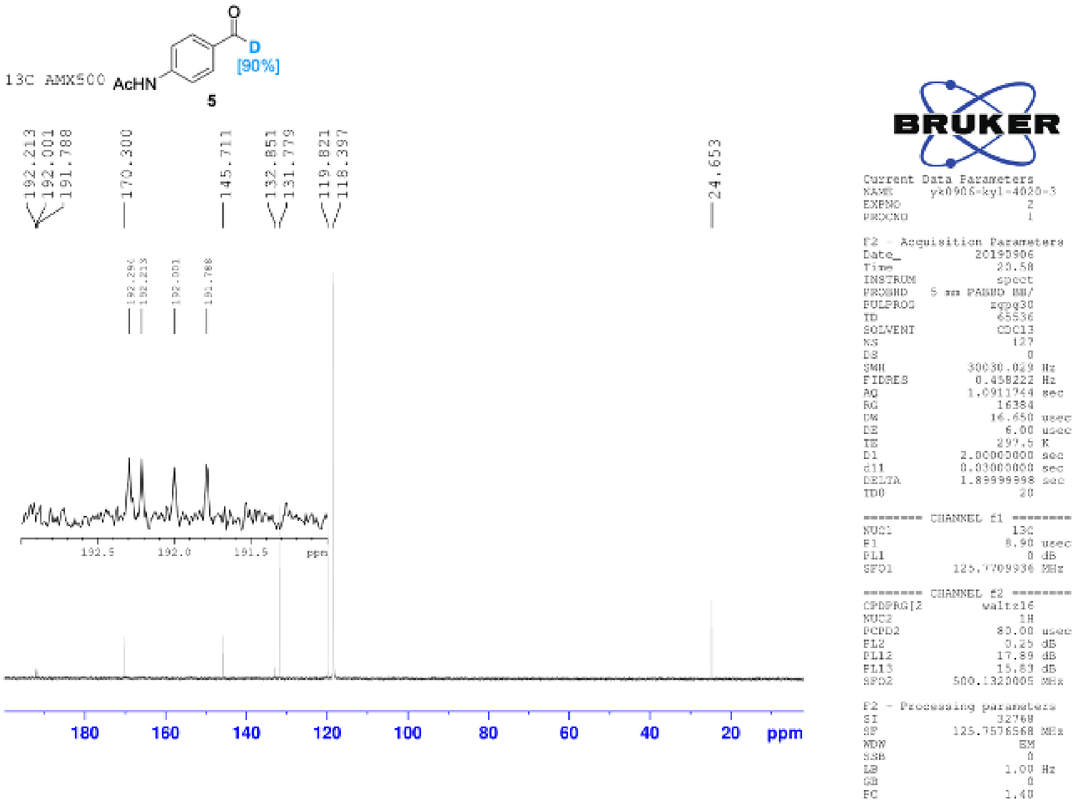

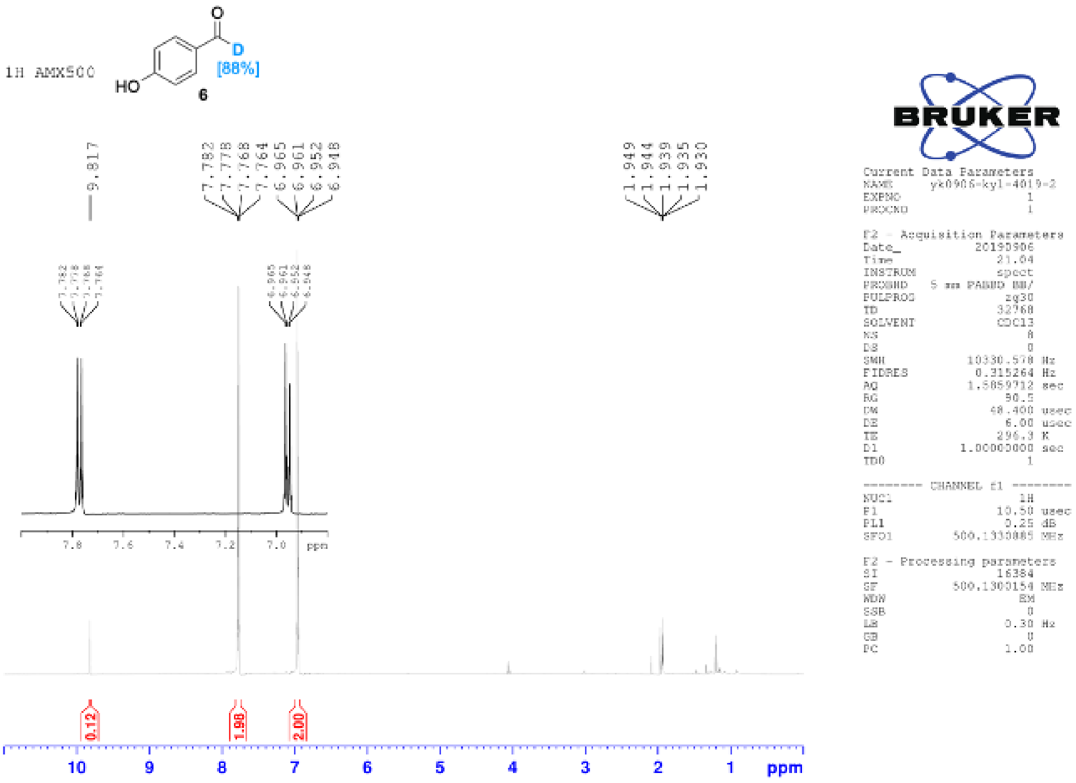

1H AMX500
O
D
[88%]
HO
6
9.817
7.782
7.778
7.768
7.764
6.965
6.961
6.952
6.948
1.949
1.944
1.939
1.935
1.930
0.12
1.98
2.00
10
9
8
7
6
5
4
3
2
1
ppm
BRUKER

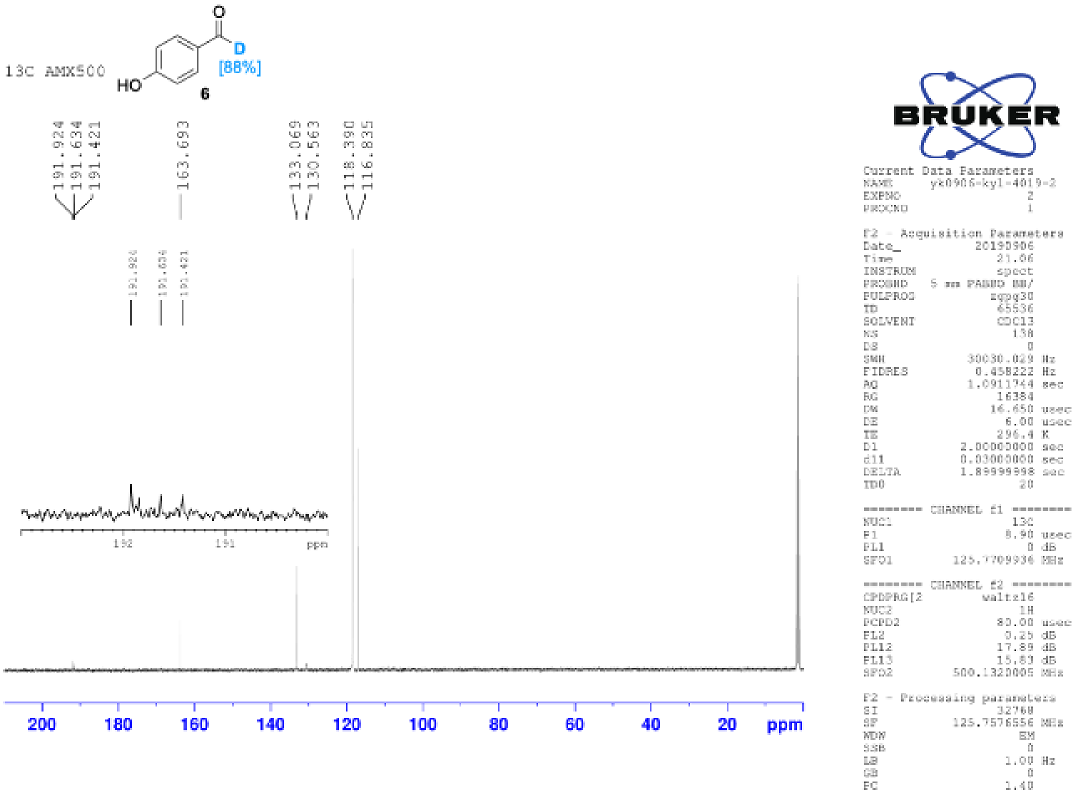

13C AMX500
O
D
[88%]
HO
6
191.924
191.634
191.421
163.693
133.069
130.563
118.390
116.835
200
180
160
140
120
100
80
60
40
20
ppm
BRUKER

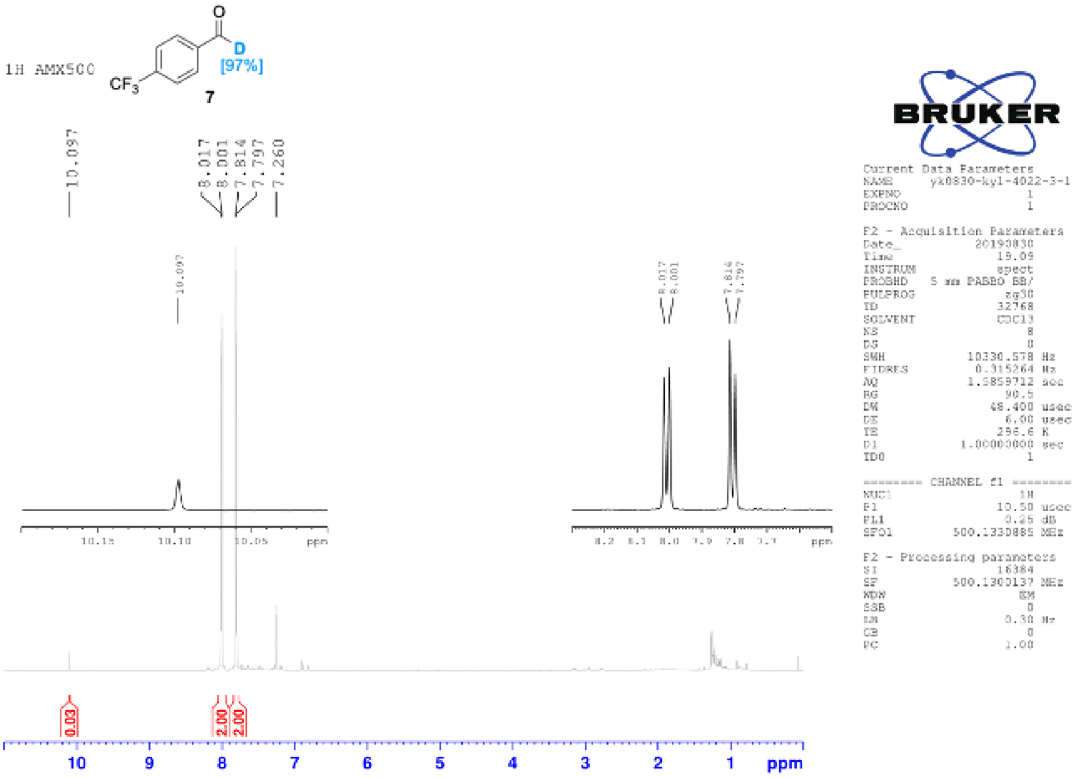
1H AMX500
O
D
[97%]
CF3
7
10.097
8.017
8.001
7.814
7.797
7.260
0.03
2.00
2.00
10 9 8 7 6 5 4 3 2 1 ppm

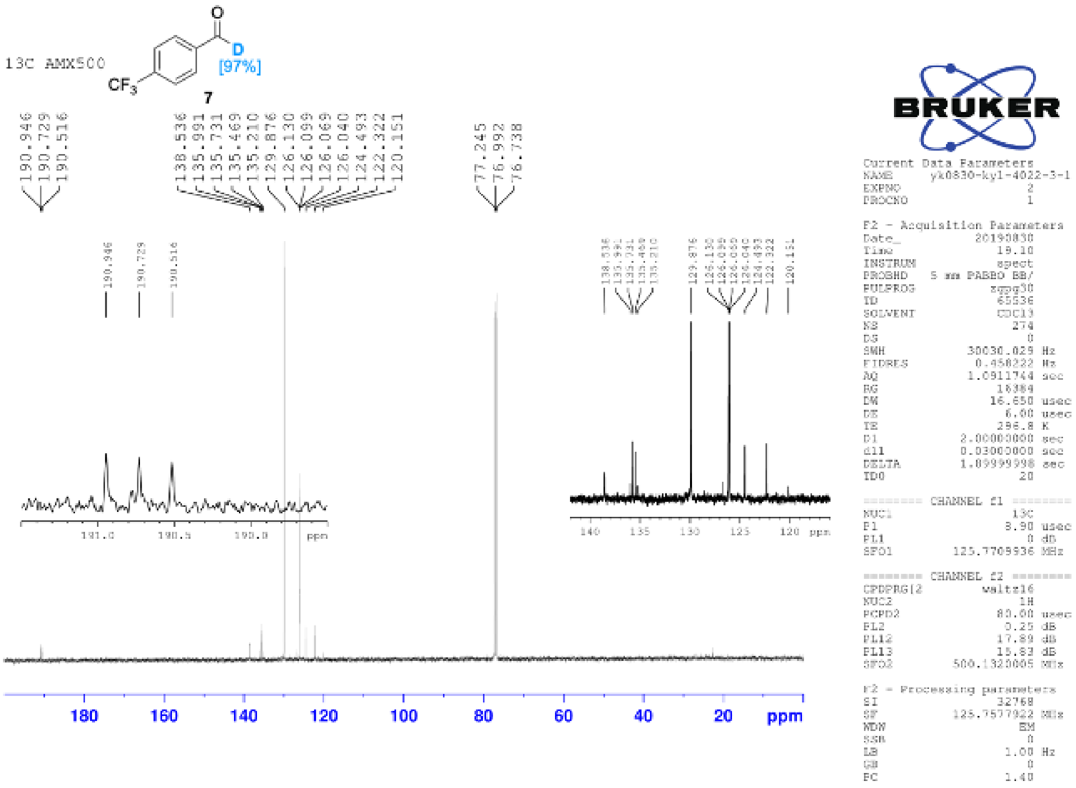
13C AMX500
O
D
[97%]
CF3
7
190.946
190.729
190.516
138.536
135.991
135.731
135.469
135.210
129.876
126.130
126.099
126.069
126.040
124.493
122.322
120.151
77.245
76.992
76.738
180 160 140 120 100 80 60 40 20 ppm

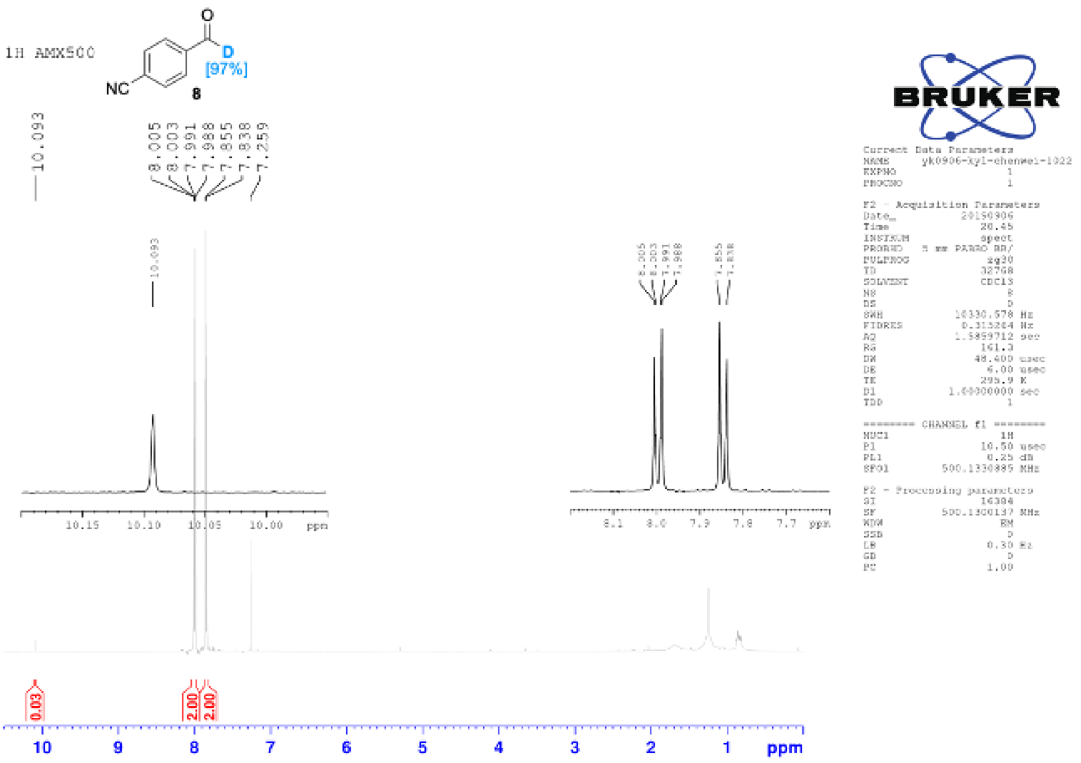
1H AMX500
NC
D
[97%]
8
10.093
8.005
8.003
7.991
7.988
7.855
7.838
7.259
BRUKER
0.03
2.00
2.00
ppm

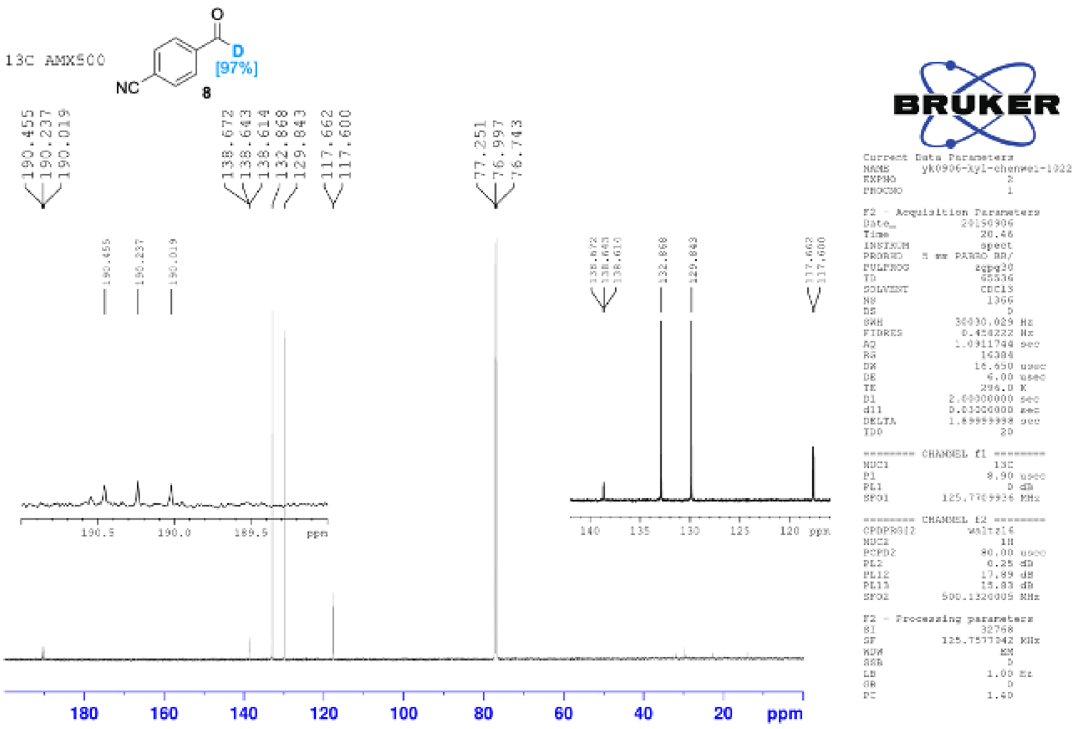
13C AMX500
NC
D
[97%]
8
190.455
190.237
190.019
138.672
138.643
138.614
132.868
129.843
117.662
117.600
77.251
76.997
76.743
BRUKER
ppm

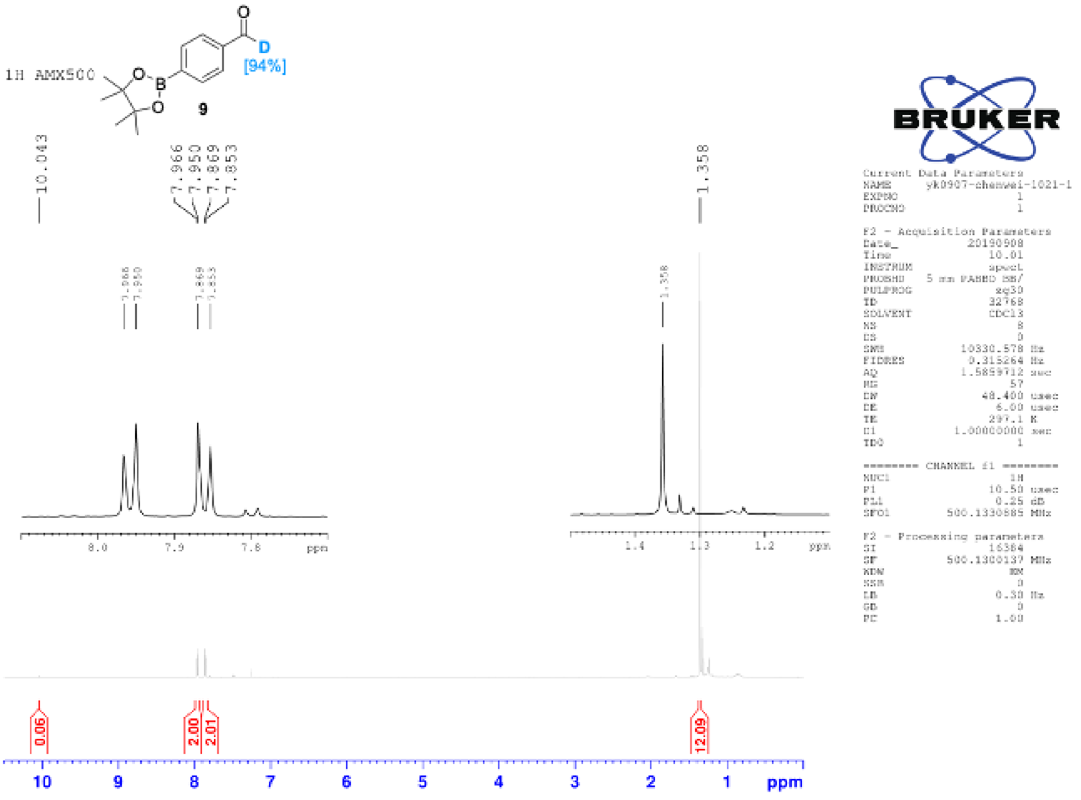
1H AMX500
D
[94%]
9
10.043
7.966
7.950
7.869
7.853
1.358
0.06
2.00
2.01
12.09
ppm

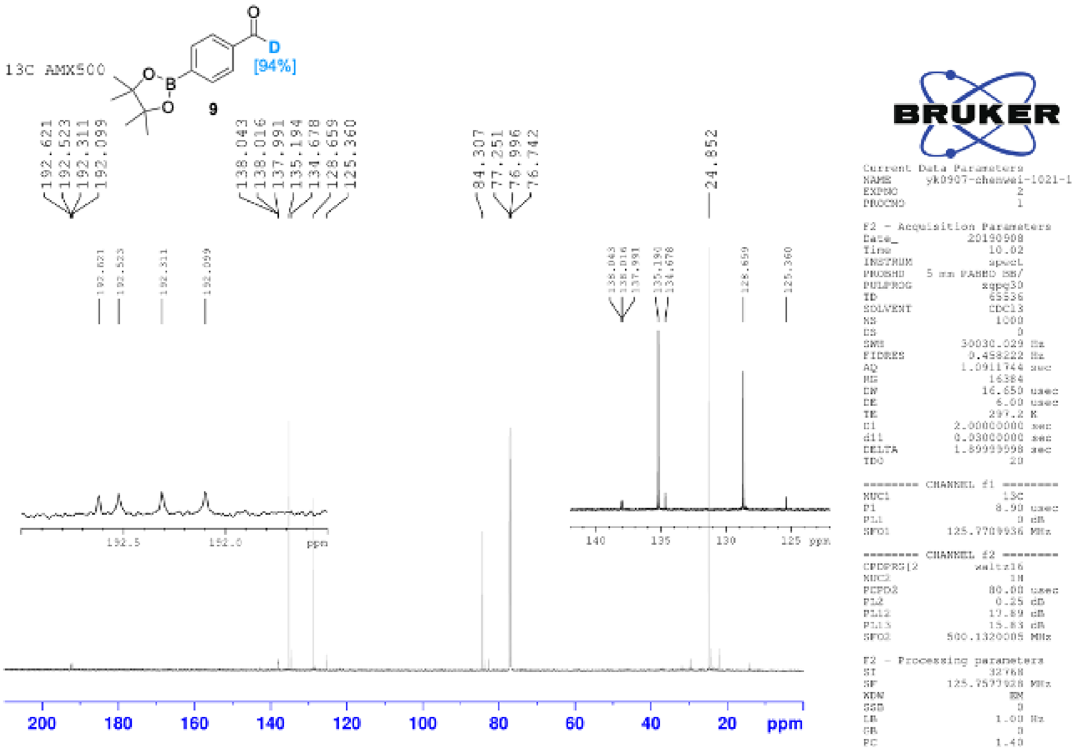
13C AMX500
D
[94%]
9
192.621
192.523
192.311
192.099
138.043
138.016
137.991
135.194
134.678
128.659
125.360
84.307
77.251
76.996
76.742
24.852
ppm

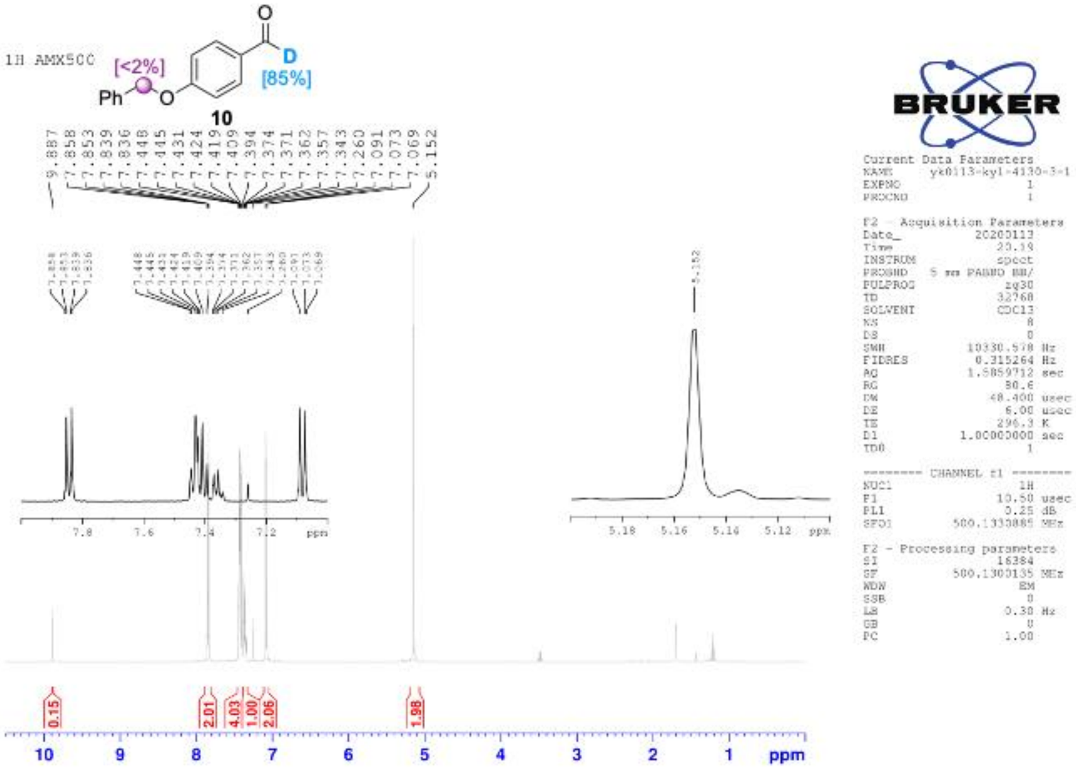
1H AMX500
[<2%]
Ph
O
D
[85%]
10
9.887 7.858 7.853 7.839 7.836 7.448 7.445 7.431 7.424 7.419 7.409 7.394 7.374 7.371 7.362 7.357 7.343 7.260 7.091 7.073 7.069 5.152
0.15 2.01 4.03 1.00 2.06 1.98
10 9 8 7 6 5 4 3 2 1 ppm
BRUKER
Current Data Parameters
NAME yk0113-kyl-4130-3-1
F2 - Acquisition Parameters
F2 - Processing parameters

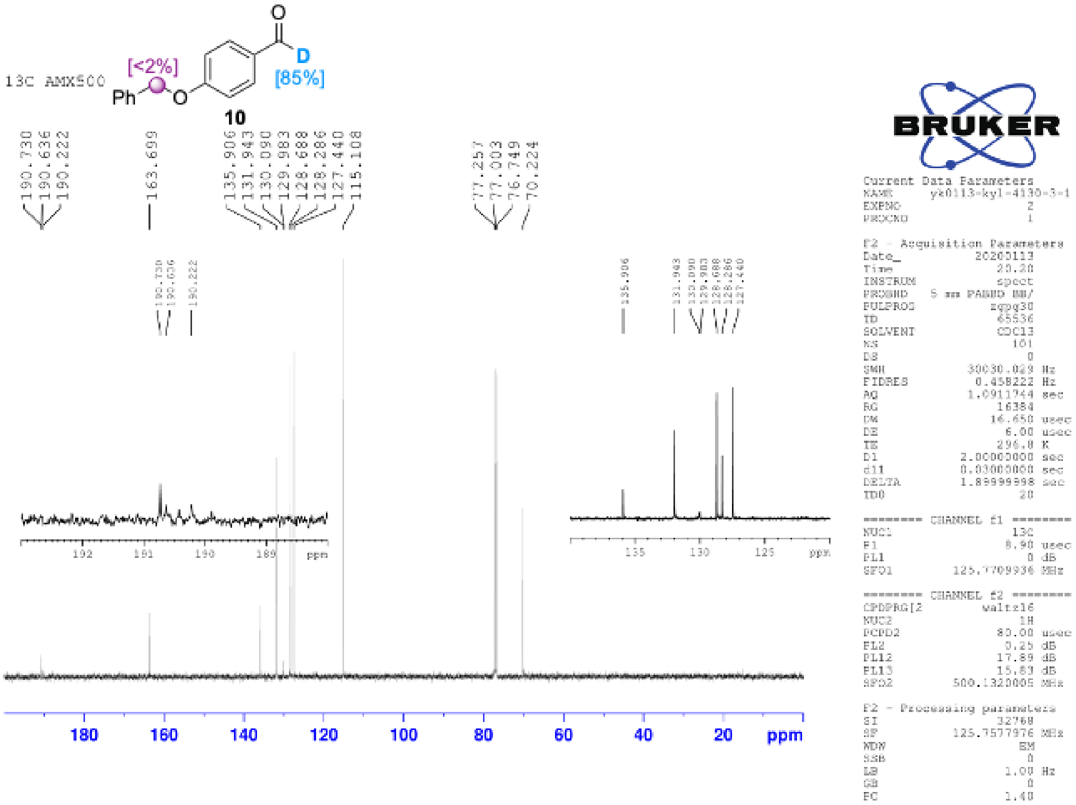
13C AMX500
[<2%]
Ph
O
D
[85%]
10
190.730 190.636 190.222 163.699 135.906 131.943 130.090 129.983 128.688 128.286 127.440 115.108 77.257 77.003 76.749 70.224
180 160 140 120 100 80 60 40 20 ppm
BRUKER
Current Data Parameters
NAME yk0113-kyl-4130-3-1
F2 - Acquisition Parameters
F2 - Processing parameters

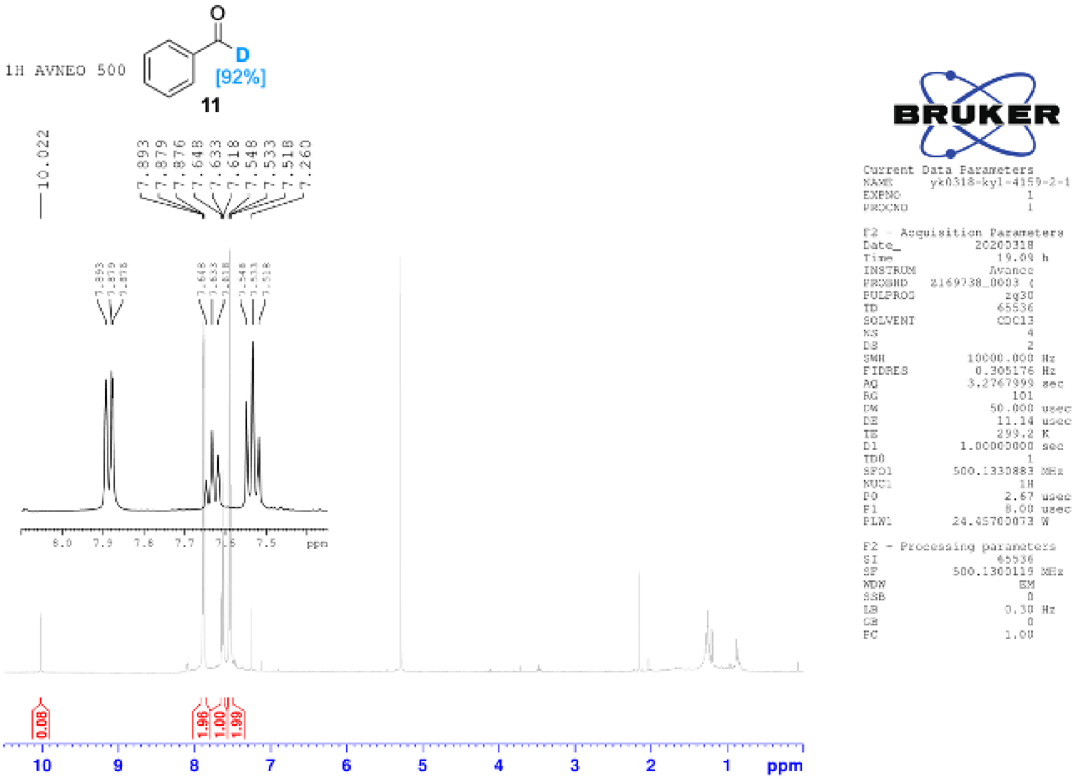
1H AVNEO 500
O
D
[92%]
11
10.022
7.893
7.879
7.876
7.648
7.633
7.618
7.548
7.533
7.518
7.260
0.08
1.96
1.00
1.99
ppm
BRUKER

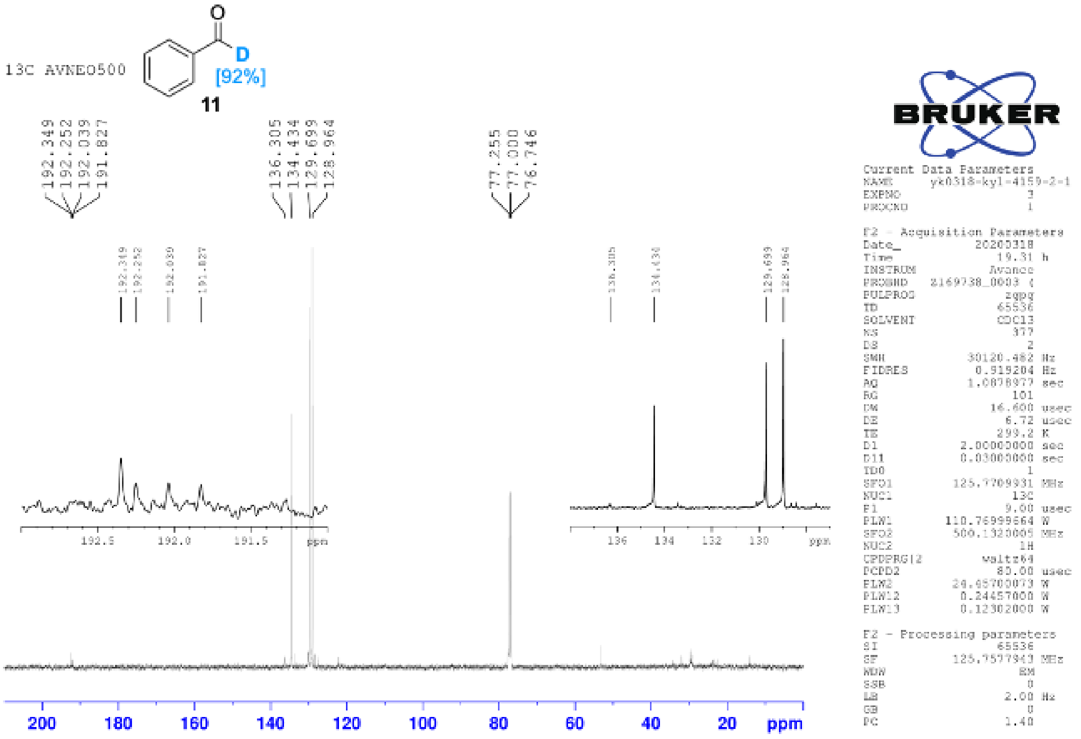
13C AVNEO500
O
D
[92%]
11
192.349
192.252
192.039
191.827
136.305
134.434
129.699
128.964
77.255
77.000
76.746
ppm
BRUKER

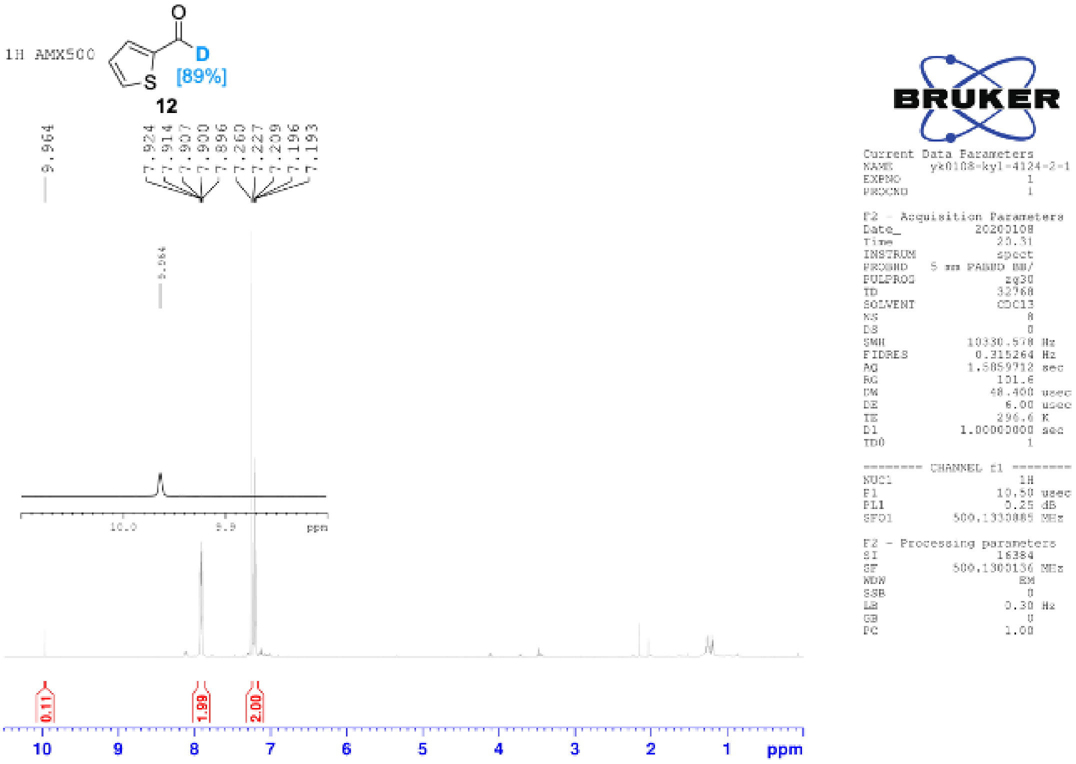
1H AMX500
D
[89%]
12
9.964
7.924
7.914
7.907
7.900
7.896
7.260
7.227
7.209
7.196
7.193
0.11
1.99
2.00
10
9
8
7
6
5
4
3
2
1
ppm
BRUKER
Current Data Parameters
NAME yk0108-kyl-4124-2-1
EXPNO 1
PROCNO 1
F2 - Acquisition Parameters
Date_ 20200108
INSTRUM spect
PULPROG zg30
TD 32768
SOLVENT CDCl3
NS 8
DS 0
SWH 10330.578 Hz
FIDRES 0.315264 Hz
AQ 1.5859712 sec
RG 101.6
DW 48.400 usec
DE 6.00 usec
TE 296.6 K
D1 1.00000000 sec
TD0 1
======== CHANNEL f1 ========
NUC1 1H
P1 10.50 usec
PL1 0.25 dB
SFO1 500.1330885 MHz
F2 - Processing parameters
SI 16384
SF 500.1300136 MHz
WDW EM
SSB 0
LB 0.30 Hz
GB 0
PC 1.00

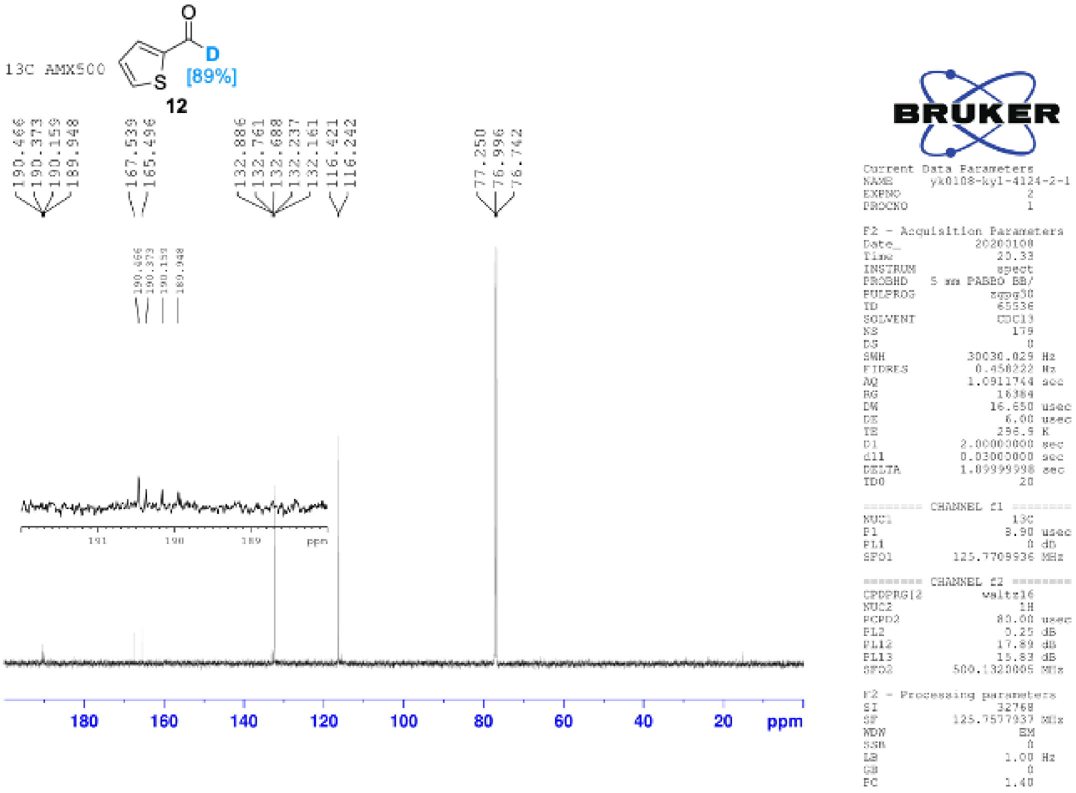
13C AMX500
D
[89%]
12
190.466
190.373
190.159
189.948
167.539
165.496
132.886
132.761
132.688
132.237
132.161
116.421
116.242
77.250
76.996
76.742
180
160
140
120
100
80
60
40
20
ppm
BRUKER
Current Data Parameters
NAME yk0108-kyl-4124-2-1
EXPNO 2
PROCNO 1
F2 - Acquisition Parameters
Date_ 20200108
INSTRUM spect
PULPROG zgpg30
TD 65536
SOLVENT CDCl3
NS 179
DS 0
SWH 30030.029 Hz
FIDRES 0.458222 Hz
AQ 1.0911744 sec
RG 16384
DW 16.650 usec
DE 6.00 usec
TE 296.9 K
D1 2.00000000 sec
d11 0.03000000 sec
DELTA 1.89999998 sec
TD0 20
======== CHANNEL f1 ========
NUC1 13C
P1 8.90 usec
PL1 0 dB
SFO1 125.7709936 MHz
======== CHANNEL f2 ========
CPDPRG2 waltz16
NUC2 1H
PCPD2 80.00 usec
PL2 0.25 dB
PL12 17.89 dB
PL13 15.83 dB
SFO2 500.1320005 MHz
F2 - Processing parameters
SI 32768
SF 125.7577937 MHz
WDW EM
SSB 0
LB 1.00 Hz
GB 0
PC 1.40

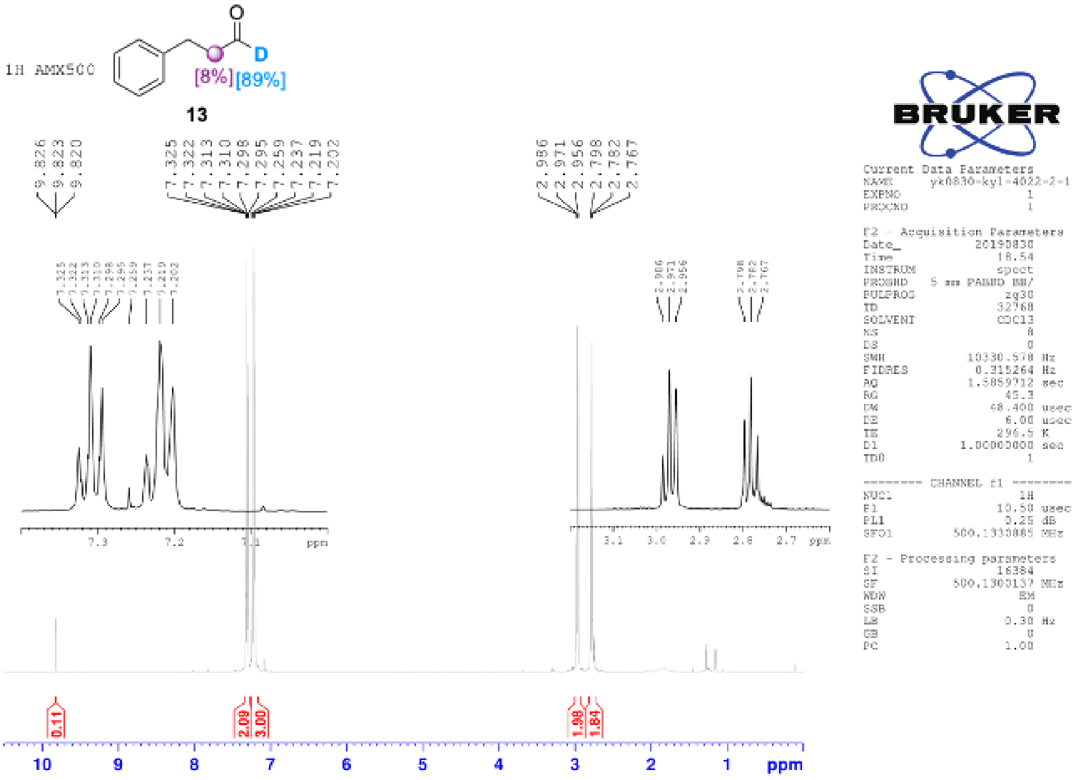
1H AMX500
[8%]
[89%]
D
O
13
9.826
9.823
9.820
7.325
7.322
7.313
7.310
7.298
7.295
7.259
7.237
7.219
7.202
2.986
2.971
2.956
2.798
2.782
2.767
0.11
2.09
3.00
1.98
1.84
ppm

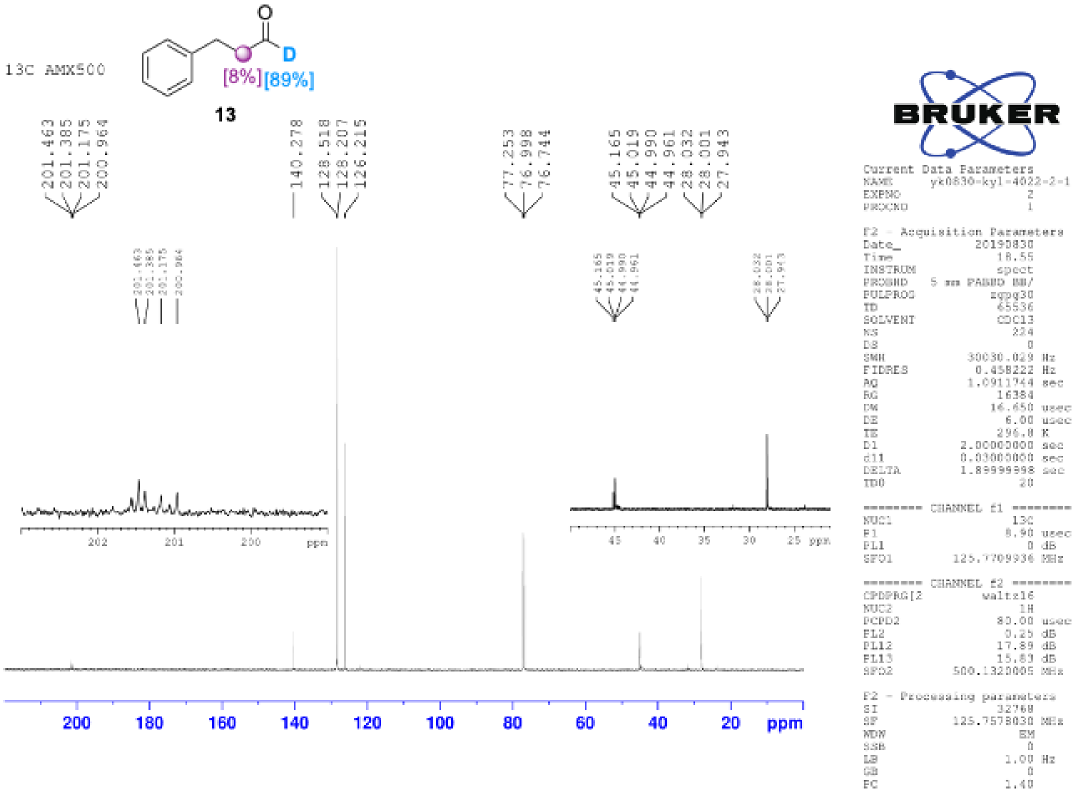
13C AMX500
[8%]
[89%]
D
O
13
201.463
201.385
201.175
200.964
140.278
128.518
128.207
126.215
77.253
76.998
76.744
45.165
45.019
44.990
44.961
28.032
28.001
27.943
ppm

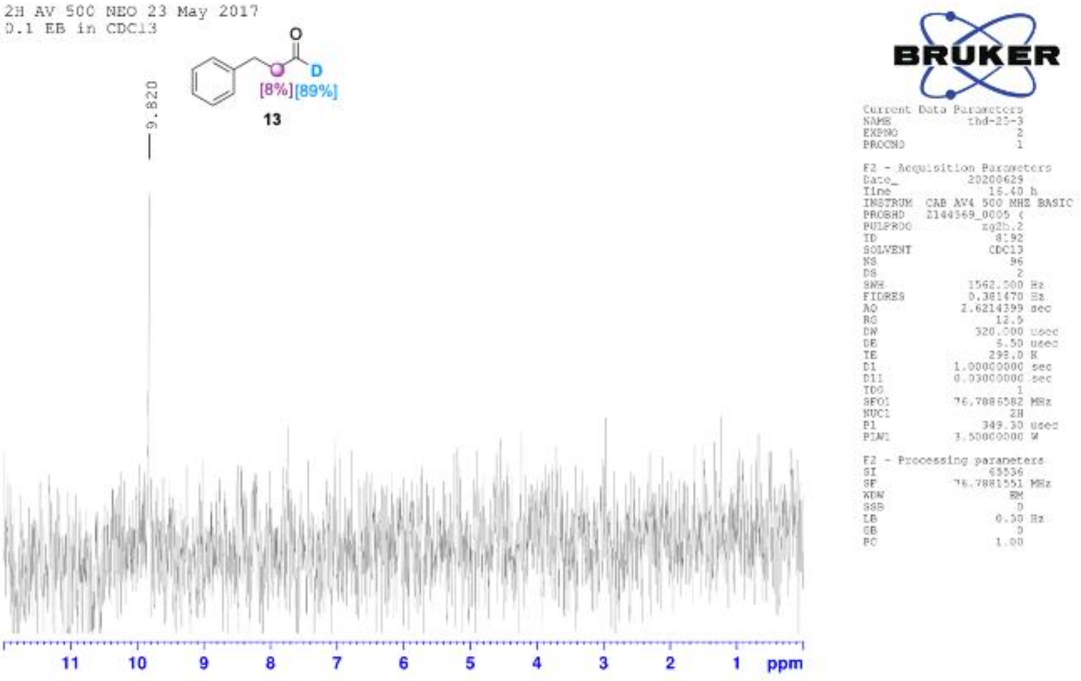
2H AV 500 NEO 23 May 2017
0.1 EB in CDCl3
[8%][89%]
D
13
9.820
Current Data Parameters
F2 - Acquisition Parameters
F2 - Processing parameters
ppm

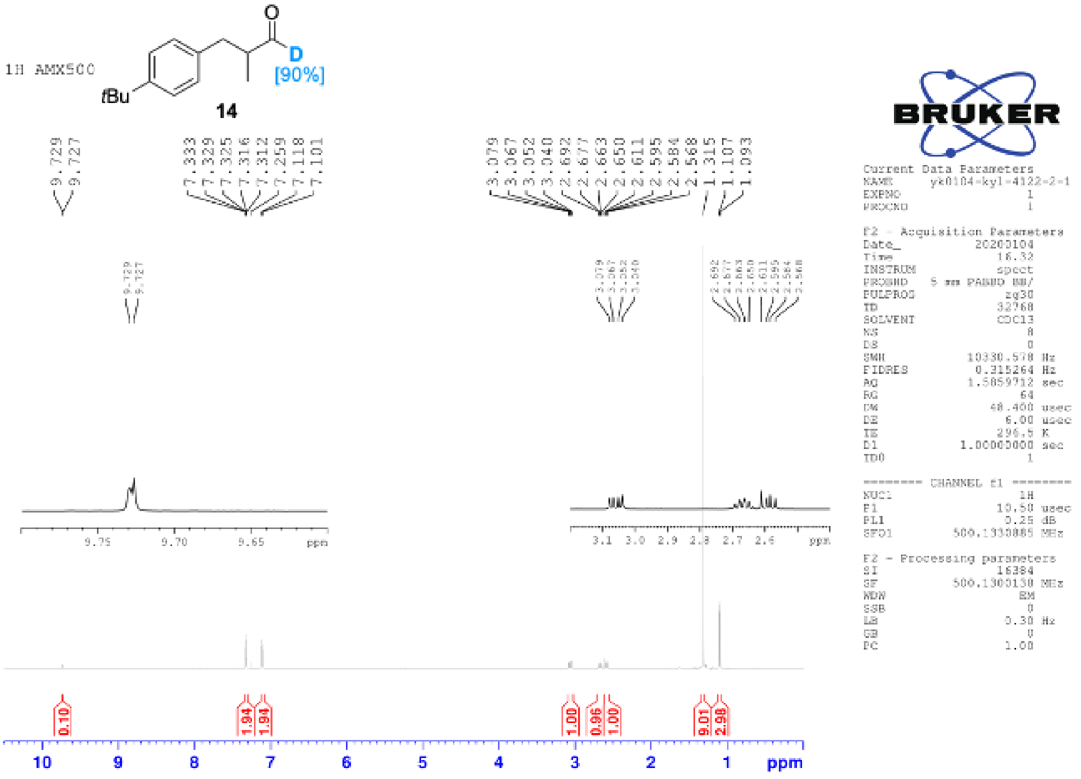
1H AMX500
tBu
D
[90%]
14
Current Data Parameters
F2 - Acquisition Parameters
F2 - Processing parameters
ppm

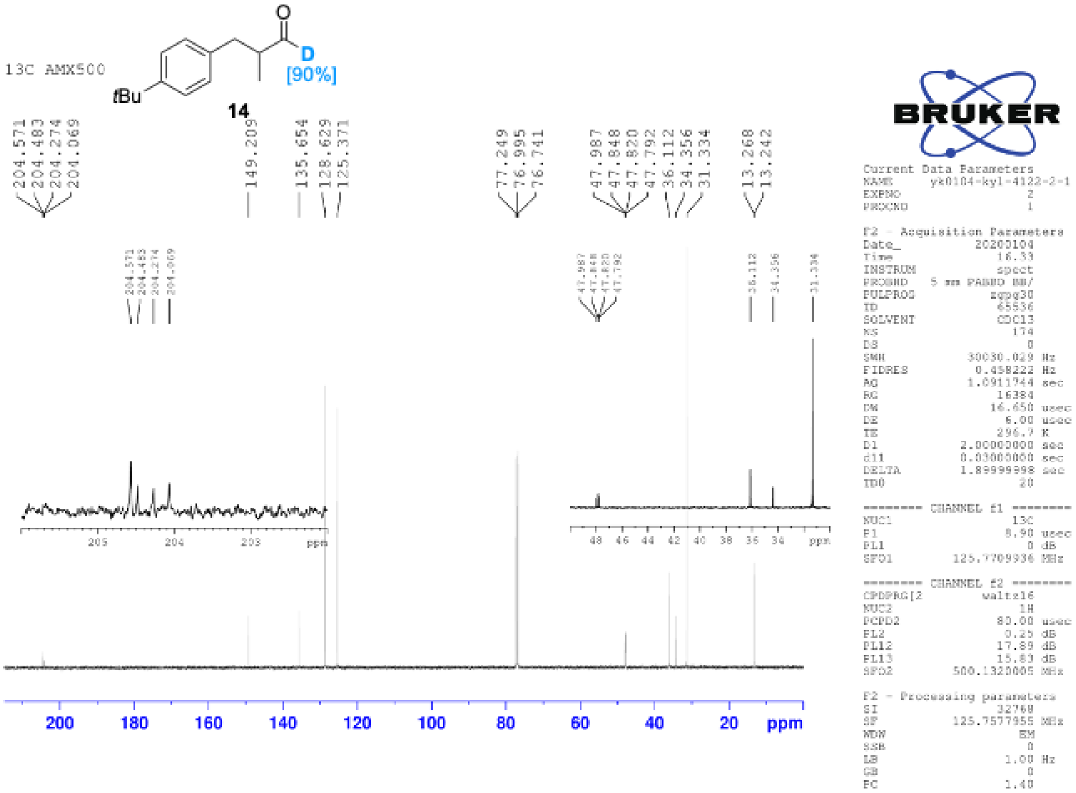
13C AMX500
tBu
14
D
[90%]
204.571
204.483
204.274
204.069
149.209
135.654
128.629
125.371
77.249
76.995
76.741
47.987
47.848
47.820
47.792
36.112
34.356
31.334
13.268
13.242
200
180
160
140
120
100
80
60
40
20
ppm
Current Data Parameters
NAME yk0104-ky1-4122-2-1
EXPNO 2
PROCNO 1
F2 - Acquisition Parameters
Date_ 20200104
Time 16.33
INSTRUM spect
PROBHD 5 mm PABBO BB/
PULPROG zgpg30
TD 65536
SOLVENT CDCl3
NS 174
DS 0
SWH 30030.029 Hz
FIDRES 0.458222 Hz
AQ 1.0911744 sec
RG 16384
DW 16.650 usec
DE 6.00 usec
TE 296.7 K
D1 2.00000000 sec
d11 0.03000000 sec
DELTA 1.89999998 sec
TD0 20
======== CHANNEL f1 ========
NUC1 13C
P1 8.90 usec
PL1 0 dB
SFO1 125.7709936 MHz
======== CHANNEL f2 ========
CPDPRG[2 waltz16
NUC2 1H
PCPD2 80.00 usec
PL2 0.25 dB
PL12 17.89 dB
PL13 15.83 dB
SFO2 500.1320005 MHz
F2 - Processing parameters
SI 32768
SF 125.7577955 MHz
WDW EM
SSB 0
LB 1.00 Hz
GB 0
PC 1.40

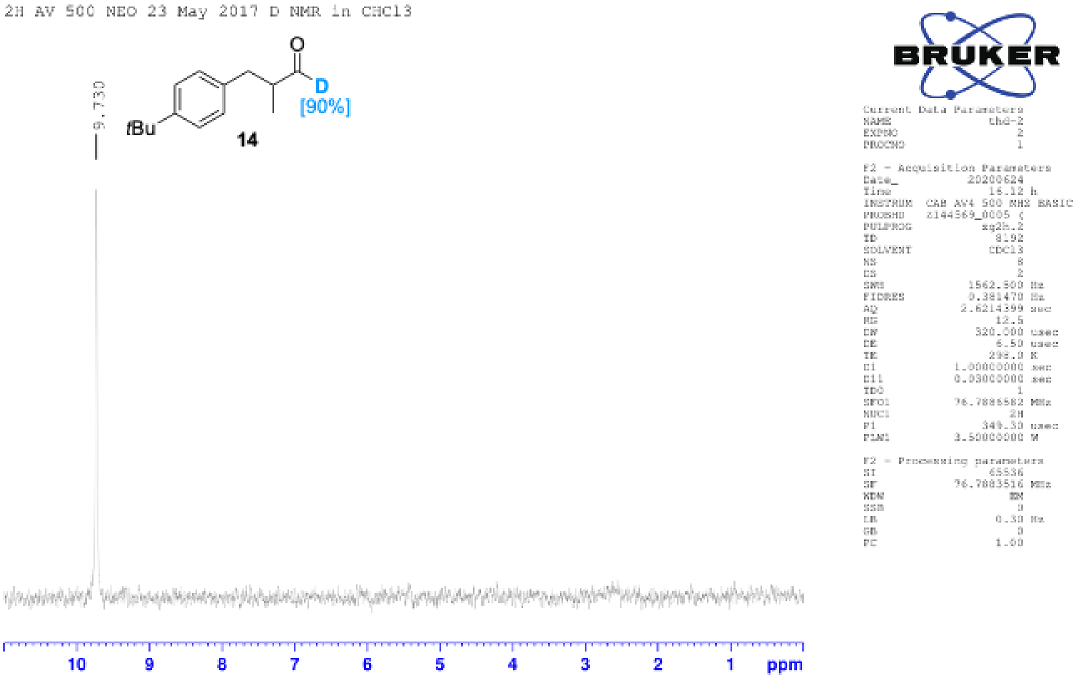
2H AV 500 NEO 23 May 2017 D NMR in CHCl3
tBu
14
D
[90%]
9.730
10
9
8
7
6
5
4
3
2
1
ppm
Current Data Parameters
NAME thd-2
EXPNO 2
PROCNO 1
F2 - Acquisition Parameters
Date_ 20200624
Time 16.12 h
INSTRUM CAB AV4 500 MHZ BASIC
PROBHD Z144569_0005 (
PULPROG zg2h.2
TD 8192
SOLVENT CDCl3
NS 8
DS 2
SWH 1562.500 Hz
FIDRES 0.381470 Hz
AQ 2.6214399 sec
RG 12.5
DW 320.000 usec
DE 6.50 usec
TE 298.0 K
D1 1.00000000 sec
D11 0.03000000 sec
TD0 1
SFO1 76.7886582 MHz
NUC1 2H
P1 349.30 usec
PLW1 3.50000000 W
F2 - Processing parameters
SI 65536
SF 76.7883516 MHz
WDW EM
SSB 0
LB 0.30 Hz
GB 0
PC 1.00

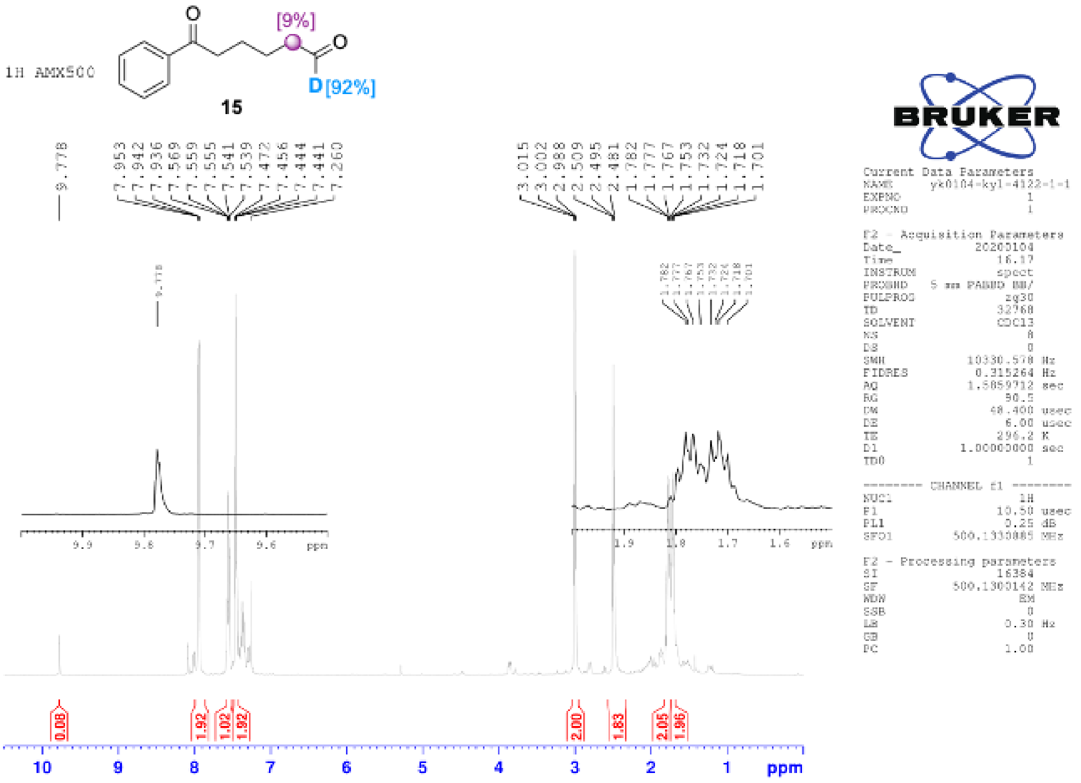
1H AMX500
[9%]
D[92%]
15
9.778
7.953
7.942
7.936
7.569
7.559
7.555
7.541
7.539
7.472
7.456
7.444
7.441
7.260
3.015
3.002
2.988
2.509
2.495
2.481
1.782
1.777
1.767
1.753
1.732
1.724
1.718
1.701
BRUKER
0.08
1.92
1.02
1.92
2.00
1.83
2.05
1.96
ppm

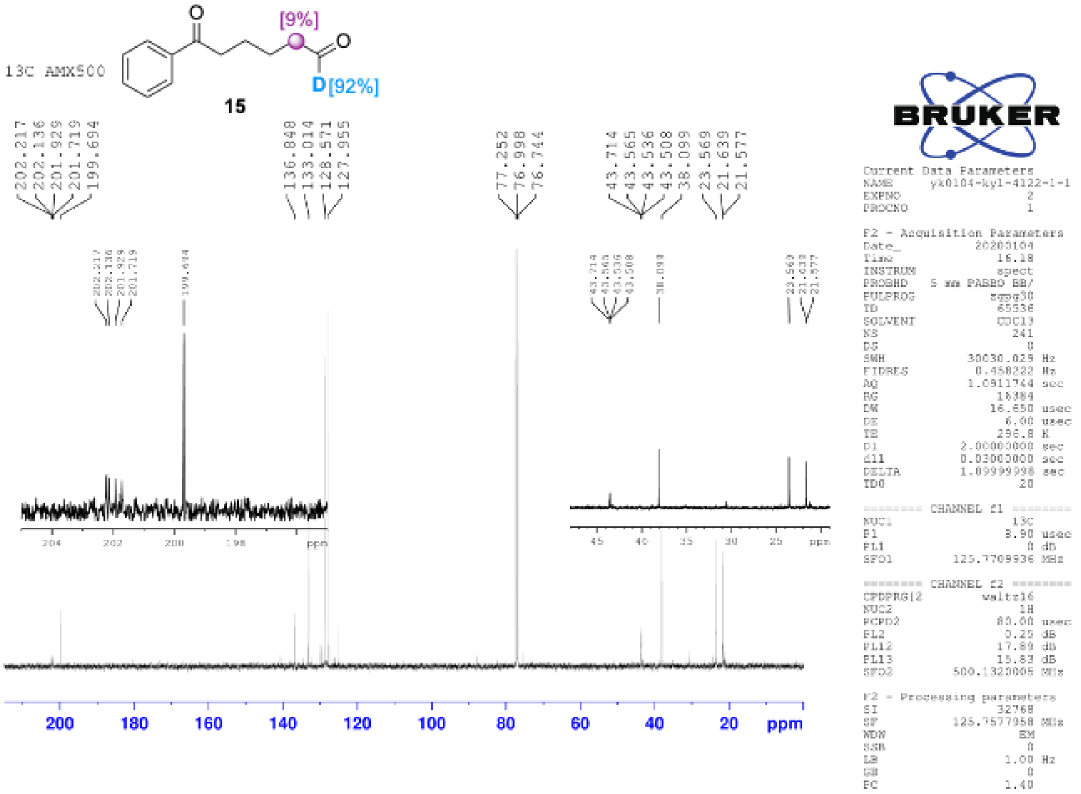
13C AMX500
[9%]
D[92%]
15
202.217
202.136
201.929
201.719
199.694
136.848
133.014
128.571
127.955
77.252
76.998
76.744
43.714
43.565
43.536
43.508
38.099
23.569
21.639
21.577
BRUKER
ppm

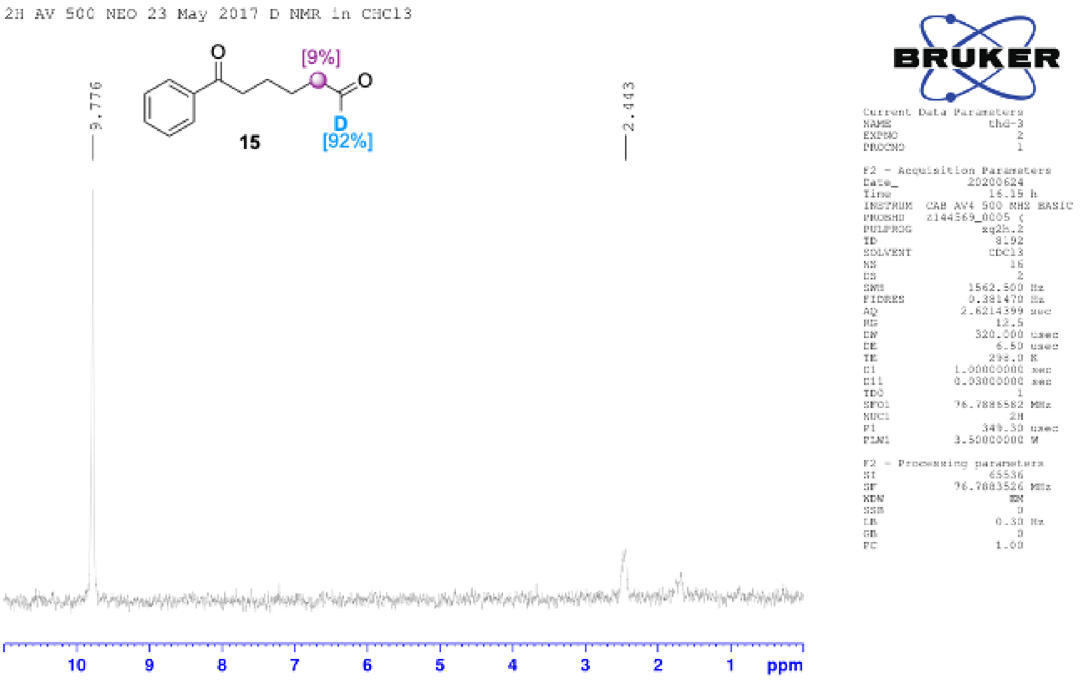
2H AV 500 NEO 23 May 2017 D NMR in CHCl3
[9%]
15
D
[92%]
9.776
2.443
ppm

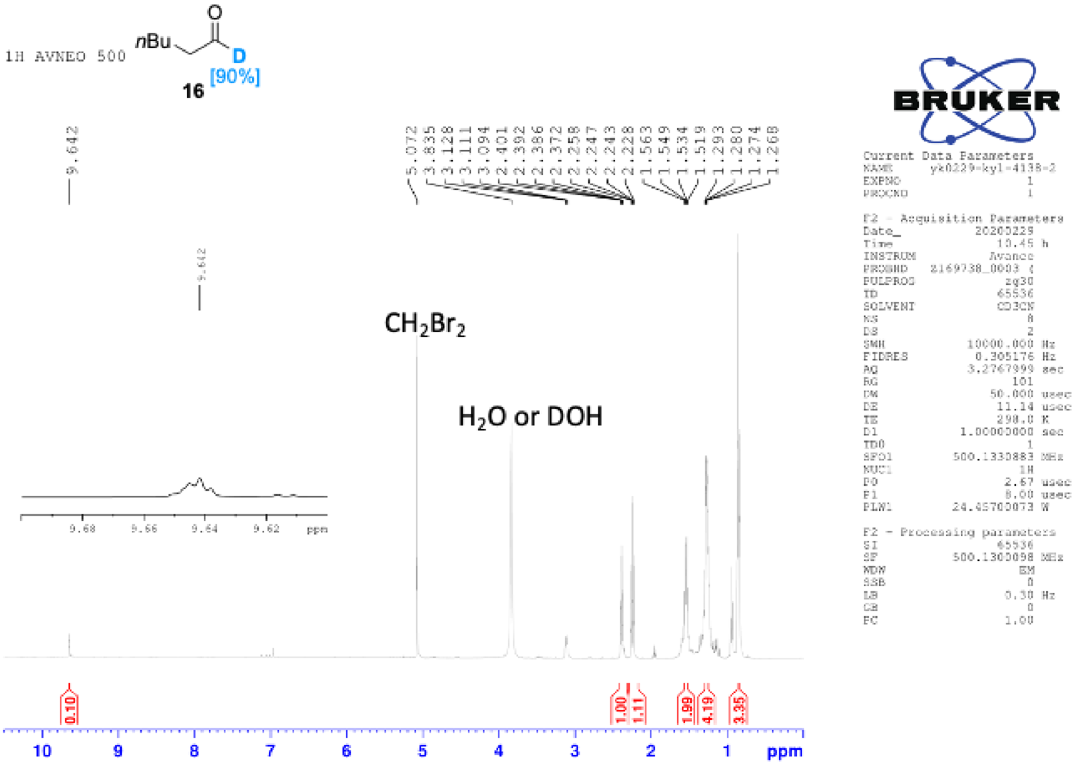
1H AVNEO 500
nBu
D
[90%]
16
9.642
CH2Br2
H2O or DOH
0.10
1.00
1.11
1.99
4.19
3.35
ppm

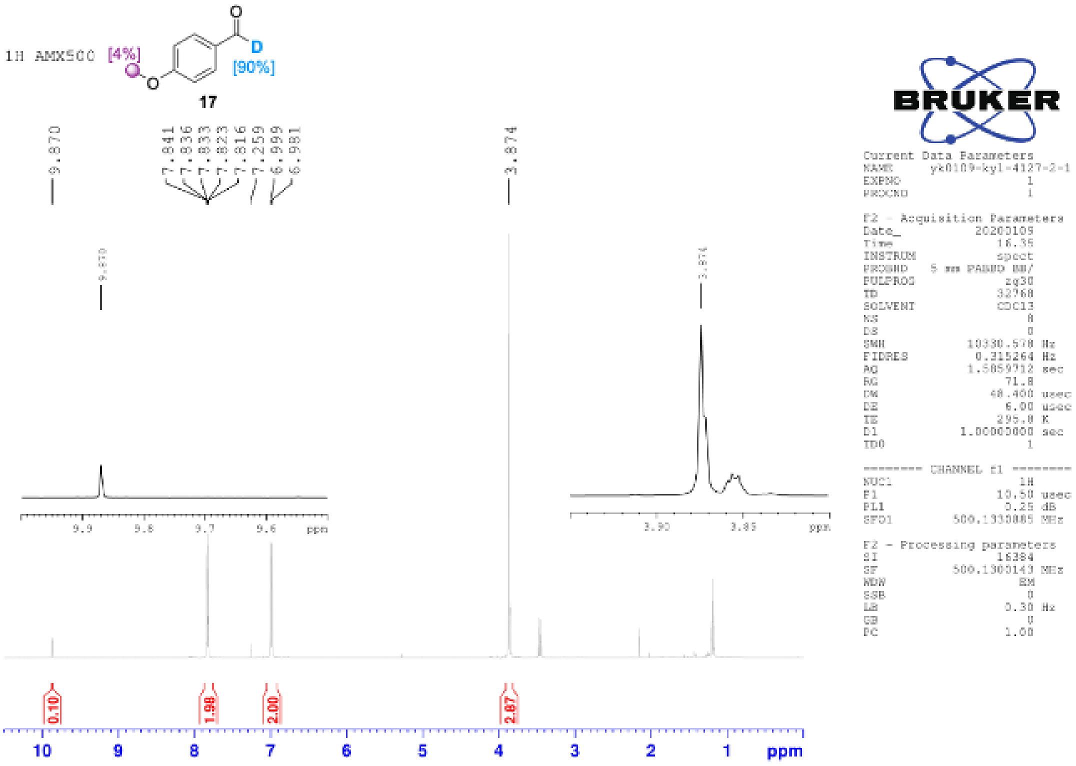
1H AMX500
[4%]
D
[90%]
17
9.870
7.841
7.836
7.833
7.823
7.816
7.259
6.999
6.981
3.874
0.10
1.98
2.00
2.87
ppm
Current Data Parameters
NAME yk0109-kyl-4127-2-1
EXPNO 1
PROCNO 1
F2 - Acquisition Parameters
Date_ 20200109
Time 16.35
INSTRUM spect
PROBHD 5 mm PABBO BB/
PULPROG zg30
TD 32768
SOLVENT CDCl3
NS 8
DS 0
SWH 10330.578 Hz
FIDRES 0.315264 Hz
AQ 1.5859712 sec
RG 71.8
DW 48.400 usec
DE 6.00 usec
TE 295.8 K
D1 1.00000000 sec
TD0 1
======== CHANNEL f1 ========
NUC1 1H
P1 10.50 usec
PL1 0.25 dB
SFO1 500.1330885 MHz
F2 - Processing parameters
SI 16384
SF 500.1300143 MHz
WDW EM
SSB 0
LB 0.30 Hz
GB 0
PC 1.00

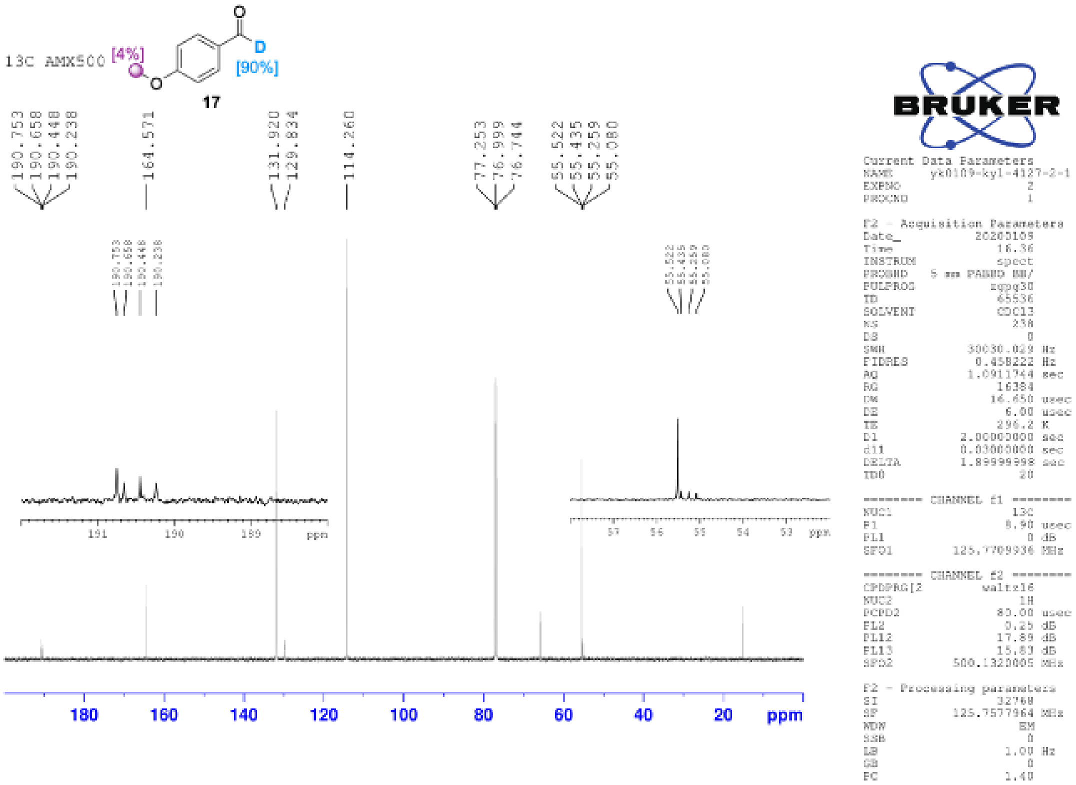
13C AMX500
[4%]
D
[90%]
17
190.753
190.658
190.448
190.238
164.571
131.920
129.834
114.260
77.253
76.999
76.744
55.522
55.435
55.259
55.080
ppm
Current Data Parameters
NAME yk0109-kyl-4127-2-1
EXPNO 2
PROCNO 1
F2 - Acquisition Parameters
Date_ 20200109
Time 16.36
INSTRUM spect
PROBHD 5 mm PABBO BB/
PULPROG zgpg30
TD 65536
SOLVENT CDCl3
NS 238
DS 0
SWH 30030.029 Hz
FIDRES 0.458222 Hz
AQ 1.0911744 sec
RG 16384
DW 16.650 usec
DE 6.00 usec
TE 296.2 K
D1 2.00000000 sec
d11 0.03000000 sec
DELTA 1.89999998 sec
TD0 20
======== CHANNEL f1 ========
NUC1 13C
P1 8.90 usec
PL1 0 dB
SFO1 125.7709936 MHz
======== CHANNEL f2 ========
CPDPRG[2 waltz16
NUC2 1H
PCPD2 80.00 usec
PL2 0.25 dB
PL12 17.89 dB
PL13 15.83 dB
SFO2 500.1320005 MHz
F2 - Processing parameters
SI 32768
SF 125.7577964 MHz
WDW EM
SSB 0
LB 1.00 Hz
GB 0
PC 1.40

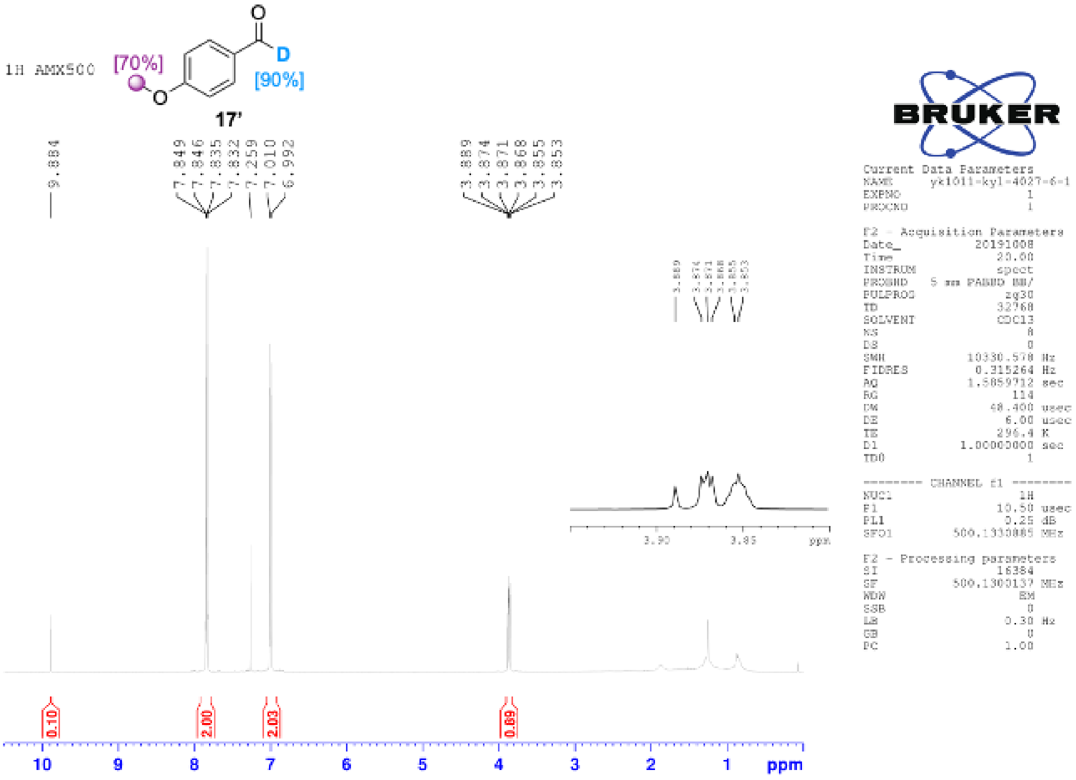
1H AMX500
[70%]
D
[90%]
17'
9.884
7.849
7.846
7.835
7.832
7.259
7.010
6.992
3.889
3.874
3.871
3.868
3.855
3.853
0.10
2.00
2.03
0.89
10 9 8 7 6 5 4 3 2 1 ppm
3.90 3.85 ppm
BRUKER
Current Data Parameters
NAME yk1011-kyl-4027-6-1
EXPNO 1
PROCNO 1
F2 - Acquisition Parameters
Date_ 20191008
INSTRUM spect
PULPROG zg30
TD 32768
SOLVENT CDCl3
NS 8
DS 0
SWH 10330.578 Hz
FIDRES 0.315264 Hz
AQ 1.5859712 sec
RG 114
DW 48.400 usec
DE 6.00 usec
TE 296.4 K
D1 1.00000000 sec
TD0 1
======== CHANNEL f1 ========
NUC1 1H
P1 10.50 usec
PL1 0.25 dB
SFO1 500.1330885 MHz
F2 - Processing parameters
SI 16384
SF 500.1300137 MHz
WDW EM
SSB 0
LB 0.30 Hz
GB 0
PC 1.00

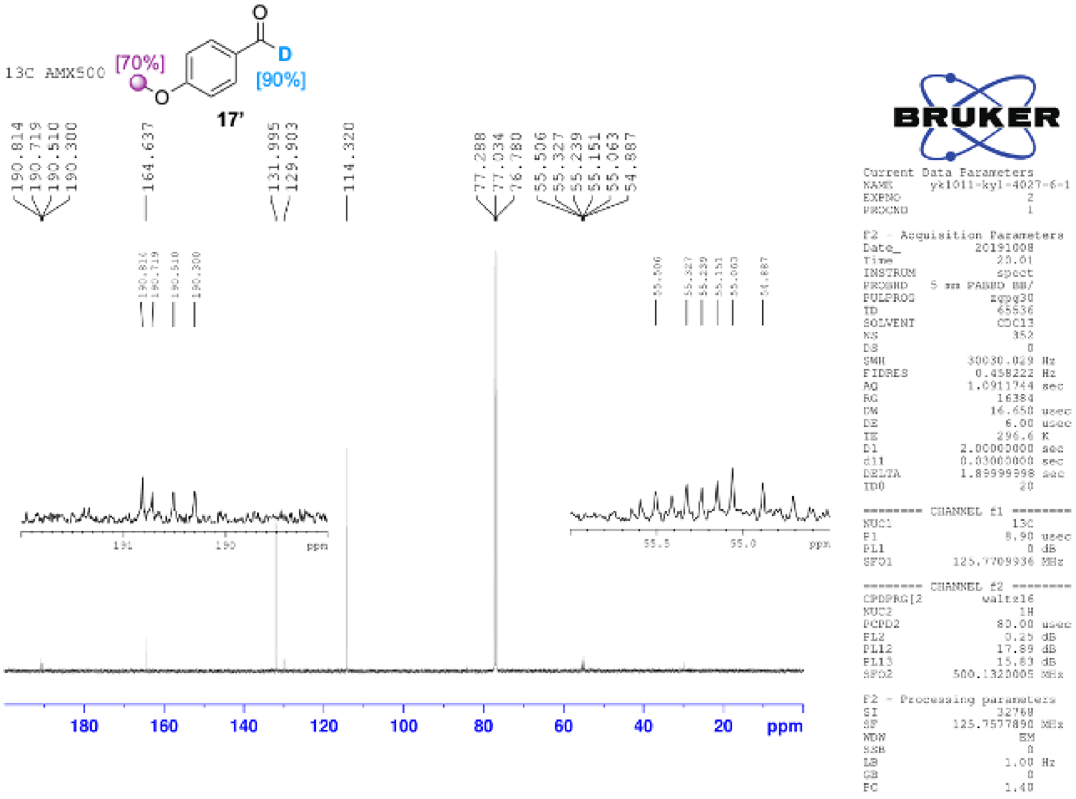
13C AMX500
[70%]
D
[90%]
17'
190.814
190.719
190.510
190.300
164.637
131.995
129.903
114.320
77.288
77.034
76.780
55.506
55.327
55.239
55.151
55.063
54.887
191 190 ppm
55.5 55.0 ppm
180 160 140 120 100 80 60 40 20 ppm
BRUKER
Current Data Parameters
NAME yk1011-kyl-4027-6-1
EXPNO 2
PROCNO 1
F2 - Acquisition Parameters
Date_ 20191008
INSTRUM spect
PULPROG zgpg30
TD 65536
SOLVENT CDCl3
NS 352
DS 0
SWH 30030.029 Hz
FIDRES 0.458222 Hz
AQ 1.0911744 sec
RG 16384
DW 16.650 usec
DE 6.00 usec
TE 296.6 K
D1 2.00000000 sec
d11 0.03000000 sec
DELTA 1.89999998 sec
TD0 20
======== CHANNEL f1 ========
NUC1 13C
P1 8.90 usec
PL1 0 dB
SFO1 125.7709936 MHz
======== CHANNEL f2 ========
CPDPRG[2 waltz16
NUC2 1H
PCPD2 80.00 usec
PL2 0.25 dB
PL12 17.89 dB
PL13 15.83 dB
SFO2 500.1320005 MHz
F2 - Processing parameters
SI 32768
SF 125.7577890 MHz
WDW EM
SSB 0
LB 1.00 Hz
GB 0
PC 1.40

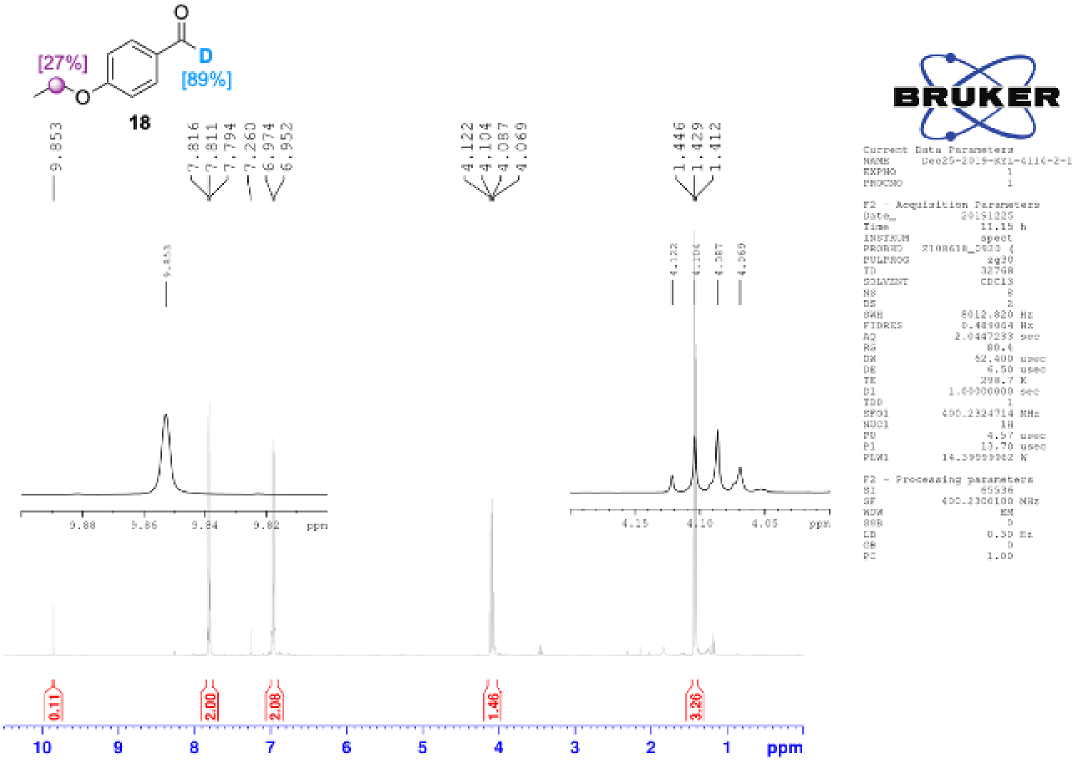
[27%]
D
[89%]
18
9.853
7.816
7.811
7.794
7.260
6.974
6.952
4.122
4.104
4.087
4.069
1.446
1.429
1.412
0.11
2.00
2.08
1.46
3.26
ppm
BRUKER

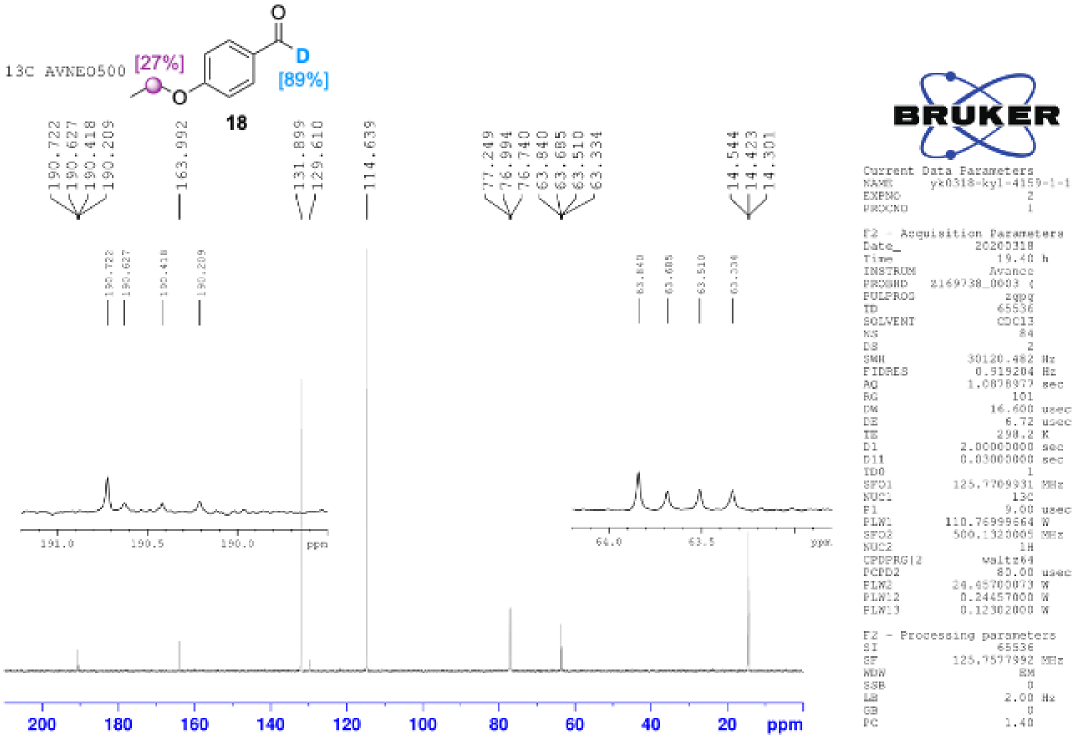
13C AVNEO500
[27%]
D
[89%]
18
190.722
190.627
190.418
190.209
163.992
131.899
129.610
114.639
77.249
76.994
76.740
63.840
63.685
63.510
63.334
14.544
14.423
14.301
ppm
BRUKER

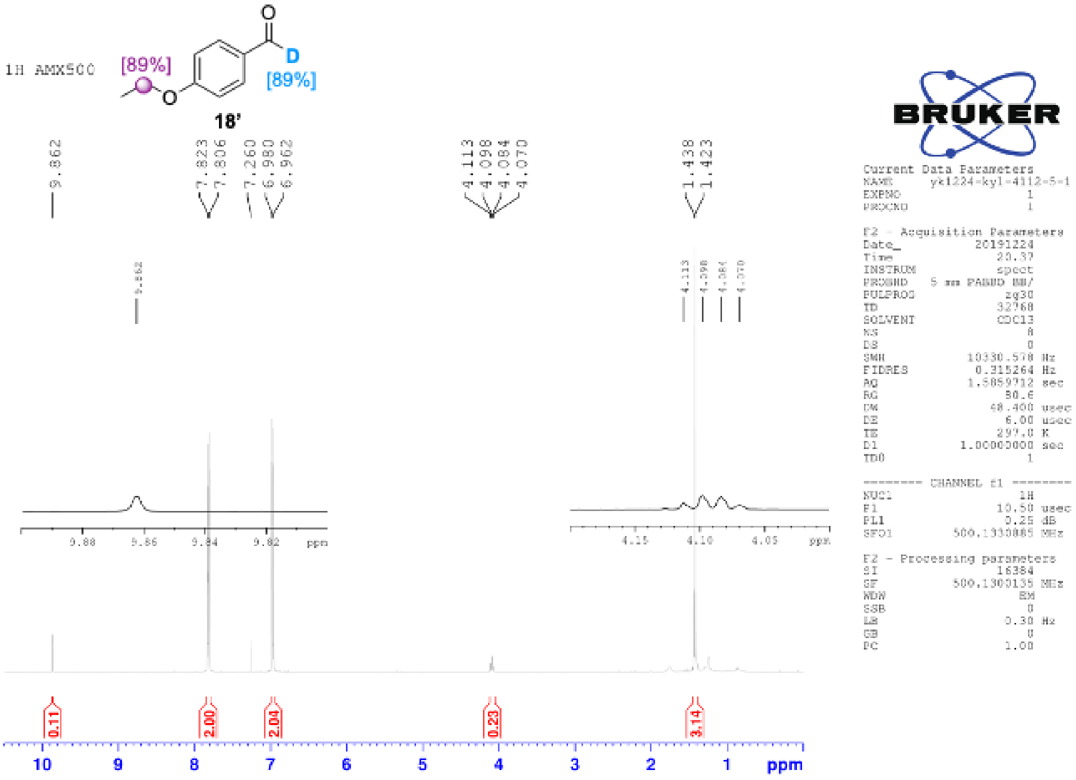
1H AMX500
[89%]
D
[89%]
18'
BRUKER

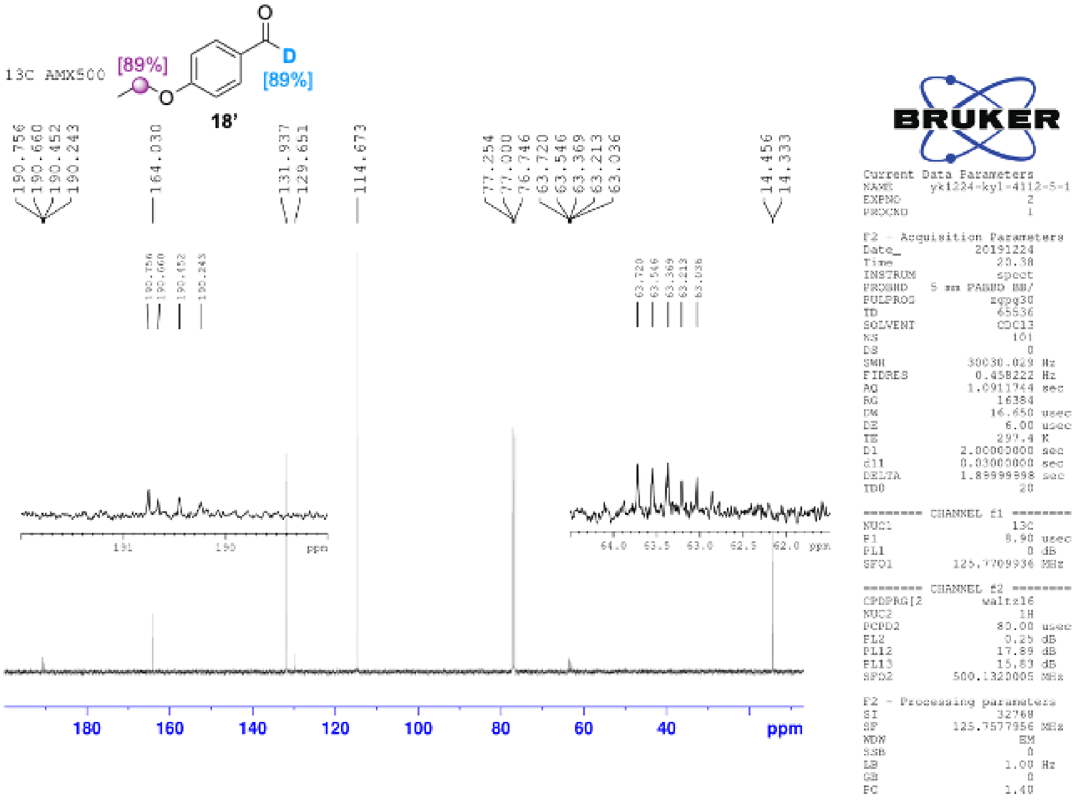
13C AMX500
[89%]
D
[89%]
18'
BRUKER

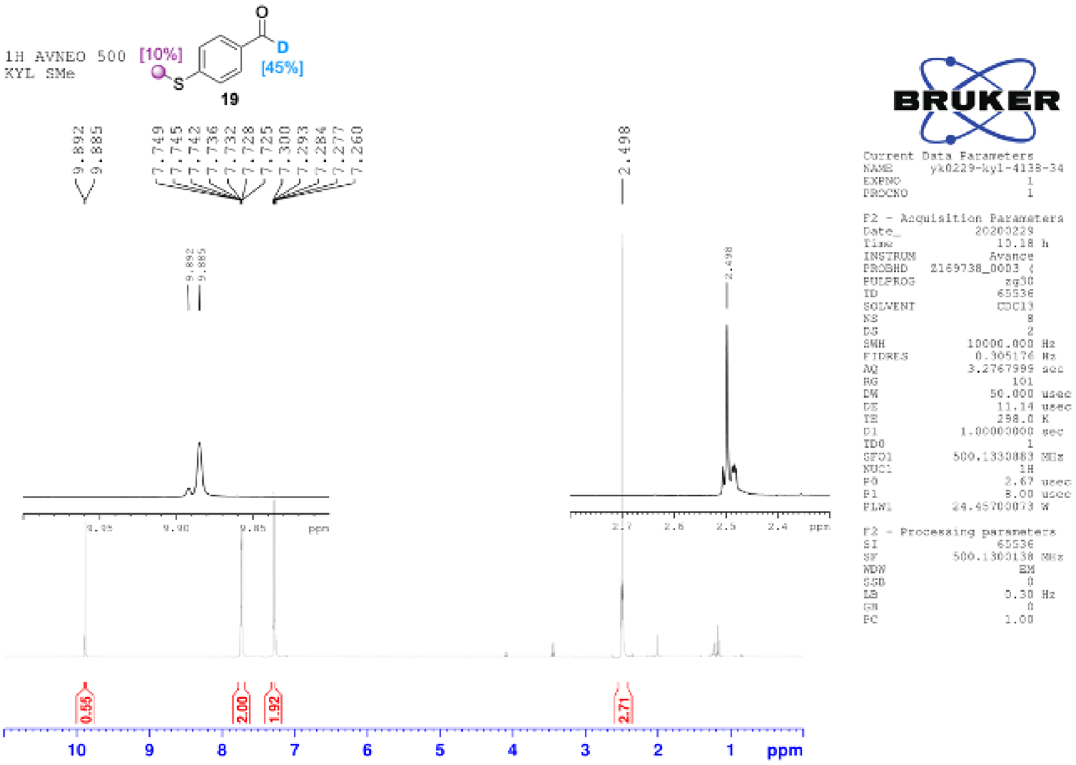
1H AVNEO 500
KYL SMe
[10%]
[45%]
D
O
S
19
9.892
9.885
7.749
7.745
7.742
7.736
7.732
7.728
7.725
7.300
7.293
7.284
7.277
7.260
2.498
0.55
2.00
1.92
2.71
10 9 8 7 6 5 4 3 2 1 ppm
Current Data Parameters
NAME yk0229-kyl-4138-34
EXPNO 1
PROCNO 1
F2 - Acquisition Parameters
Date_ 20200229
Time 10.18 h
INSTRUM Avance
PROBHD 2169738_0003 (
PULPROG zg30
TD 65536
SOLVENT CDCl3
NS 8
DS 2
SWH 10000.000 Hz
FIDRES 0.305176 Hz
AQ 3.2767999 sec
RG 101
DW 50.000 usec
DE 11.14 usec
TE 298.0 K
D1 1.00000000 sec
TD0 1
SFO1 500.1330883 MHz
NUC1 1H
P0 2.67 usec
P1 8.00 usec
PLW1 24.45700073 W
F2 - Processing parameters
SI 65536
SF 500.1300138 MHz
WDW EM
SSB 0
LB 0.30 Hz
GB 0
PC 1.00

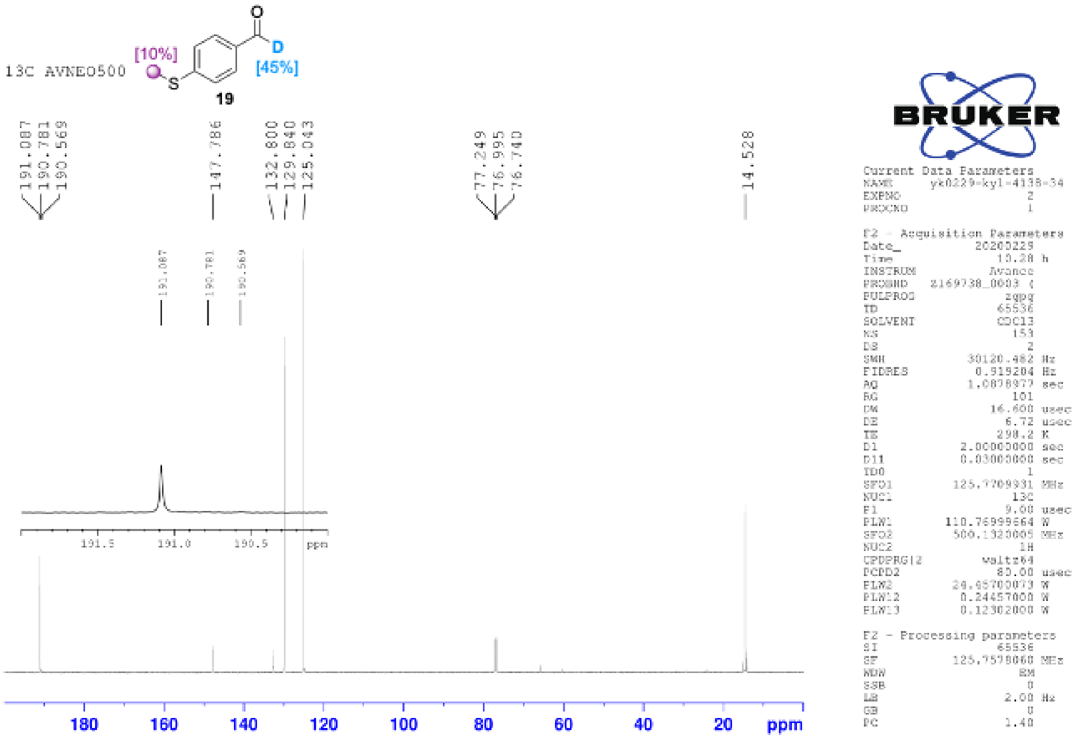
13C AVNEO500
[10%]
[45%]
D
O
S
19
191.087
190.781
190.569
147.786
132.800
129.840
125.043
77.249
76.995
76.740
14.528
191.5 191.0 190.5 ppm
180 160 140 120 100 80 60 40 20 ppm
Current Data Parameters
NAME yk0229-kyl-4138-34
EXPNO 2
PROCNO 1
F2 - Acquisition Parameters
Date_ 20200229
Time 10.28 h
INSTRUM Avance
PROBHD 2169738_0003 (
PULPROG zgpg
TD 65536
SOLVENT CDCl3
NS 153
DS 2
SWH 30120.482 Hz
FIDRES 0.919204 Hz
AQ 1.0878977 sec
RG 101
DW 16.600 usec
DE 6.72 usec
TE 298.2 K
D1 2.00000000 sec
D11 0.03000000 sec
TD0 1
SFO1 125.7709931 MHz
NUC1 13C
P1 9.00 usec
PLW1 110.76999664 W
SFO2 500.1320005 MHz
NUC2 1H
CPDPRG[2 waltz64
PCPD2 80.00 usec
PLW2 24.45700073 W
PLW12 0.24457000 W
PLW13 0.12302000 W
F2 - Processing parameters
SI 65536
SF 125.7578060 MHz
WDW EM
SSB 0
LB 2.00 Hz
GB 0
PC 1.40

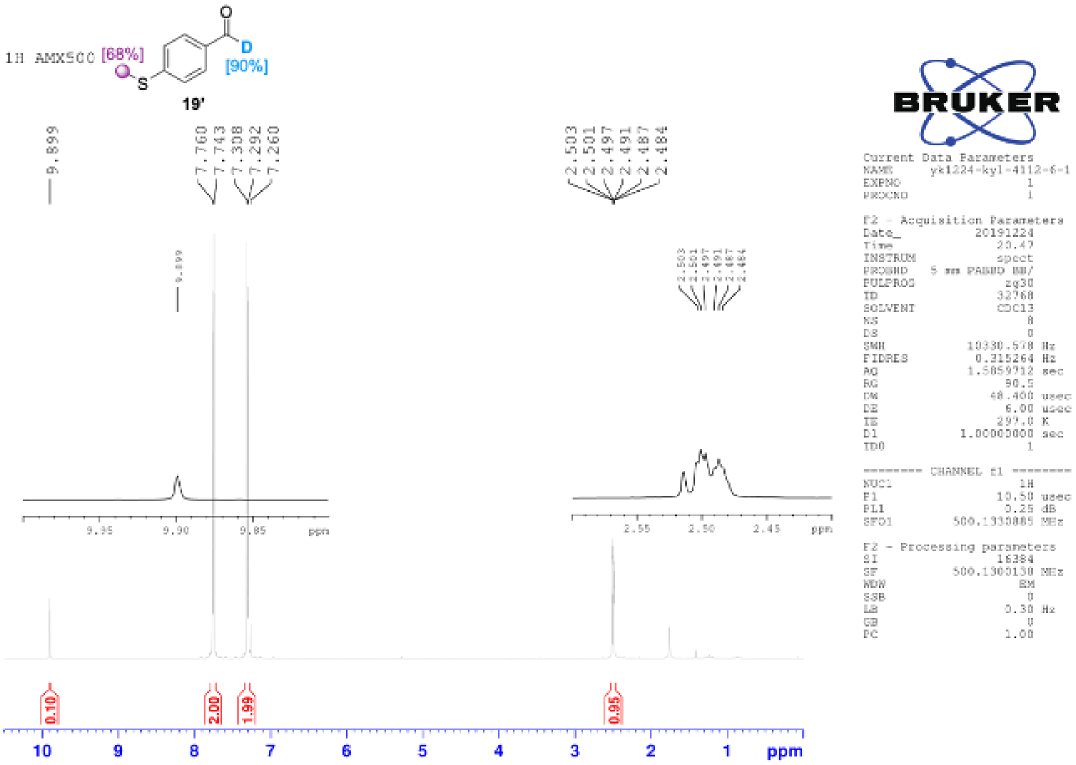
1H AMX500
[68%]
S
D
[90%]
19'
9.899
7.760
7.743
7.308
7.292
7.260
2.503
2.501
2.497
2.491
2.487
2.484
0.10
2.00
1.99
0.95
10 9 8 7 6 5 4 3 2 1 ppm
BRUKER
Current Data Parameters
NAME yk1224-kyl-4112-6-1
EXPNO 1
PROCNO 1
F2 - Acquisition Parameters
Date_ 20191224
Time 20.47
INSTRUM spect
PROBHD 5 mm PABBO BB/
PULPROG zg30
TD 32768
SOLVENT CDCl3
NS 8
DS 0
SWH 10330.578 Hz
FIDRES 0.315264 Hz
AQ 1.5859712 sec
RG 90.5
DW 48.400 usec
DE 6.00 usec
TE 297.0 K
D1 1.00000000 sec
TD0 1
======== CHANNEL f1 ========
NUC1 1H
P1 10.50 usec
PL1 0.25 dB
SFO1 500.1330885 MHz
F2 - Processing parameters
SI 16384
SF 500.1300130 MHz
WDW EM
SSB 0
LB 0.30 Hz
GB 0
PC 1.00

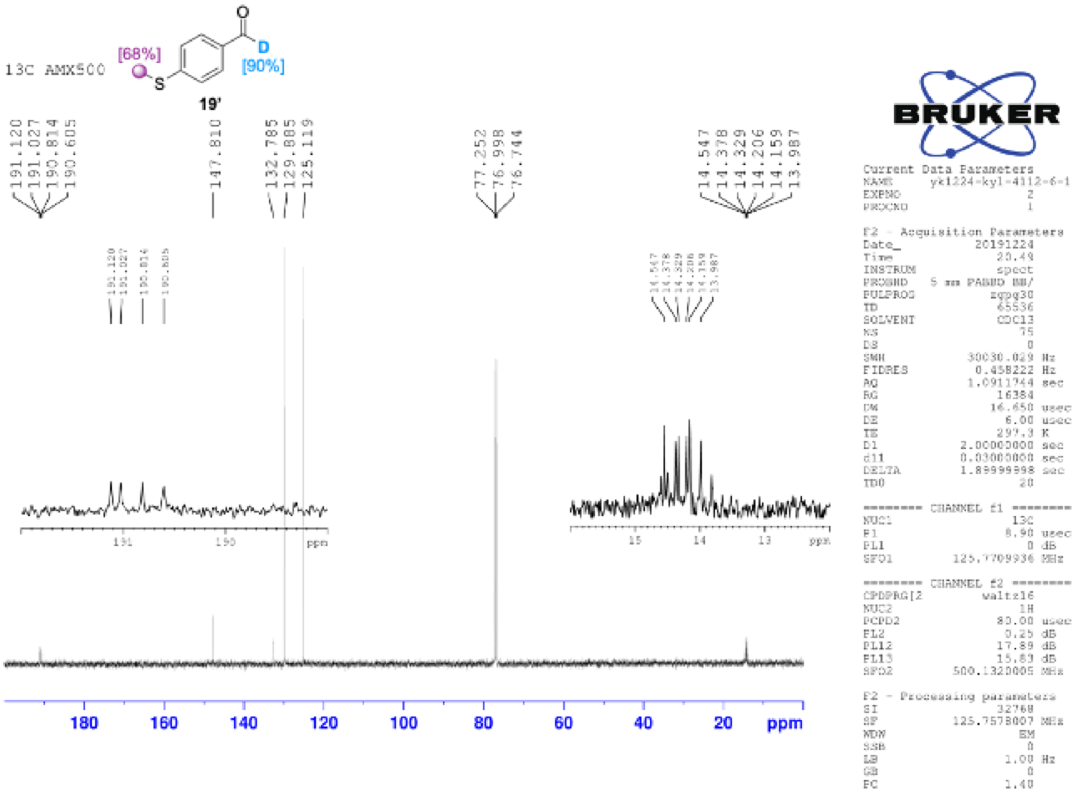
13C AMX500
[68%]
S
D
[90%]
19'
191.120
191.027
190.814
190.605
147.810
132.785
129.885
125.119
77.252
76.998
76.744
14.547
14.378
14.329
14.206
14.159
13.987
180 160 140 120 100 80 60 40 20 ppm
BRUKER
Current Data Parameters
NAME yk1224-kyl-4112-6-1
EXPNO 2
PROCNO 1
F2 - Acquisition Parameters
Date_ 20191224
Time 20.49
INSTRUM spect
PROBHD 5 mm PABBO BB/
PULPROG zgpg30
TD 65536
SOLVENT CDCl3
NS 75
DS 0
SWH 30030.029 Hz
FIDRES 0.458222 Hz
AQ 1.0911744 sec
RG 16384
DW 16.650 usec
DE 6.00 usec
TE 297.3 K
D1 2.00000000 sec
d11 0.03000000 sec
DELTA 1.89999998 sec
TD0 20
======== CHANNEL f1 ========
NUC1 13C
P1 8.90 usec
PL1 0 dB
SFO1 125.7709936 MHz
======== CHANNEL f2 ========
CPDPRG[2 waltz16
NUC2 1H
PCPD2 80.00 usec
PL2 0.25 dB
PL12 17.89 dB
PL13 15.83 dB
SFO2 500.1320005 MHz
F2 - Processing parameters
SI 32768
SF 125.7578007 MHz
WDW EM
SSB 0
LB 1.00 Hz
GB 0
PC 1.40

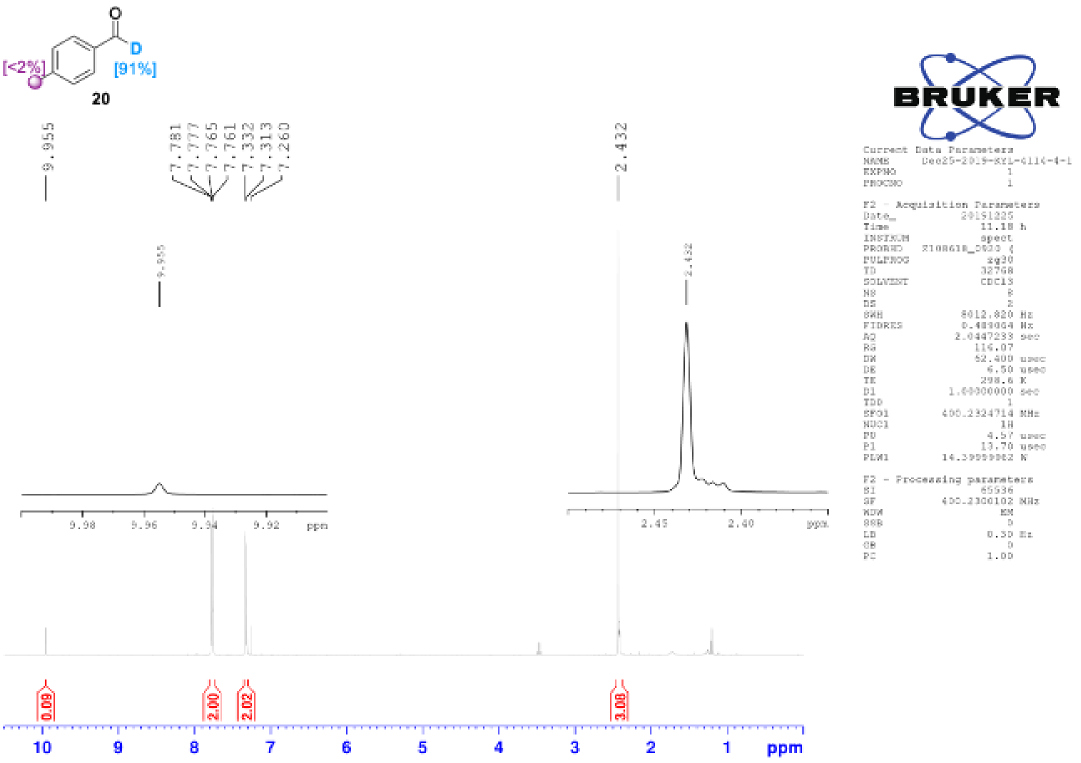
O
D
[91%]
[<2%]
20
9.955
7.781 7.777 7.765 7.761 7.332 7.313 7.260
2.432
0.09
2.00
2.02
3.08
ppm
BRUKER

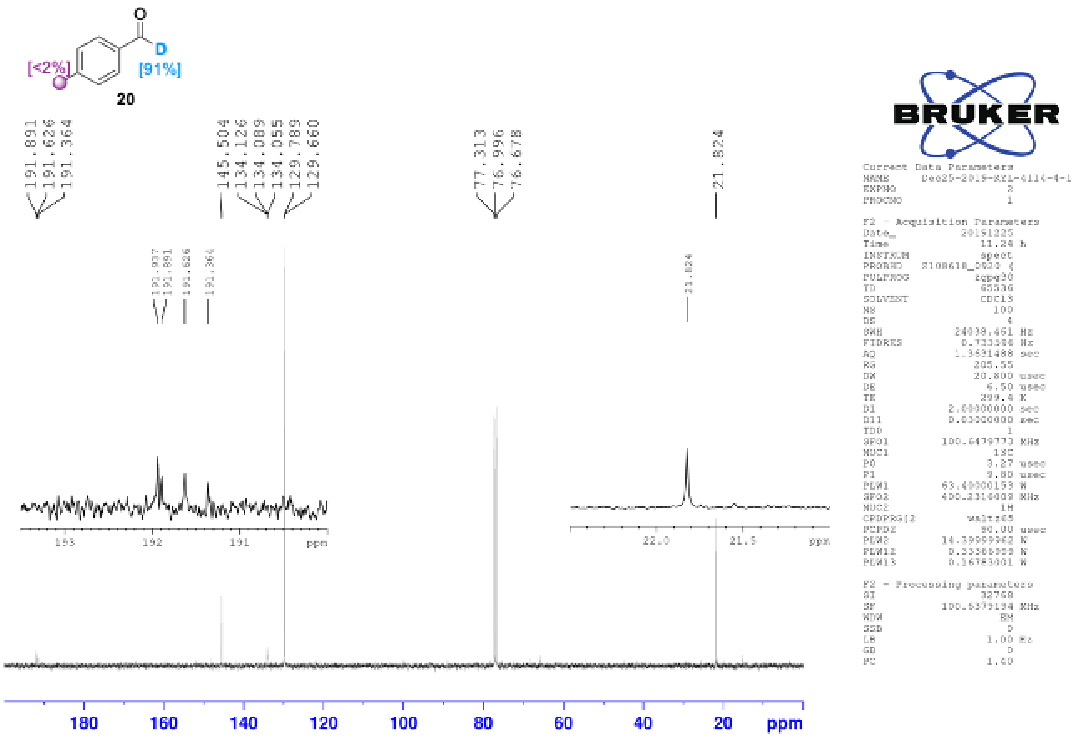
O
D
[91%]
[<2%]
20
191.891 191.626 191.364
145.504 134.126 134.089 134.055 129.789 129.660
77.313 76.996 76.678
21.824
ppm
BRUKER

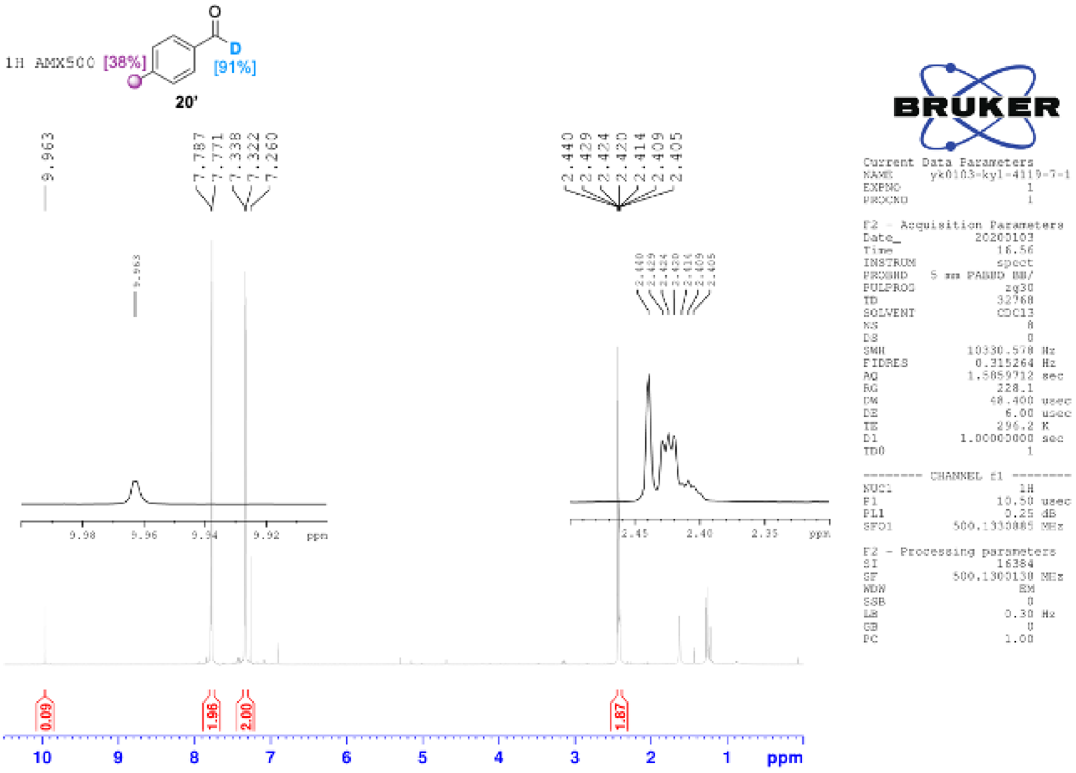
1H AMX500
[38%]
D
[91%]
20'
9.963
7.787
7.771
7.338
7.322
7.260
2.440
2.429
2.424
2.420
2.414
2.409
2.405
BRUKER
0.09
1.96
2.00
1.87
ppm

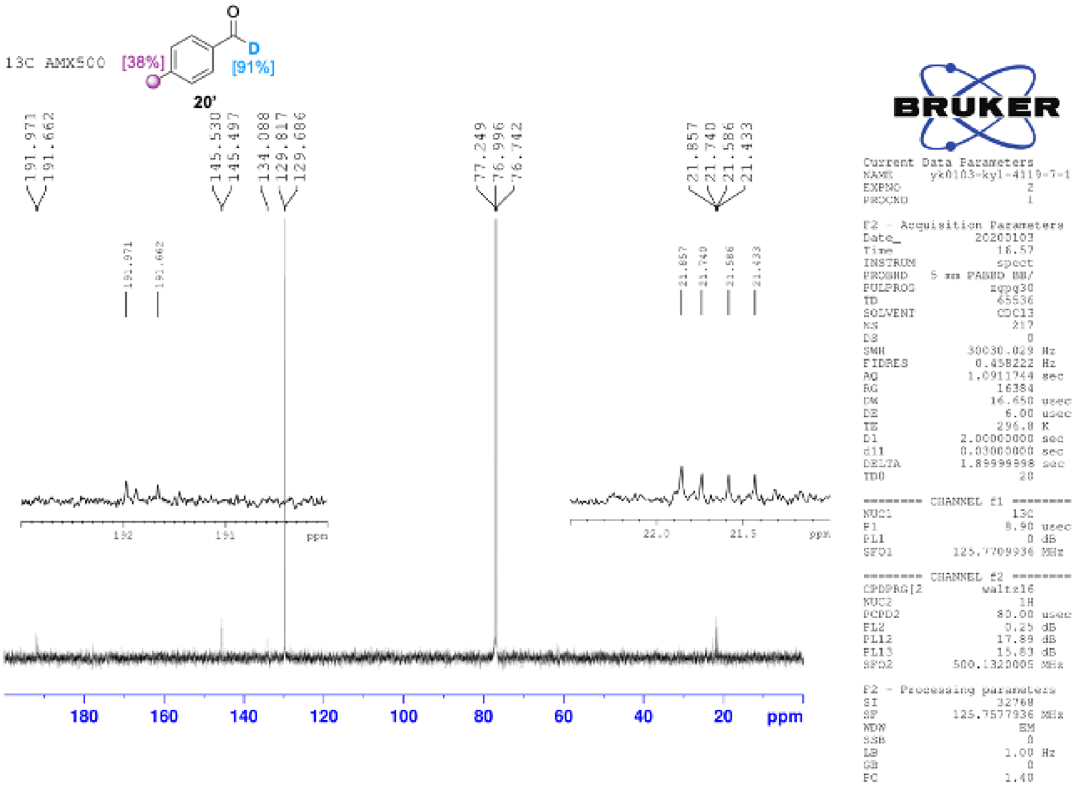
13C AMX500
[38%]
D
[91%]
20'
191.971
191.662
145.530
145.497
134.088
129.817
129.686
77.249
76.996
76.742
21.857
21.740
21.586
21.433
BRUKER
ppm

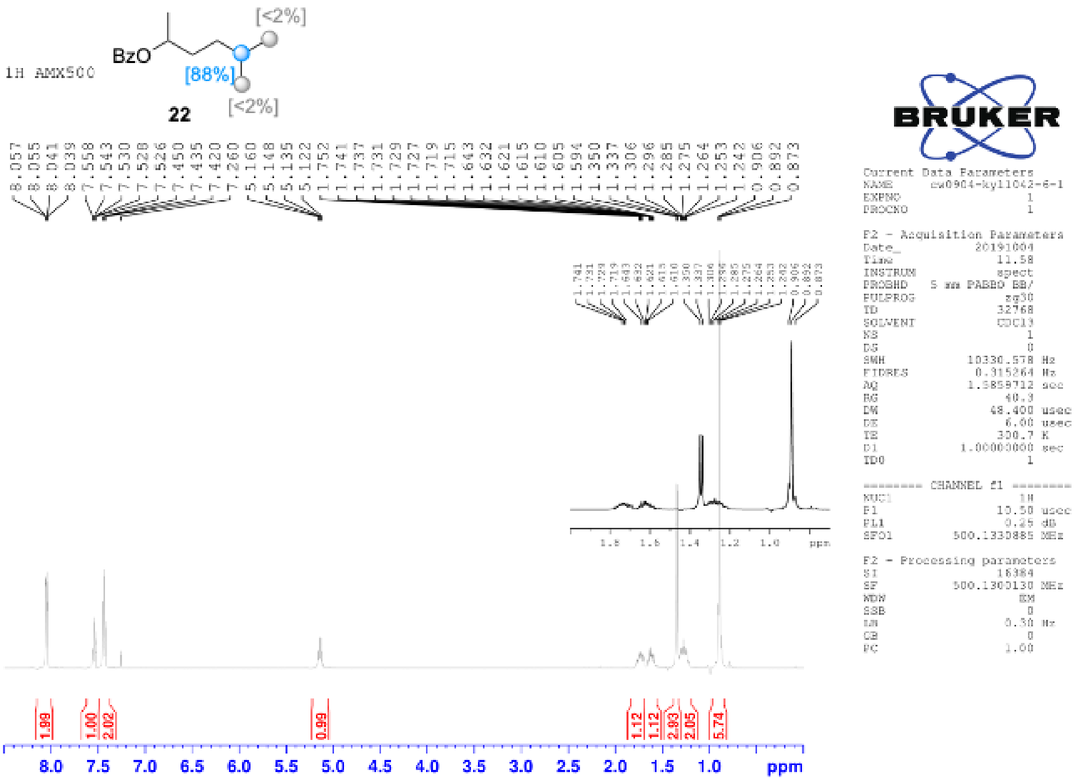
1H AMX500
BzO
[88%]
[<2%]
[<2%]
22
Current Data Parameters
NAME cw0904-kyl1042-6-1
EXPNO 1
PROCNO 1
F2 - Acquisition Parameters
Date_ 20191004
SOLVENT CDCl3
F2 - Processing parameters
ppm

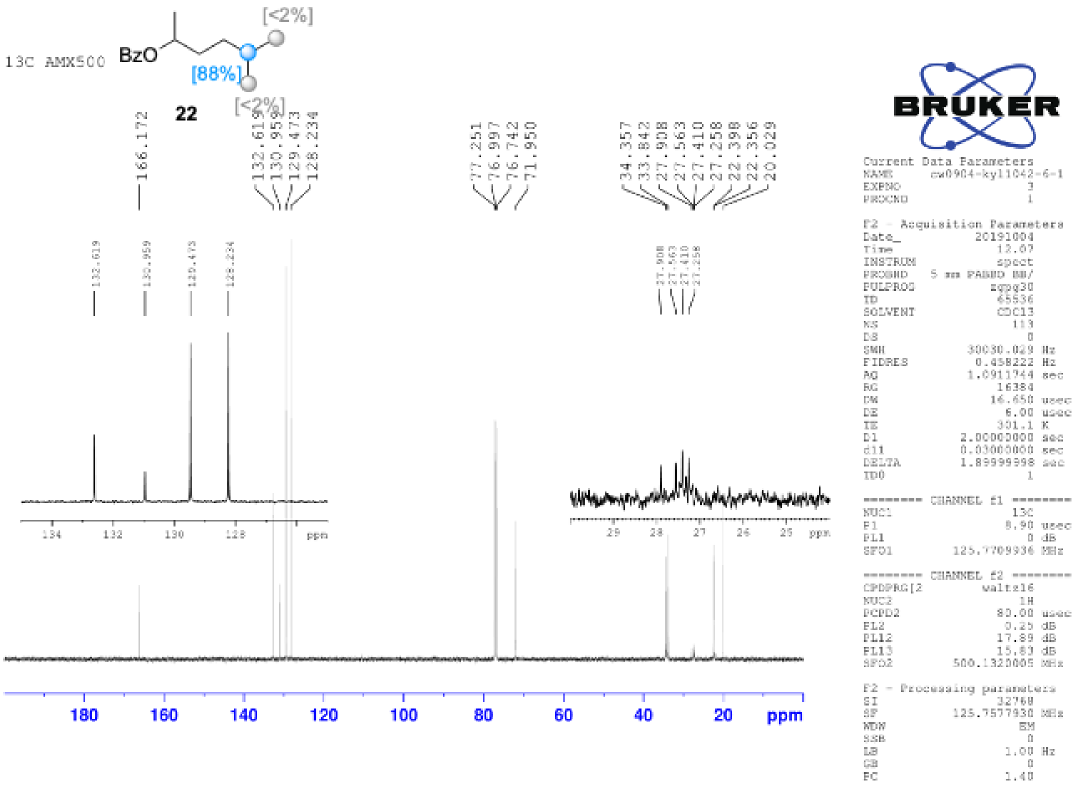
13C AMX500
BzO
[88%]
[<2%]
[<2%]
22
166.172
132.619
130.959
129.473
128.234
77.251
76.997
76.742
71.950
34.357
33.842
27.908
27.563
27.410
27.258
22.398
22.356
20.029
Current Data Parameters
NAME cw0904-kyl1042-6-1
EXPNO 3
PROCNO 1
F2 - Acquisition Parameters
Date_ 20191004
SOLVENT CDCl3
F2 - Processing parameters
ppm

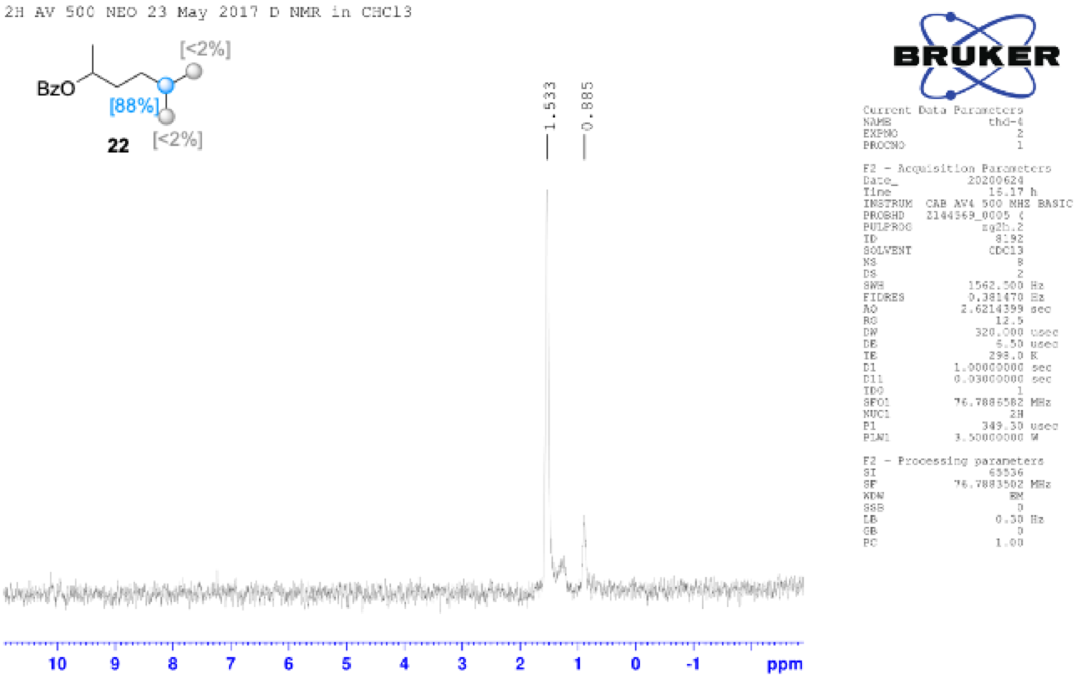
2H AV 500 NEO 23 May 2017 D NMR in CHCl3
BzO
[<2%]
[88%]
[<2%]
22
1.533
0.885
BRUKER
Current Data Parameters
NAME thd-4
EXPNO 2
PROCNO 1
F2 - Acquisition Parameters
Date_ 20200624
Time 16.17 h
INSTRUM CAB AV4 500 MHZ BASIC
PROBHD 2144369_0005 (
PULPROG zg2h.2
TD 8192
SOLVENT CDCl3
NS 8
DS 2
SWH 1562.500 Hz
FIDRES 0.381470 Hz
AQ 2.6214399 sec
RG 12.5
DW 320.000 usec
DE 6.50 usec
TE 298.0 K
D1 1.00000000 sec
D11 0.03000000 sec
TD0 1
SFO1 76.7886582 MHz
NUC1 2H
P1 349.30 usec
PLW1 3.50000000 W
F2 - Processing parameters
SI 65536
SF 76.7883502 MHz
WDW EM
SSB 0
LB 0.30 Hz
GB 0
PC 1.00
10 9 8 7 6 5 4 3 2 1 0 -1 ppm

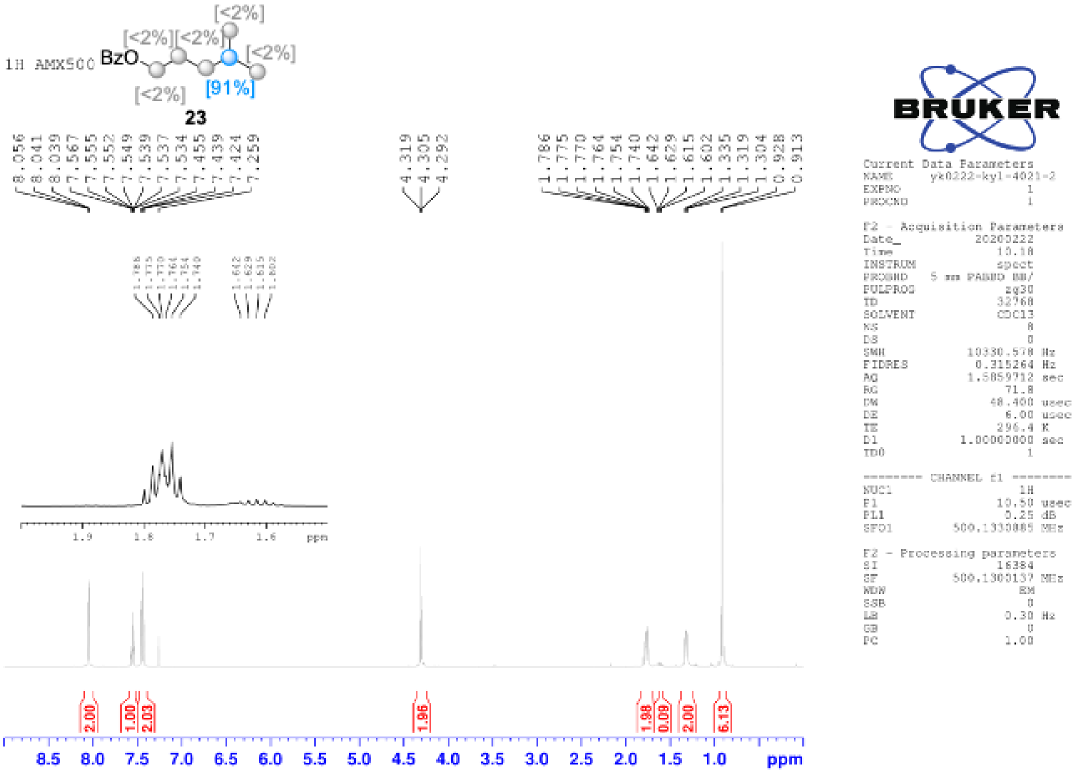
1H AMX500
BzO
[<2%]
[<2%]
[<2%]
[<2%]
[<2%]
[91%]
23
8.056 8.041 8.039 7.567 7.555 7.552 7.549 7.539 7.537 7.534 7.455 7.439 7.424 7.259
4.319 4.305 4.292
1.786 1.775 1.770 1.764 1.754 1.740 1.642 1.629 1.615 1.602 1.335 1.319 1.304 0.928 0.913
BRUKER
Current Data Parameters
NAME yk0222-kyl-4021-2
EXPNO 1
PROCNO 1
F2 - Acquisition Parameters
Date_ 20200222
Time 10.18
INSTRUM spect
PROBHD 5 mm PABBO BB/
PULPROG zg30
TD 32768
SOLVENT CDCl3
NS 8
DS 0
SWH 10330.578 Hz
FIDRES 0.315264 Hz
AQ 1.5859712 sec
RG 71.8
DW 48.400 usec
DE 6.00 usec
TE 296.4 K
D1 1.00000000 sec
TD0 1
======== CHANNEL f1 ========
NUC1 1H
P1 10.50 usec
PL1 0.25 dB
SFO1 500.1330885 MHz
F2 - Processing parameters
SI 16384
SF 500.1300137 MHz
WDW EM
SSB 0
LB 0.30 Hz
GB 0
PC 1.00
1.9 1.8 1.7 1.6 ppm
2.00 1.00 2.03 1.96 1.98 0.09 2.00 6.13
8.5 8.0 7.5 7.0 6.5 6.0 5.5 5.0 4.5 4.0 3.5 3.0 2.5 2.0 1.5 1.0 ppm

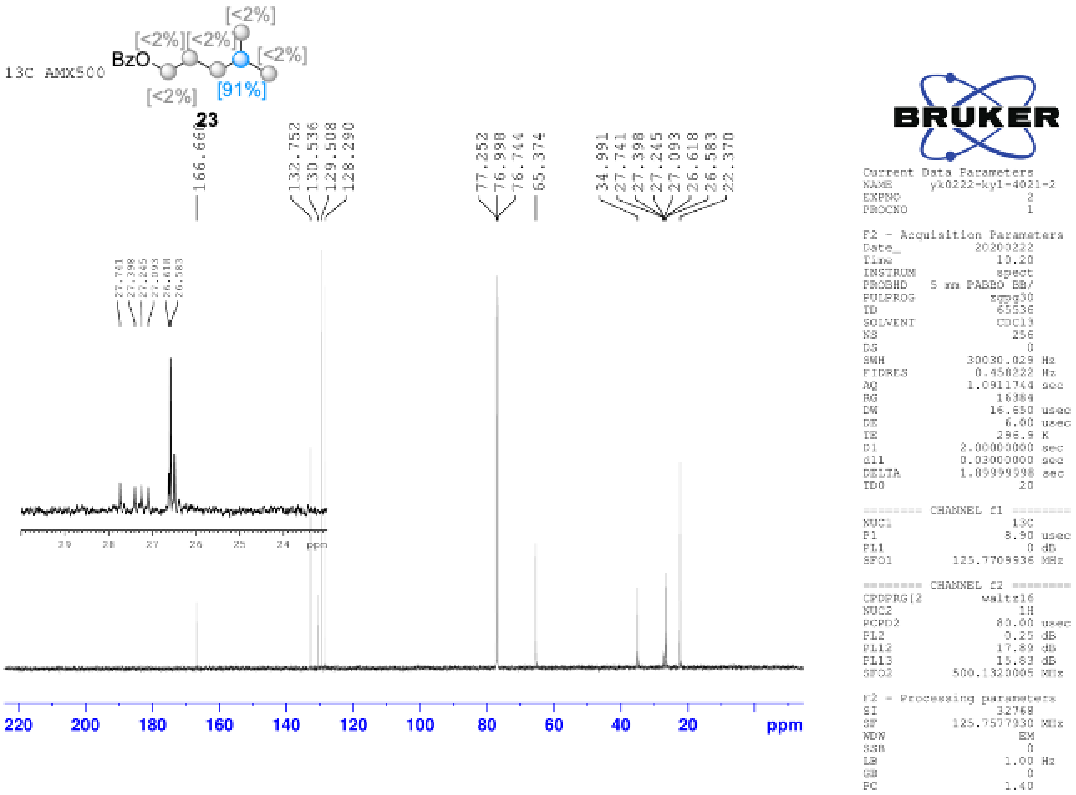
13C AMX500
BzO
[<2%]
[91%]
23
166.660
132.752
130.536
129.508
128.290
77.252
76.998
76.744
65.374
34.991
27.741
27.398
27.245
27.093
26.618
26.583
22.370
ppm

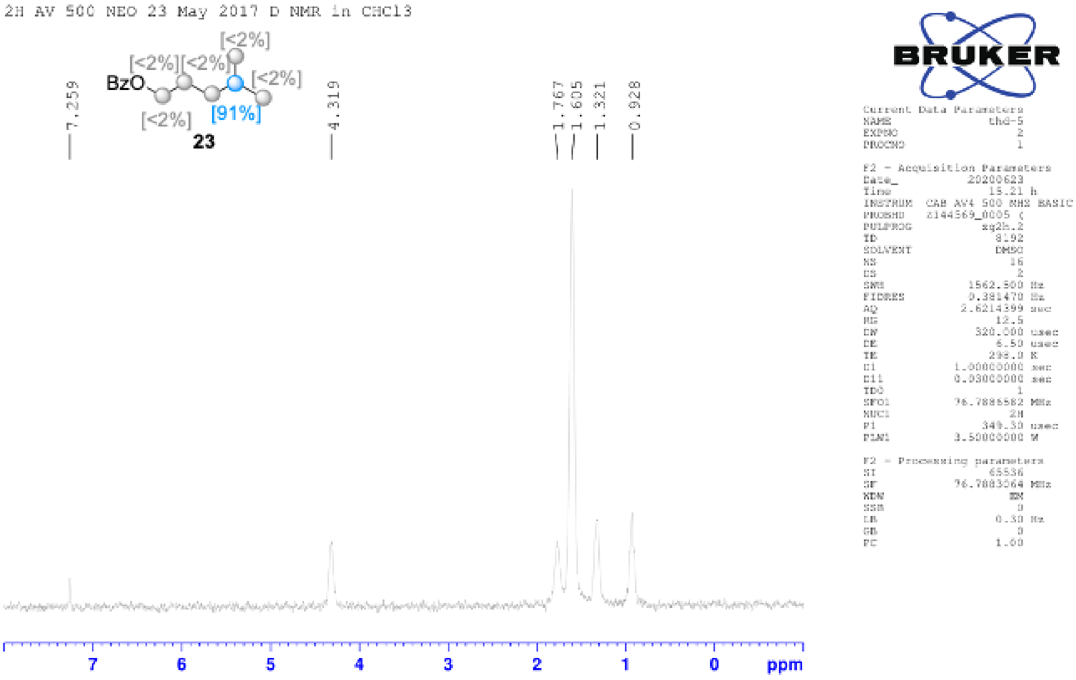
2H AV 500 NEO 23 May 2017 D NMR in CHCl3
BzO
[<2%]
[91%]
23
7.259
4.319
1.767
1.605
1.321
0.928
ppm

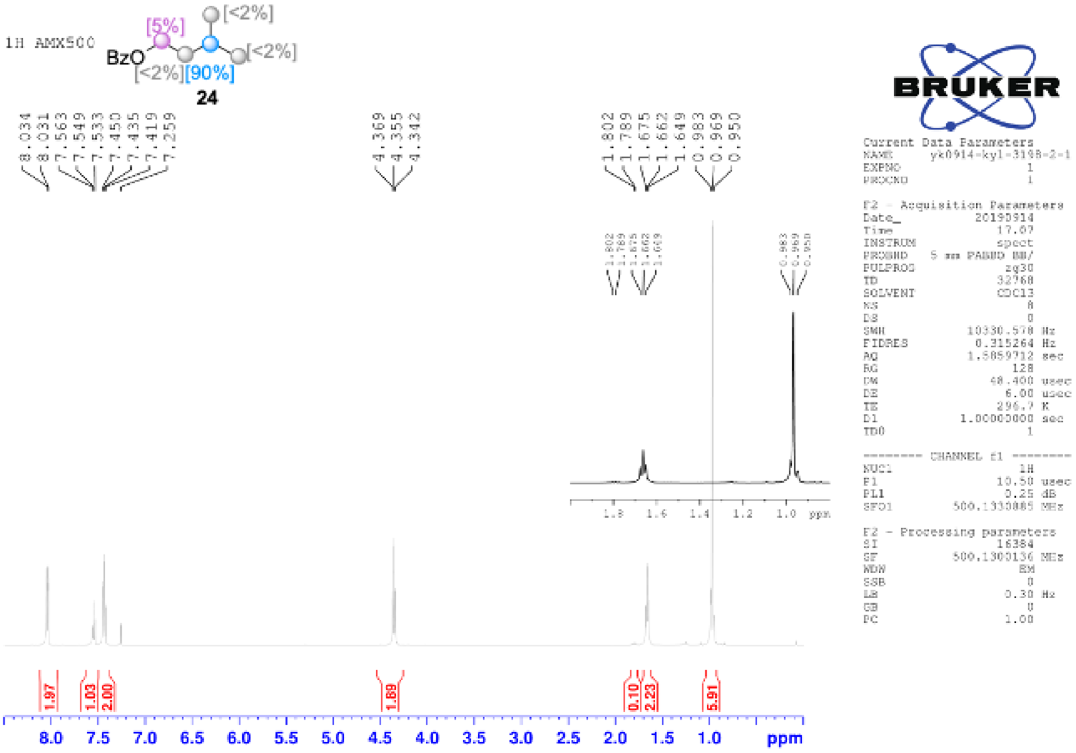
1H AMX500
BzO
[5%]
[<2%]
[<2%]
[<2%]
[90%]
24
8.034
8.031
7.563
7.549
7.533
7.450
7.435
7.419
7.259
4.369
4.355
4.342
1.802
1.789
1.675
1.662
1.649
0.983
0.969
0.950
1.97
1.03
2.00
1.89
0.10
2.23
5.91
8.0 7.5 7.0 6.5 6.0 5.5 5.0 4.5 4.0 3.5 3.0 2.5 2.0 1.5 1.0 ppm
BRUKER

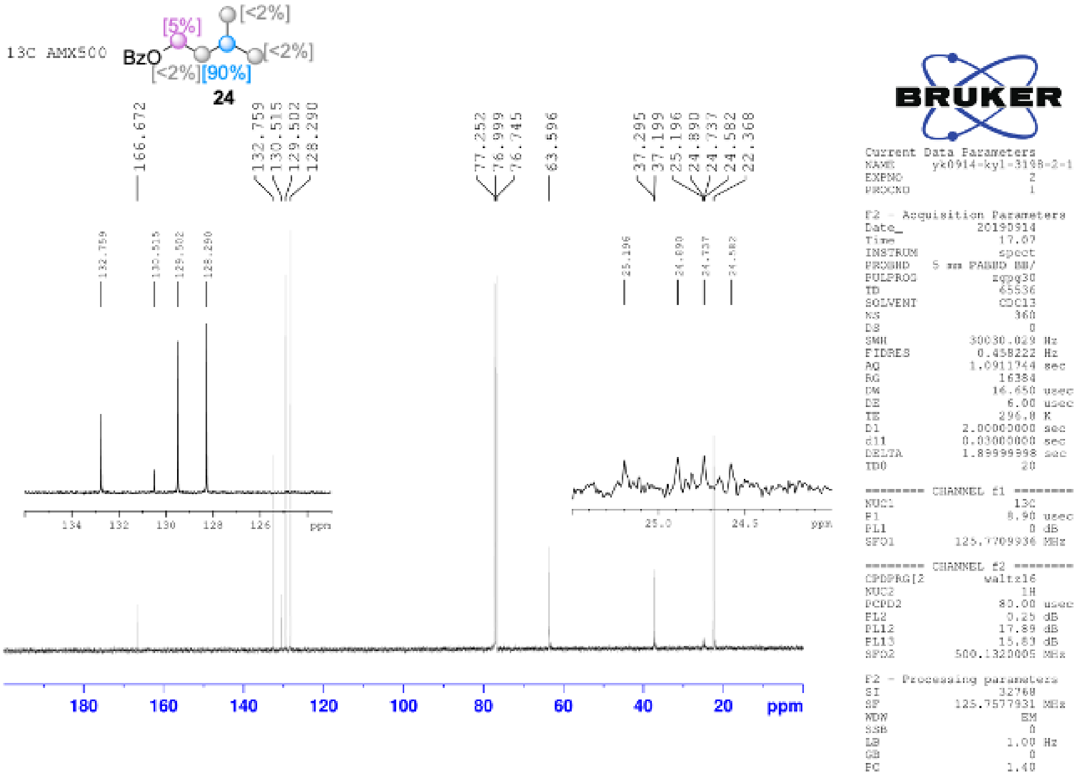
13C AMX500
BzO
[5%]
[<2%]
[<2%]
[<2%]
[90%]
24
166.672
132.759
130.515
129.502
128.290
77.252
76.999
76.745
63.596
37.295
37.199
25.196
24.890
24.737
24.582
22.368
180 160 140 120 100 80 60 40 20 ppm
BRUKER

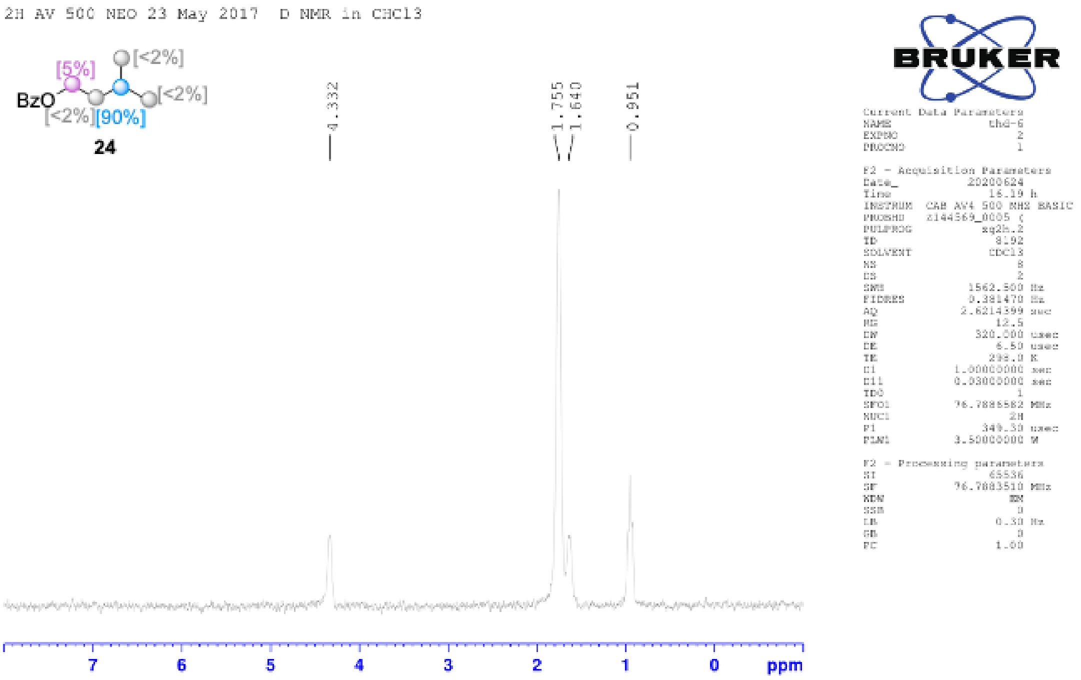

2H AV 500 NEO 23 May 2017 D NMR in CHCl3
BzO
[5%]
[<2%]
[<2%]
[90%]
[<2%]
24
4.332
1.755
1.640
0.951
BRUKER
7
6
5
4
3
2
1
0
ppm

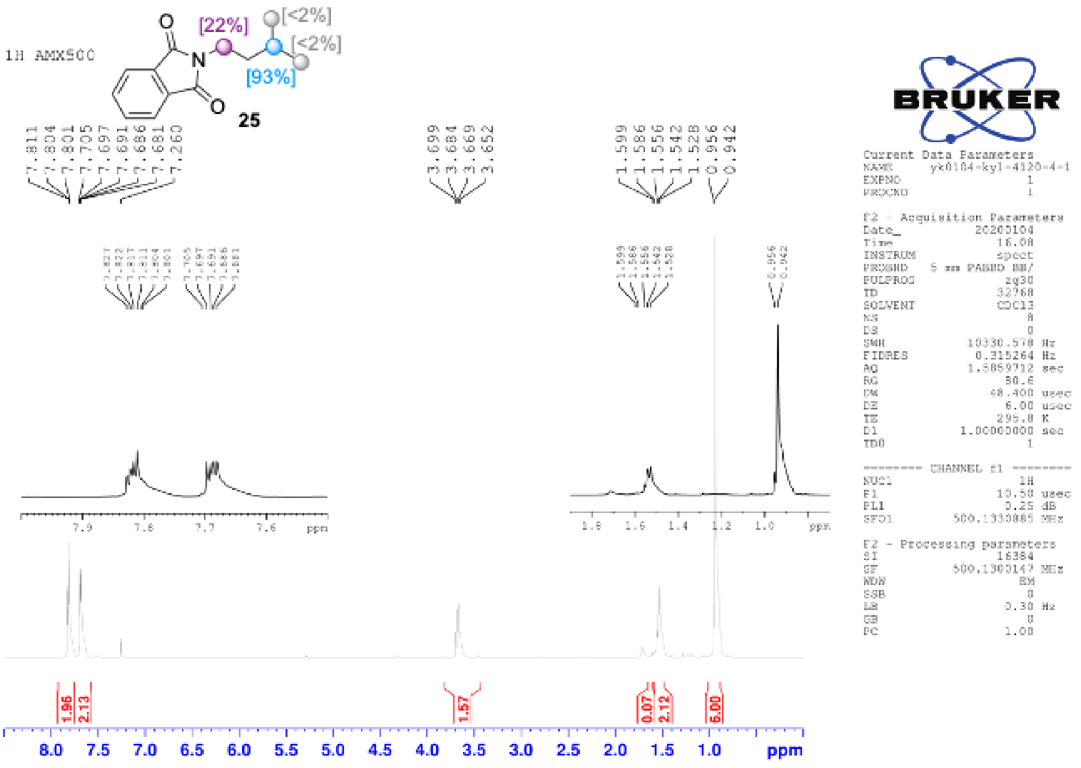

1H AMX500
[22%]
[<2%]
[<2%]
[93%]
25
7.811
7.804
7.801
7.705
7.697
7.691
7.686
7.681
7.260
3.699
3.684
3.669
3.652
1.599
1.586
1.556
1.542
1.528
0.956
0.942
BRUKER
1.96
2.13
1.57
0.07
2.12
6.00
8.0
7.5
7.0
6.5
6.0
5.5
5.0
4.5
4.0
3.5
3.0
2.5
2.0
1.5
1.0
ppm

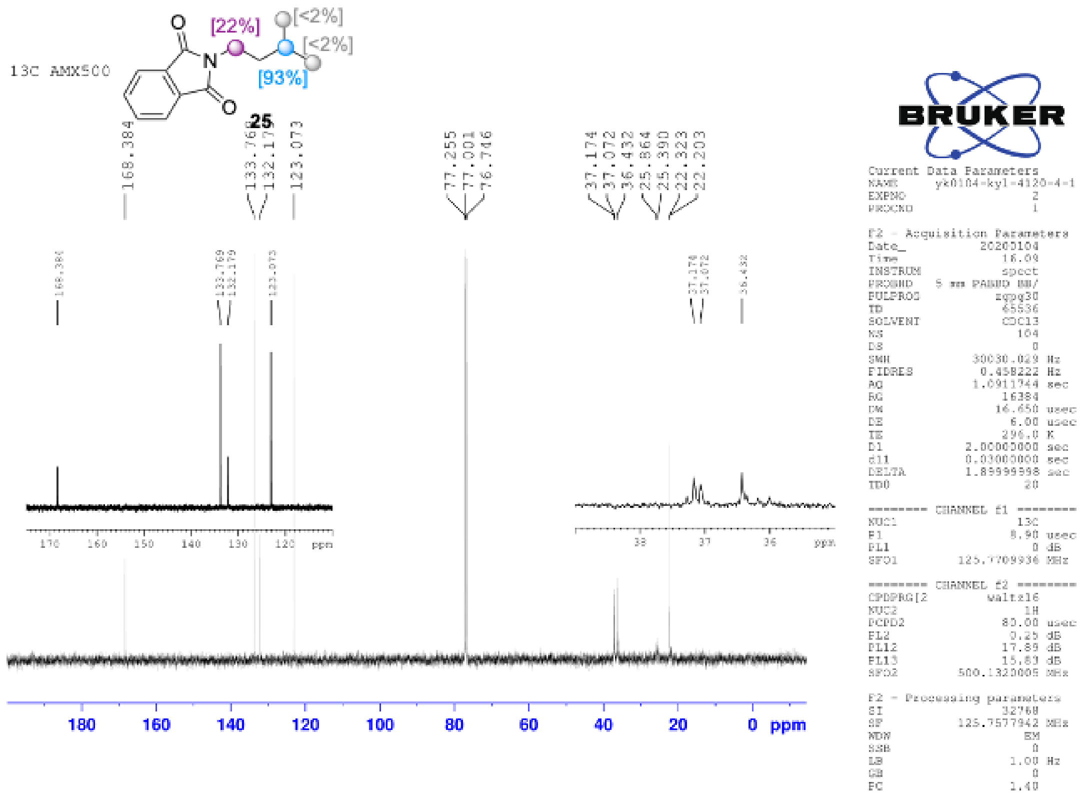

13C AMX500
[22%]
[<2%]
[<2%]
[93%]
25
168.384
133.769
132.179
123.073
77.255
77.001
76.746
37.174
37.072
36.432
25.864
25.390
22.323
22.203
BRUKER
Current Data Parameters
NAME yk0104-kyl-4120-4-1
EXPNO 2
PROCNO 1
F2 - Acquisition Parameters
Date_ 20200104
Time 16.09
INSTRUM spect
PROBHD 5 mm PABBO BB/
PULPROG zgpg30
TD 65536
SOLVENT CDCl3
NS 104
DS 0
SWH 30030.029 Hz
FIDRES 0.458222 Hz
AQ 1.0911744 sec
RG 16384
DW 16.650 usec
DE 6.00 usec
TE 296.0 K
D1 2.00000000 sec
d11 0.03000000 sec
DELTA 1.89999998 sec
TD0 20
======== CHANNEL f1 ========
NUC1 13C
P1 8.90 usec
PL1 0 dB
SFO1 125.7709936 MHz
======== CHANNEL f2 ========
CPDPRG[2 waltz16
NUC2 1H
PCPD2 80.00 usec
PL2 0.25 dB
PL12 17.89 dB
PL13 15.83 dB
SFO2 500.1320005 MHz
F2 - Processing parameters
SI 32768
SF 125.7577942 MHz
WDW EM
SSB 0
LB 1.00 Hz
GB 0
PC 1.40
ppm

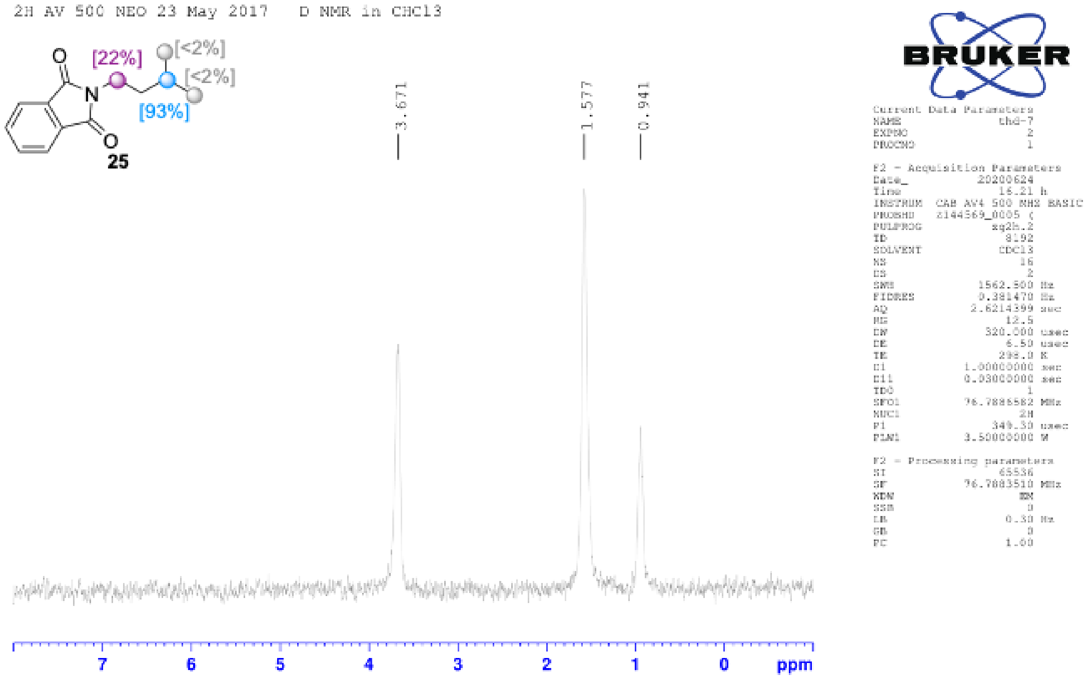

2H AV 500 NEO 23 May 2017 D NMR in CHCl3
[22%]
[<2%]
[<2%]
[93%]
25
3.671
1.577
0.941
BRUKER
Current Data Parameters
NAME thd-7
EXPNO 2
PROCNO 1
F2 - Acquisition Parameters
Date_ 20200624
Time 16.21 h
INSTRUM CAB AV4 500 MHZ BASIC
PULPROG zg2h.2
TD 8192
SOLVENT CDCl3
NS 16
DS 2
SWH 1562.500 Hz
FIDRES 0.381470 Hz
AQ 2.6214399 sec
RG 12.5
DW 320.000 usec
DE 6.50 usec
TE 298.0 K
D1 1.00000000 sec
D11 0.03000000 sec
TD0 1
SFO1 76.7886582 MHz
NUC1 2H
F2 - Processing parameters
SI 65536
SF 76.7883510 MHz
WDW EM
SSB 0
LB 0.30 Hz
GB 0
PC 1.00
ppm

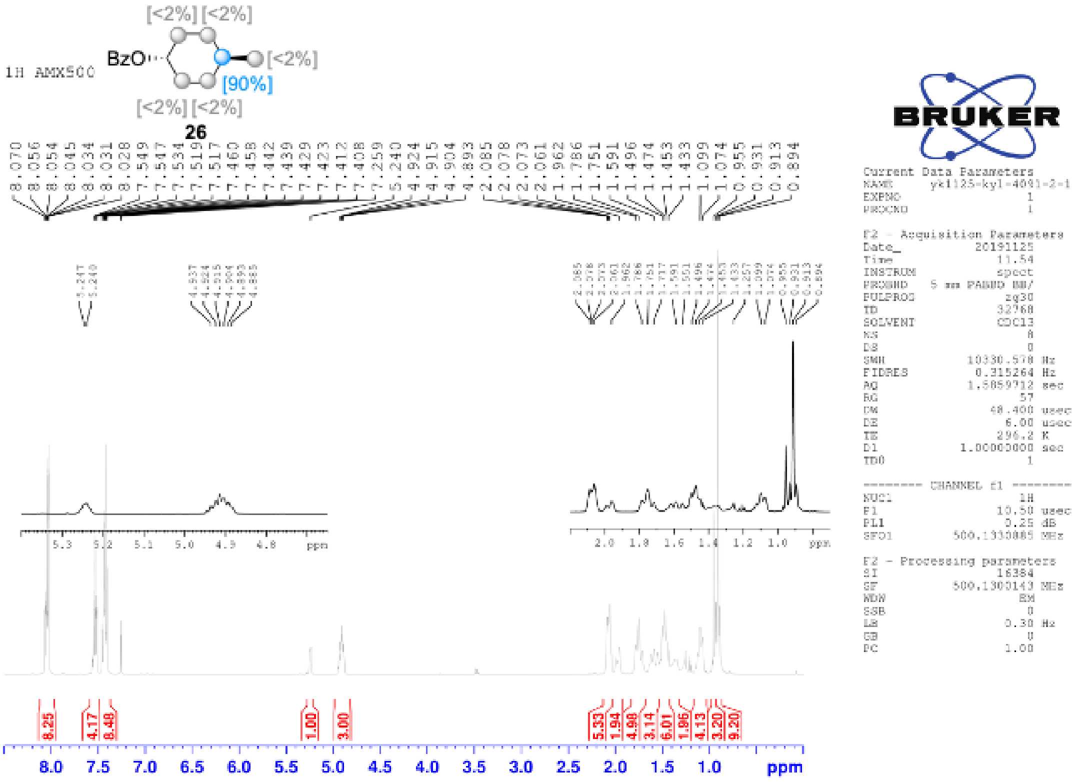
1H AMX500
BzO
[<2%] [<2%]
[<2%]
[90%]
[<2%] [<2%]
26
BRUKER
ppm

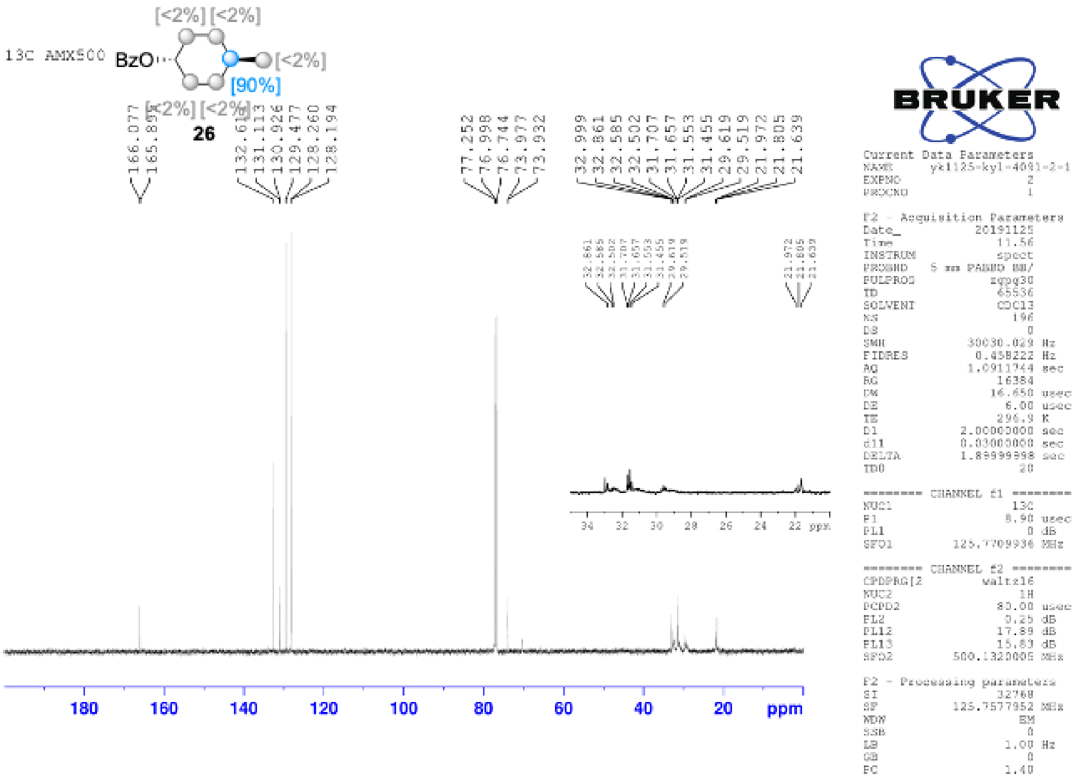
13C AMX500
BzO
[<2%] [<2%]
[<2%]
[90%]
[<2%] [<2%]
26
BRUKER
ppm

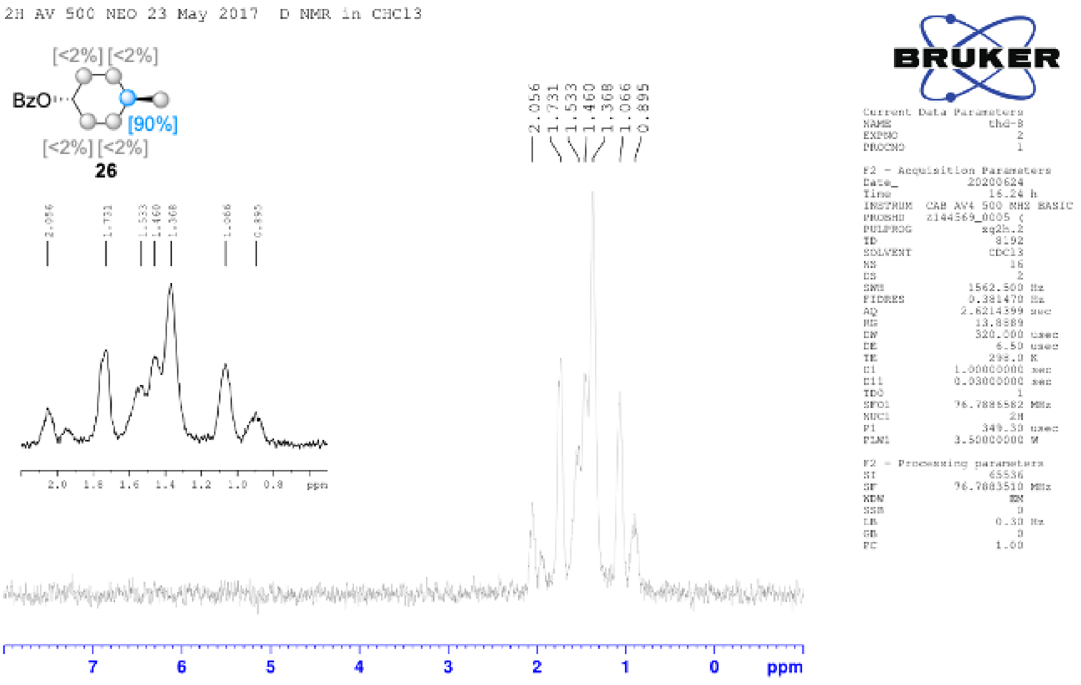
2H AV 500 NEO 23 May 2017 D NMR in CHCl3
BzO
[<2%] [<2%]
[90%]
[<2%] [<2%]
26
BRUKER
ppm

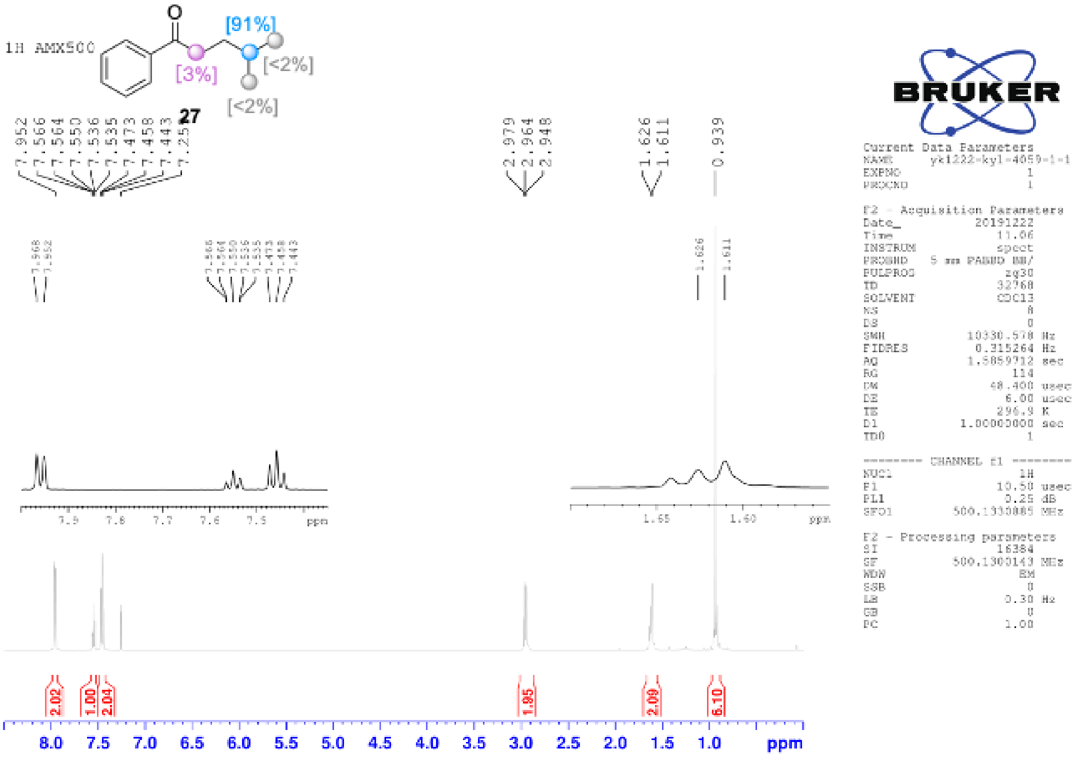
1H AMX500
[91%]
[3%]
[<2%]
[<2%]
27
BRUKER
ppm

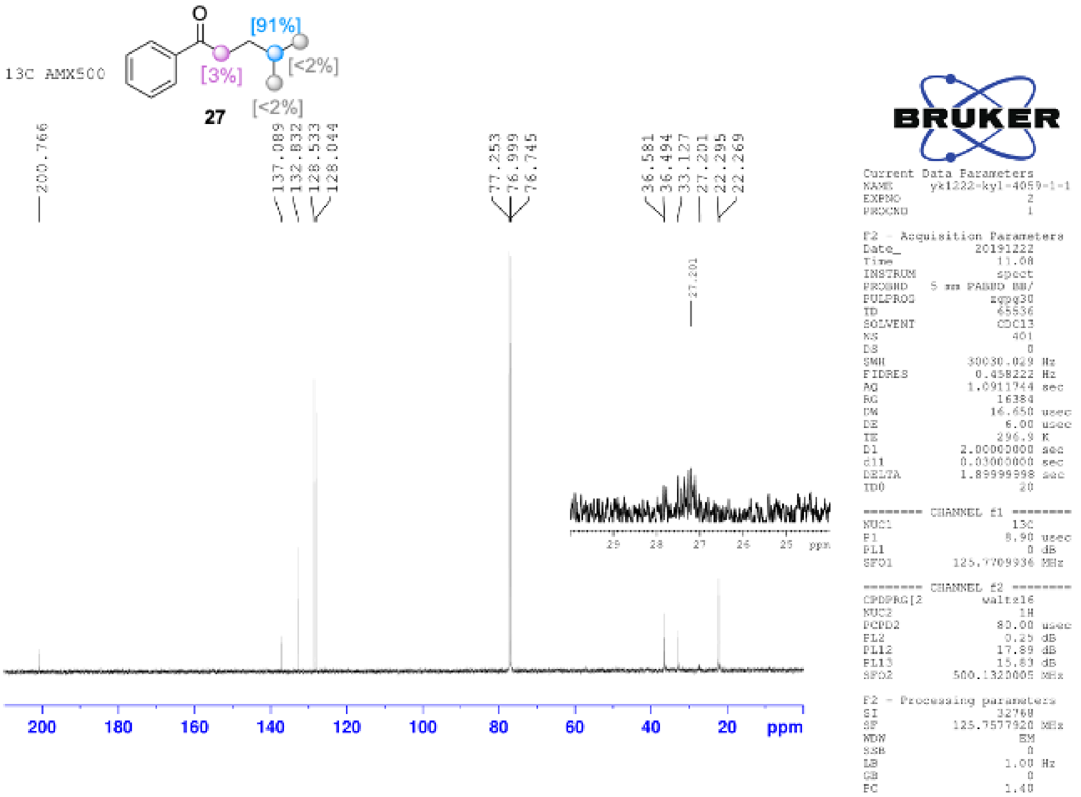
13C AMX500
[91%]
[3%]
[<2%]
[<2%]
27
200.766
137.089
132.832
128.533
128.044
77.253
76.999
76.745
36.581
36.494
33.127
27.201
22.295
22.269
27.201
200
180
160
140
120
100
80
60
40
20
ppm
BRUKER
Current Data Parameters
NAME yk1222-kyl-4059-1-1
EXPNO 2
PROCNO 1
F2 - Acquisition Parameters
Date_ 20191222
Time 11.08
INSTRUM spect
PROBHD 5 mm PABBO BB/
PULPROG zgpg30
TD 65536
SOLVENT CDCl3
NS 401
DS 0
SWH 30030.029 Hz
FIDRES 0.458222 Hz
AQ 1.0911744 sec
RG 16384
DW 16.650 usec
DE 6.00 usec
TE 296.9 K
D1 2.00000000 sec
d11 0.03000000 sec
DELTA 1.89999998 sec
TD0 20
======== CHANNEL f1 ========
NUC1 13C
P1 8.90 usec
PL1 0 dB
SFO1 125.7709936 MHz
======== CHANNEL f2 ========
CPDPRG[2 waltz16
NUC2 1H
PCPD2 80.00 usec
PL2 0.25 dB
PL12 17.89 dB
PL13 15.83 dB
SFO2 500.1320005 MHz
F2 - Processing parameters
SI 32768
SF 125.7577920 MHz
WDW EM
SSB 0
LB 1.00 Hz
GB 0
PC 1.40

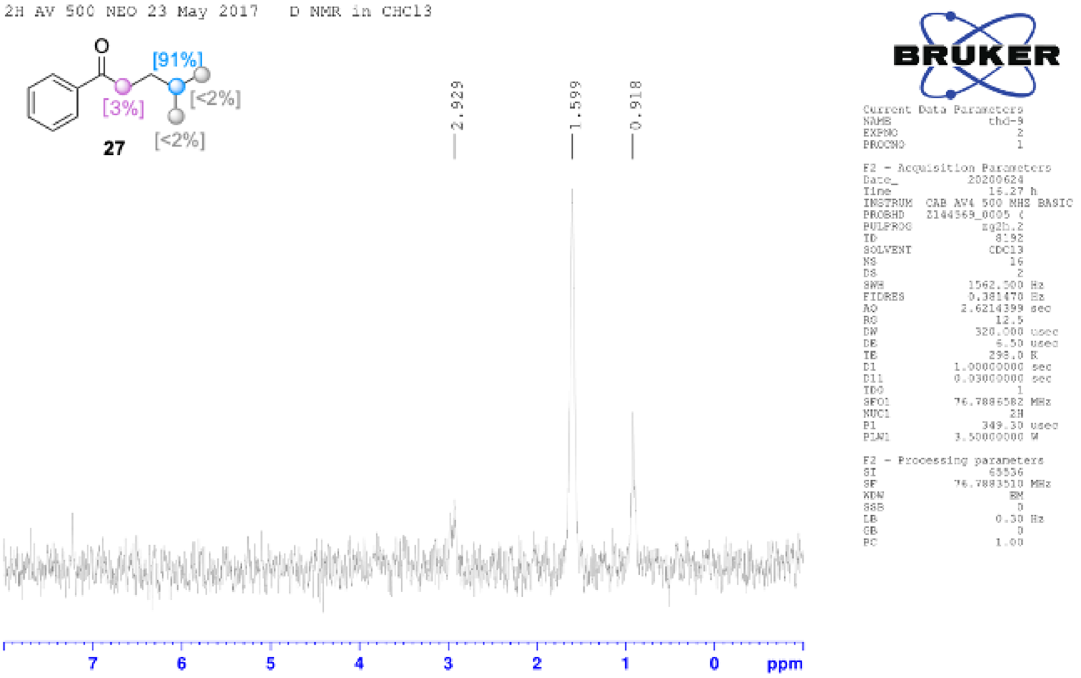
2H AV 500 NEO 23 May 2017 D NMR in CHCl3
[91%]
[3%]
[<2%]
[<2%]
27
2.929
1.599
0.918
7
6
5
4
3
2
1
0
ppm
BRUKER
Current Data Parameters
NAME thd-9
EXPNO 2
PROCNO 1
F2 - Acquisition Parameters
Date_ 20200624
Time 16.27 h
INSTRUM CAB AV4 500 MHz BASIC
PROBHD Z144369_0005 (
PULPROG zg2h.2
TD 8192
SOLVENT CDCl3
NS 16
DS 2
SWH 1562.500 Hz
FIDRES 0.381470 Hz
AQ 2.6214399 sec
RG 12.5
DW 320.000 usec
DE 6.50 usec
TE 298.0 K
D1 1.00000000 sec
D11 0.03000000 sec
TD0 1
SFO1 76.7886582 MHz
NUC1 2H
P1 349.30 usec
PLW1 3.50000000 W
F2 - Processing parameters
SI 65536
SF 76.7883510 MHz
WDW EM
SSB 0
LB 0.30 Hz
GB 0
PC 1.00

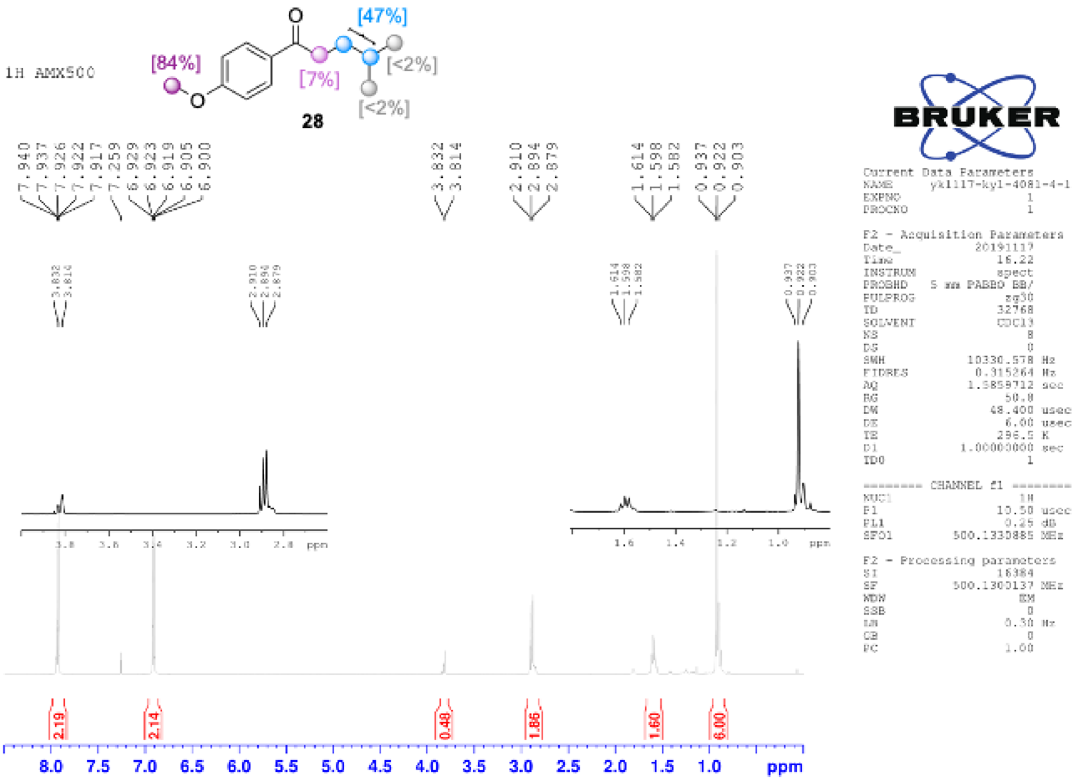
1H AMX500
[84%]
[7%]
[47%]
[<2%]
[<2%]
28
Current Data Parameters
NAME yk1117-kyl-4081-4-1
EXPNO 1
PROCNO 1
F2 - Acquisition Parameters
Date_ 20191117
Time 16.22
INSTRUM spect
PROBHD 5 mm PABBO BB/
PULPROG zg30
TD 32768
SOLVENT CDCl3
NS 8
DS 0
SWH 10330.578 Hz
FIDRES 0.315264 Hz
AQ 1.5859712 sec
RG 50.8
DW 48.400 usec
DE 6.00 usec
TE 296.5 K
D1 1.00000000 sec
TD0 1
======== CHANNEL f1 ========
NUC1 1H
P1 10.50 usec
PL1 0.25 dB
SFO1 500.1330885 MHz
F2 - Processing parameters
SI 16384
SF 500.1300137 MHz
WDW EM
SSB 0
LB 0.30 Hz
GB 0
PC 1.00
ppm

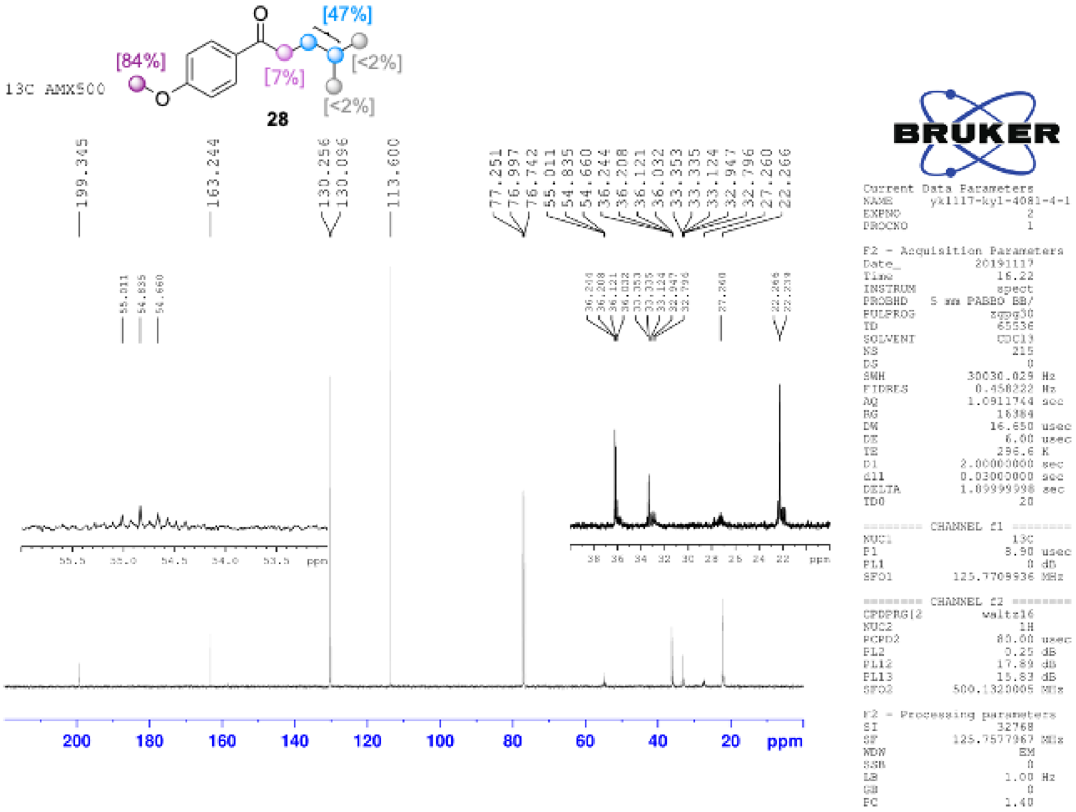
13C AMX500
[84%]
[7%]
[47%]
[<2%]
[<2%]
28
Current Data Parameters
NAME yk1117-kyl-4081-4-1
EXPNO 2
PROCNO 1
F2 - Acquisition Parameters
Date_ 20191117
Time 16.22
INSTRUM spect
PROBHD 5 mm PABBO BB/
PULPROG zgpg30
TD 65536
SOLVENT CDCl3
NS 215
DS 0
SWH 30030.029 Hz
FIDRES 0.458222 Hz
AQ 1.0911744 sec
RG 16384
DW 16.650 usec
DE 6.00 usec
TE 296.6 K
D1 2.00000000 sec
d11 0.03000000 sec
DELTA 1.89999998 sec
TD0 20
======== CHANNEL f1 ========
NUC1 13C
P1 9.90 usec
PL1 0 dB
SFO1 125.7709936 MHz
======== CHANNEL f2 ========
CPDPRG[2 waltz16
NUC2 1H
PCPD2 80.00 usec
PL2 0.25 dB
PL12 17.89 dB
PL13 15.83 dB
SFO2 500.1320005 MHz
F2 - Processing parameters
SI 32768
SF 125.7577967 MHz
WDW EM
SSB 0
LB 1.00 Hz
GB 0
PC 1.40
ppm

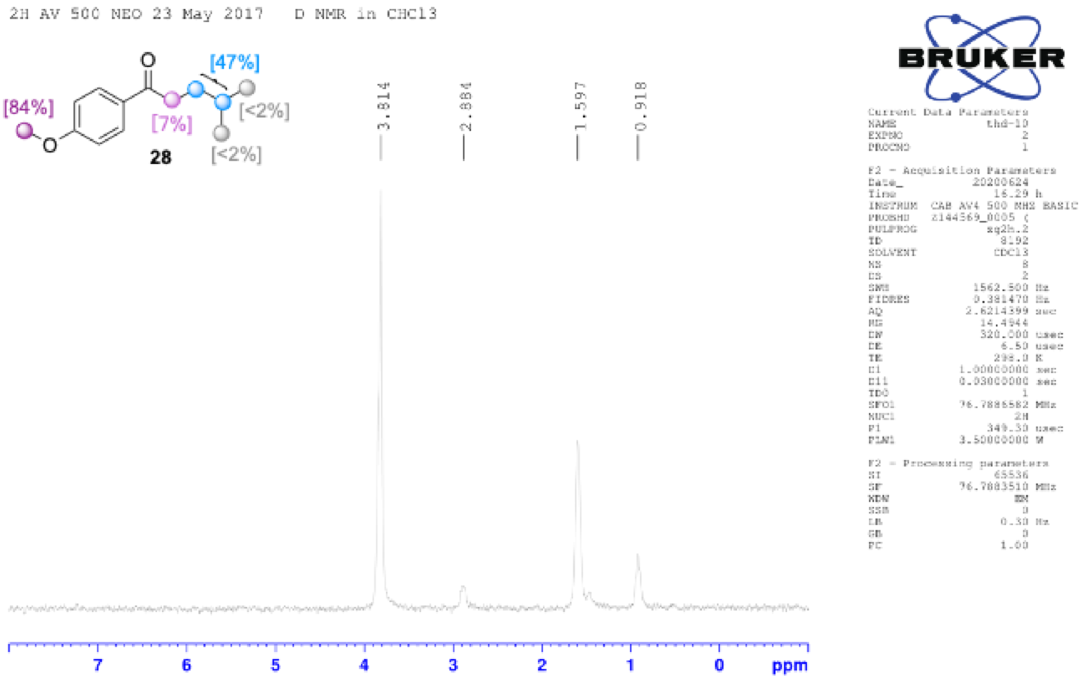
2H AV 500 NEO 23 May 2017 D NMR in CHCl3
[84%]
[7%]
[47%]
[<2%]
[<2%]
28
3.814
2.884
1.597
0.918
Current Data Parameters
NAME thd-10
EXPNO 2
PROCNO 1
F2 - Acquisition Parameters
Date_ 20200624
SOLVENT CDCl3
F2 - Processing parameters
ppm

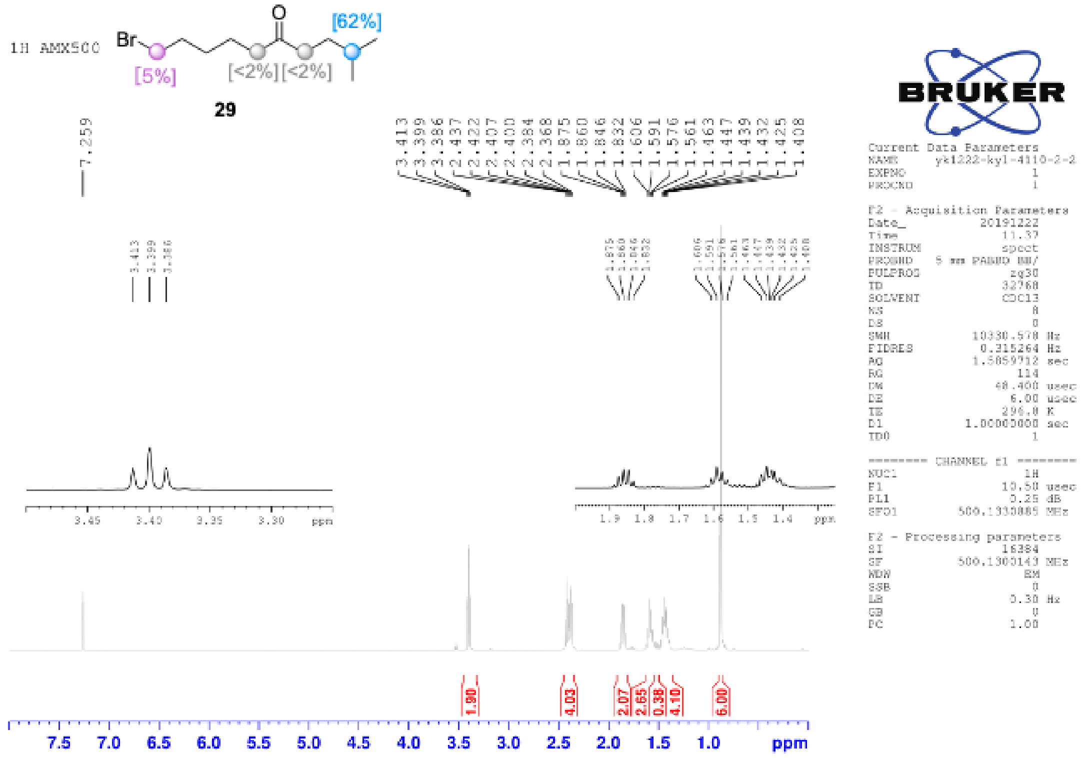
1H AMX500
Br
[5%]
[<2%][<2%]
[62%]
29
7.259
3.413
3.399
3.386
1.90
4.03
2.07
2.65
0.38
4.10
6.00
Current Data Parameters
NAME yk1222-kyl-4110-2-2
EXPNO 1
PROCNO 1
F2 - Acquisition Parameters
Date_ 20191222
SOLVENT CDCl3
======== CHANNEL f1 ========
NUC1 1H
F2 - Processing parameters
ppm

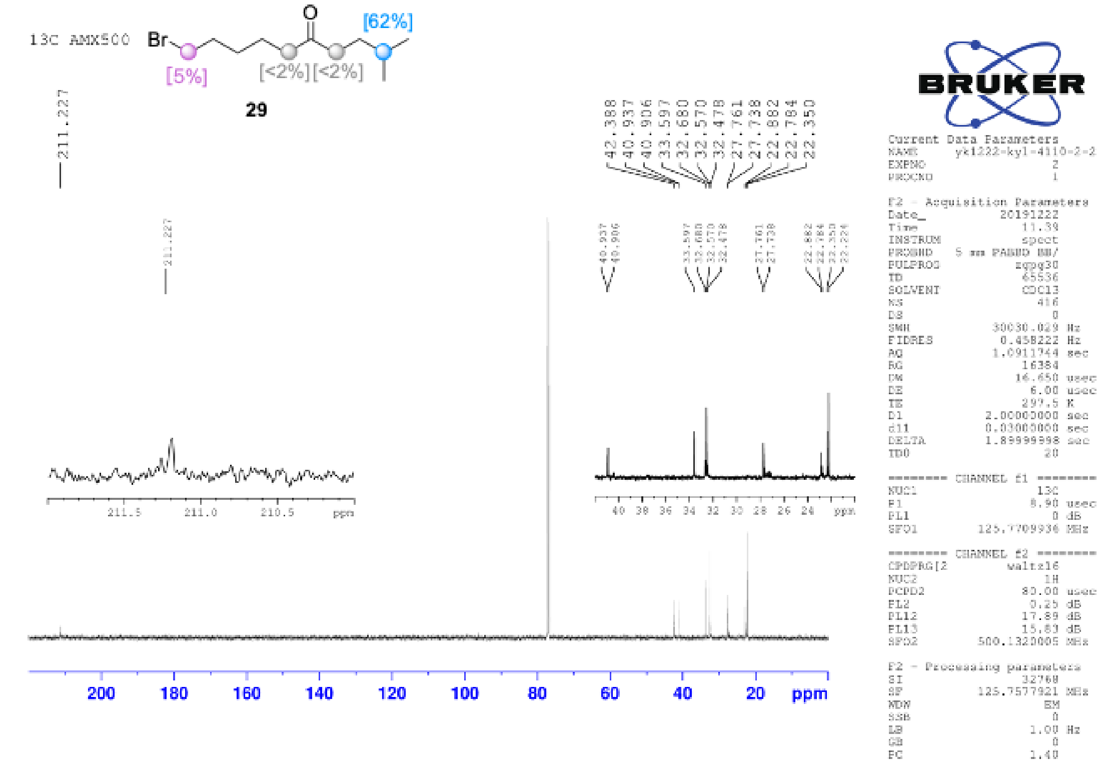
13C AMX500
Br
O
[62%]
[5%]
[<2%][<2%]
29
211.227
42.388
40.937
40.906
33.597
32.680
32.570
32.478
27.761
27.738
22.882
22.784
22.350
BRUKER
Current Data Parameters
NAME yk1222-ky1-4110-2-2
EXPNO 2
PROCNO 1
F2 - Acquisition Parameters
Date_ 20191222
INSTRUM spect
PULPROG zgpg30
TD 65536
SOLVENT CDCl3
NUC1 13C
SFO1 125.7709936 MHz
NUC2 1H
SFO2 500.1320005 MHz
SF 125.7577921 MHz
ppm

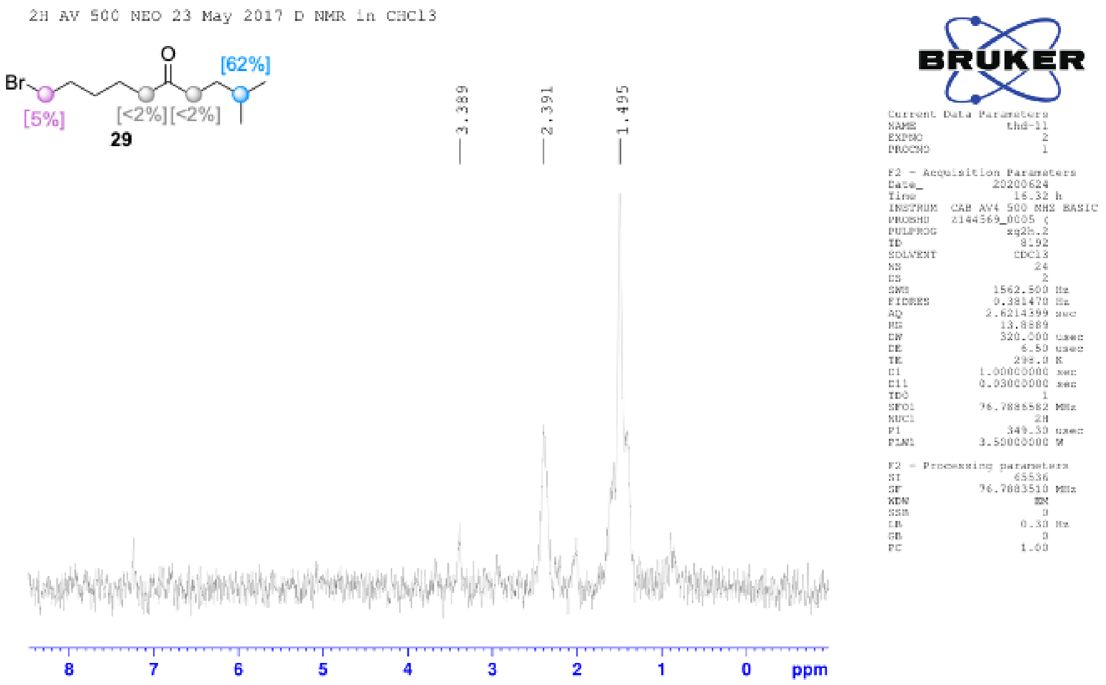
2H AV 500 NEO 23 May 2017 D NMR in CHCl3
Br
O
[62%]
[5%]
[<2%][<2%]
29
3.389
2.391
1.495
BRUKER
Current Data Parameters
NAME thd-11
EXPNO 2
PROCNO 1
F2 - Acquisition Parameters
Date_ 20200624
INSTRUM CAB AV4 500 MHZ BASIC
PULPROG zg2h.2
TD 8192
SOLVENT CDCl3
NUC1 2H
SFO1 76.7886582 MHz
SF 76.7883510 MHz
ppm

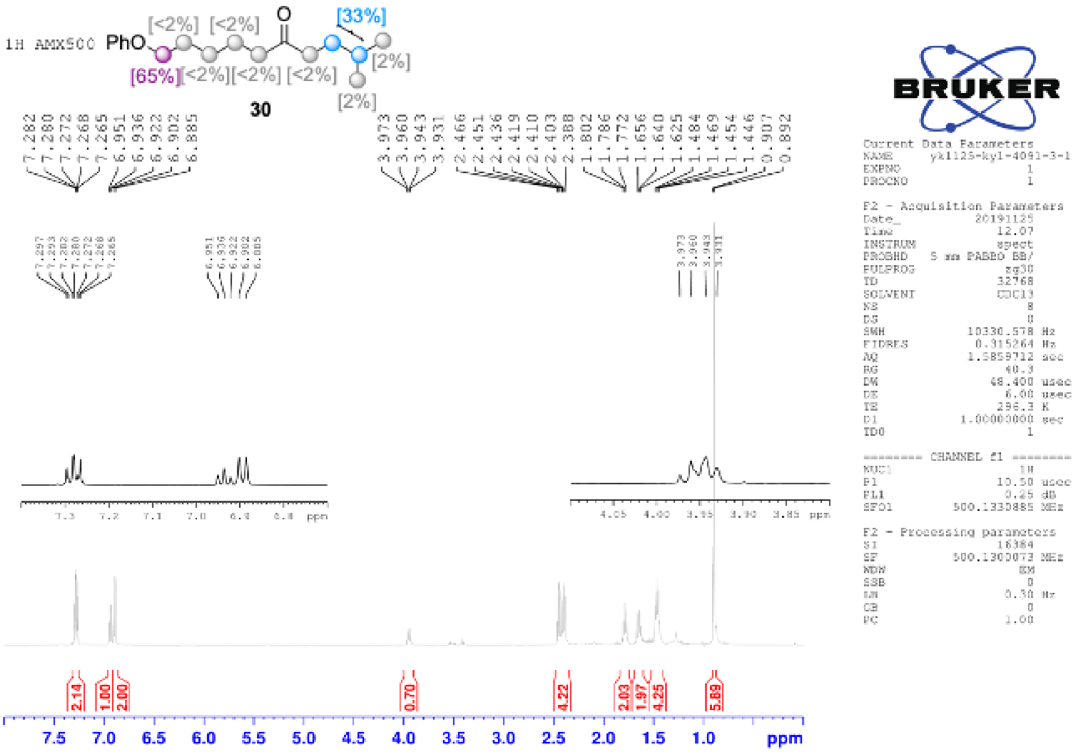
1H AMX500
PhO
[<2%] [<2%]
[33%]
[2%]
[65%][<2%][<2%] [<2%]
[2%]
30
7.282 7.280 7.272 7.268 7.265 6.951 6.936 6.922 6.902 6.885
3.973 3.960 3.943 3.931 2.466 2.451 2.436 2.419 2.410 2.403 2.388 1.802 1.786 1.772 1.656 1.640 1.625 1.484 1.469 1.454 1.446 0.907 0.892
2.14 1.00 2.00 0.70 4.22 2.03 1.97 4.25 5.89
ppm
BRUKER

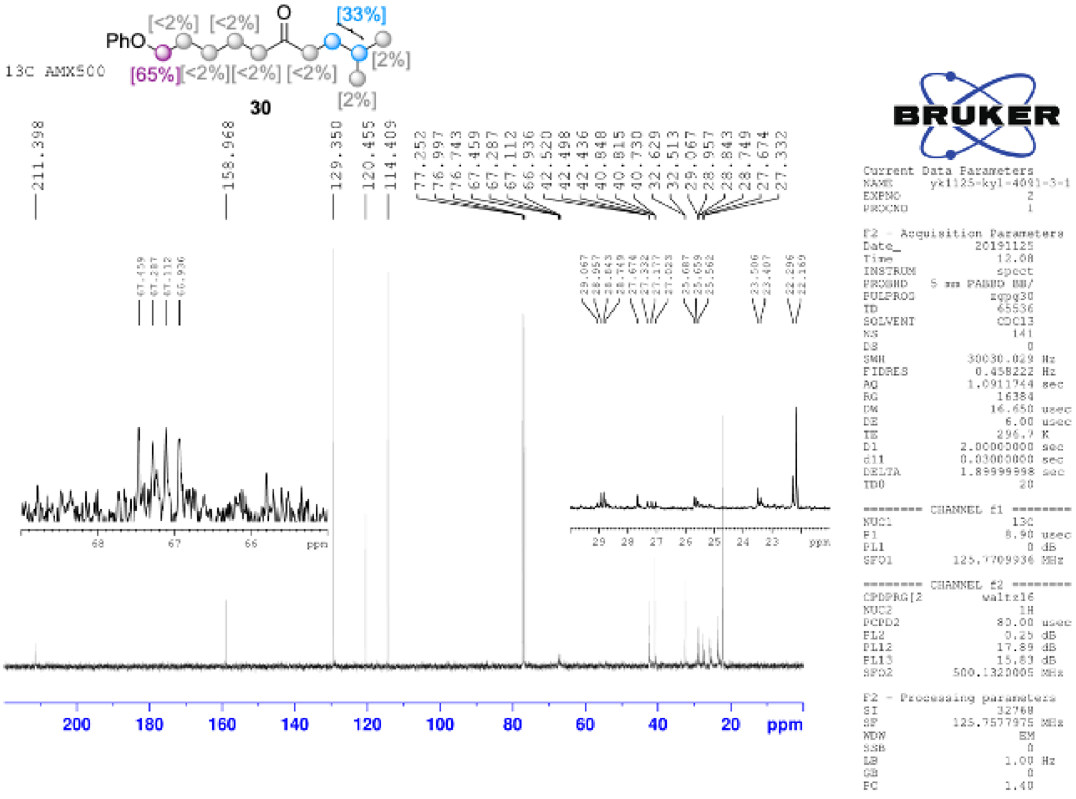
13C AMX500
PhO
[<2%] [<2%]
[33%]
[2%]
[65%][<2%][<2%] [<2%]
[2%]
30
211.398 158.968 129.350 120.455 114.409 77.252 76.997 76.743 67.459 67.287 67.112 66.936 42.520 42.498 42.436 40.848 40.815 40.730 32.629 32.513 29.067 28.957 28.843 28.749 27.674 27.332
ppm
BRUKER

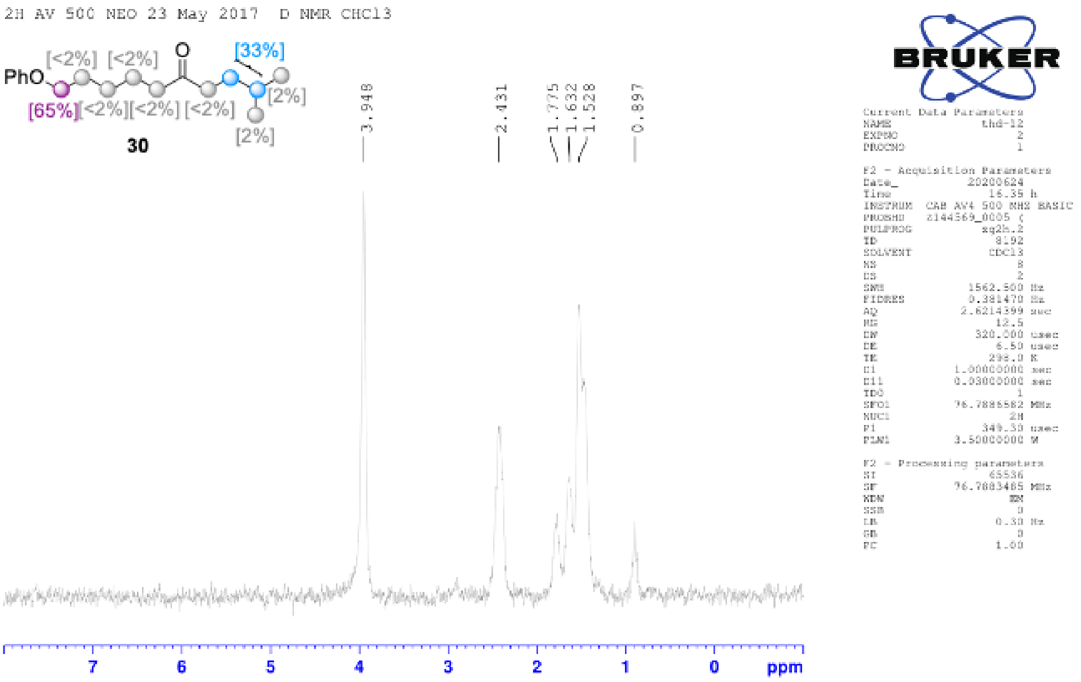

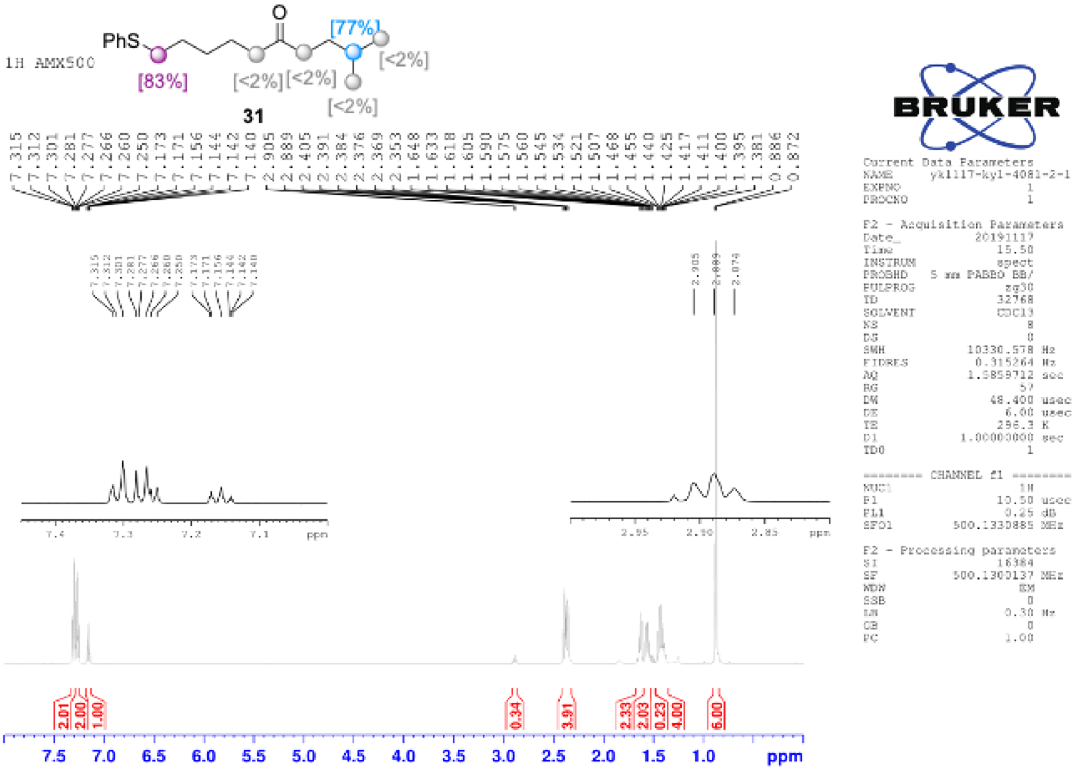

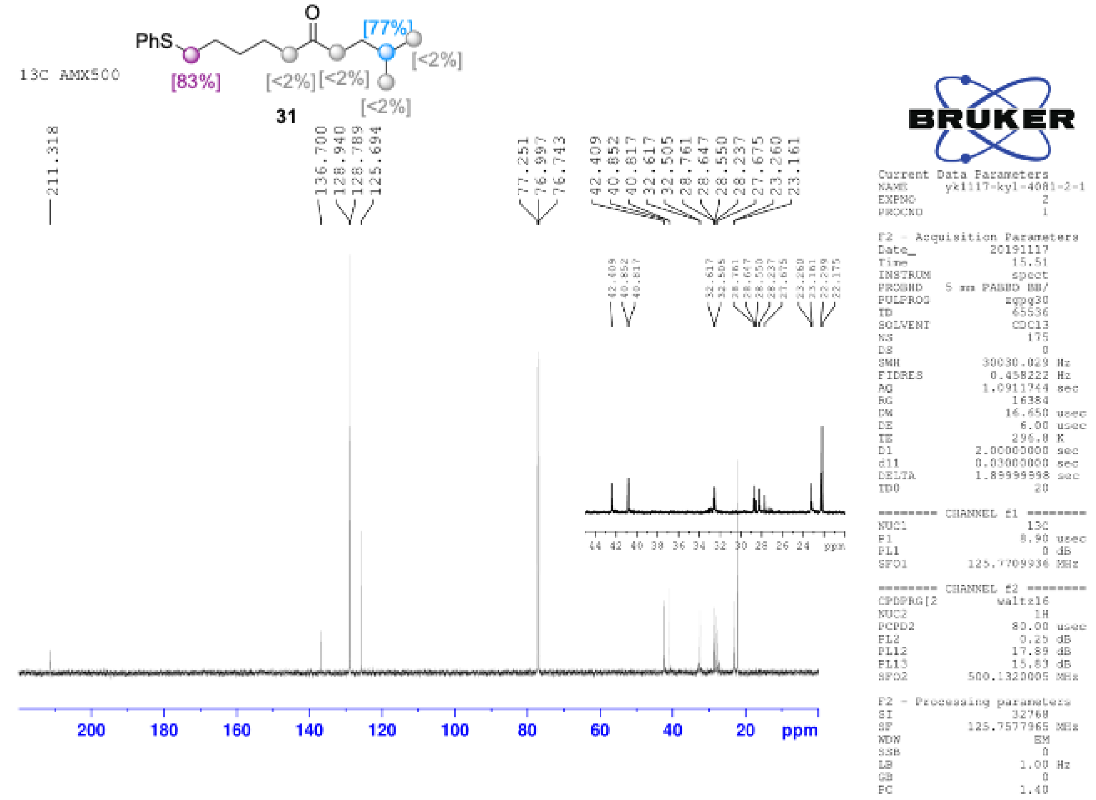
13C AMX500
PhS
[83%]
[<2%][<2%]
[77%]
[<2%]
[<2%]
31
211.318
136.700
128.940
128.789
125.694
77.251
76.997
76.743
42.409
40.852
40.817
32.617
32.505
28.761
28.647
28.550
28.237
27.675
23.260
23.161
BRUKER
200 180 160 140 120 100 80 60 40 20 ppm

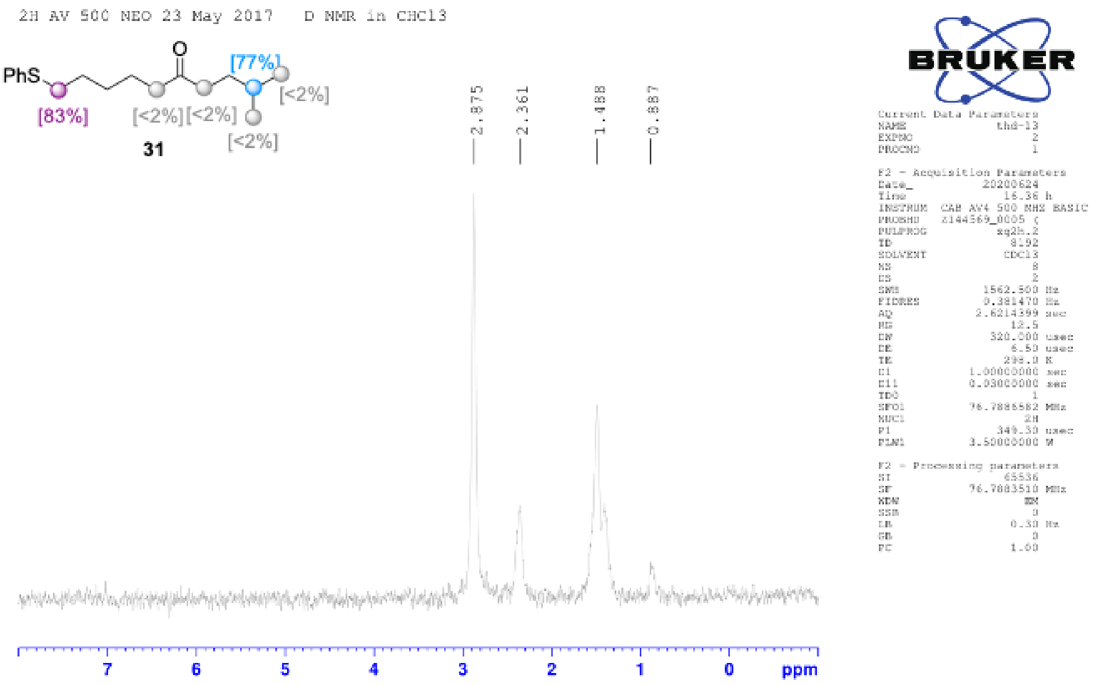
2H AV 500 NEO 23 May 2017 D NMR in CHCl3
PhS
[83%]
[<2%][<2%]
[77%]
[<2%]
[<2%]
31
2.875
2.361
1.488
0.887
BRUKER
7 6 5 4 3 2 1 0 ppm

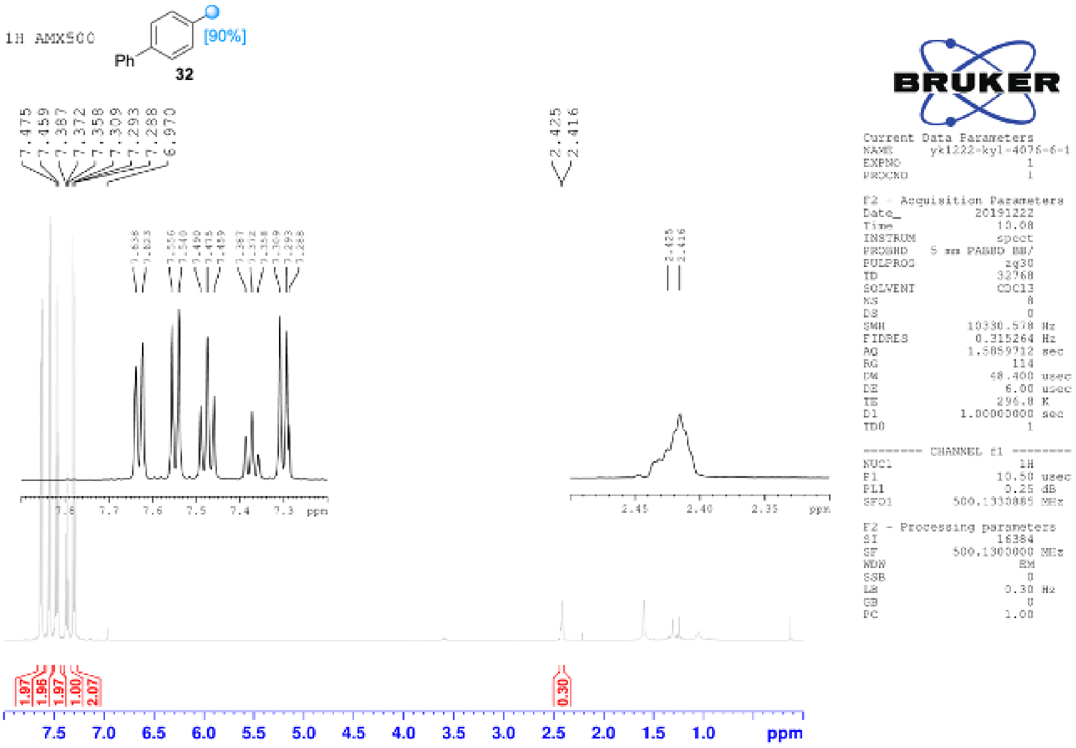
1H AMX500
[90%]
Ph
32
BRUKER
Current Data Parameters
F2 - Acquisition Parameters
F2 - Processing parameters
ppm

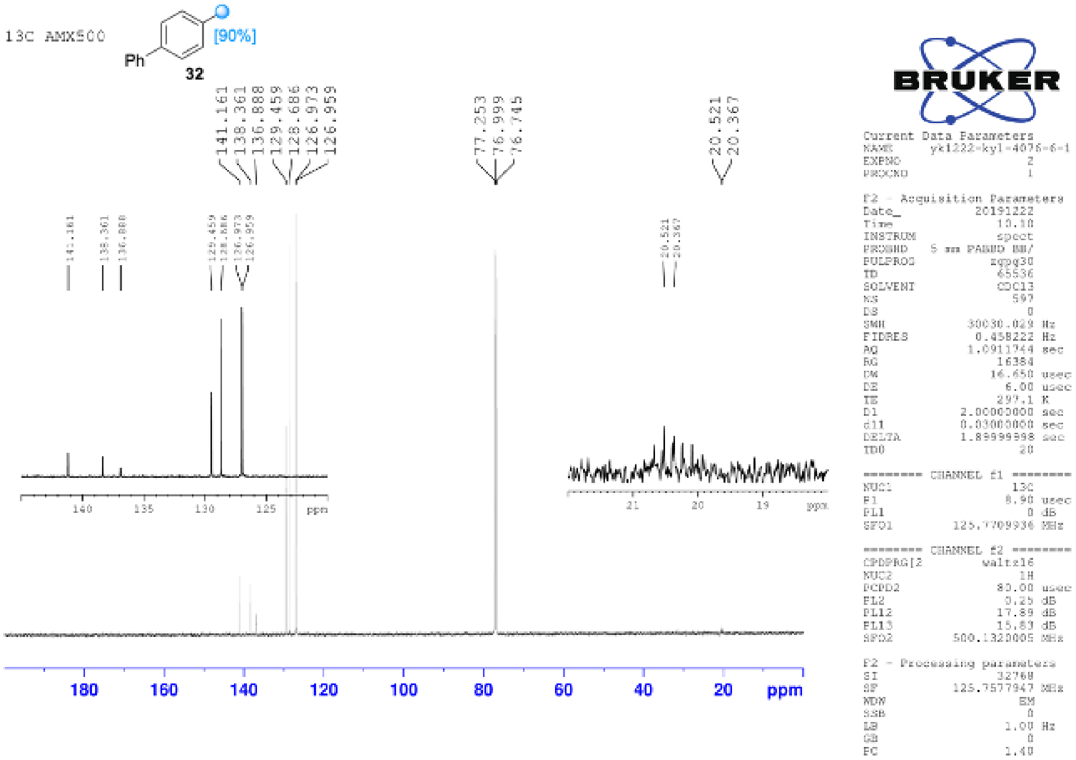
13C AMX500
[90%]
Ph
32
BRUKER
Current Data Parameters
F2 - Acquisition Parameters
F2 - Processing parameters
ppm

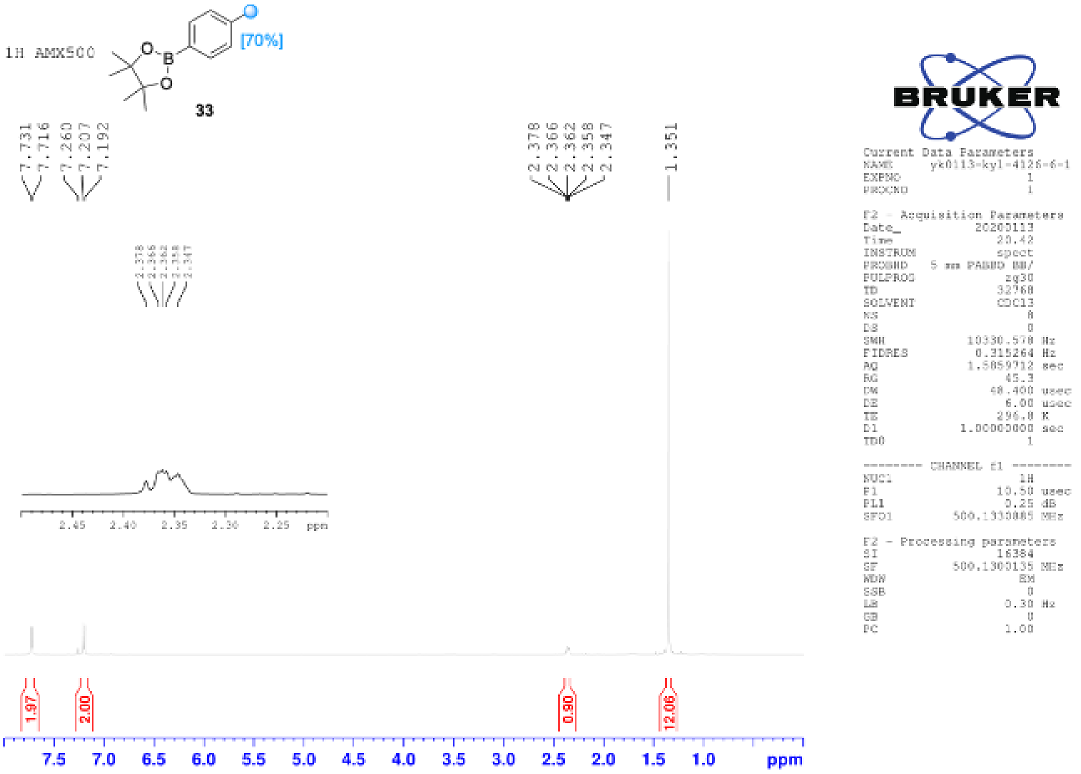

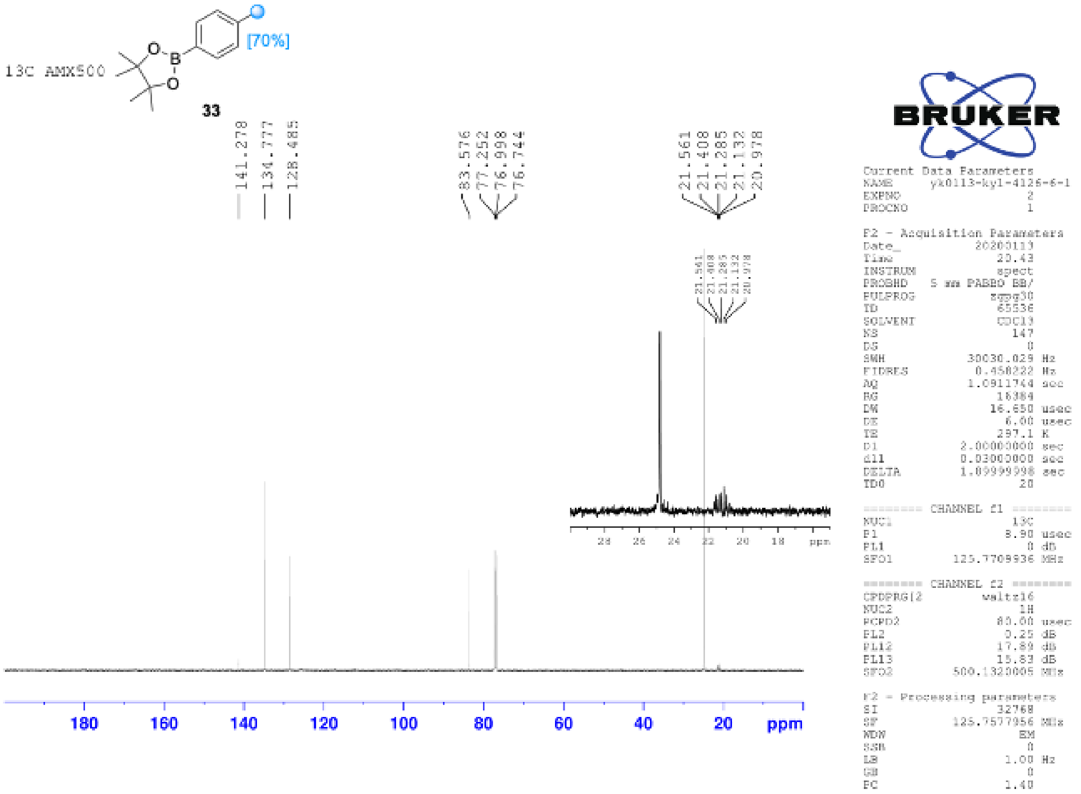

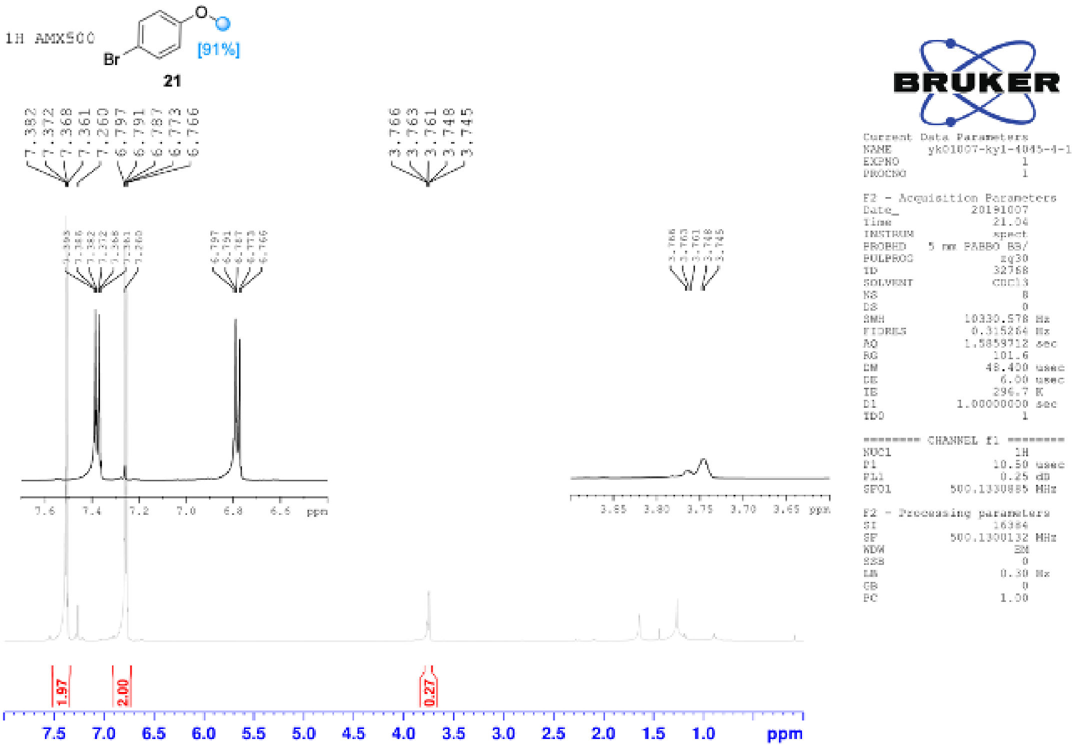
1H AMX500
Br
O
[91%]
21
7.382
7.372
7.368
7.361
7.260
6.797
6.791
6.787
6.773
6.766
3.766
3.763
3.761
3.748
3.745
BRUKER
1.97
2.00
0.27
7.5
7.0
6.5
6.0
5.5
5.0
4.5
4.0
3.5
3.0
2.5
2.0
1.5
1.0
ppm

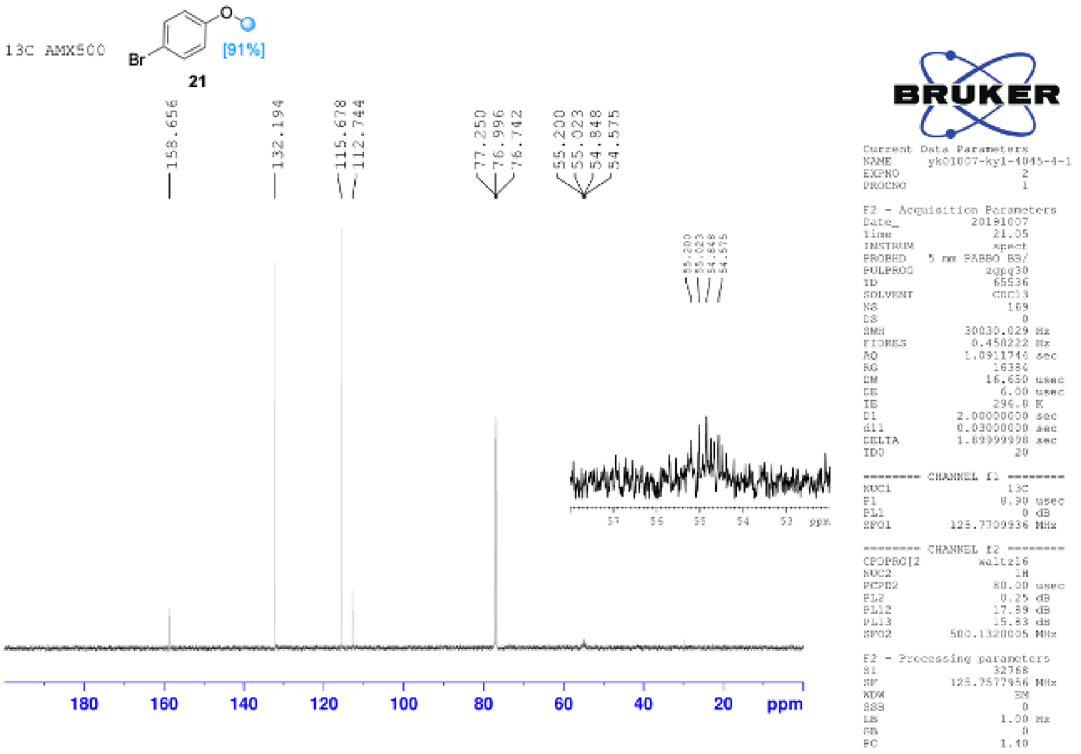
13C AMX500
Br
O
[91%]
21
158.656
132.194
115.678
112.744
77.250
76.996
76.742
55.200
55.023
54.848
54.575
BRUKER
180
160
140
120
100
80
60
40
20
ppm

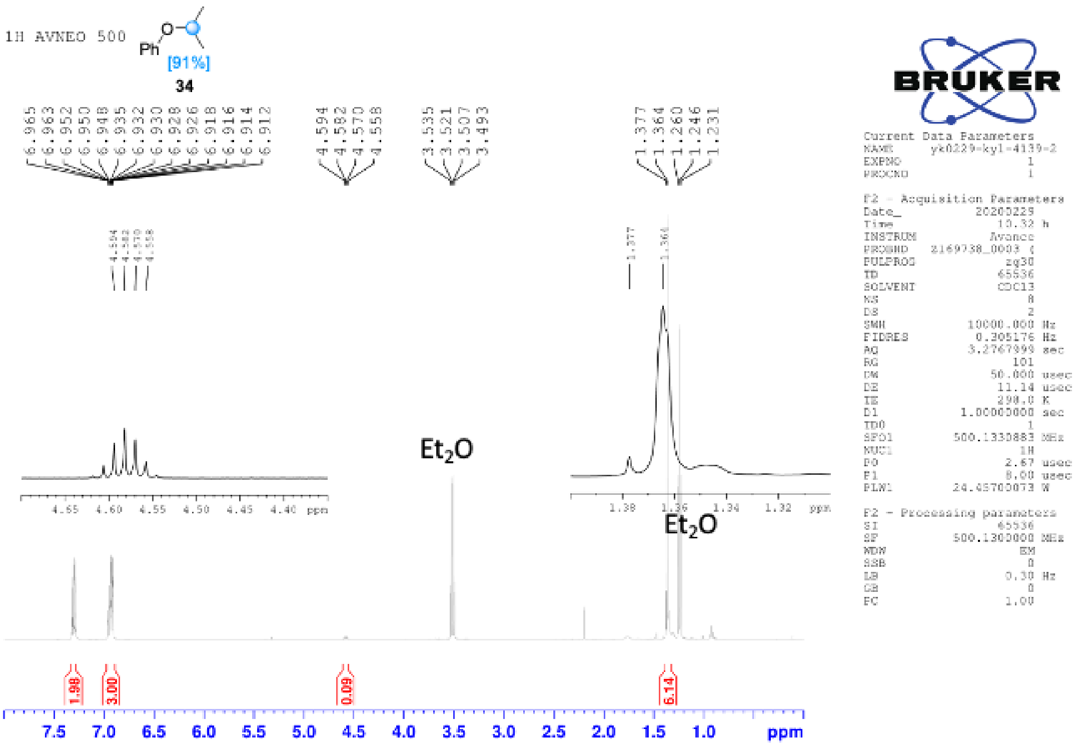
1H AVNEO 500
[91%]
34
Et2O
Et2O
1.98
3.00
0.09
6.14
ppm

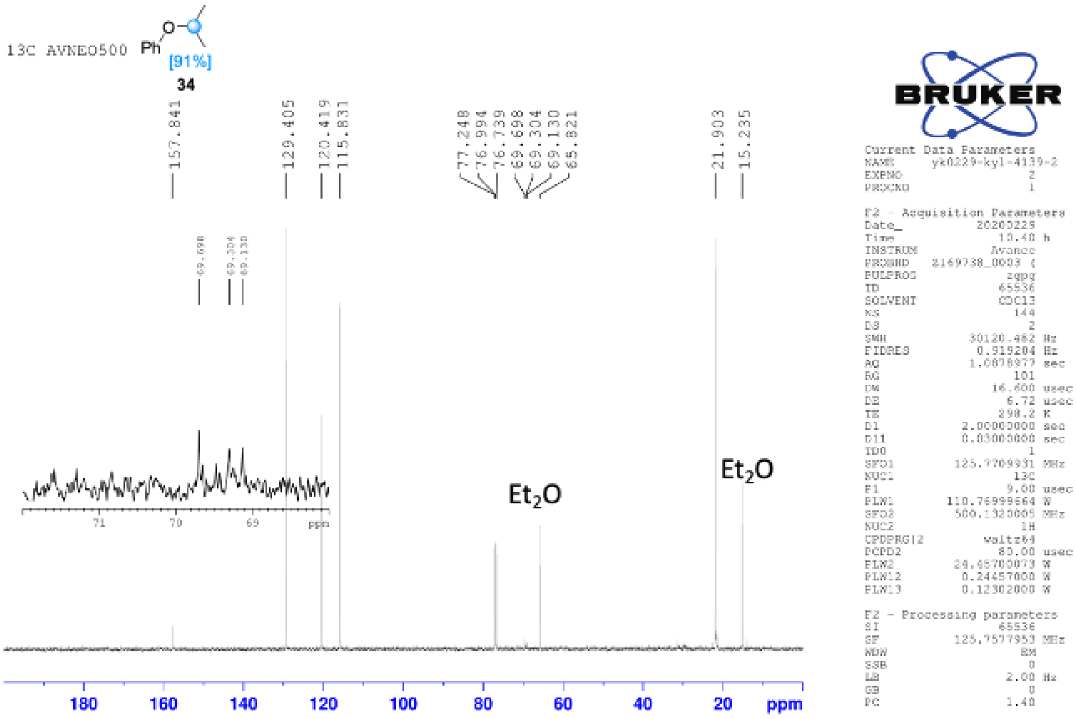
13C AVNEO500
[91%]
34
Et2O
Et2O
ppm

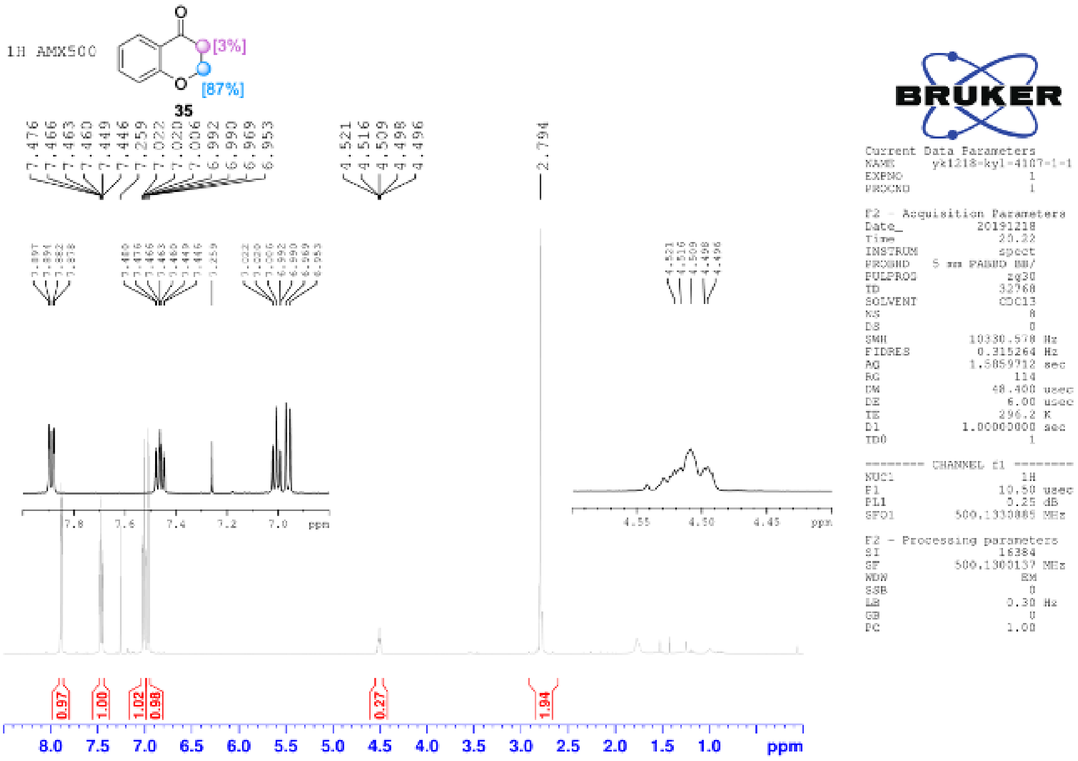
1H AMX500
[3%]
[87%]
35
BRUKER
ppm

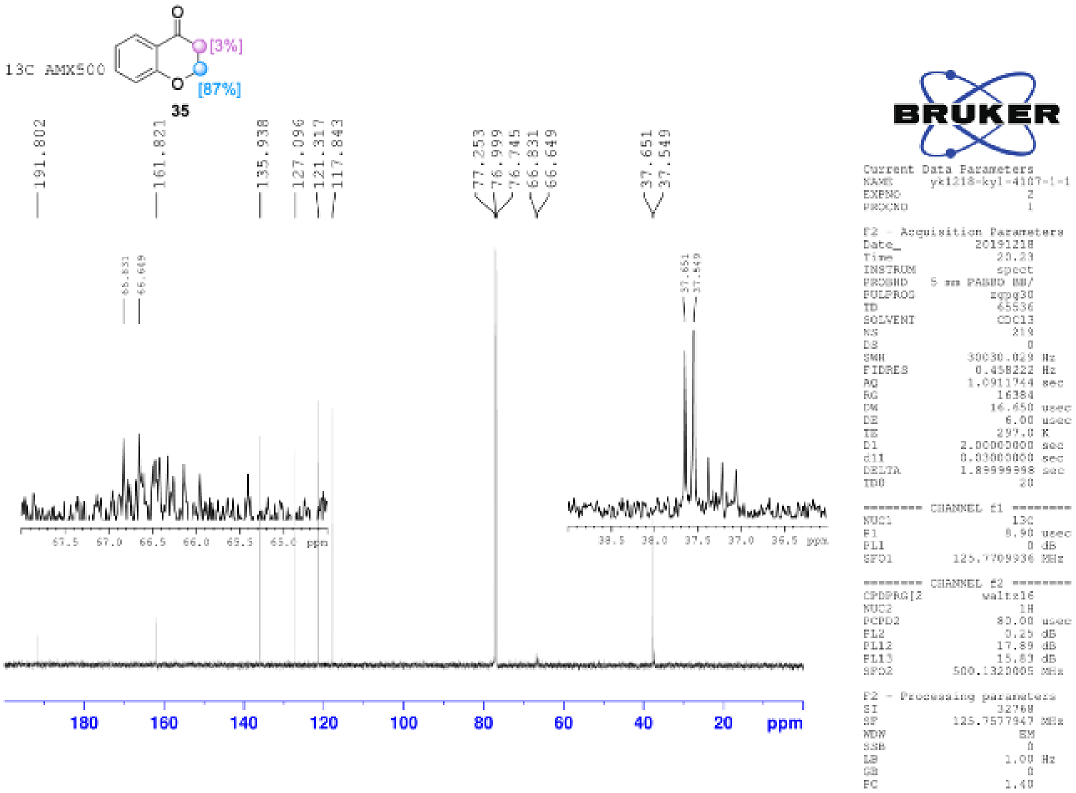
13C AMX500
[3%]
[87%]
35
BRUKER
ppm

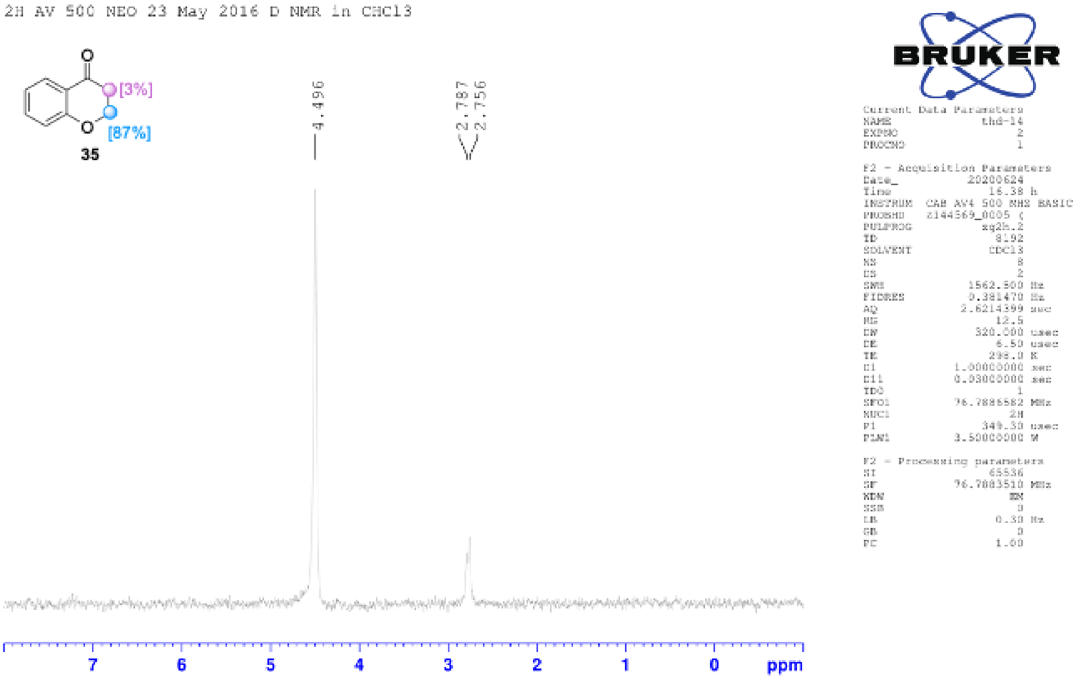
2H AV 500 NEO 23 May 2016 D NMR in CHCl3
[3%]
[87%]
35
4.496
2.787
2.756
ppm
Current Data Parameters
NAME thd-14
EXPNO 2
PROCNO 1
F2 - Acquisition Parameters
Date_ 20200624
SOLVENT CDCl3
NS 8
F2 - Processing parameters
SI 65536

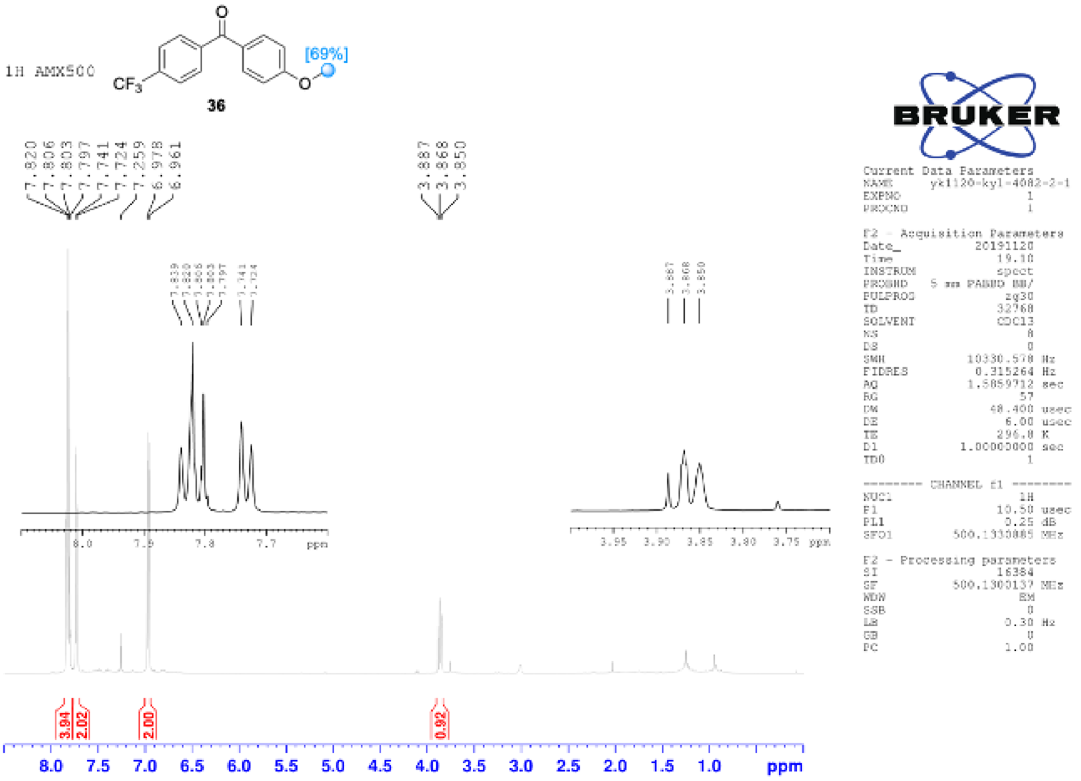
1H AMX500
CF3
[69%]
36
7.820
7.806
7.803
7.797
7.741
7.724
7.259
6.978
6.961
3.887
3.868
3.850
3.94
2.02
2.00
0.92
ppm
Current Data Parameters
NAME yk1120-ky1-4082-2-1
EXPNO 1
PROCNO 1
F2 - Acquisition Parameters
Date_ 20191120
SOLVENT CDCl3
NS 8
F2 - Processing parameters
SI 16384

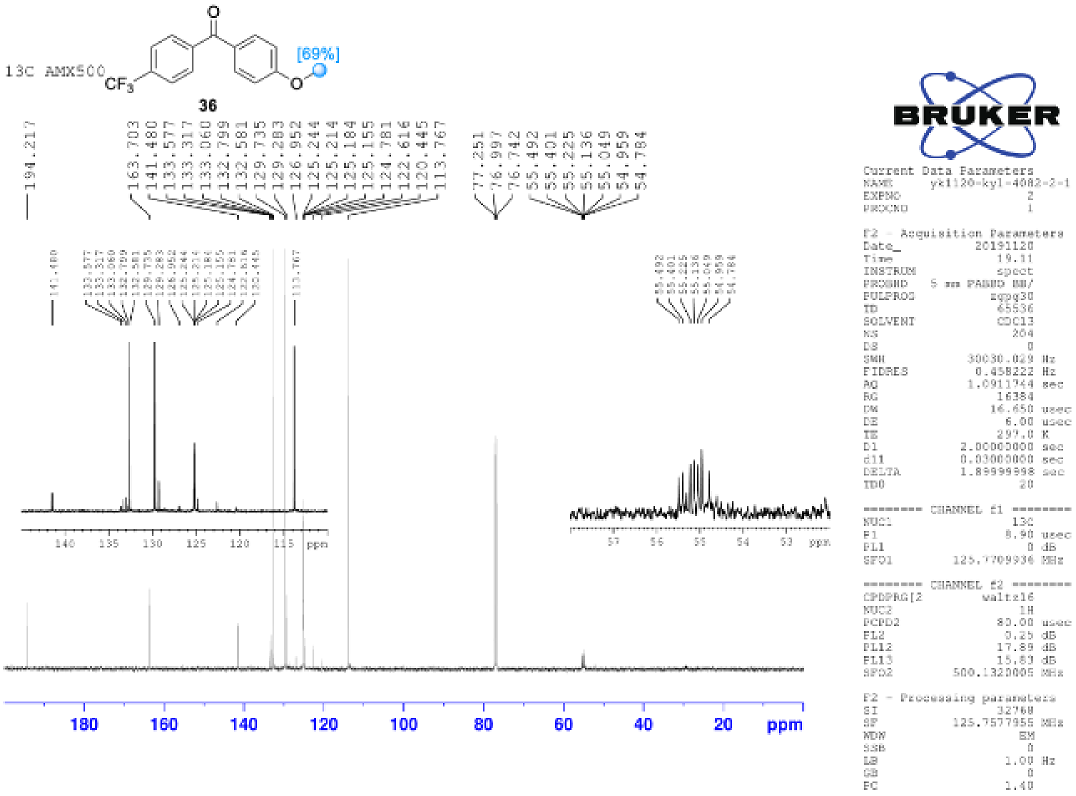

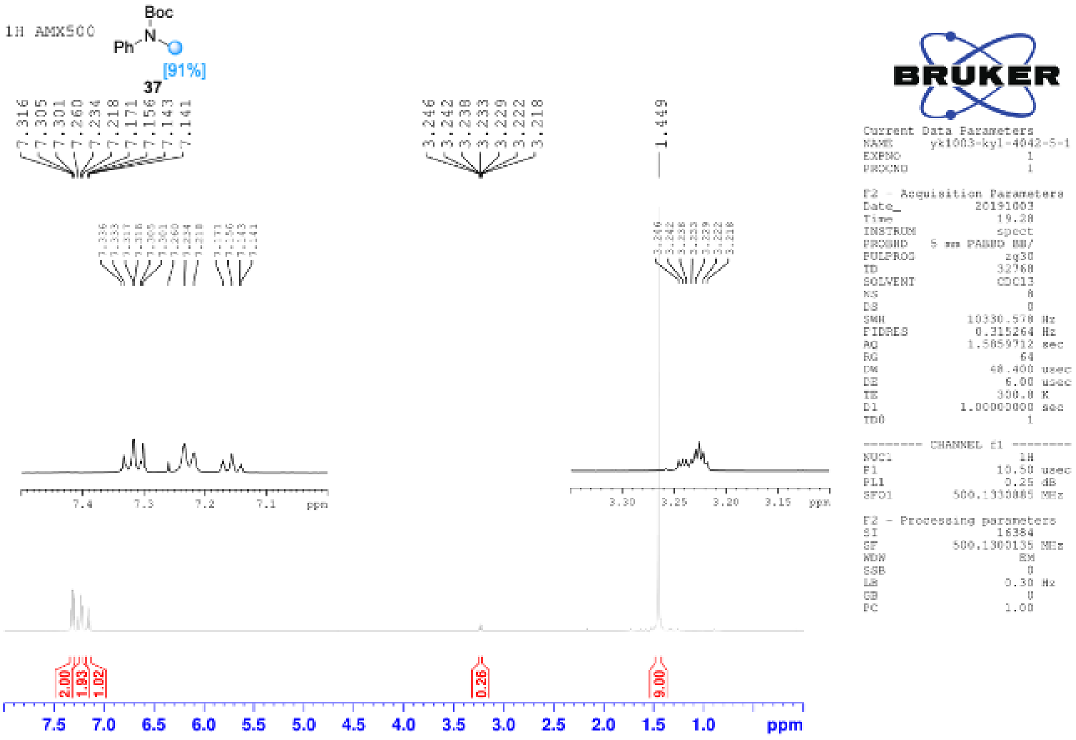

13C AMX500

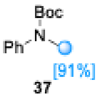

37

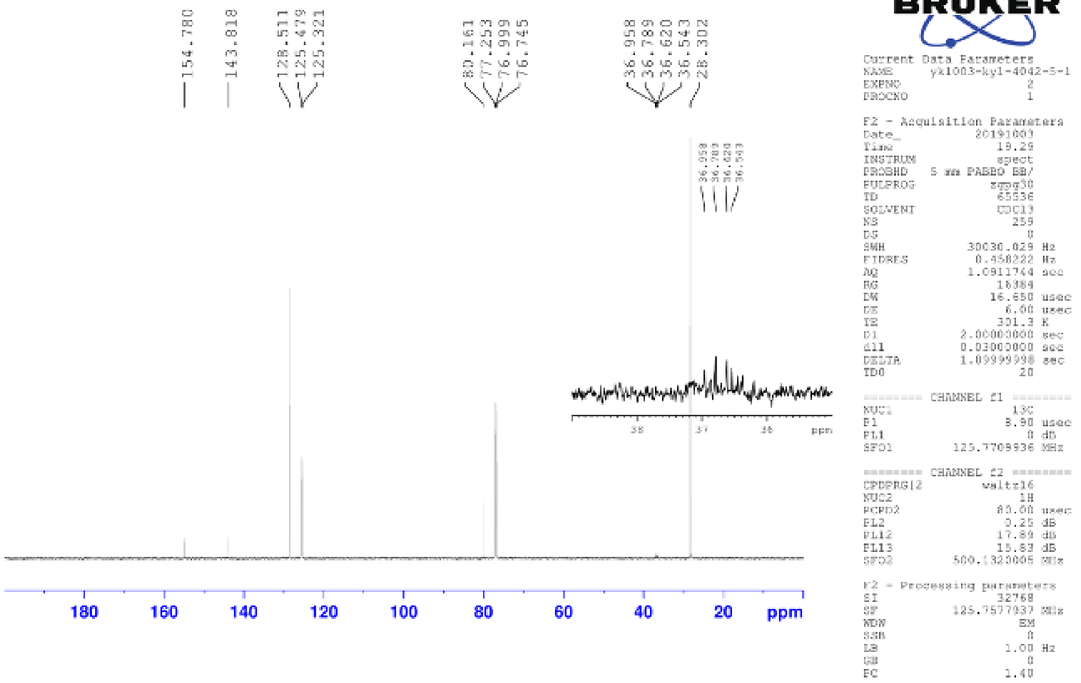

1H AMX500

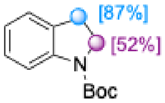

38

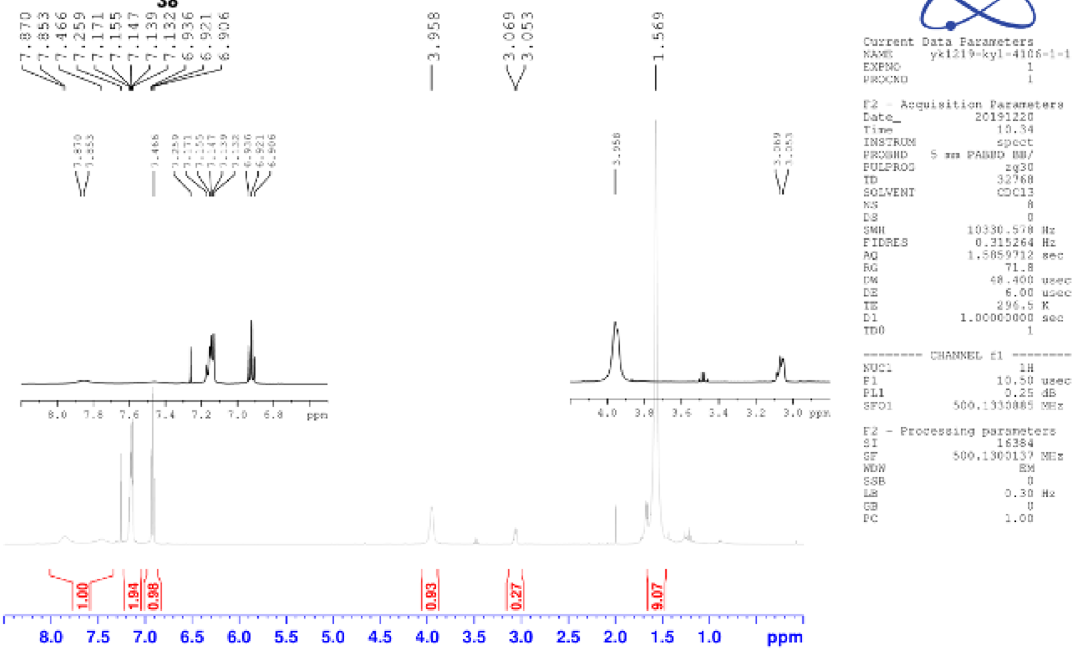

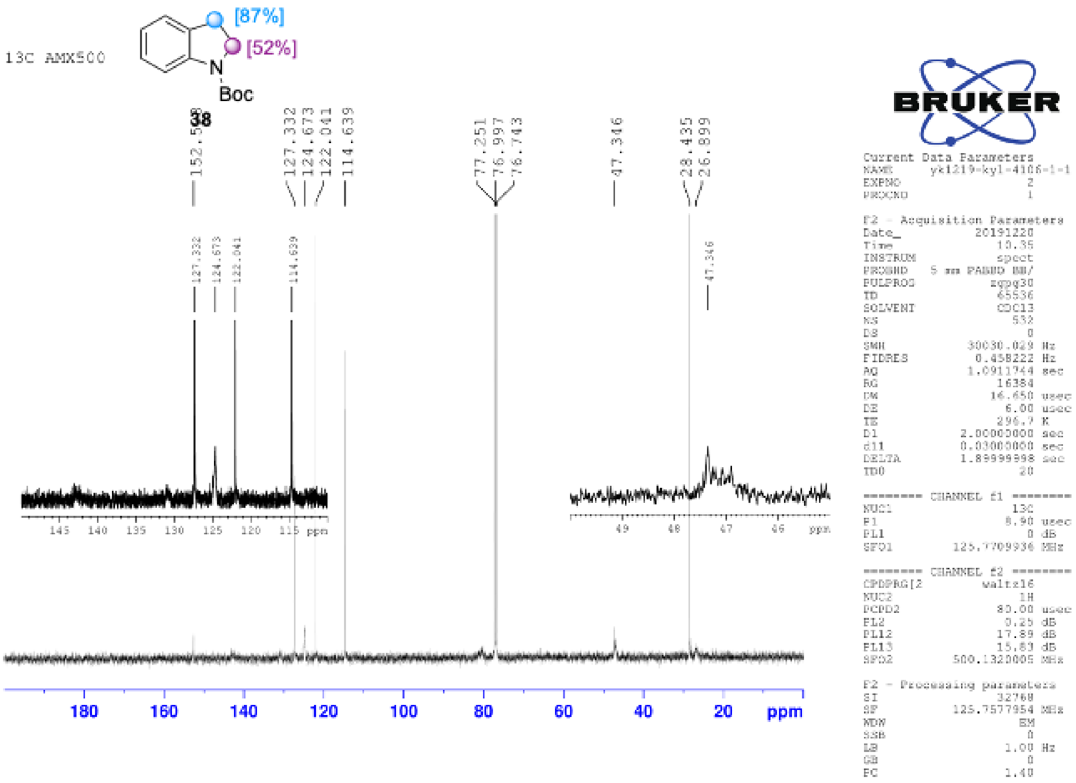
13C AMX500
[87%]
[52%]
N
Boc
38
152.58
127.332
124.673
122.041
114.639
77.251
76.997
76.743
47.346
28.435
26.899
BRUKER
Current Data Parameters
ppm

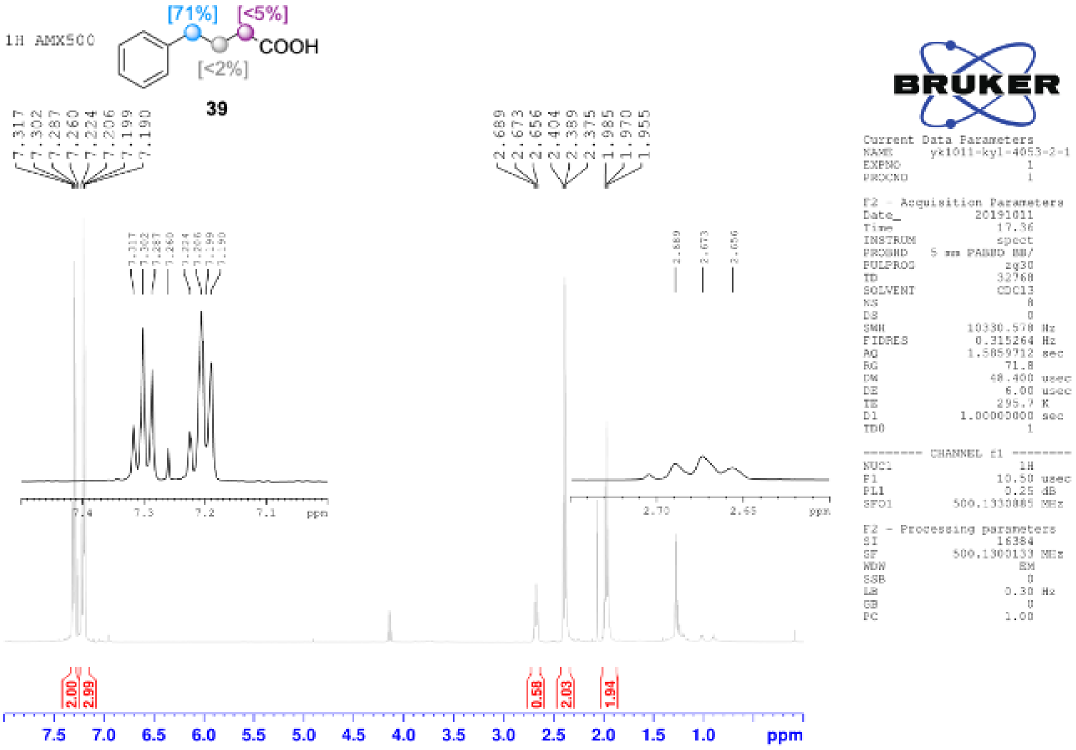
1H AMX500
[71%]
[<5%]
[<2%]
COOH
39
7.317
7.302
7.287
7.260
7.224
7.206
7.199
7.190
2.689
2.673
2.656
2.404
2.389
2.375
1.985
1.970
1.955
2.00
2.99
0.58
2.03
1.94
BRUKER
Current Data Parameters
ppm

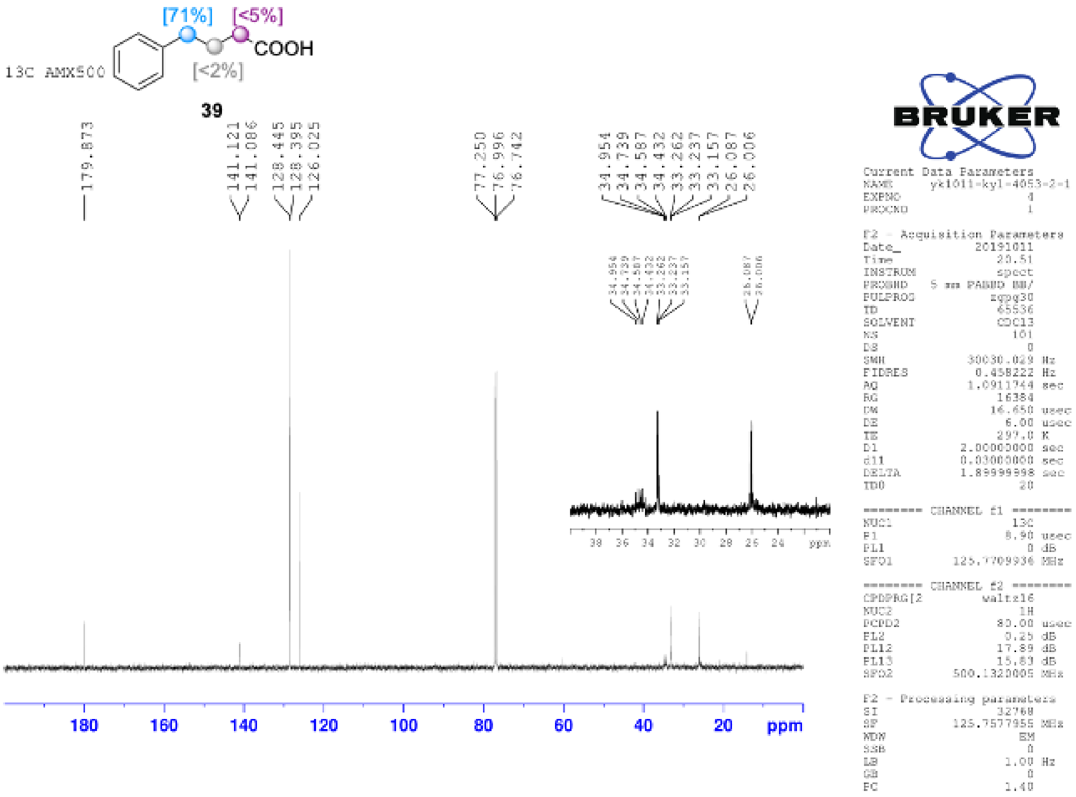
13C AMX500
[71%]
[<5%]
[<2%]
COOH
39
179.873
141.121
141.086
128.445
128.395
126.025
77.250
76.996
76.742
34.954
34.739
34.587
34.432
33.262
33.237
33.157
26.087
26.006
ppm
Current Data Parameters
NAME yk1011-ky1-4053-2-1
EXPNO 4
PROCNO 1
F2 - Acquisition Parameters
Date_ 20191011
Time 20.51
INSTRUM spect
PROBHD 5 mm PABBO BB/
PULPROG zgpg30
TD 65536
SOLVENT CDCl3
NS 101
DS 0
SWH 30030.029 Hz
FIDRES 0.458222 Hz
AQ 1.0911744 sec
RG 16384
DW 16.650 usec
DE 6.00 usec
TE 297.0 K
D1 2.00000000 sec
d11 0.03000000 sec
DELTA 1.89999998 sec
TD0 20
======== CHANNEL f1 ========
NUC1 13C
P1 8.90 usec
PL1 0 dB
SFO1 125.7709936 MHz
======== CHANNEL f2 ========
CPDPRG[2 waltz16
NUC2 1H
PCPD2 80.00 usec
PL2 0.25 dB
PL12 17.89 dB
PL13 15.83 dB
SFO2 500.1320005 MHz
F2 - Processing parameters
SI 32768
SF 125.7577955 MHz
WDW EM
SSB 0
LB 1.00 Hz
GB 0
PC 1.40

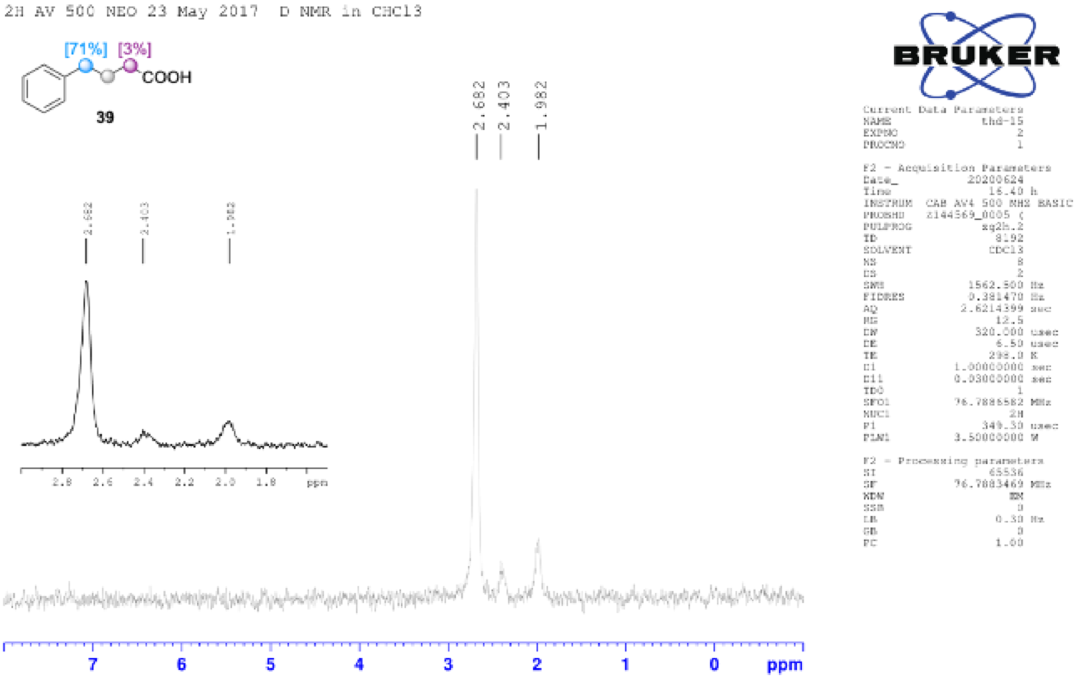
2H AV 500 NEO 23 May 2017 D NMR in CHCl3
[71%]
[3%]
COOH
39
2.682
2.403
1.982
ppm
Current Data Parameters
NAME thd-15
EXPNO 2
PROCNO 1
F2 - Acquisition Parameters
Date_ 20200624
Time 16.40 h
INSTRUM CAB AV4 500 MHZ BASIC
PROBHD Z144569_0005 (
PULPROG zg2h.2
TD 8192
SOLVENT CDCl3
NS 8
DS 2
SWH 1562.500 Hz
FIDRES 0.381470 Hz
AQ 2.6214399 sec
RG 12.5
DW 320.000 usec
DE 6.50 usec
TE 298.0 K
D1 1.00000000 sec
D11 0.03000000 sec
TD0 1
SFO1 76.7886582 MHz
NUC1 2H
P1 349.30 usec
PLW1 3.50000000 W
F2 - Processing parameters
SI 65536
SF 76.7883469 MHz
WDW EM
SSB 0
LB 0.30 Hz
GB 0
PC 1.00

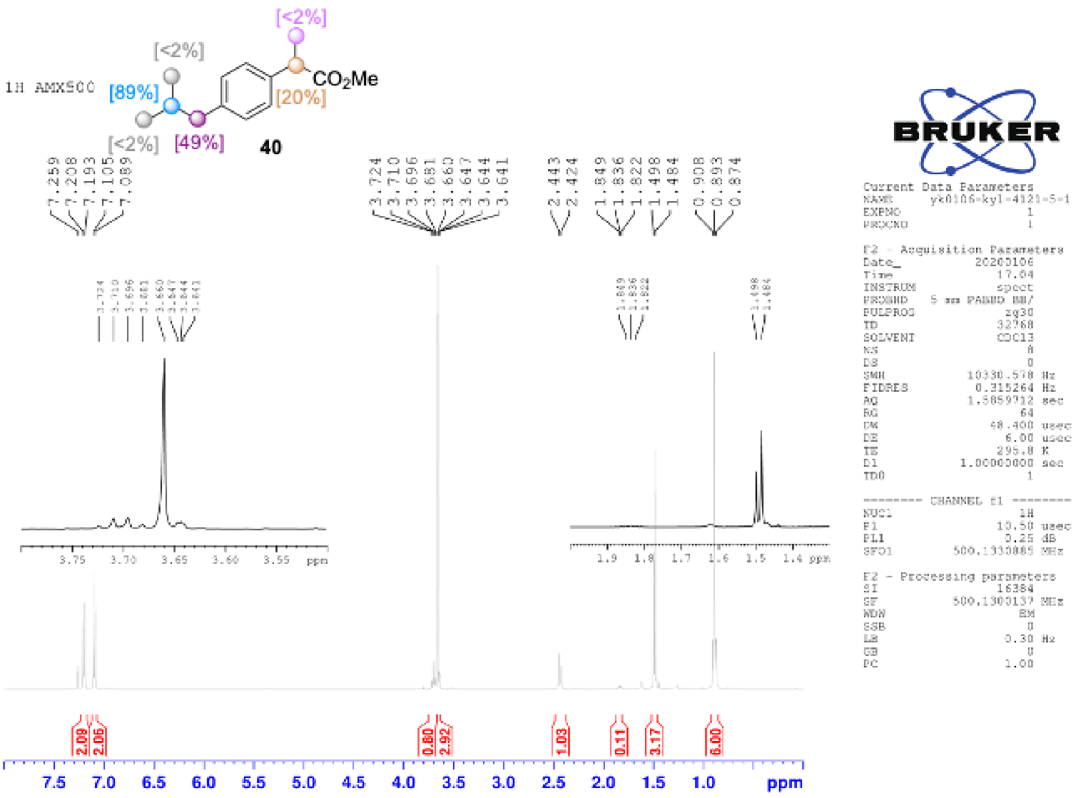

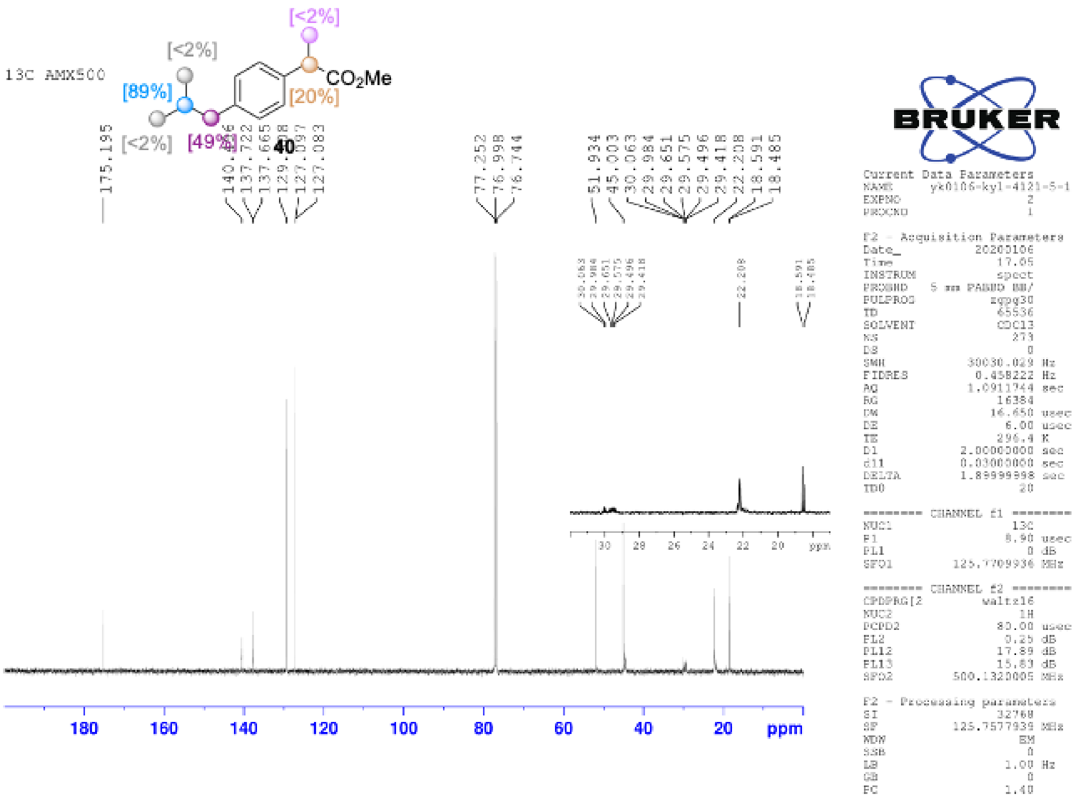

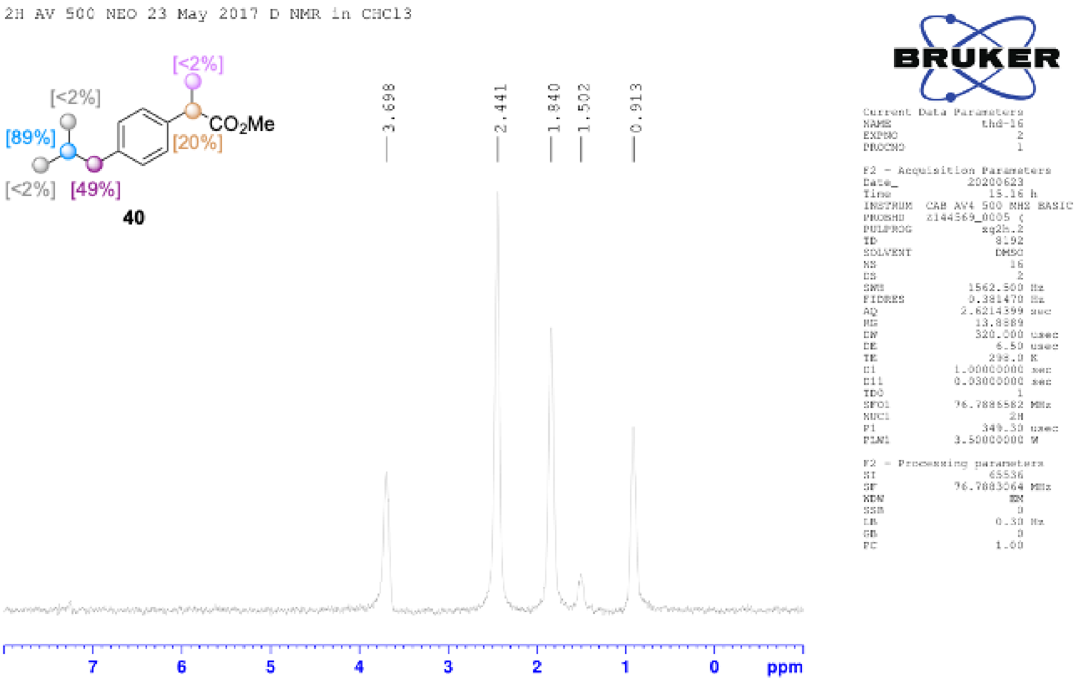
2H AV 500 NEO 23 May 2017 D NMR in CHCl3
[<2%]
[<2%]
[89%]
CO2Me
[20%]
[<2%]
[49%]
40
3.698
2.441
1.840
1.502
0.913
BRUKER
Current Data Parameters
F2 - Acquisition Parameters
F2 - Processing parameters
7
6
5
4
3
2
1
0
ppm

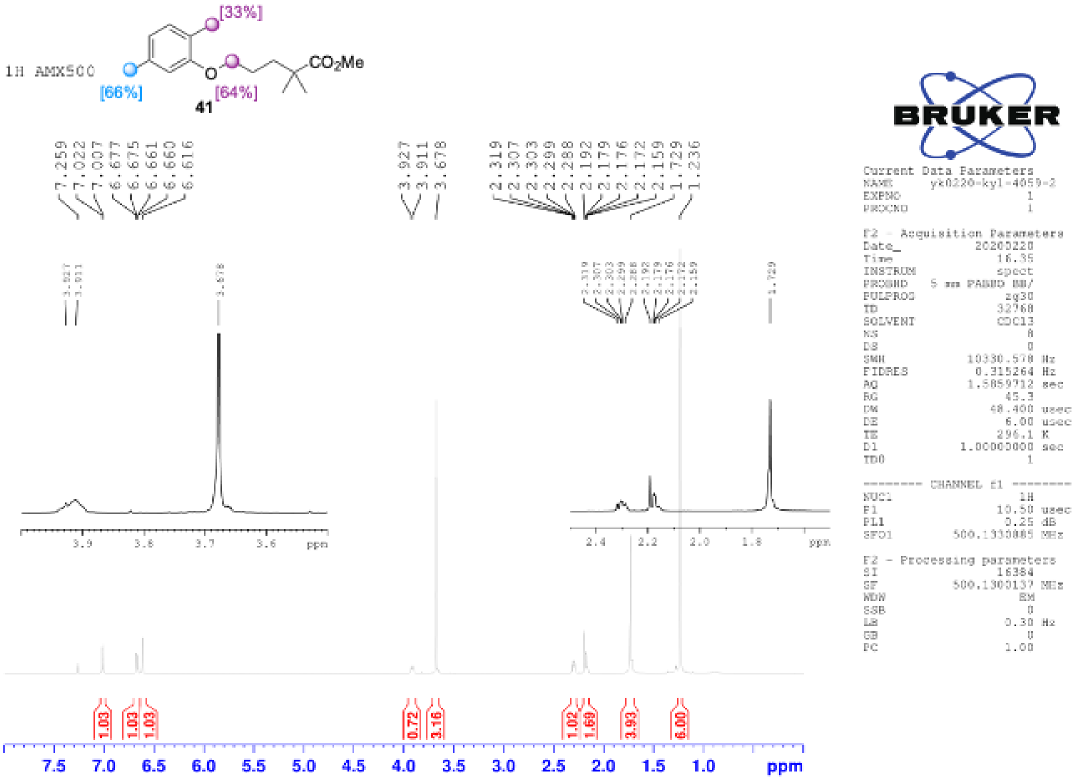
1H AMX500
[33%]
CO2Me
[66%]
[64%]
41
7.259
7.022
7.007
6.677
6.675
6.661
6.660
6.616
3.927
3.911
3.678
2.319
2.307
2.303
2.299
2.288
2.192
2.179
2.176
2.172
2.159
1.729
1.236
BRUKER
Current Data Parameters
F2 - Acquisition Parameters
CHANNEL f1
F2 - Processing parameters
1.03
1.03
1.03
0.72
3.16
1.02
1.69
3.93
6.00
7.5
7.0
6.5
6.0
5.5
5.0
4.5
4.0
3.5
3.0
2.5
2.0
1.5
1.0
ppm

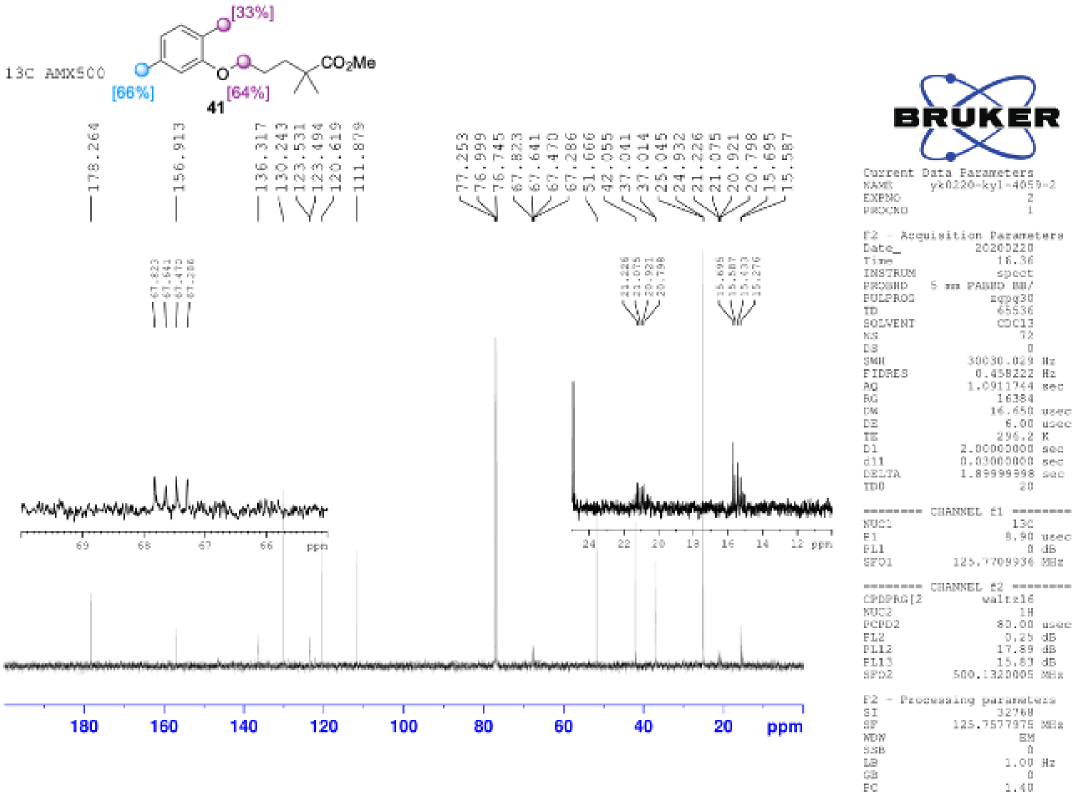
13C AMX500
[33%]
[66%]
[64%]
CO2Me
41
178.264
156.913
136.317
130.243
123.531
123.494
120.619
111.879
77.253
76.999
76.745
67.823
67.641
67.470
67.286
51.666
42.055
37.041
37.014
25.045
24.932
21.226
21.075
20.921
20.798
15.695
15.587
BRUKER
180
160
140
120
100
80
60
40
20
ppm

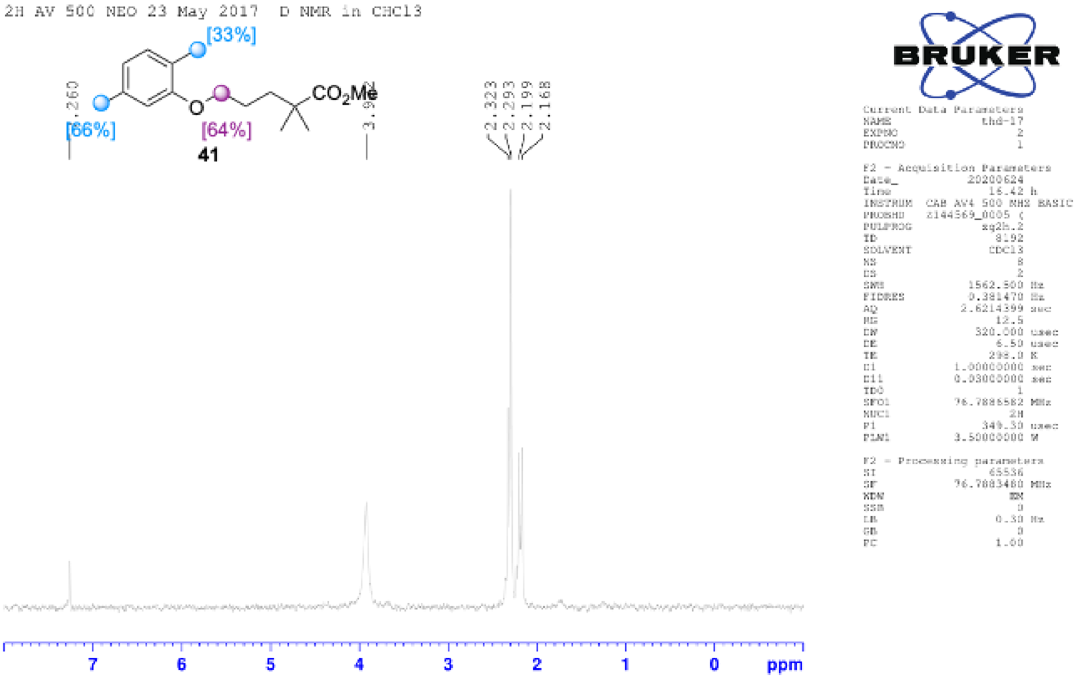
2H AV 500 NEO 23 May 2017 D NMR in CHCl3
[33%]
[66%]
[64%]
CO2Me
41
2.323
2.293
2.199
2.168
BRUKER
7
6
5
4
3
2
1
0
ppm

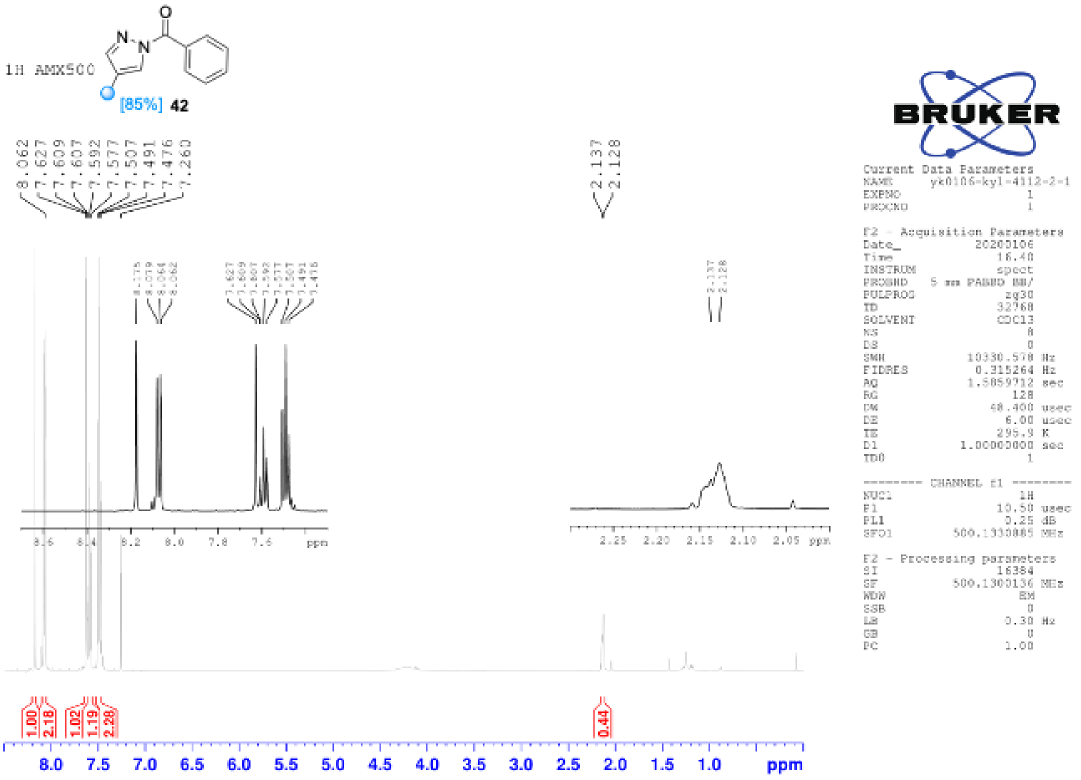
1H AMX500
[85%] 42
8.062 7.627 7.609 7.607 7.592 7.577 7.507 7.491 7.476 7.260
2.137 2.128
1.00 2.18 1.02 1.19 2.28
0.44
8.0 7.5 7.0 6.5 6.0 5.5 5.0 4.5 4.0 3.5 3.0 2.5 2.0 1.5 1.0 ppm
BRUKER

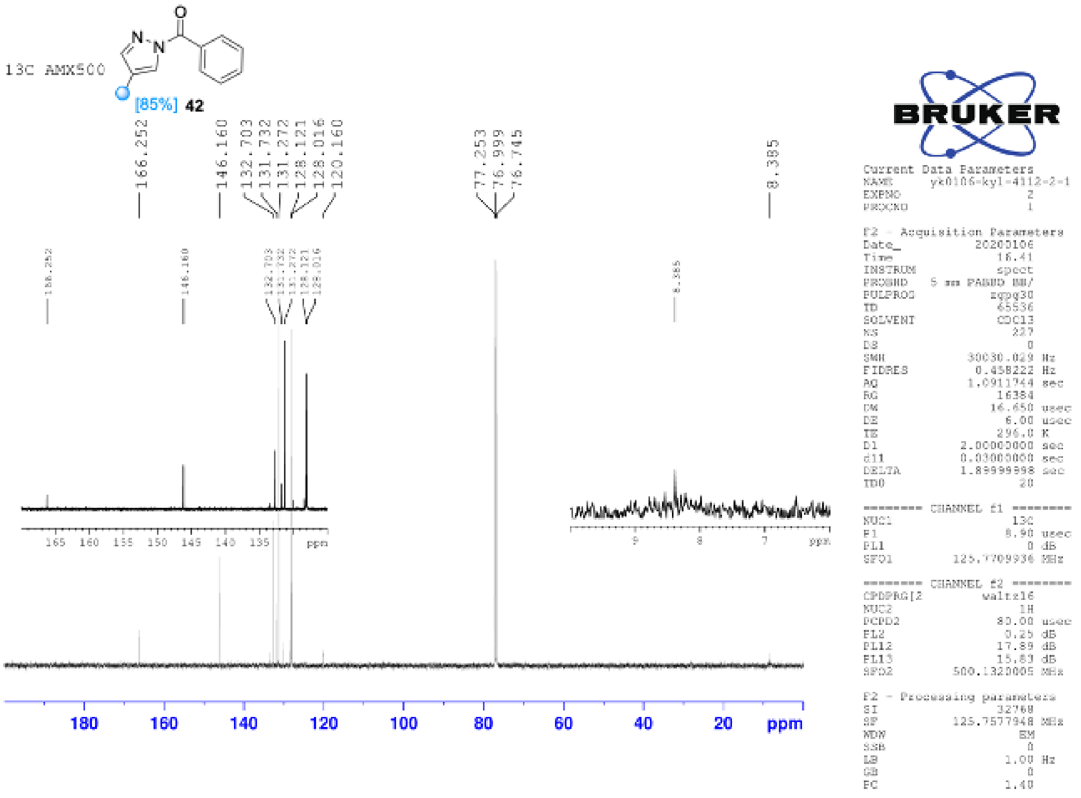
13C AMX500
[85%] 42
166.252
146.160
132.703 131.732 131.272 128.121 128.016 120.160
77.253 76.999 76.745
8.385
180 160 140 120 100 80 60 40 20 ppm
BRUKER

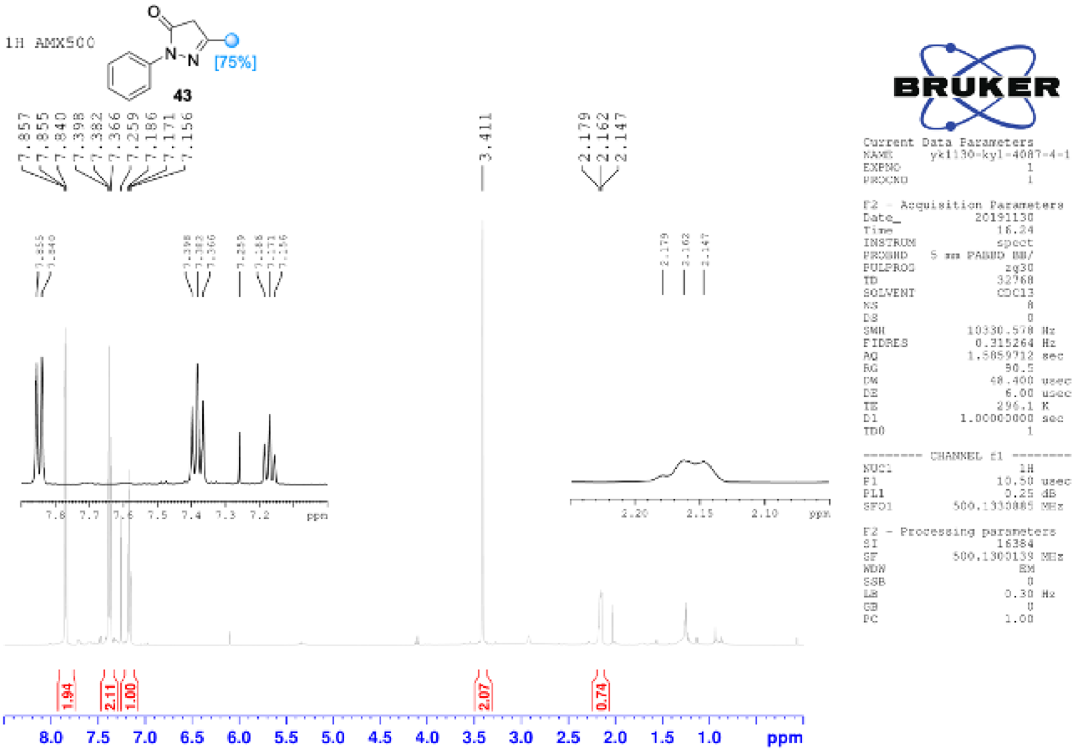
1H AMX500
[75%]
43
7.857 7.855 7.840 7.398 7.382 7.366 7.259 7.186 7.171 7.156
3.411
2.179 2.162 2.147
1.94 2.11 1.00 2.07 0.74
ppm
BRUKER

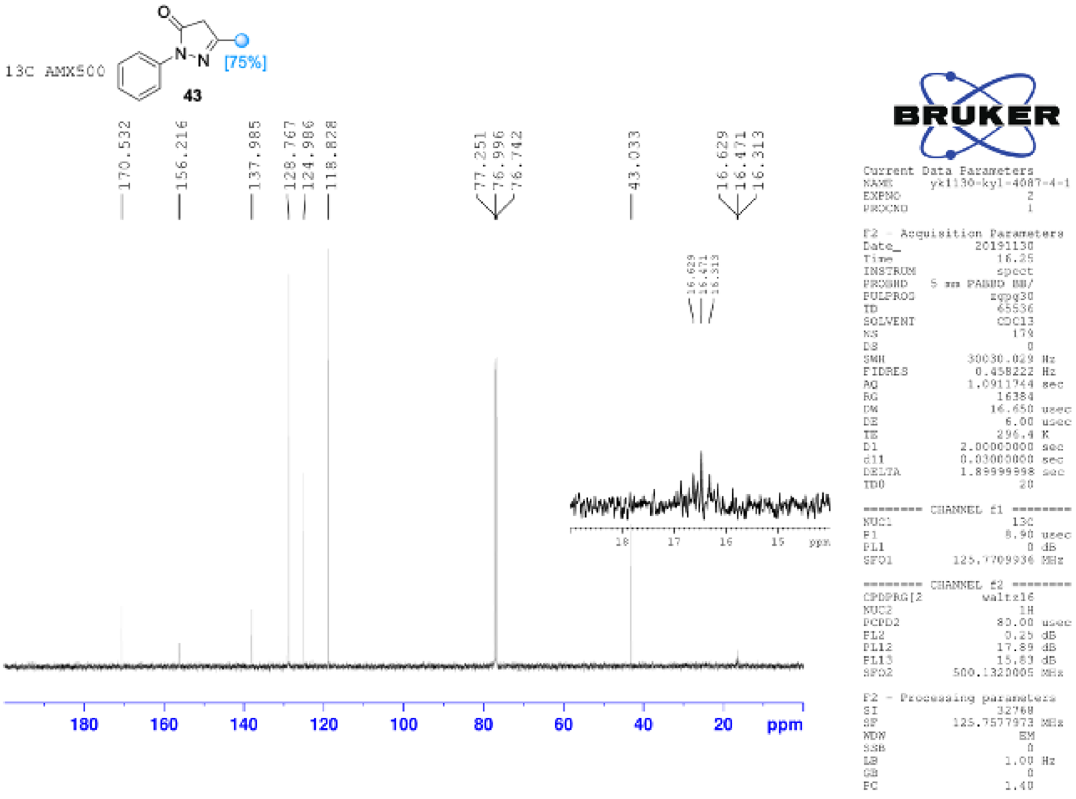
13C AMX500
[75%]
43
170.532
156.216
137.985
128.767
124.986
118.828
77.251 76.996 76.742
43.033
16.629 16.471 16.313
ppm
BRUKER

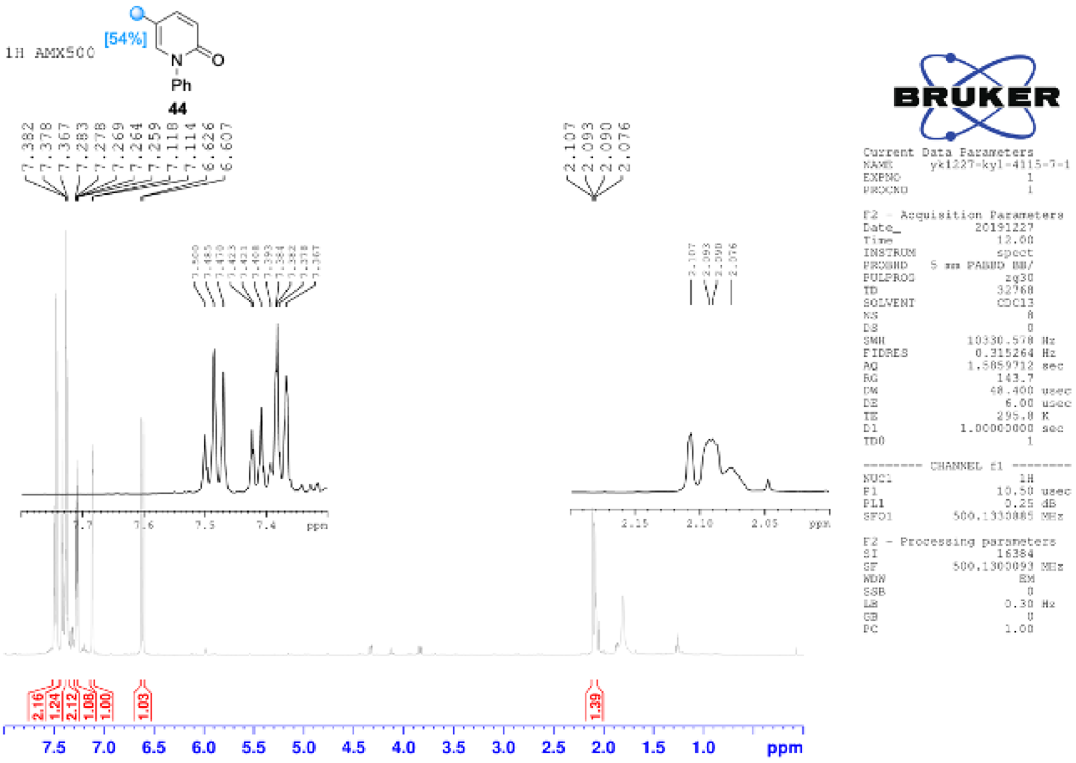
1H AMX500
[54%]
N
O
Ph
44
7.382 7.378 7.367 7.283 7.278 7.269 7.264 7.259 7.118 7.114 6.626 6.607
2.107 2.093 2.090 2.076
2.16 1.24 2.12 1.08 1.00 1.03 1.39
7.5 7.0 6.5 6.0 5.5 5.0 4.5 4.0 3.5 3.0 2.5 2.0 1.5 1.0 ppm
BRUKER

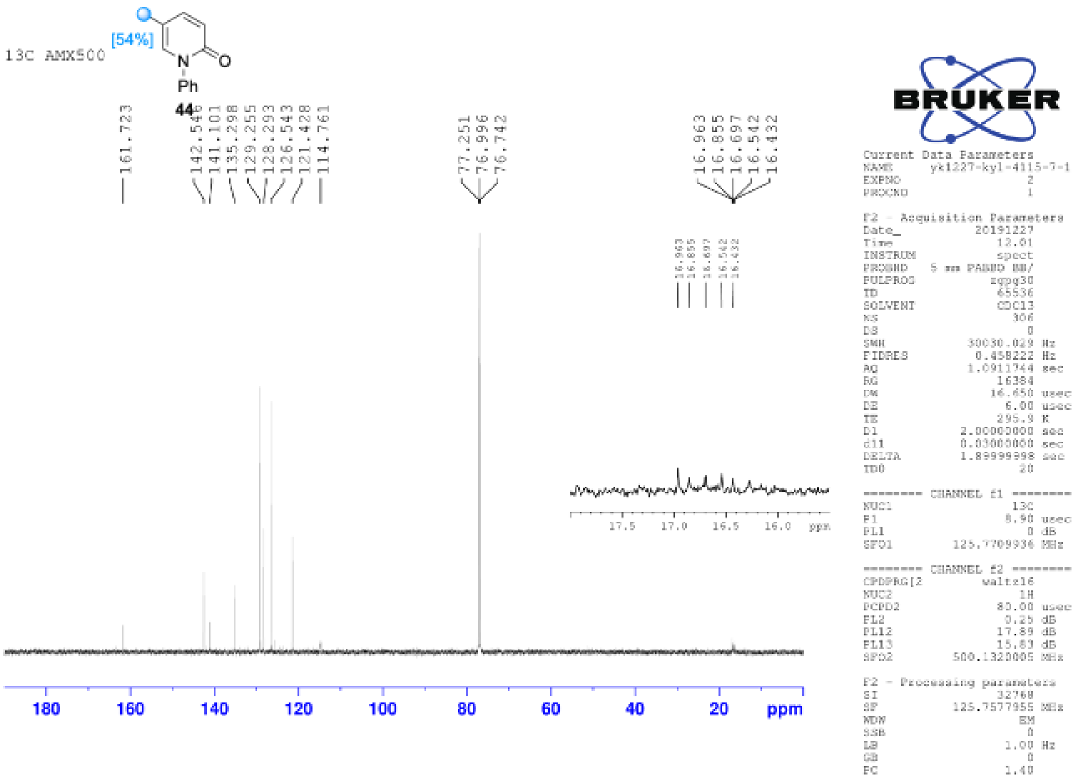
13C AMX500
[54%]
N
O
Ph
44
161.723
142.549 141.101 135.298 129.255 128.293 126.543 121.428 114.761
77.251 76.996 76.742
16.963 16.855 16.697 16.542 16.432
180 160 140 120 100 80 60 40 20 ppm
BRUKER

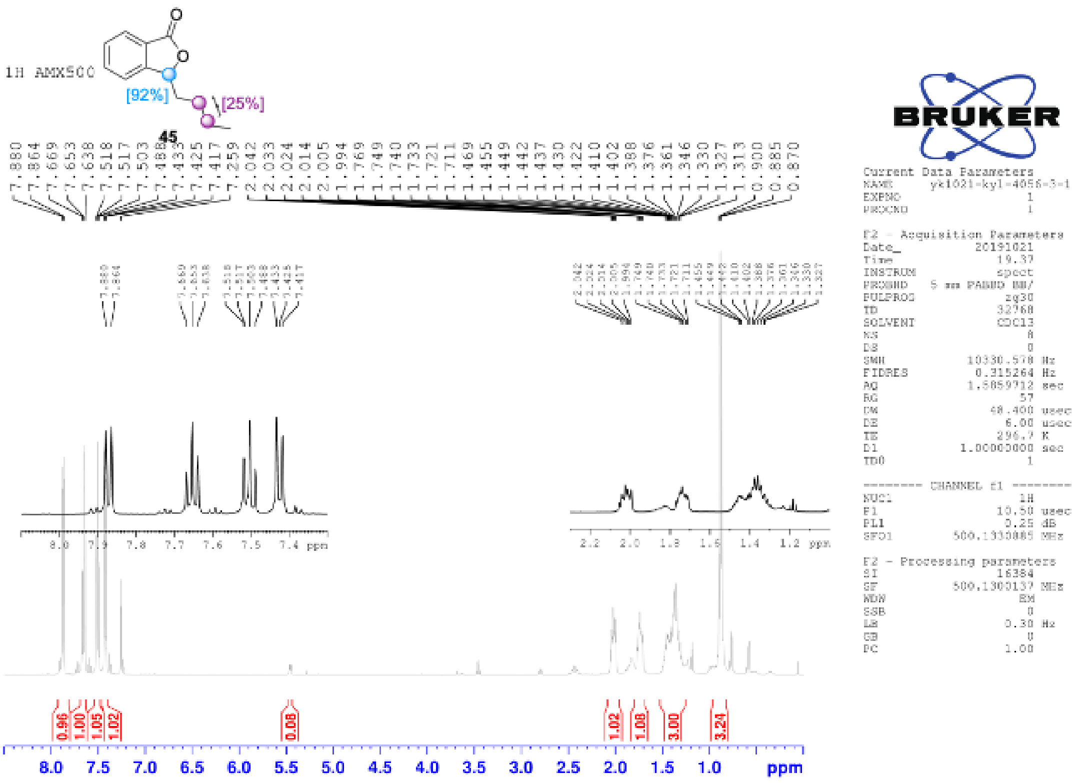

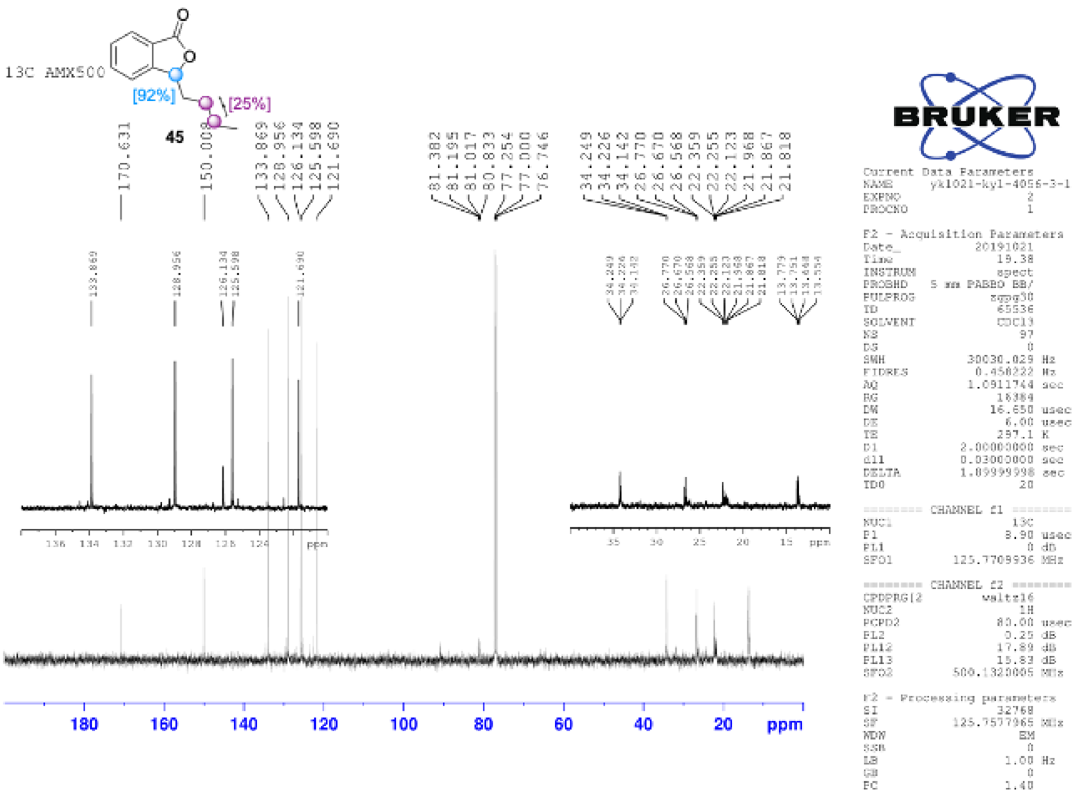

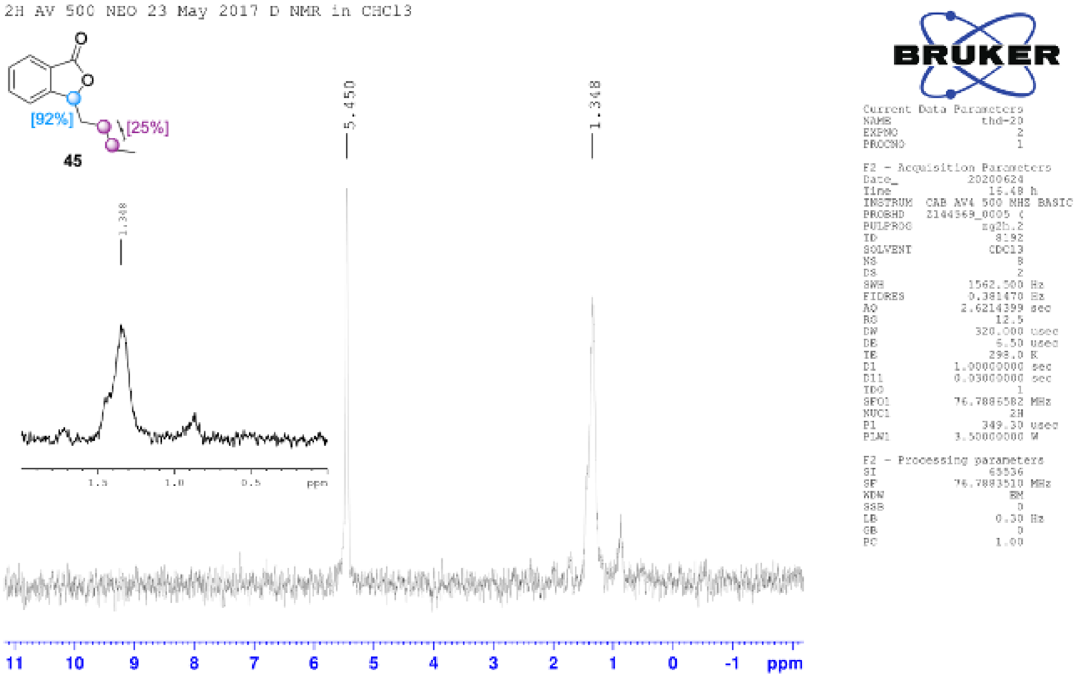
2H AV 500 NEO 23 May 2017 D NMR in CHCl3
[92%]
[25%]
45
5.450
1.348
BRUKER

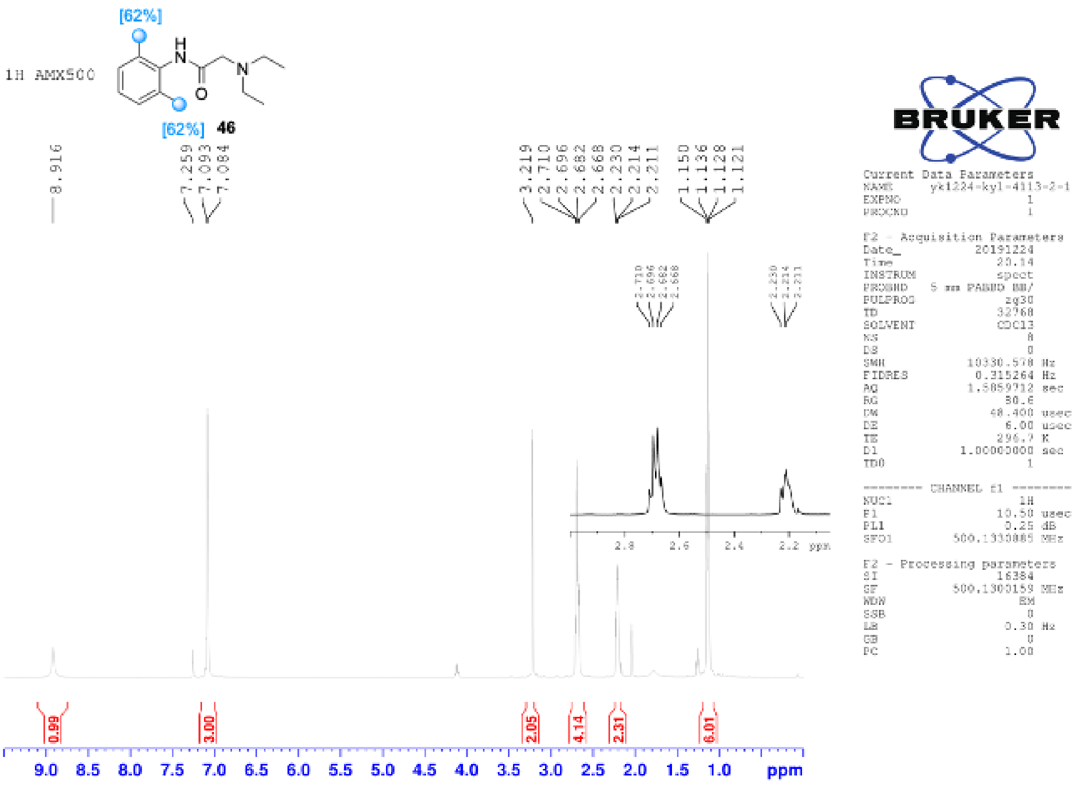
1H AMX500
[62%]
[62%]
46
BRUKER

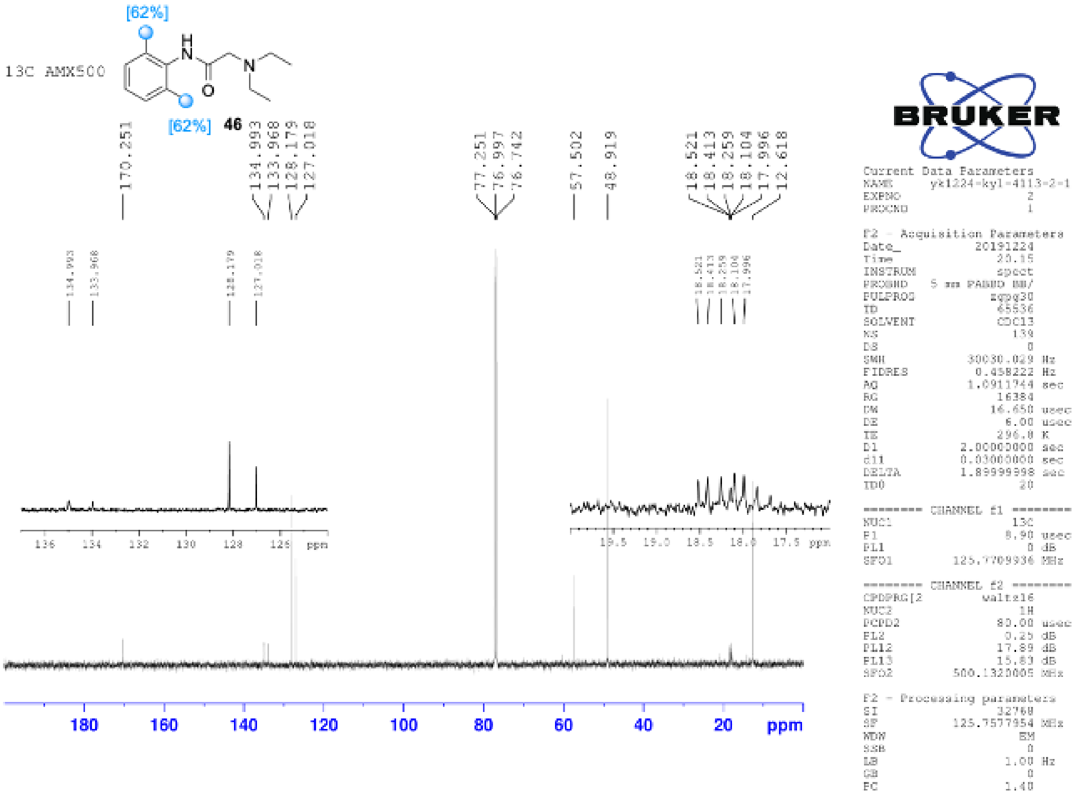
13C AMX500
[62%]
[62%]
46
170.251
134.993
133.968
128.179
127.018
77.251
76.997
76.742
57.502
48.919
18.521
18.413
18.259
18.104
17.996
12.618
BRUKER
180
160
140
120
100
80
60
40
20
ppm

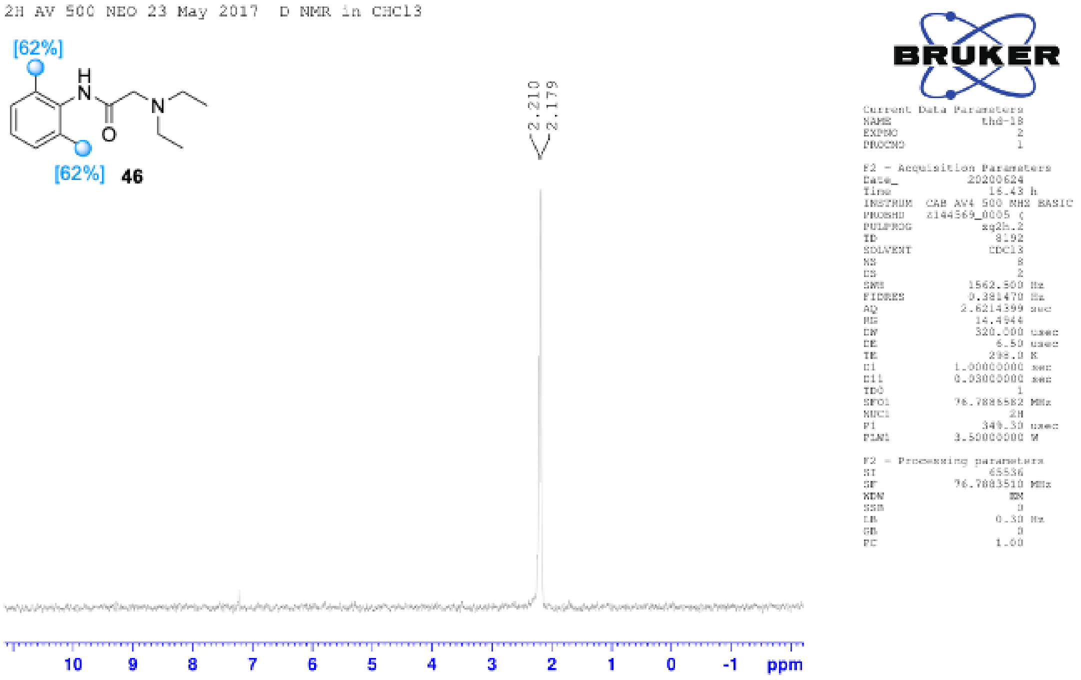
2H AV 500 NEO 23 May 2017 D NMR in CHCl3
[62%]
[62%]
46
2.210
2.179
BRUKER
10
9
8
7
6
5
4
3
2
1
0
-1
ppm

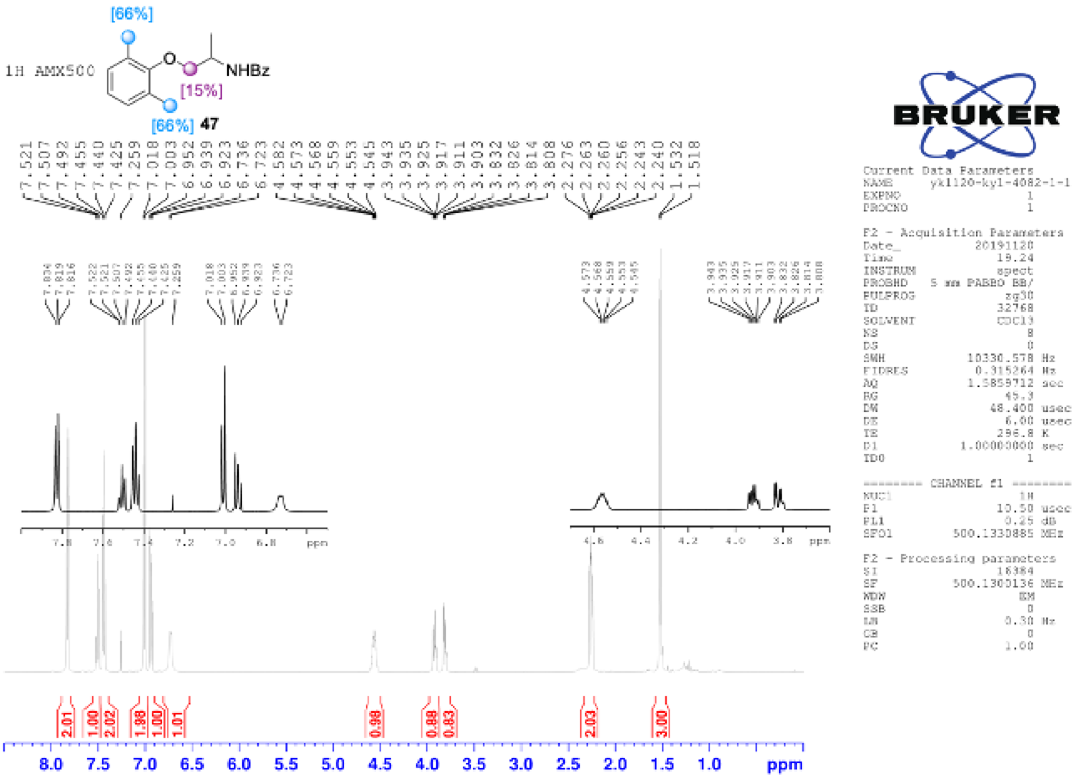
1H AMX500
[66%]
NHBz
[15%]
[66%]
47
BRUKER
ppm

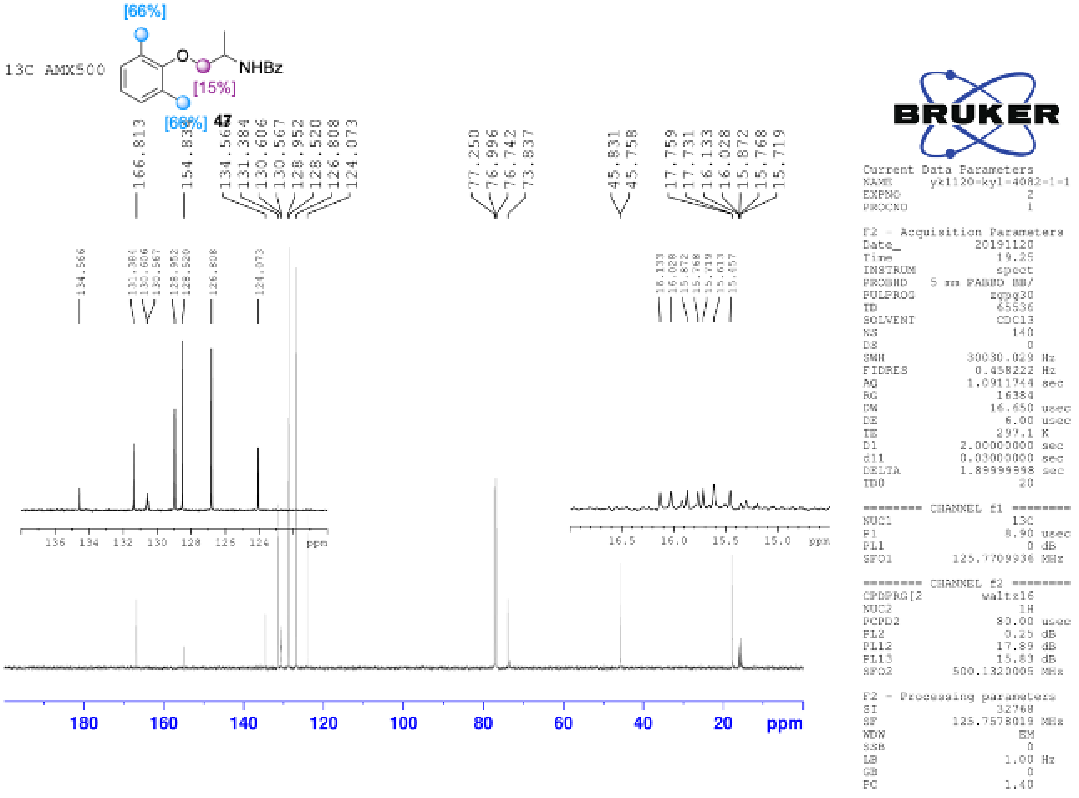
13C AMX500
[66%]
NHBz
[15%]
47
BRUKER
ppm

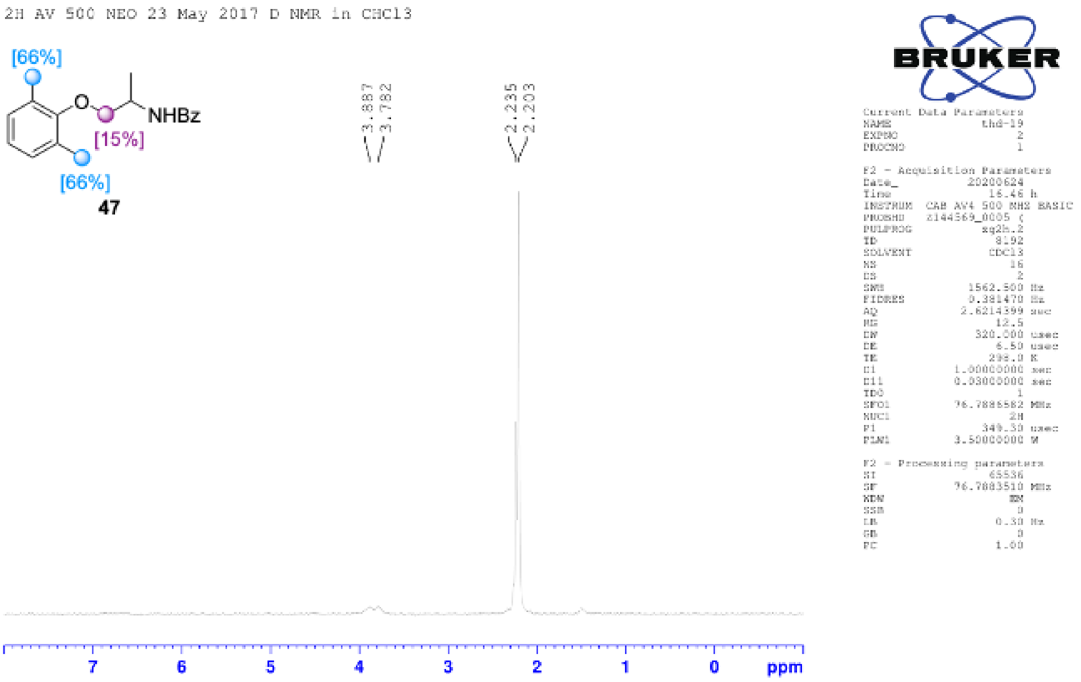
2H AV 500 NEO 23 May 2017 D NMR in CHCl3
[66%]
[15%]
[66%]
47
NHBz
3.887
3.782
2.235
2.203
ppm
BRUKER

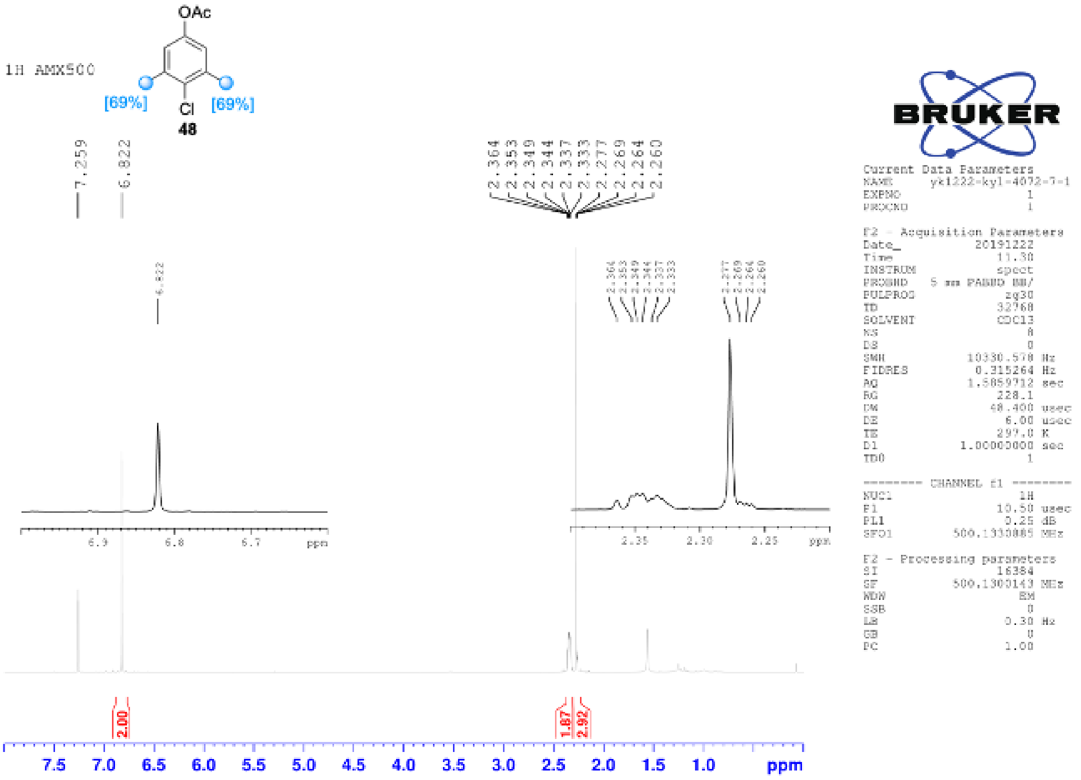
1H AMX500
OAc
Cl
[69%]
[69%]
48
7.259
6.822
2.00
1.87
2.92
ppm
BRUKER

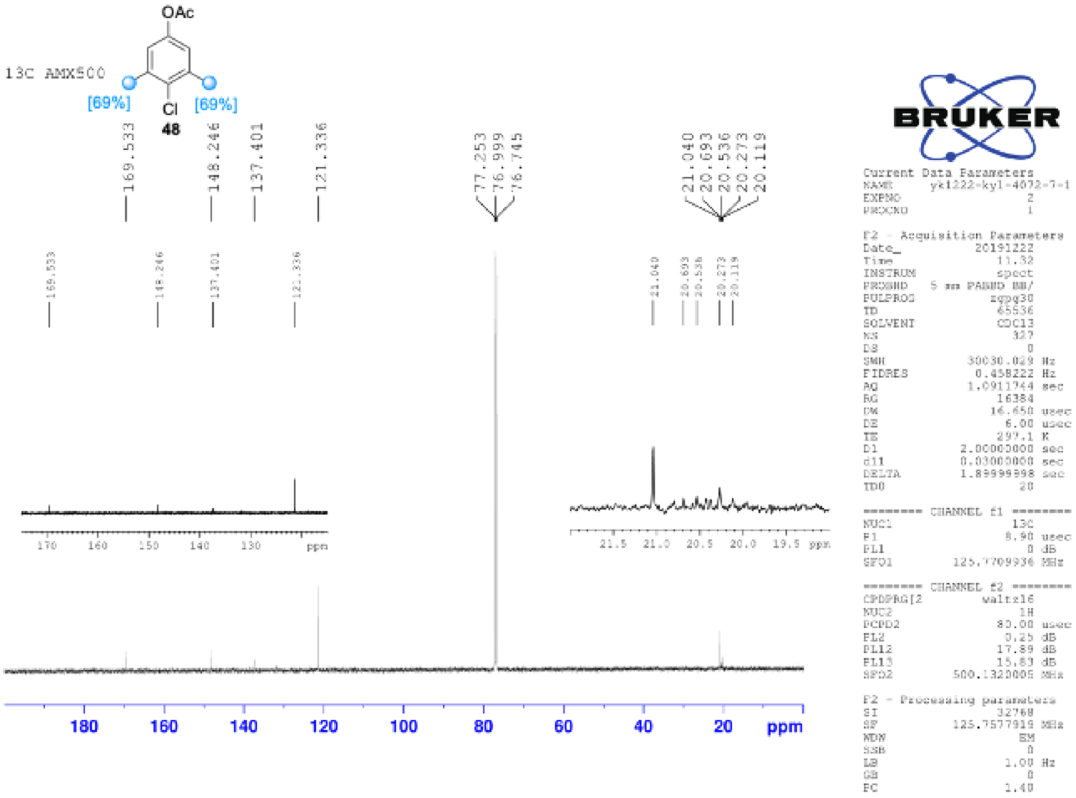
13C AMX500
OAc
Cl
[69%]
[69%]
48
169.533
148.246
137.401
121.336
77.253
76.999
76.745
21.040
20.693
20.536
20.273
20.119
BRUKER
Current Data Parameters
NAME yk1222-kyl-4072-7-1
EXPNO 2
PROCNO 1
F2 - Acquisition Parameters
Date_ 20191222
Time 11.32
INSTRUM spect
PROBHD 5 mm PABBO BB/
PULPROG zgpg30
TD 65536
SOLVENT CDCl3
NS 327
DS 0
SWH 30030.029 Hz
FIDRES 0.458222 Hz
AQ 1.0911744 sec
RG 16384
DW 16.650 usec
DE 6.00 usec
TE 297.1 K
D1 2.00000000 sec
d11 0.03000000 sec
DELTA 1.89999998 sec
TD0 20
======== CHANNEL f1 ========
NUC1 13C
P1 8.90 usec
PL1 0 dB
SFO1 125.7709936 MHz
======== CHANNEL f2 ========
CPDPRG[2 waltz16
NUC2 1H
PCPD2 80.00 usec
PL2 0.25 dB
PL12 17.89 dB
PL13 15.83 dB
SFO2 500.1320005 MHz
F2 - Processing parameters
SI 32768
SF 125.7577919 MHz
WDW EM
SSB 0
LB 1.00 Hz
GB 0
PC 1.40
180
160
140
120
100
80
60
40
20
ppm

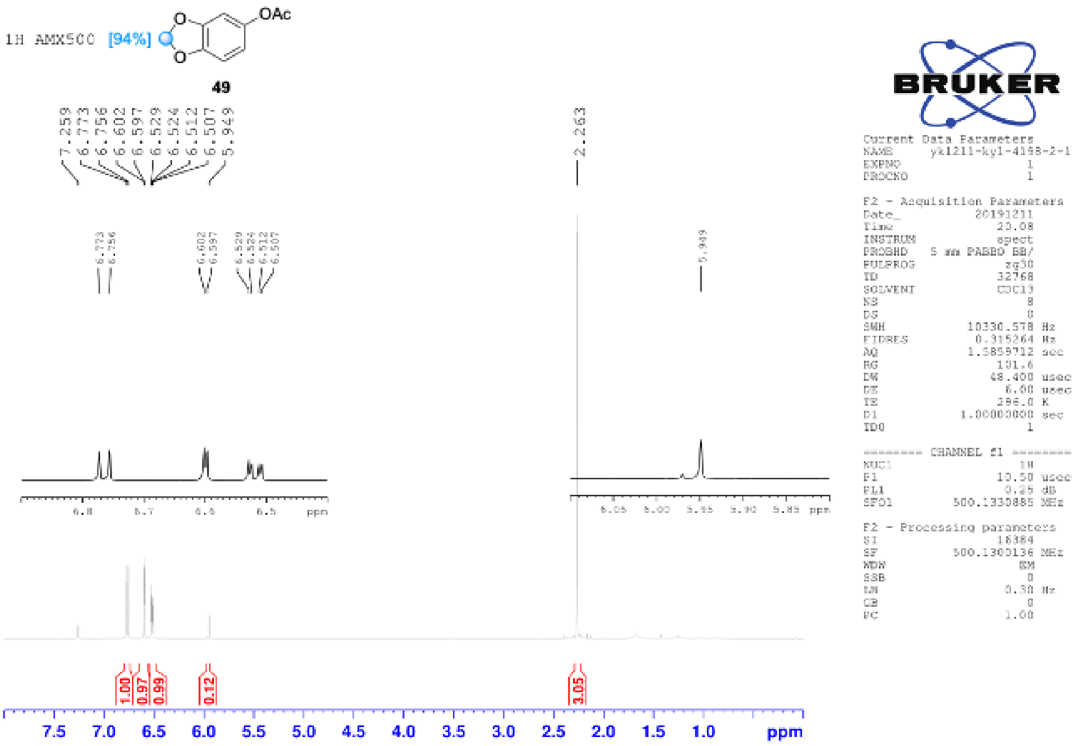
1H AMX500
[94%]
O
O
OAc
49
7.259
6.773
6.756
6.602
6.597
6.529
6.524
6.512
6.507
5.949
2.263
BRUKER
Current Data Parameters
NAME yk1211-kyl-4198-2-1
EXPNO 1
PROCNO 1
F2 - Acquisition Parameters
Date_ 20191211
Time 20.08
INSTRUM spect
PROBHD 5 mm PABBO BB/
PULPROG zg30
TD 32768
SOLVENT CDCl3
NS 8
DS 0
SWH 10330.578 Hz
FIDRES 0.315264 Hz
AQ 1.5859712 sec
RG 101.6
DW 48.400 usec
DE 6.00 usec
TE 296.0 K
D1 1.00000000 sec
TD0 1
======== CHANNEL f1 ========
NUC1 1H
P1 10.50 usec
PL1 0.25 dB
SFO1 500.1330885 MHz
F2 - Processing parameters
SI 16384
SF 500.1300136 MHz
WDW EM
SSB 0
LB 0.30 Hz
GB 0
PC 1.00
1.00
0.97
0.99
0.12
3.05
7.5
7.0
6.5
6.0
5.5
5.0
4.5
4.0
3.5
3.0
2.5
2.0
1.5
1.0
ppm

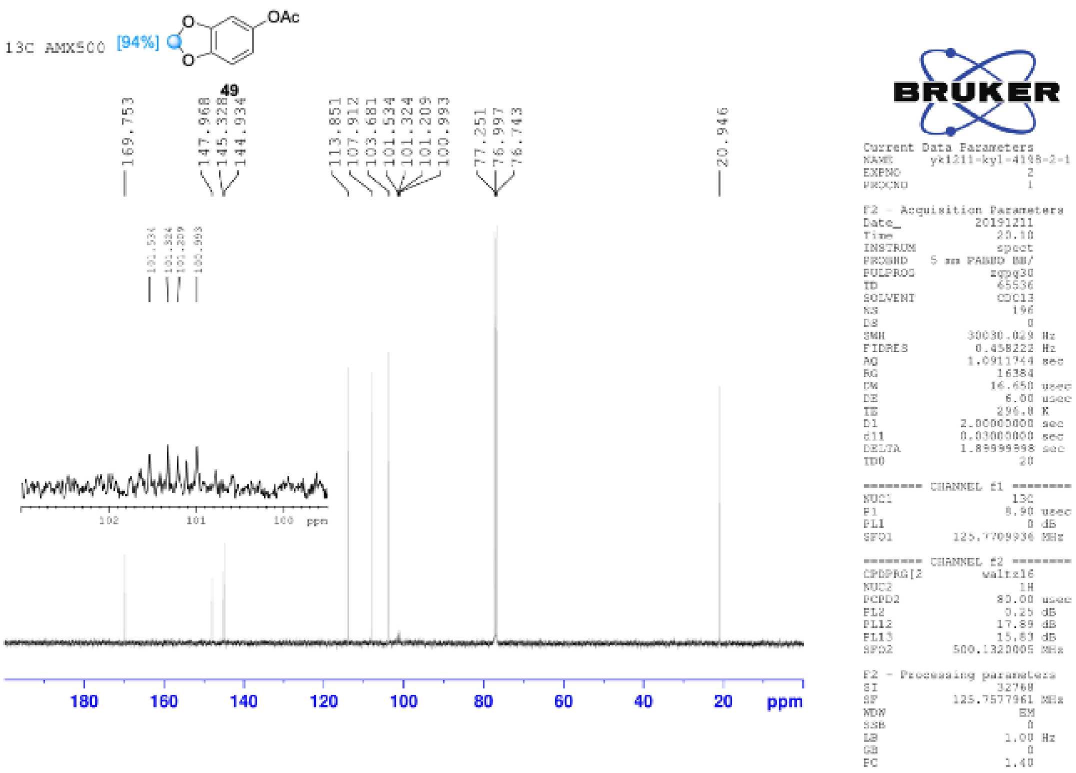
13C AMX500
[94%]
OAc
49
169.753
147.968
145.328
144.934
113.851
107.912
103.681
101.534
101.324
101.209
100.993
77.251
76.997
76.743
20.946
180 160 140 120 100 80 60 40 20 ppm
BRUKER

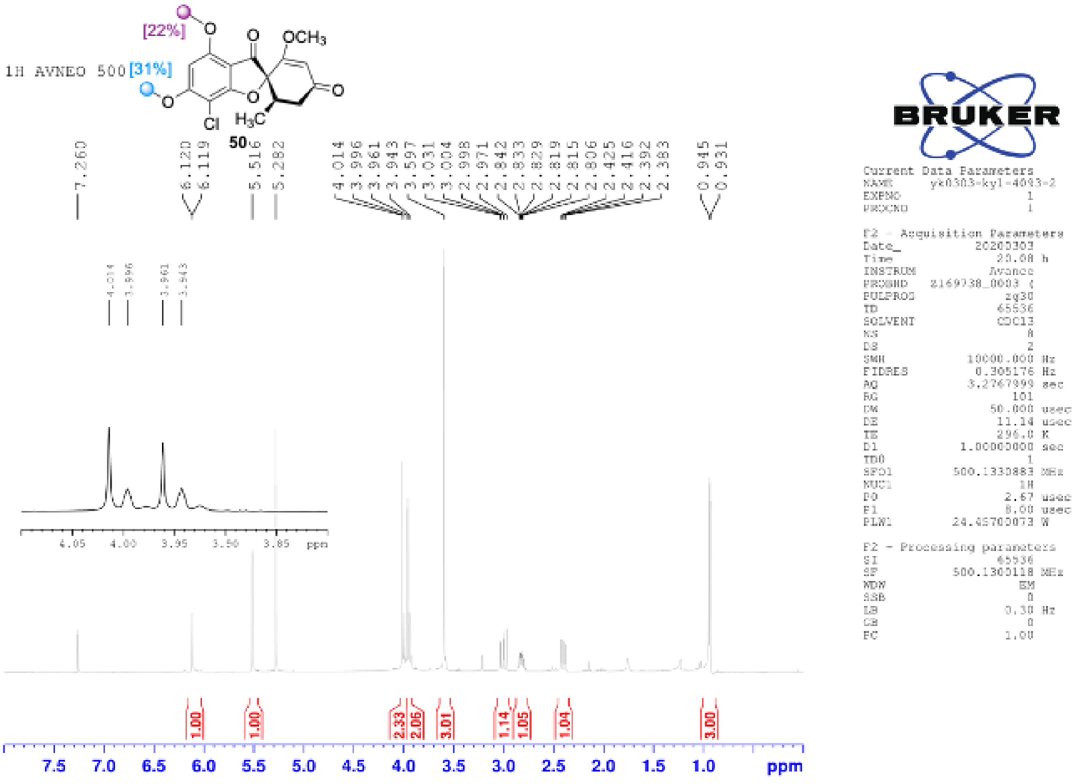
1H AVNEO 500
[22%]
[31%]
OCH3
Cl
H3C
50
7.260
6.120
6.119
5.516
5.282
4.014
3.996
3.961
3.943
3.597
3.031
3.004
2.998
2.971
2.842
2.833
2.829
2.819
2.815
2.806
2.425
2.416
2.392
2.383
0.945
0.931
1.00
1.00
2.33
2.06
3.01
1.14
1.05
1.04
3.00
7.5 7.0 6.5 6.0 5.5 5.0 4.5 4.0 3.5 3.0 2.5 2.0 1.5 1.0 ppm
BRUKER

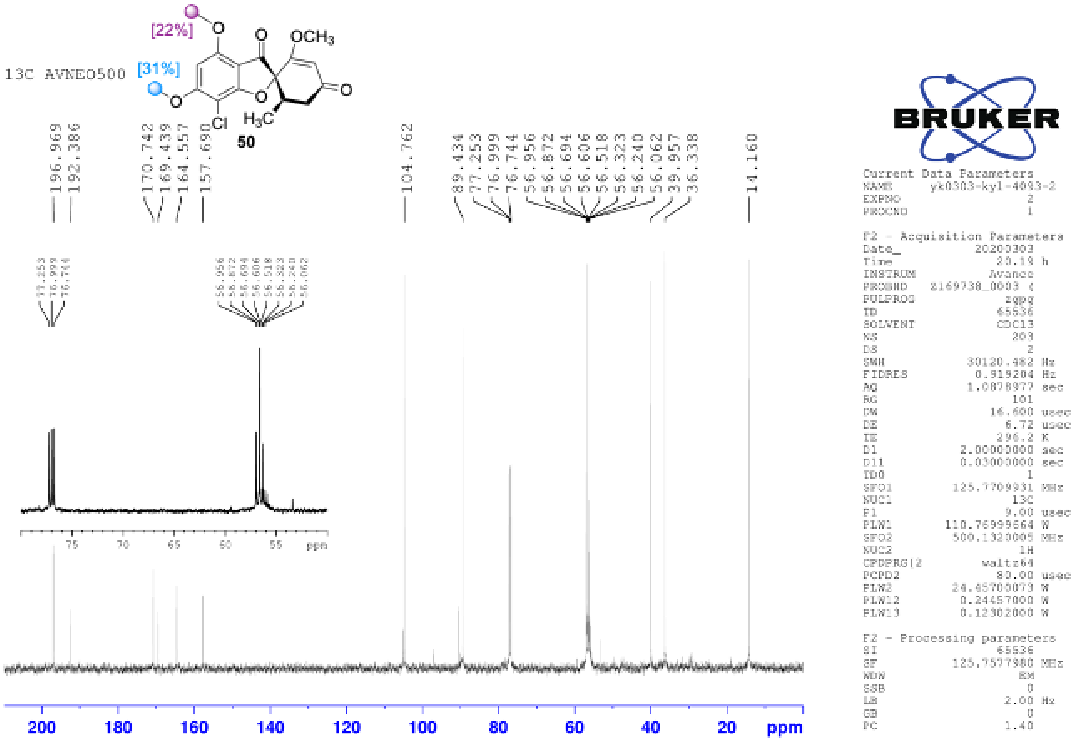
13C AVNEO500
[22%]
[31%]
OCH3
Cl
H3C
50
196.969
192.386
170.742
169.439
164.557
157.690
104.762
89.434
77.253
76.999
76.744
56.956
56.872
56.694
56.606
56.518
56.323
56.240
56.062
39.957
36.338
14.160
BRUKER
200
180
160
140
120
100
80
60
40
20
ppm

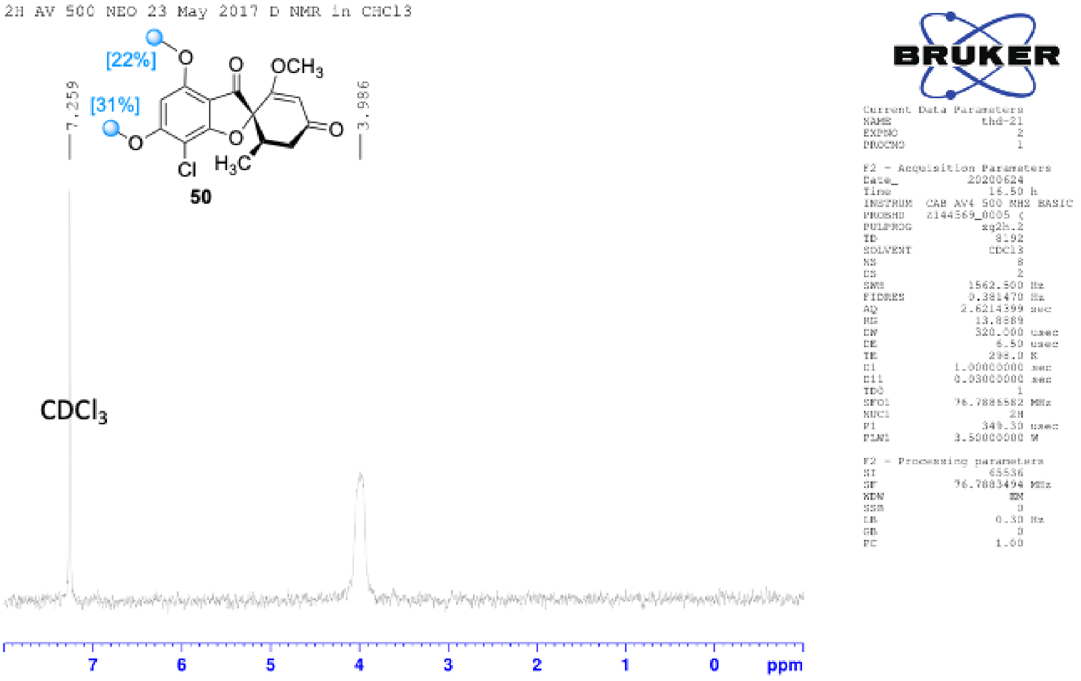
2H AV 500 NEO 23 May 2017 D NMR in CHCl3
[22%]
[31%]
OCH3
Cl
H3C
50
7.259
3.986
CDCl3
BRUKER
7
6
5
4
3
2
1
0
ppm

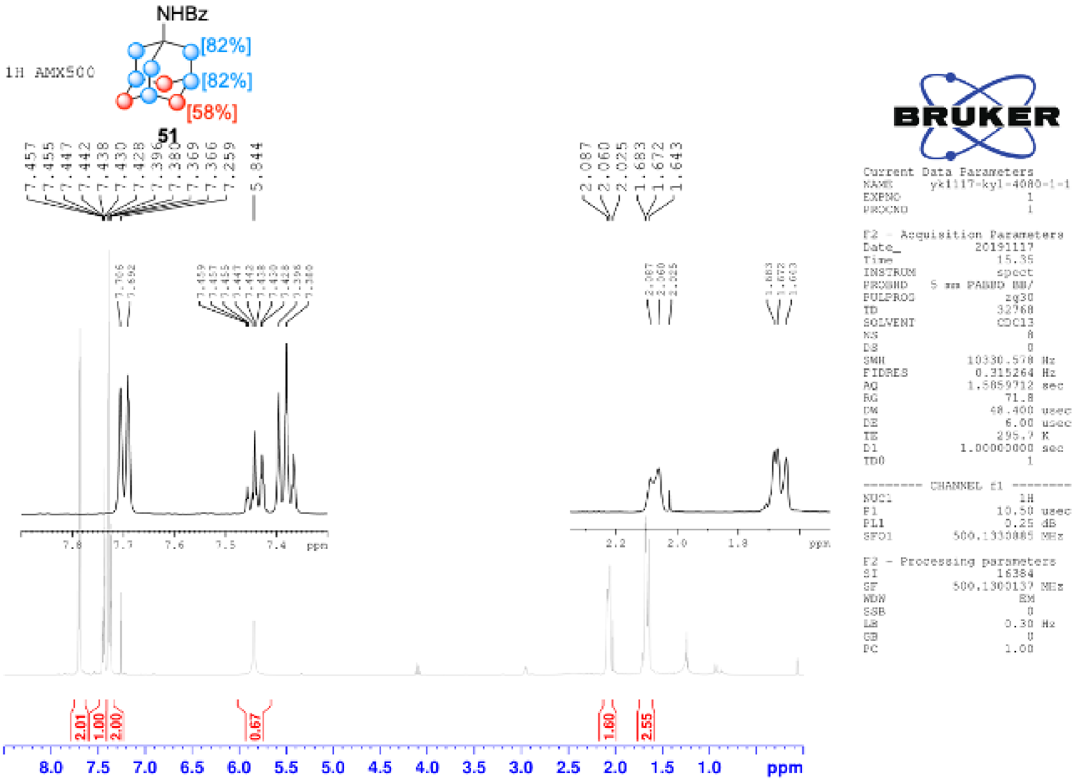
1H AMX500
NHBz
[82%]
[82%]
[58%]
51
BRUKER
ppm

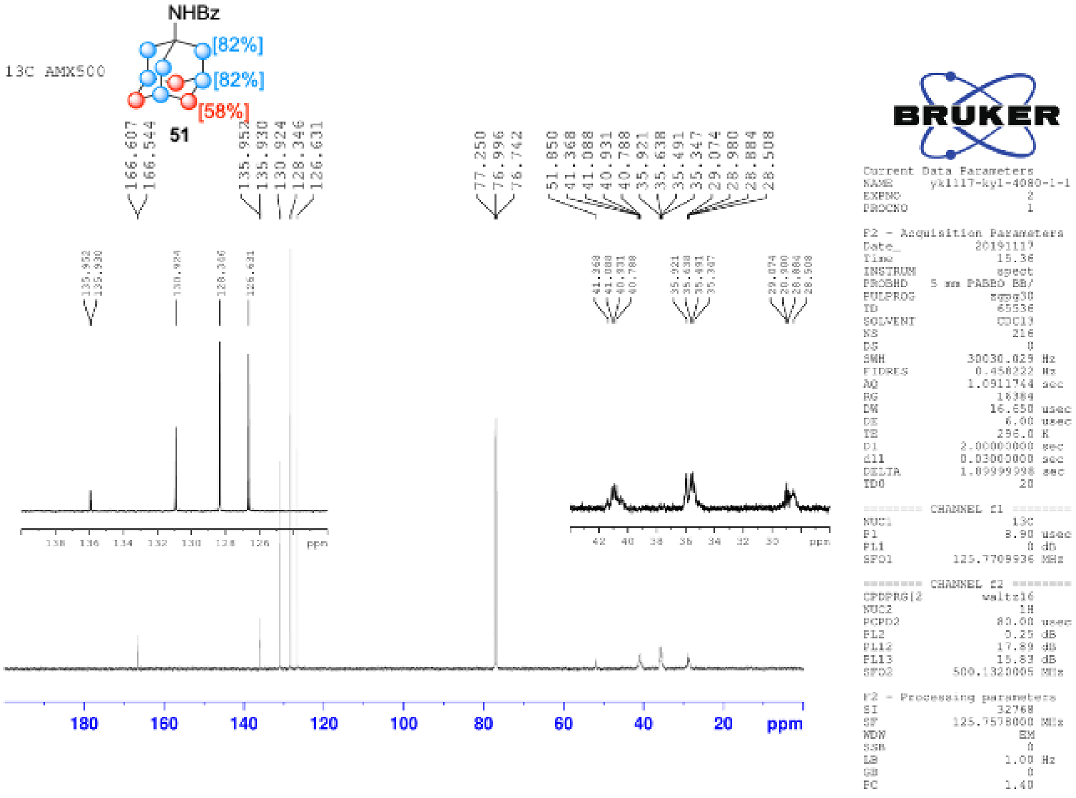
13C AMX500
NHBz
[82%]
[82%]
[58%]
51
BRUKER
ppm

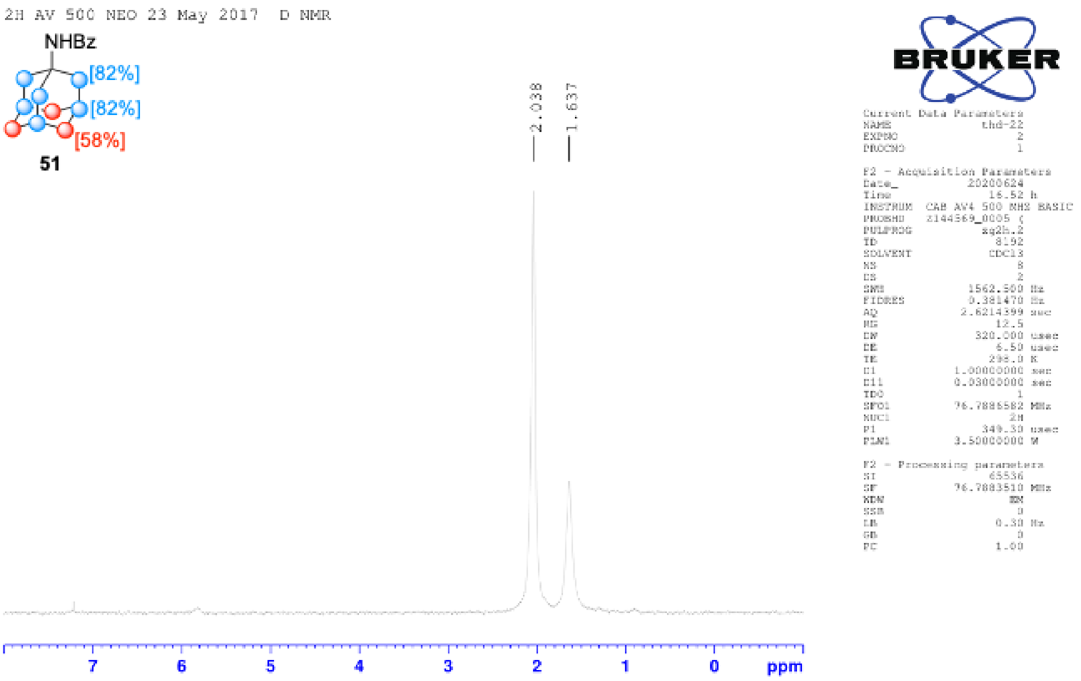
2H AV 500 NEO 23 May 2017 D NMR
NHBz
[82%]
[82%]
[58%]
51
2.038
1.637
BRUKER
ppm

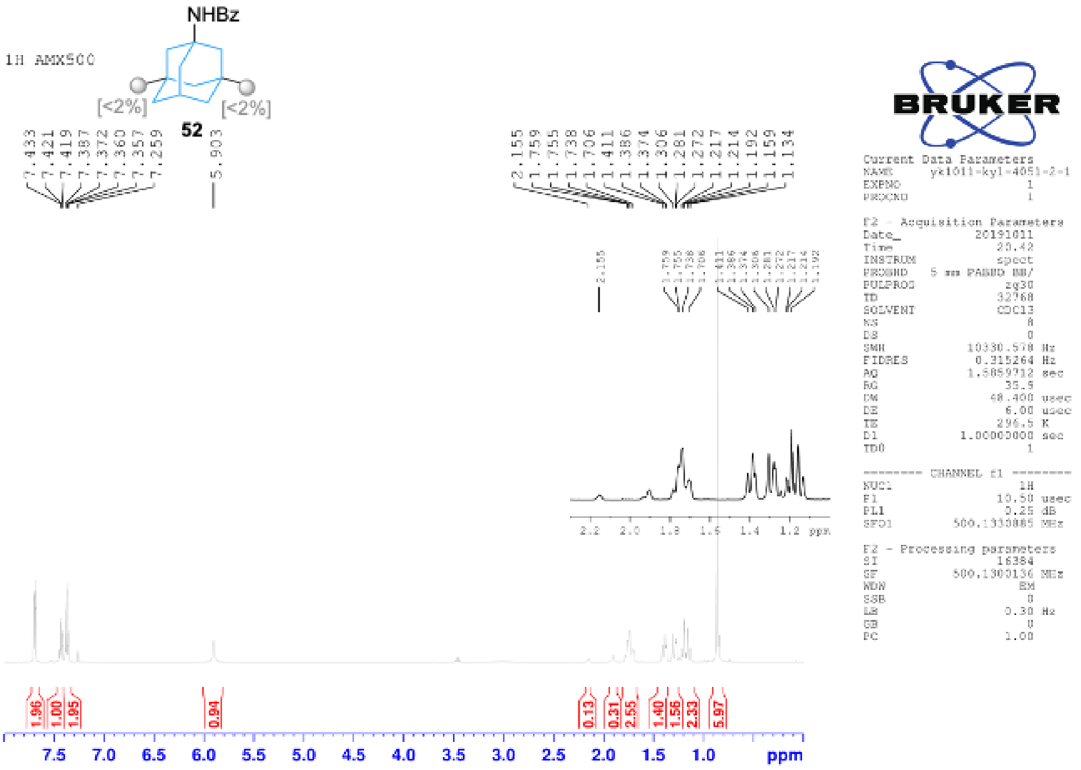
1H AMX500
NHBz
[<2%]
[<2%]
52
5.903
BRUKER
ppm

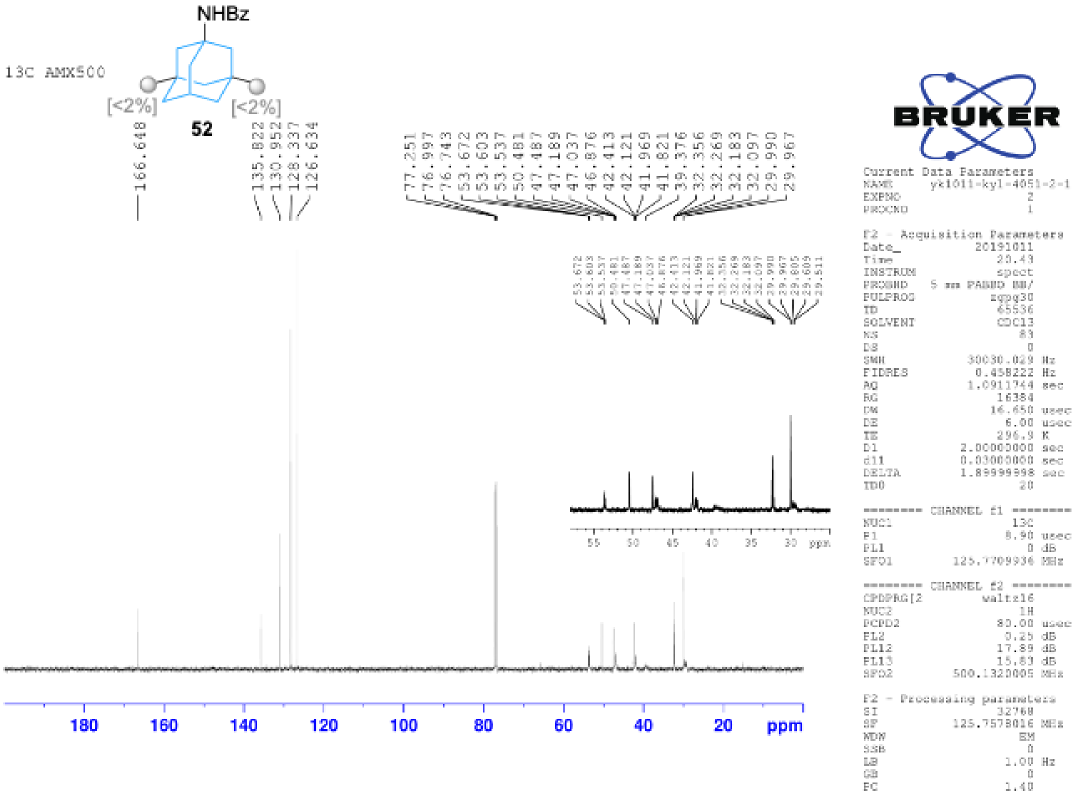
13C AMX500
NHBz
[<2%]
[<2%]
52
166.648
135.822
130.952
128.337
126.634
77.251
76.997
76.743
53.672
53.603
53.537
50.481
47.487
47.189
47.037
46.876
42.413
42.121
41.969
41.821
39.376
32.356
32.269
32.183
32.097
29.990
29.967
BRUKER
ppm

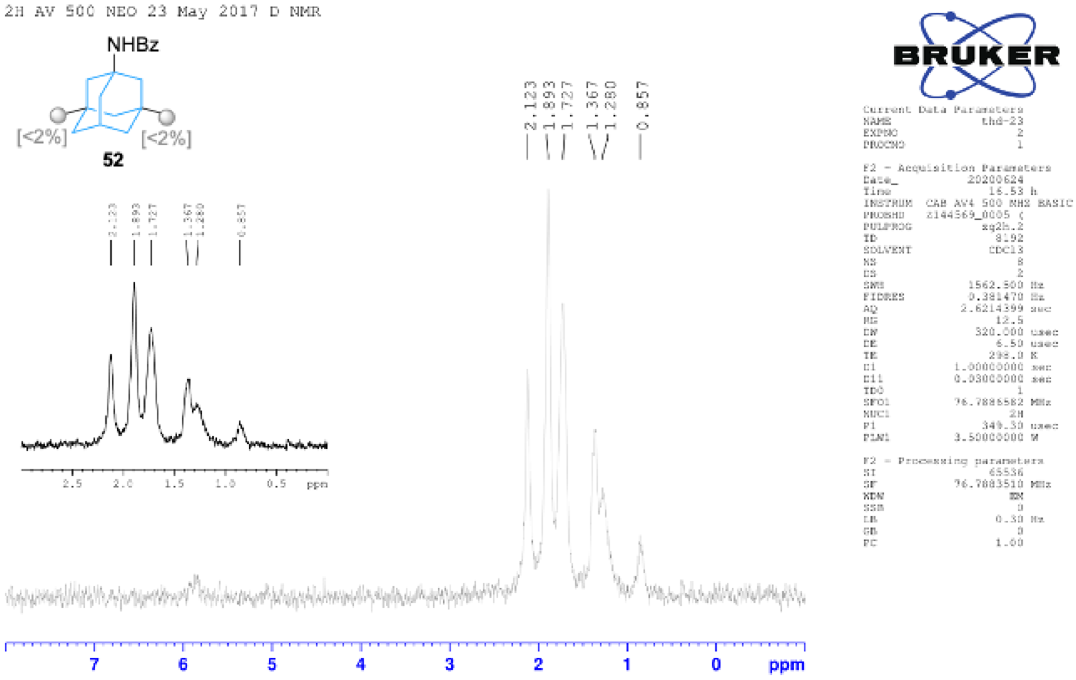
2H AV 500 NEO 23 May 2017 D NMR
NHBz
[<2%]
[<2%]
52
2.123
1.893
1.727
1.367
1.280
0.857
BRUKER
ppm

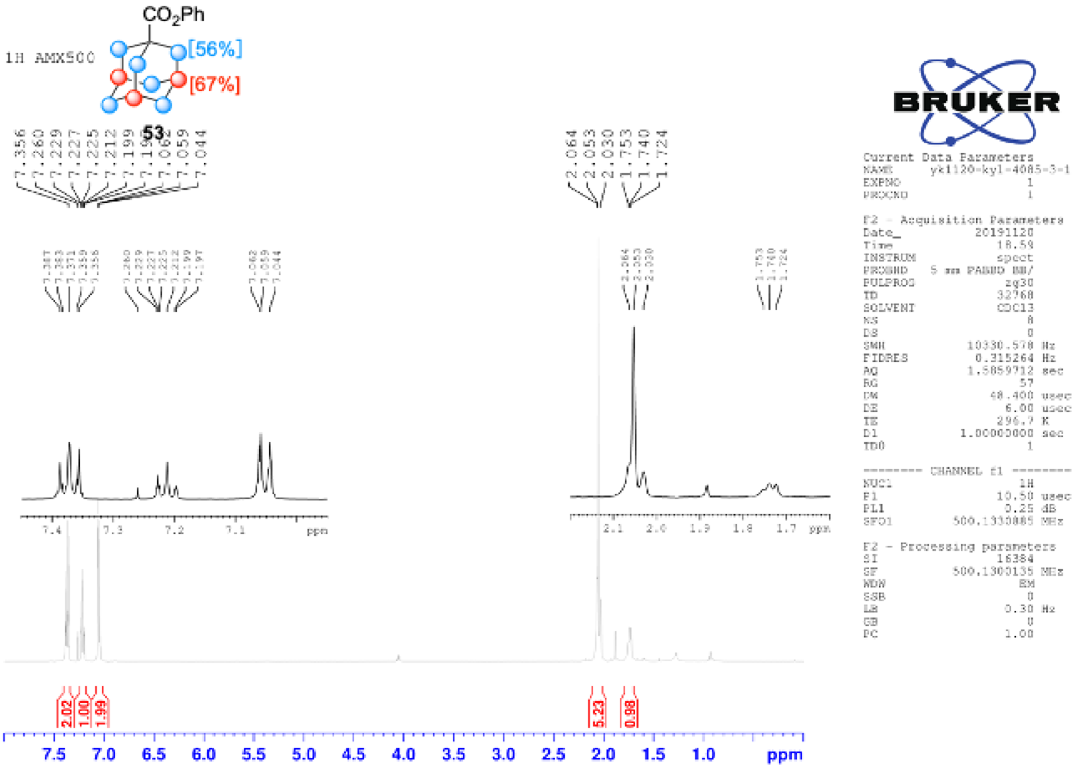
1H AMX500
CO2Ph
[56%]
[67%]
53
7.356
7.260
7.229
7.227
7.225
7.212
7.199
7.062
7.059
7.044
2.064
2.053
2.030
1.753
1.740
1.724
2.02
1.00
1.99
5.23
0.98
ppm
BRUKER
Current Data Parameters
NAME yk1120-kyl-4085-3-1
EXPNO 1
PROCNO 1
F2 - Acquisition Parameters
Date_ 20191120
INSTRUM spect
PROBHD 5 mm PABBO BB/
PULPROG zg30
TD 32768
SOLVENT CDCl3
NS 8
DS 0
SWH 10330.578 Hz
FIDRES 0.315264 Hz
AQ 1.5859712 sec
RG 57
DW 48.400 usec
DE 6.00 usec
TE 296.7 K
D1 1.00000000 sec
TD0 1
======== CHANNEL f1 ========
NUC1 1H
P1 10.50 usec
PL1 0.25 dB
SFO1 500.1330885 MHz
F2 - Processing parameters
SI 16384
SF 500.1300135 MHz
WDW EM
SSB 0
LB 0.30 Hz
GB 0
PC 1.00

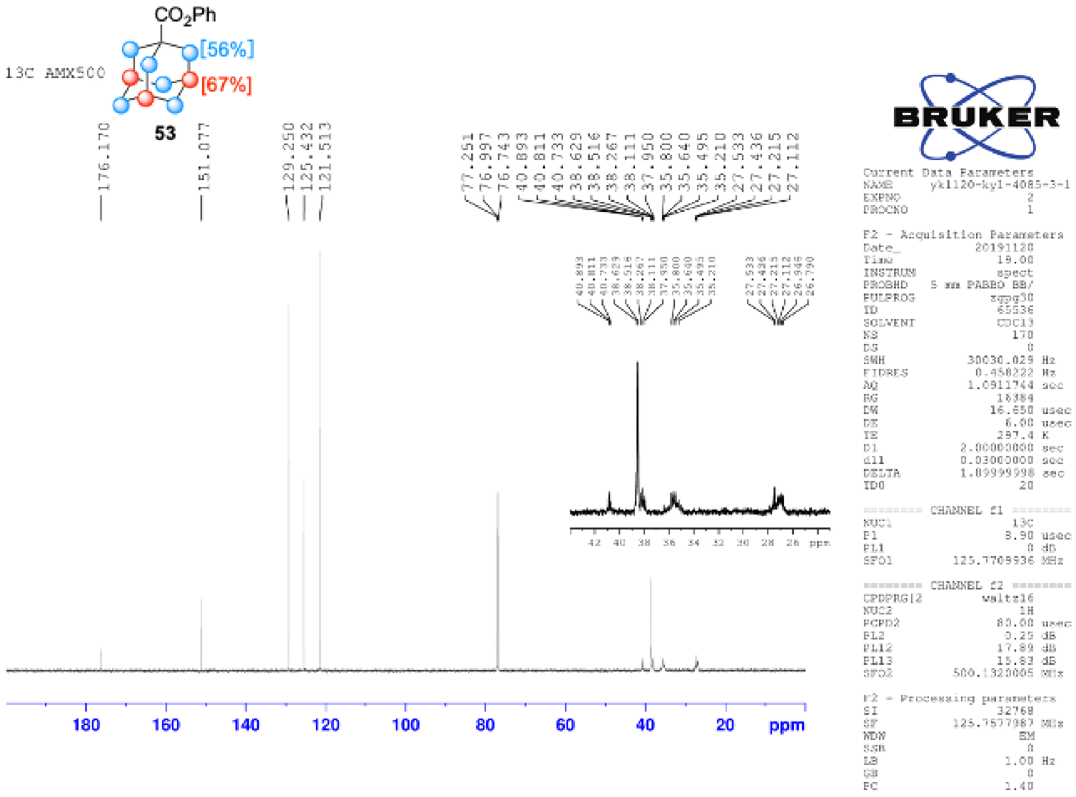
13C AMX500
CO2Ph
[56%]
[67%]
53
176.170
151.077
129.250
125.432
121.513
77.251
76.997
76.743
40.893
40.811
40.733
38.629
38.516
38.267
38.111
37.950
35.800
35.640
35.495
35.210
27.533
27.436
27.215
27.112
ppm
BRUKER
Current Data Parameters
NAME yk1120-kyl-4085-3-1
EXPNO 2
PROCNO 1
F2 - Acquisition Parameters
Date_ 20191120
Time 19.00
INSTRUM spect
PROBHD 5 mm PABBO BB/
PULPROG zgpg30
TD 65536
SOLVENT CDCl3
NS 170
DS 0
SWH 30030.029 Hz
FIDRES 0.458222 Hz
AQ 1.0911744 sec
RG 16384
DW 16.650 usec
DE 6.00 usec
TE 297.4 K
D1 2.00000000 sec
d11 0.03000000 sec
DELTA 1.09999998 sec
TD0 20
======== CHANNEL f1 ========
NUC1 13C
P1 8.90 usec
PL1 0 dB
SFO1 125.7709936 MHz
======== CHANNEL f2 ========
CPDPRG[2 waltz16
NUC2 1H
PCPD2 80.00 usec
PL2 0.25 dB
PL12 17.89 dB
PL13 15.83 dB
SFO2 500.1320005 MHz
F2 - Processing parameters
SI 32768
SF 125.7577987 MHz
WDW EM
SSB 0
LB 1.00 Hz
GB 0
PC 1.40

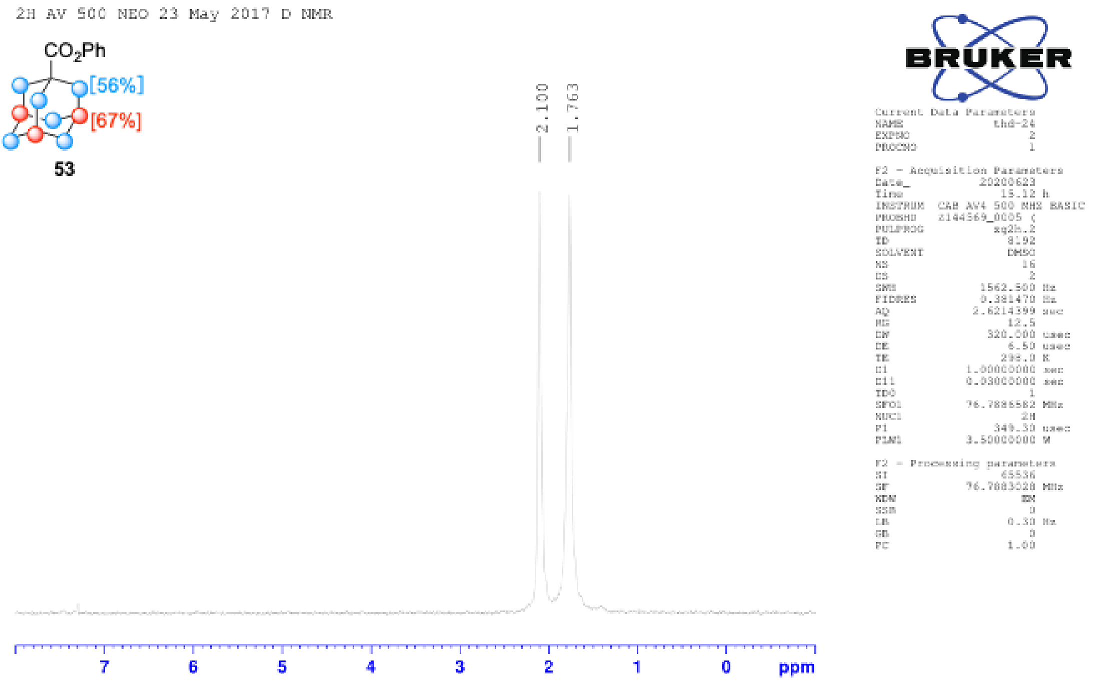
2H AV 500 NEO 23 May 2017 D NMR
CO2Ph
[56%]
[67%]
53
2.100
1.763
BRUKER
7
6
5
4
3
2
1
0
ppm

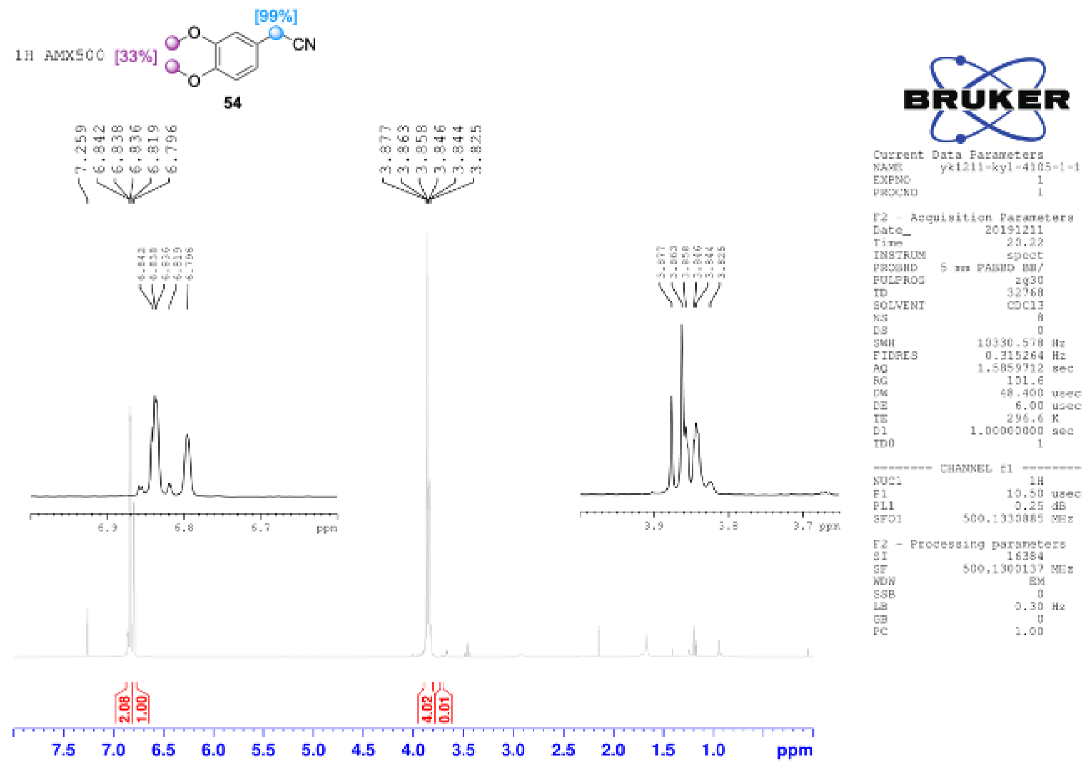
1H AMX500
[33%]
[99%]
CN
54
7.259
6.842
6.838
6.836
6.819
6.796
3.877
3.863
3.858
3.846
3.844
3.825
BRUKER
2.08
1.00
4.02
0.01
7.5
7.0
6.5
6.0
5.5
5.0
4.5
4.0
3.5
3.0
2.5
2.0
1.5
1.0
ppm

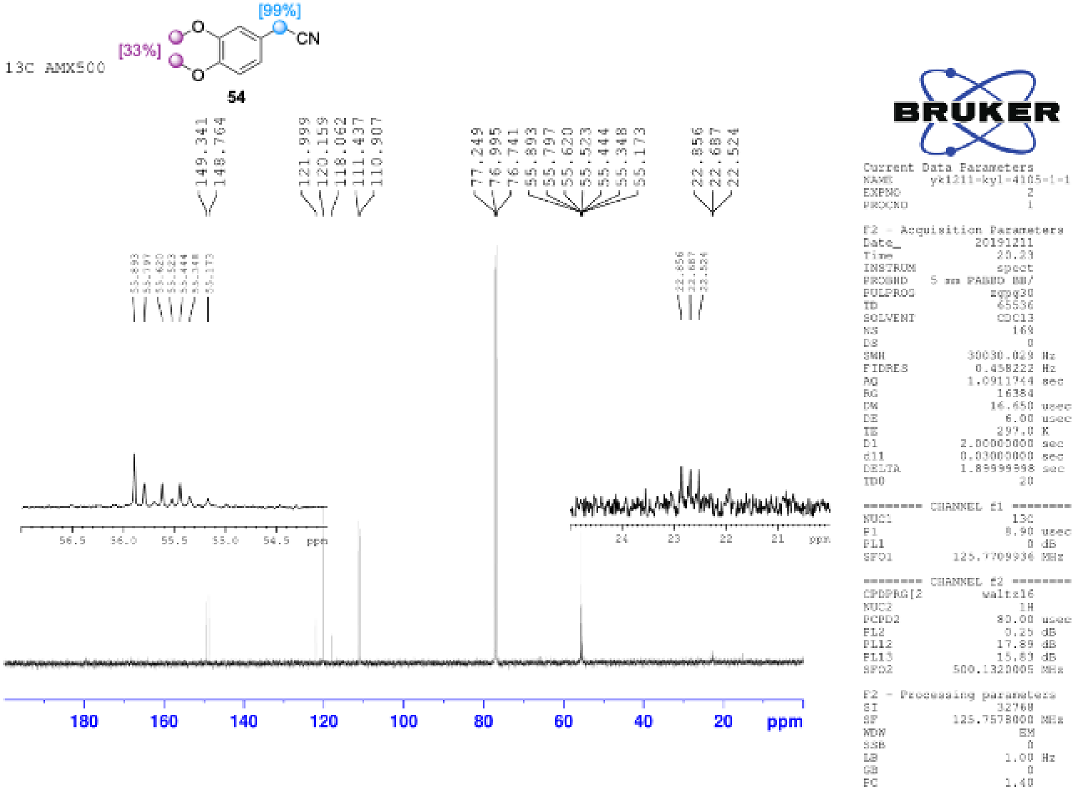
13C AMX500
[33%]
[99%]
CN
54
BRUKER
ppm

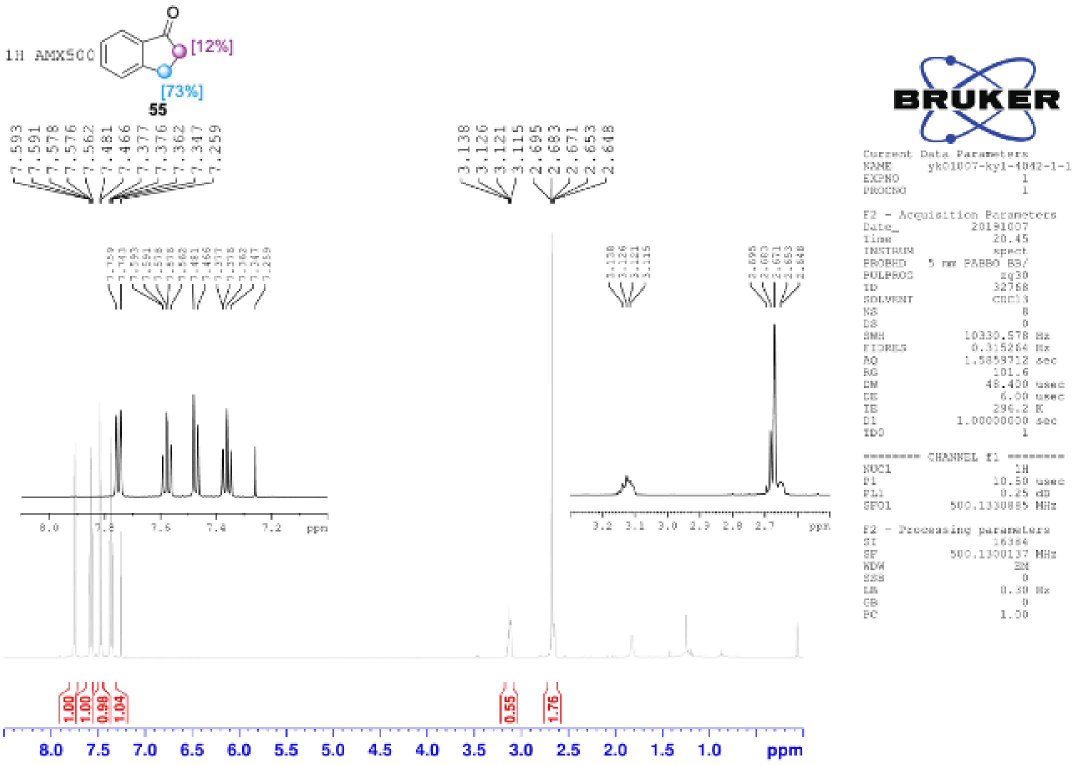
1H AMX500
[12%]
[73%]
55
BRUKER
ppm

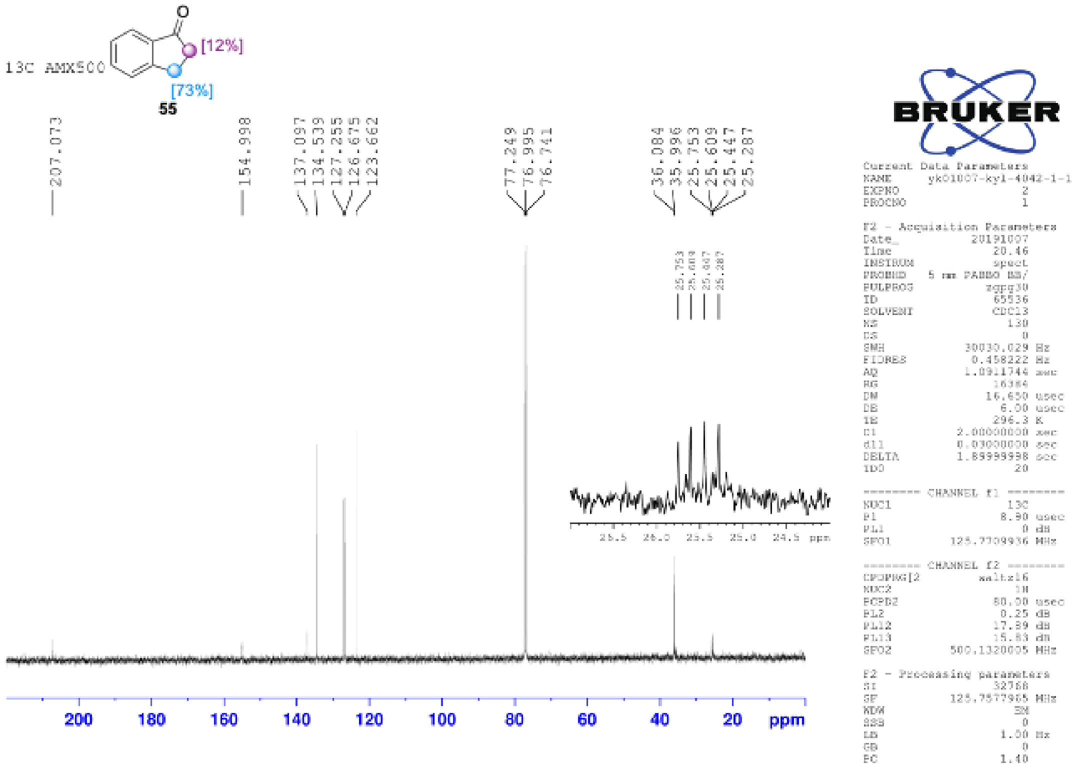
13C AMX500
[12%]
[73%]
55
207.073
154.998
137.097
134.539
127.255
126.675
123.662
77.249
76.995
76.741
36.084
35.996
25.753
25.609
25.447
25.287
BRUKER
200 180 160 140 120 100 80 60 40 20 ppm

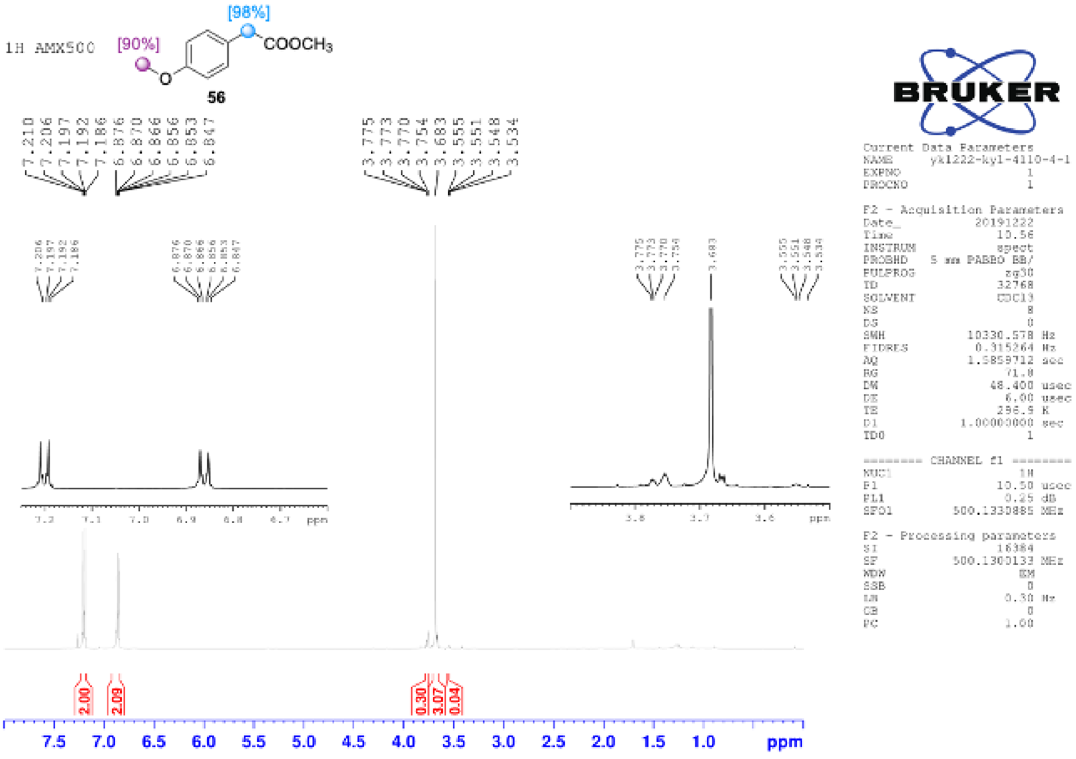
1H AMX500
[90%]
[98%]
COOCH3
56
7.210
7.206
7.197
7.192
7.186
6.876
6.870
6.866
6.856
6.853
6.847
3.775
3.773
3.770
3.754
3.683
3.555
3.551
3.548
3.534
BRUKER
2.00
2.09
0.30
3.07
0.04
7.5 7.0 6.5 6.0 5.5 5.0 4.5 4.0 3.5 3.0 2.5 2.0 1.5 1.0 ppm

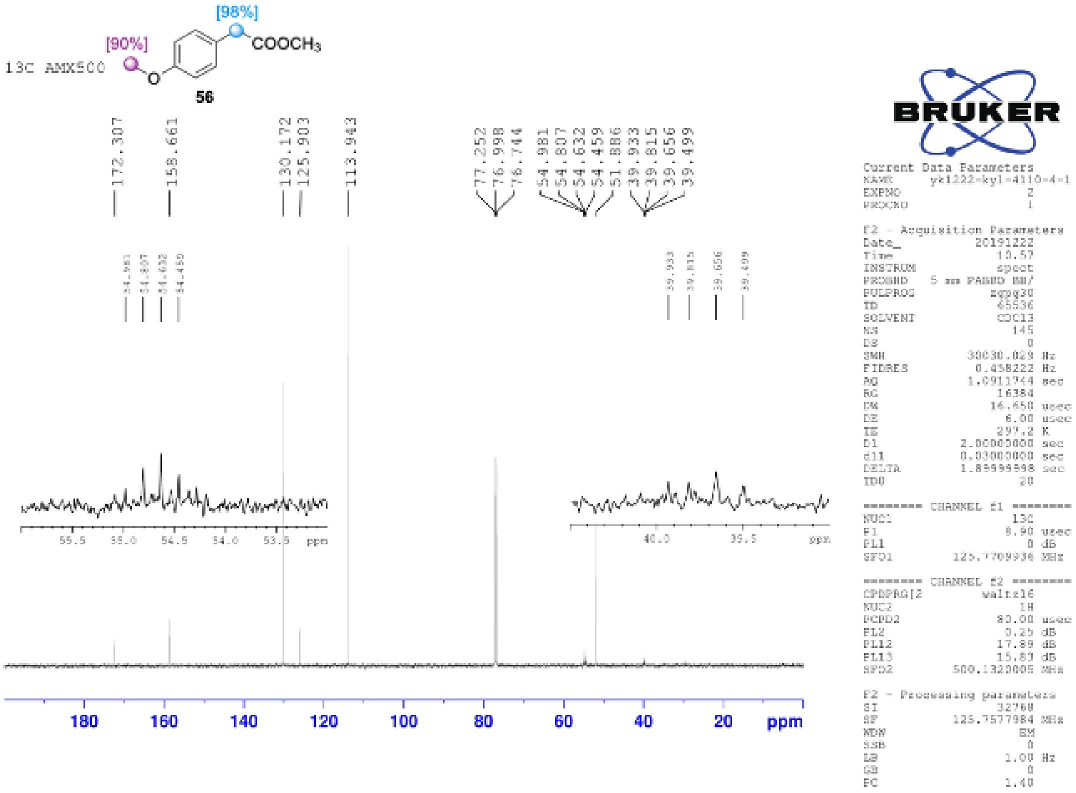
13C AMX500
[90%]
[98%]
COOCH3
56
172.307
158.661
130.172
125.903
113.943
77.252
76.998
76.744
54.981
54.807
54.632
54.459
51.886
39.933
39.815
39.656
39.499
BRUKER
Current Data Parameters
F2 - Acquisition Parameters
F2 - Processing parameters
180 160 140 120 100 80 60 40 20 ppm

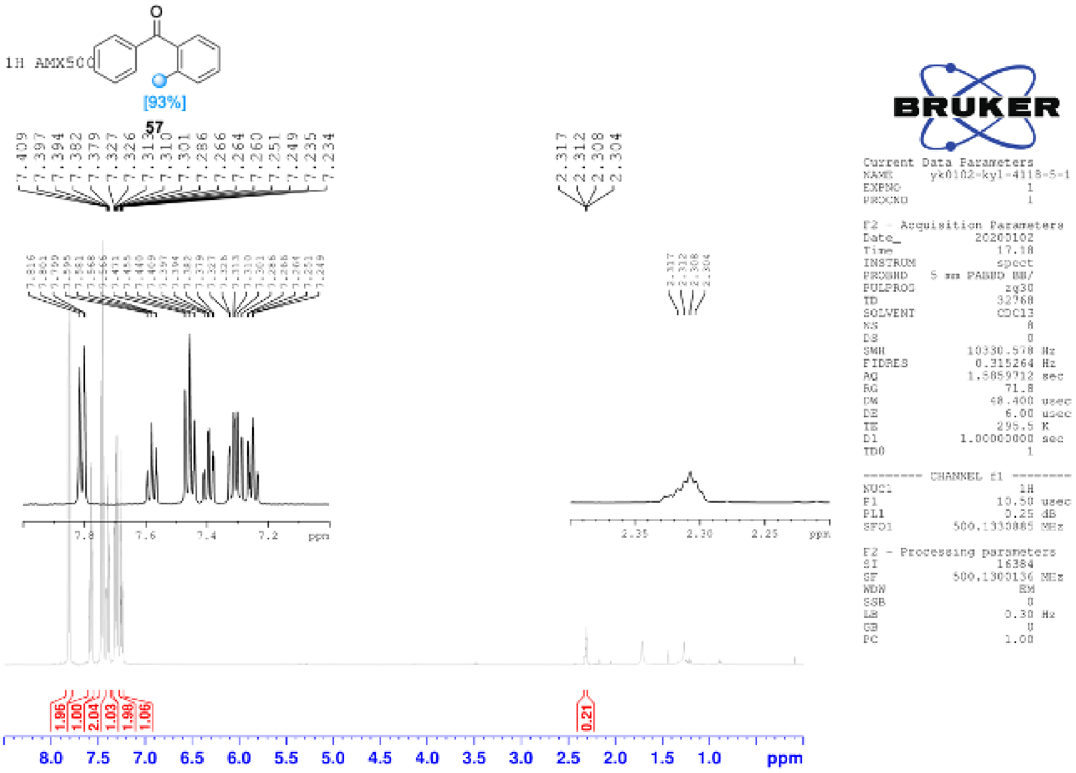
1H AMX500
O
[93%]
57
7.409
7.397
7.394
7.382
7.379
7.327
7.326
7.313
7.310
7.301
7.286
7.266
7.264
7.260
7.251
7.249
7.235
7.234
2.317
2.312
2.308
2.304
BRUKER
Current Data Parameters
F2 - Acquisition Parameters
F2 - Processing parameters
1.96
1.00
2.04
1.03
1.99
1.06
0.21
8.0 7.5 7.0 6.5 6.0 5.5 5.0 4.5 4.0 3.5 3.0 2.5 2.0 1.5 1.0 ppm

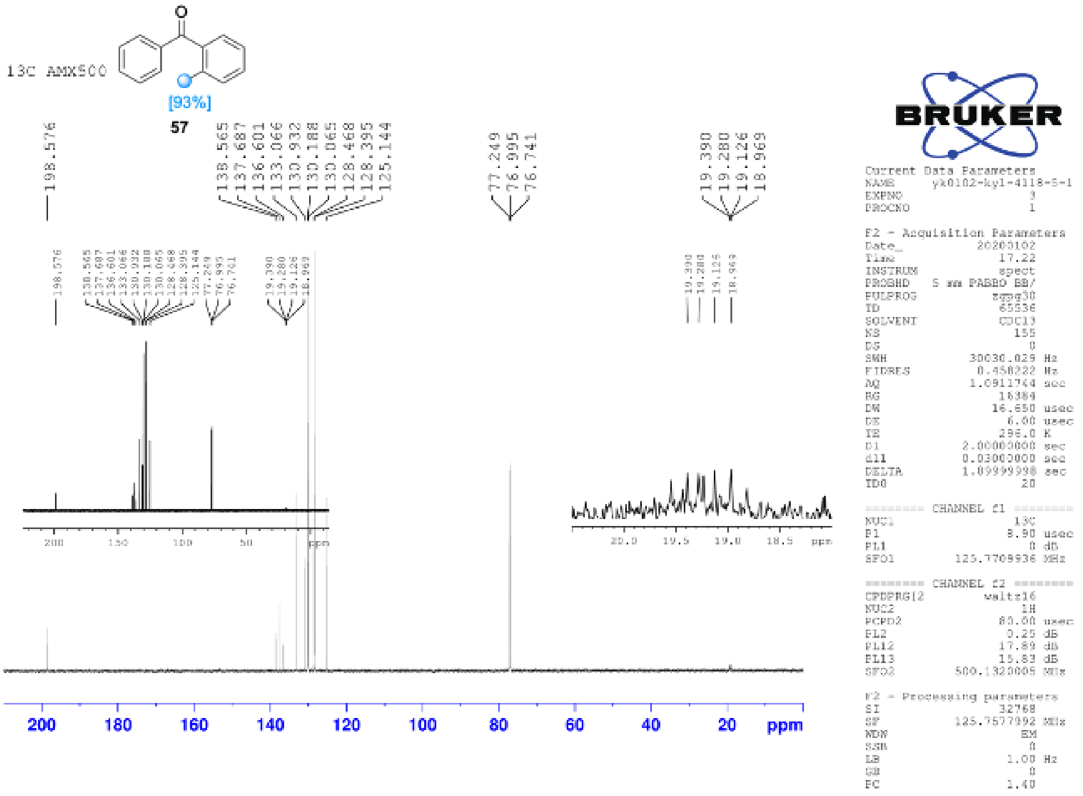
13C AMX500
O
[93%]
57
198.576
138.565
137.687
136.601
133.066
130.932
130.188
130.065
128.468
128.395
125.144
77.249
76.995
76.741
19.390
19.280
19.126
18.969
Current Data Parameters
NAME yk0102-kyl-4118-5-1
EXPNO 3
PROCNO 1
F2 - Acquisition Parameters
Date_ 20200102
Time 17.22
INSTRUM spect
PROBHD 5 mm PABBO BB/
PULPROG zgpg30
TD 65536
SOLVENT CDCl3
NS 155
DS 0
SWH 30030.029 Hz
FIDRES 0.458222 Hz
AQ 1.0911744 sec
RG 16384
DW 16.650 usec
DE 6.00 usec
TE 296.0 K
D1 2.00000000 sec
d11 0.03000000 sec
DELTA 1.89999998 sec
TD0 20
======== CHANNEL f1 ========
NUC1 13C
P1 8.90 usec
PL1 0 dB
SFO1 125.7709936 MHz
======== CHANNEL f2 ========
CPDPRG2 waltz16
NUC2 1H
PCPD2 80.00 usec
PL2 0.25 dB
PL12 17.89 dB
PL13 15.83 dB
SFO2 500.1320005 MHz
F2 - Processing parameters
SI 32768
SF 125.7577992 MHz
WDW EM
SSB 0
LB 1.00 Hz
GB 0
PC 1.40
200 180 160 140 120 100 80 60 40 20 ppm

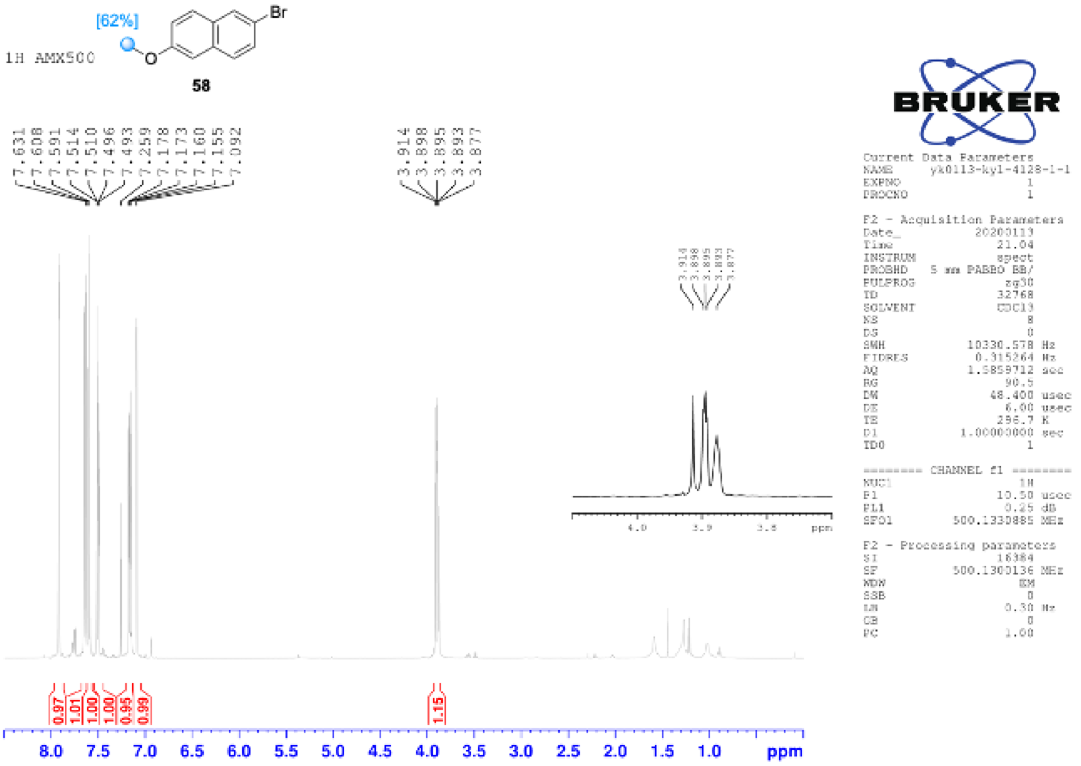
1H AMX500
[62%]
O
Br
58
7.631
7.608
7.591
7.514
7.510
7.496
7.493
7.259
7.178
7.173
7.160
7.155
7.092
3.914
3.898
3.895
3.893
3.877
Current Data Parameters
NAME yk0113-kyl-4128-1-1
EXPNO 1
PROCNO 1
F2 - Acquisition Parameters
Date_ 20200113
Time 21.04
INSTRUM spect
PROBHD 5 mm PABBO BB/
PULPROG zg30
TD 32768
SOLVENT CDCl3
NS 8
DS 0
SWH 10330.578 Hz
FIDRES 0.315264 Hz
AQ 1.5859712 sec
RG 90.5
DW 48.400 usec
DE 6.00 usec
TE 296.7 K
D1 1.00000000 sec
TD0 1
======== CHANNEL f1 ========
NUC1 1H
P1 10.50 usec
PL1 0.25 dB
SFO1 500.1330885 MHz
F2 - Processing parameters
SI 16384
SF 500.1300136 MHz
WDW EM
SSB 0
LB 0.30 Hz
GB 0
PC 1.00
0.97
1.01
1.00
1.00
0.95
0.99
1.15
8.0 7.5 7.0 6.5 6.0 5.5 5.0 4.5 4.0 3.5 3.0 2.5 2.0 1.5 1.0 ppm

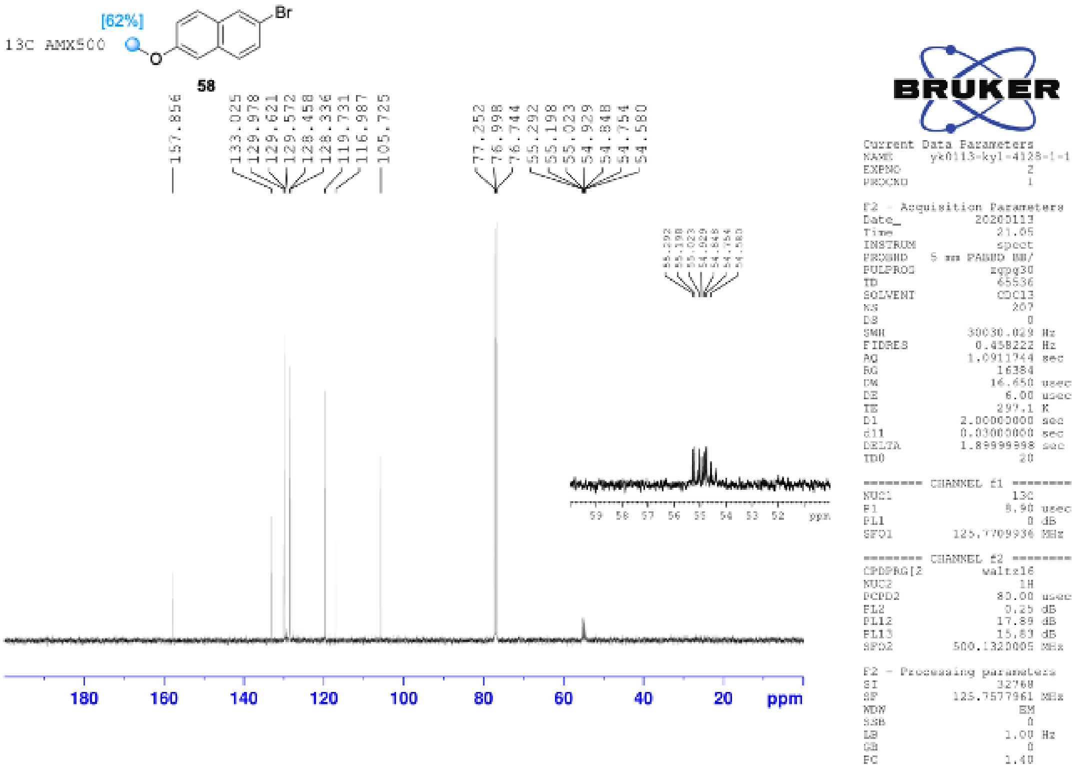
13C AMX500
[62%]
Br
O
58
157.856
133.025
129.978
129.621
129.572
128.458
128.336
119.731
116.987
105.725
77.252
76.998
76.744
55.292
55.198
55.023
54.929
54.848
54.754
54.580
BRUKER
180
160
140
120
100
80
60
40
20
ppm

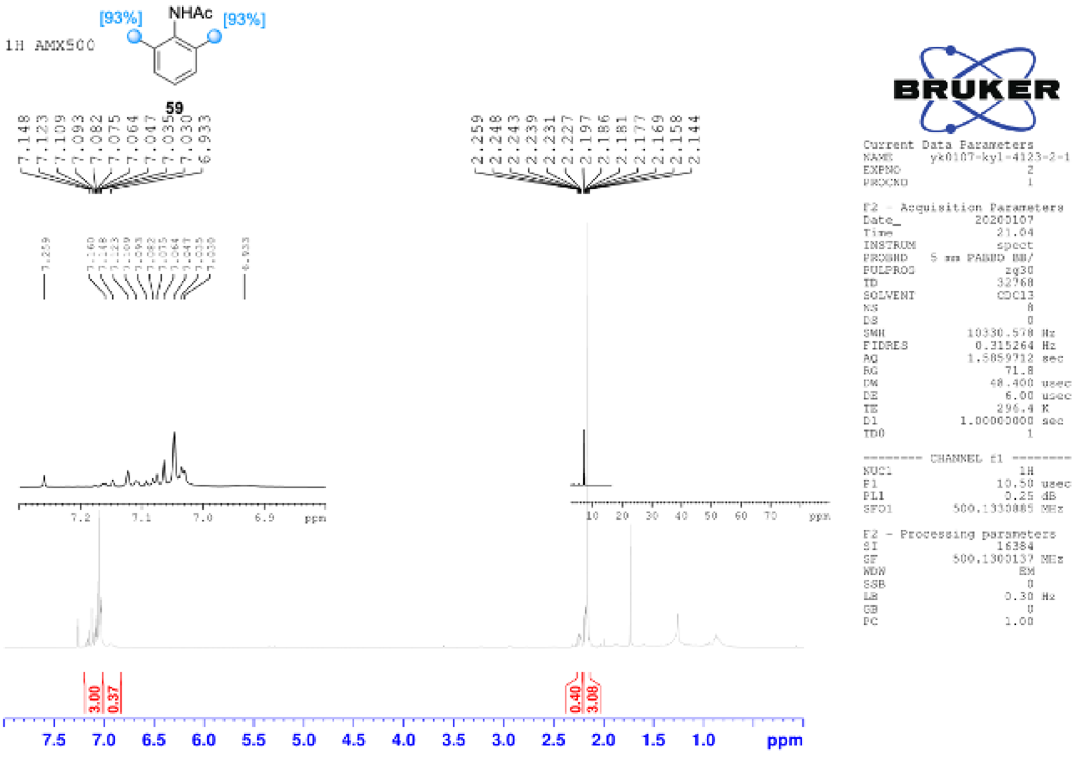
1H AMX500
[93%]
NHAc
[93%]
59
7.148
7.123
7.109
7.093
7.082
7.075
7.064
7.047
7.035
7.030
6.933
2.259
2.248
2.243
2.239
2.231
2.227
2.197
2.186
2.181
2.177
2.169
2.158
2.144
BRUKER
3.00
0.37
0.40
3.08
7.5
7.0
6.5
6.0
5.5
5.0
4.5
4.0
3.5
3.0
2.5
2.0
1.5
1.0
ppm

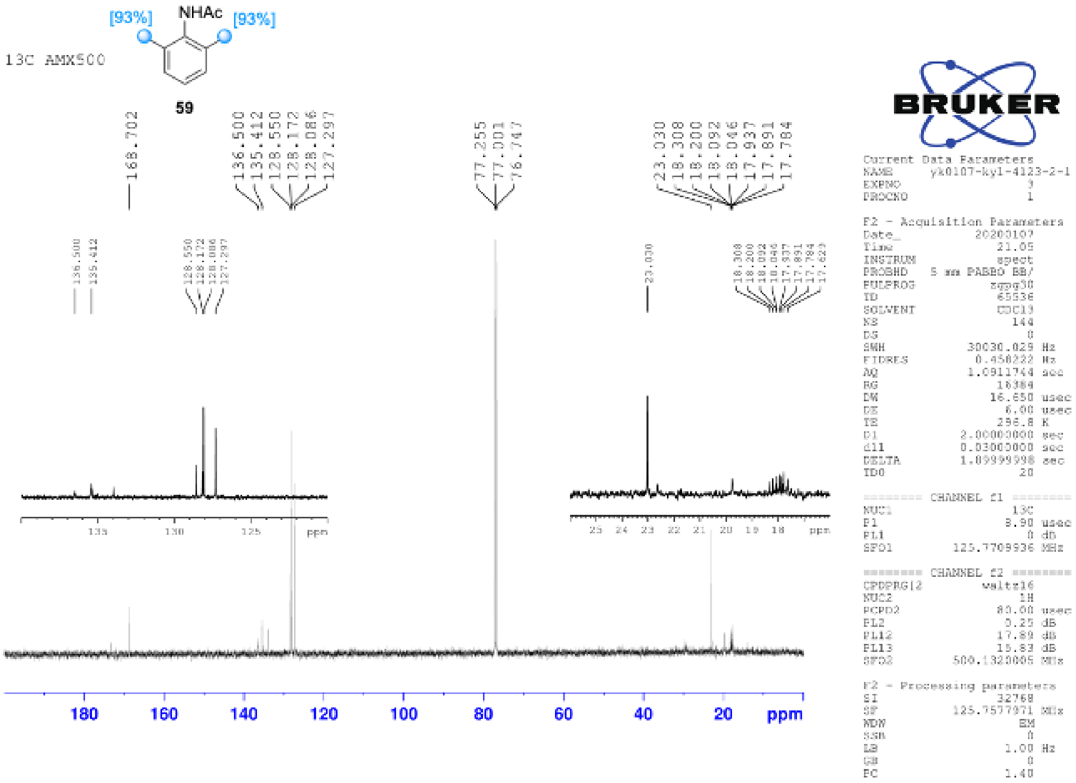
[93%]
NHAc
[93%]
59
13C AMX500
168.702
136.500
135.412
128.550
128.172
128.086
127.297
77.255
77.001
76.747
23.030
18.308
18.200
18.092
18.046
17.937
17.891
17.784
180
160
140
120
100
80
60
40
20
ppm
BRUKER
Current Data Parameters
NAME yk0107-kyl-4123-2-1
EXPNO 3
PROCNO 1
F2 - Acquisition Parameters
Date_ 20200107
Time 21.05
INSTRUM spect
PROBHD 5 mm PABBO BB/
PULPROG zgpg30
TD 65536
SOLVENT CDCl3
NS 144
DS 0
SWH 30030.029 Hz
FIDRES 0.458222 Hz
AQ 1.0911744 sec
RG 16384
DW 16.650 usec
DE 6.00 usec
TE 296.8 K
D1 2.00000000 sec
d11 0.03000000 sec
DELTA 1.89999998 sec
TD0 20
======== CHANNEL f1 ========
NUC1 13C
P1 9.90 usec
PL1 0 dB
SFO1 125.7709936 MHz
======== CHANNEL f2 ========
CPDPRG2 waltz16
NUC2 1H
PCPD2 80.00 usec
PL2 0.25 dB
PL12 17.89 dB
PL13 15.83 dB
SFO2 500.1320005 MHz
F2 - Processing parameters
SI 32768
SF 125.7577971 MHz
WDW EM
SSB 0
LB 1.00 Hz
GB 0
PC 1.40
